# Risk Factors for Delirium and Cognitive Decline Following Coronary Artery Bypass Grafting Surgery: A Systematic Review and Meta‐Analysis

**DOI:** 10.1161/JAHA.120.017275

**Published:** 2020-11-07

**Authors:** Danielle Greaves, Peter J. Psaltis, Daniel H. J. Davis, Tyler J. Ross, Erica S. Ghezzi, Amit Lampit, Ashleigh E. Smith, Hannah A. D. Keage

**Affiliations:** ^1^ Cognitive Ageing and Impairment Neurosciences Laboratory, Justice and Society Academic Unit University of South Australia Adelaide Australia; ^2^ Vascular Research Centre Lifelong Health Theme South Australian Health and Medical Research Institute Adelaide Australia; ^3^ Adelaide Medical School University of Adelaide Adelaide Australia; ^4^ Department of Cardiology Royal Adelaide Hospital Central Adelaide Local Health Network Adelaide Australia; ^5^ Medical Reasearch Council Unit for Lifelong Health and Ageing Unit at UCL London United Kingdom; ^6^ Academic Unit for Psychiatry of Old Age Department of Psychiatry University of Melbourne Melbourne Australia; ^7^ Department of Neurology Charité–Universitätsmedizin Berlin Berlin Germany; ^8^ Alliance for Research in Exercise, Nutrition and Activity Allied Health and Human Performance Academic Unit University of South Australia Adelaide Australia

**Keywords:** cognitive decline, coronary artery bypass grafting, delirium, meta‐analysis, Cardiovascular Surgery

## Abstract

**Background:**

Coronary artery bypass grafting (CABG) is known to improve heart function and quality of life, while rates of surgery‐related mortality are low. However, delirium and cognitive decline are common complications. We sought to identify preoperative, intraoperative, and postoperative risk or protective factors associated with delirium and cognitive decline (across time) in patients undergoing CABG.

**Methods and Results:**

We conducted a systematic search of Medline, PsycINFO, EMBASE, and Cochrane (March 26, 2019) for peer‐reviewed, English publications reporting post‐CABG delirium or cognitive decline data, for at least one risk factor. Random‐effects meta‐analyses estimated pooled odds ratio for categorical data and mean difference or standardized mean difference for continuous data. Ninety‐seven studies, comprising data from 60 479 patients who underwent CABG, were included. Moderate to large and statistically significant risk factors for delirium were as follows: (1) preoperative cognitive impairment, depression, stroke history, and higher European System for Cardiac Operative Risk Evaluation (EuroSCORE) score, (2) intraoperative increase in intubation time, and (3) postoperative presence of arrythmia and increased days in the intensive care unit; higher preoperative cognitive performance was protective for delirium. Moderate to large and statistically significant risk factors for acute cognitive decline were as follows: (1) preoperative depression and older age, (2) intraoperative increase in intubation time, and (3) postoperative presence of delirium and increased days in the intensive care unit. Presence of depression preoperatively was a moderate risk factor for midterm (1–6 months) post‐CABG cognitive decline.

**Conclusions:**

This meta‐analysis identified several key risk factors for delirium and cognitive decline following CABG, most of which are nonmodifiable. Future research should target preoperative risk factors, such as depression or cognitive impairment, which are potentially modifiable.

**Registration:**

URL: https://www.crd.york.ac.uk/prosp​ero/; Unique identifier: CRD42020149276.

**Nonstandard Abbreviations and Acronyms**
ACC: aortic cross‐clamp
CPB: cardiopulmonary bypass
SMD: standardized mean difference

**Clinical Perspective**

**What Is New?**

- This meta‐analysis is the first to comprehensively identify risk and protective factors for postoperative delirium and cognitive decline in patients who underwent coronary artery bypass grafting (CABG).
- Findings demonstrate that there are many risk and protective factors for delirium and cognitive decline post‐CABG, some of which are modifiable, such as depression, diabetes mellitus, hypertension, and cognitive impairment.
- The presence of preoperative depression was a common risk factor across outcomes, which at least doubled the risk of post‐CABG delirium in hospital and cognitive decline acutely and up to 6 months following surgery.

**What Are the Clinical Implications?**

- Risk and protective factors identified in this meta‐analysis could be used to improve delirium and cognitive decline risk prediction tools, leading to more accurate identification of at‐risk patients undergoing CABG, improving care and prognosis.
- Findings can inform the design of future intervention trials aimed at reducing the incidence of delirium and cognitive decline post‐CABG, by targeting identified modifiable risk factors.

Coronary artery bypass grafting (CABG) surgery is the main treatment for multivessel coronary disease and remains one of the most common cardiac procedures worldwide.^1^, ^2^ CABG has low mortality rates, and improves coronary vascularization and cardiac function.^3^ However, CABG is associated with high rates of postoperative cognitive impairments, including delirium.^4^, ^5^, ^6^

A recent meta‐analysis investigating post‐CABG cognitive outcomes (cross‐sectional approach by percentage at specific time points)^4^ revealed postoperative cognitive impairment or decline was prevalent in 43% of patients up to 4 days, and remains high (39%) up to 1 month post‐CABG. This reduces in the midterm (6–12 months) following CABG to ≈25% and increases up to nearly 40% in the long‐term (1–5 years). The presence of delirium (an acute and fluctuating syndrome of deficits in attention and arousal) was apparent in 24% of patients, up to 1 week post‐CABG, when a standardized tool was used alongside clinical criteria.^4^

The presence of cognitive decline following CABG is associated with increased depression risk and decreased quality of life, functional capacity, and the ability to perform activities of daily living.^7^ Delirium presence in older adults is associated with increased mortality, length of stay (LOS), hospital readmissions, as well as cognitive decline and dementia, along with reduced quality of life.^8^, ^9^, ^10^, ^11^ Research attempting to prevent these post‐CABG cognitive outcomes has been largely unsuccessful, including pharmacological, anesthetic intervention, and surgical techniques.^12^, ^13^, ^14^, ^15^, ^16^ There has been some evidence of therapeutic effect for advanced surgical methods, such as hypothermia and increasing systemic perfusion intraoperatively.^17^ However, the expertise and technology needed are not routinely available.

Understanding risk and protective factors for delirium and cognitive decline post‐CABG has critical clinical implications, including more precise targeting of preoperative and perioperative interventions and the development of a sensitive risk screening tool for these outcomes. The use of a prediction tool for delirium and cognitive decline in a post‐CABG setting could lead to earlier intervention opportunities, greater prognosis, and, in turn, better patient management.

Previous meta‐analyses of all surgical type cardiac patients have provided greater depth of knowledge surrounding the effects of surgery method on cognitive decline (on versus off pump)^15^, ^16^ and the effect of pharmacological and anesthetic interventions on postoperative delirium.^18^, ^19^ Specific risk or protective factors for cognitive outcomes (delirium and cognitive decline) have not been comprehensively investigated through meta‐analysis in patients undergoing CABG. In addition, no meta‐analysis has investigated the time course of effects for risk factors in relation to cognitive decline following CABG, especially in the long‐term (>12 months). This systematic review and meta‐analysis aims to investigate risk and protective factors for the following: (1) post‐CABG delirium (1–7 days) and (2) post‐CABG cognitive decline across multiple time points: short‐term (immediately postoperatively up to 1 month), midterm (1–6 months postoperatively), and long‐term (12–15 months postoperatively).

## Methods

The protocol for this systematic review and meta‐analysis was registered and published with the international prospective register of systematic reviews (PROSPERO) (registration number: CRD42020149276). This article is reported in accordance to the Preferred Reporting Items for Systematic Reviews and Meta‐Analysis guidelines.^20^ The data that support the findings of this study are available from the corresponding author on reasonable request.

### Search Strategy

We updated a search from a published meta‐analysis.^4^ We searched Medline, PsycINFO, EMBASE, and the Cochrane databases using the Ovid platform when possible. Searches of all databases were last performed on March 26, 2019. Search terms and medical subject headings used were as follows: (Coronary Artery Bypass/ OR “coronary artery bypass” OR CABG) AND (Cognition/ OR Delirium/ OR Dementia/ OR Alzheimer Disease/ OR Neuropsychological Tests/ OR Cognit* OR Deliri* OR Dementia* OR Alzheimer* MCI or “mild cognitive impairment*” OR “mild‐cognitive impairment*” OR neuropsycholo* OR POCD OR “postoperative cognitive” OR “post‐operative cognitive” OR MMSE OR "mini‐mental state examination” OR “cerebral function” OR neurocognit* OR encephalopath*). Article selection and data extraction, of the updated search, were undertaken by at least 2 reviewers (between D.G., E.S.G., and T.J.R.), with disagreements resolved by consensus.

### Study Eligibility

Inclusion criteria were as follows: peer‐reviewed, full‐text, English‐language studies that reported usable risk or protective factor data of those who had undergone CABG surgery (including CABG plus concomitant surgeries). Studies needed to report a cognitive outcome (using a standardized test result, neuropsychological battery, or a clinical diagnosis) for presence of delirium versus no delirium or cognitive decline versus no cognitive decline, and include usable data for at least one risk factor.

Exclusion criteria included the following: case series (n<5), dissertations, book chapters, protocol articles, reviews, news articles, conference abstracts, letters to the editor, editorials, and comment publications; and studies with no description of their operationalization (or definition used for categorizing participants with cognitive decline/delirium) or incomplete reporting in respect to risk factor data.

All possible risk/protective factors were tallied for presence across eligible studies (eg, data reported within text or within a table split by cognitive outcome or results of measures of association, such as odds ratios [ORs]). Unique risk factors that were reported in >10 studies (across delirium and cognitive decline) were included in this review. A list of these factors was circulated to academic clinicians (coauthors P.J.P. and D.H.J.D.) to ensure that no clinically relevant factors had been missed. This led to the additional extraction of delirium as a risk factor for cognitive decline (although only present in 3 studies). Following this, factors were categorized as follows: preoperative, intraoperative, or postoperative. Studies that did not report information pertaining to the target risk factors analyzed within the study (eg, studies reporting data related to hematocrit, height, or sepsis) were subsequently excluded (categorized as inappropriate data). In addition, if multiple studies investigated the same cohort, duplicate samples were excluded.

### Quality Assessment

Study design and reporting quality were assessed by at least 2 reviewers (between D.G., E.S.G., and T.J.R.), with disagreements resolved by consensus. An adapted tool was used, on the basis of 2 existing assessment checklists,^21^, ^22^ where higher scores indicated greater overall quality (0–12) (Data S1).

### Data Extraction

Data extracted from each included study consisted of: country, sample size, age, sex, cognitive decline/delirium assessment criteria, and risk factor data relative to time periods and cognitive outcome (delirium versus no delirium): 1 to 7 days postoperatively; postoperative cognitive decline versus no decline: short‐term (immediately postoperatively up to 1 month), midterm (1–6 months postoperatively), and long‐term (12–15 months postoperatively). There may be a small degree of overlap between the outcomes of delirium and acute cognitive decline, yet this overlap is representative of the population at this time point. Many of the studies included in this meta‐analysis did not explicitly aim to assess risk factors for these cognitive outcomes through inferential statistical analyses. Yet, these studies still reported extractable descriptive data related to the cognitive outcome (eg, table presenting counts or mean and SD for preoperative, intraoperative, and postoperative variables, split by cognitive outcome). As fewer articles reported data as a result of an inferential statistical analysis, the extraction of descriptive data was prioritized. For each risk factor, descriptive data (eg, mean and SD/event rates) were extracted when available. In the absence of descriptive data, the results of inferential statistical analyses (eg, ORs) were extracted. To increase the consistency within our analyses, only univariate (or unadjusted) data were extracted, as the number and type of covariates used within risk factor analyses varied greatly across studies. When data were reported and extracted as median and interquartile range values, they were converted to mean and SD values.^23^, ^24^ Only data pertaining to risk/protective factors could be extracted for each cognitive outcome for the time periods reported in identified studies. There were substantially fewer articles within the literature that investigate midterm and long‐term cognitive decline, compared with delirium and acute cognitive decline. Therefore, fewer risk factors could be investigated for midterm and long‐term cognitive decline. It may be the case that there are important risk factors for these time points that we were unable to identify herein with our approach.

### Statistical Analysis

Demographic data were calculated from the reported preoperative samples. The I^2^ statistic was used to express the proportion of between‐study heterogeneity out of total variance and was classified as low (I^2^=25%–50%), moderate (I^2^=50%–75%), or high (I^2^≥75%), using classification criteria suggested by Higgins et al.^25^ Total between‐study variance was quantified using τ^2^. All analyses were based on random‐effects model. Before data analyses, checks were conducted to detect extreme outliers. Effect size estimates that fell an abnormally large distance from other estimates (mainly because of separation or quasi‐separation for a given outcome) were excluded. This process did not exclude the remaining study data from remaining risk factor analyses.

All analyses were performed in Comprehensive Meta‐Analysis software (version 3). A result was considered statistically significant when *P*<0.05. Each risk or protective factor was analyzed separately and, therefore, independence from other factors cannot be assumed. Separate random effect meta‐analyses were used to estimate pooled OR for categorical risk factor data and mean difference or standardized mean difference (SMD) for continuous risk factor data, comparing cognitive outcomes (delirium versus no delirium or cognitive decline versus no cognitive decline) post‐CABG. A risk or protective factor was meta‐analyzed when data from ≥2 studies were available for the analysis. All meta‐analyses were conducted on univariate data (no multivariate data were extracted) and therefore should be interpreted as unadjusted pooled estimates. The SMD was also calculated to provide a supplementary common effect size across pooled estimates (Tables S1 through S4). SMD values can be interpreted using the same cutoff as Cohen *d,* where ≥0.20, ≥0.50, and ≥0.80 are considered as small, moderate, and large, respectively.^26^ For cognitive decline post‐CABG, analyses were conducted for each time point: short‐term (immediately postoperatively up to 1 month), midterm (1–6 months postoperatively), and long‐term (12–15 months postoperatively). Some of the extracted predictor variables were presented as both categorical and continuous data across articles (eg, education >12 years [categorical] or total years of education [continuous]). Others provided data that could be sorted into multiple categories (eg, preoperative cognitive test scores): (1) different cognitive tests used between studies (SMD used) or (2) the same test used between studies, such as Mini‐Mental State Examination (mean difference used). In these cases, subanalyses were performed for each data format or category, for each risk factor. For statistically significant results, small study effect was examined by visually inspecting funnel plots of effect size versus SE.^27^ When at least 10 studies were available for analyses, small study effect was formally assessed using the Egger test of the intercept.^28^ When there was evidence for small study effect (1‐tailed *P*<0.1), we used the Duvall and Tweedie^29^ trim and fill method to quantify the extent of potential bias. When there were <10 studies, we performed sensitivity analyses by removing outliers.

Random‐effects meta‐regressions (using mean age as a covariate within the analysis) were performed to investigate whether age was related to the pooled effect estimates. Only analyses containing both risk factor and age data of ≥10 studies, as stated in recent Cochrane guidelines,^30^ were interpreted. We also performed stratified random‐effects subgroup analyses to investigate any possible effects of diagnostic approach for delirium (inclusion of a standardized instrument versus none) for each risk factor. For this, stratified random‐effects meta‐analyses were performed for each risk or protective factor variable relative to (1) studies using a standardized instrument (eg, Confusion Assessment Method or the Delirium Rating Scale) to inform the reference standard and (2) studies not using a specific instrument. Therefore, 2 subgroup meta‐analyses were conducted for each risk factor variable (1 of studies using a diagnostic tool and 1 of studies using no tool), allowing comparison of the pooled estimates. Subgroup analyses investigating differing methods of classifying cognitive decline were not conducted because of the limited numbers of articles across most time points.

## Results

**Figure 1:**
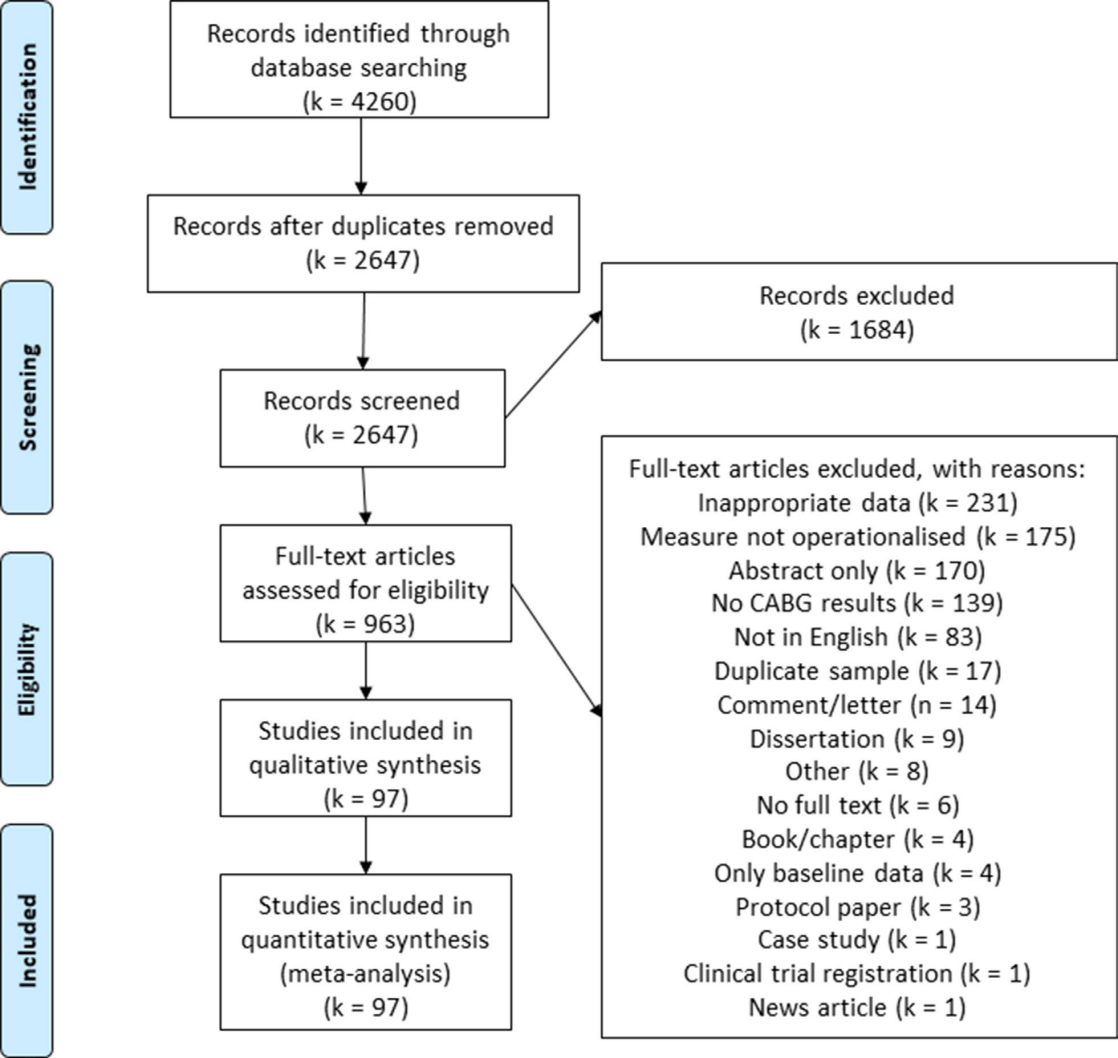
Preferred Reporting Items for Systematic Reviews and Meta‐Analysis flow diagram. CABG indicates coronary artery bypass grafting.

The search identified 4260 articles, of which 2647 records were screened by title and abstract, following duplicate removal. Full‐text screening was conducted on 963 articles; of these, 97 were included in this review (Figure 1, see Table S5 for articles excluded and rationale for exclusion, at full‐text review stage).

The 97 included studies were published across 4 decades, with 3, 7, 38, and 49 studies published in the 1980s, 1990s, 2000s, and 2010s, respectively. Of the included studies, 17 were conducted in the United States, 13 in Japan, 9 in Canada, 8 in Australia, and 6 each in China and the Netherlands. The remaining 38 studies were conducted across 22 individual countries. The included articles comprised data from 60 479 patients, with individual study sample sizes ranging from 8 to 14 262. The mean age of patients across included studies was 64.54 years, and 68.55% of patients were men (calculated only from studies with available data). The included studies were of good quality on the basis of the critical appraisals, ranging from 4 to 12, with a median study score of 10 (of 12) and interquartile range of 8 to 11.5. No studies were excluded from the analysis on the basis of their quality (see Table S6 for individual study information).

Preanalysis checks for extreme outliers resulted in data from 3 studies being excluded from separate analyses (delirium analyses of: presence of depression, kidney injury, and LOS in intensive care unit [ICU]); however, these studies remained within other analyses and therefore were not excluded from this article.

### Delirium

Data from 48 individual studies were used within 33 analyses (including subcategory analyses), investigating 27 separate risk or protective factors for delirium presence post‐CABG. Across the analyses, heterogeneity of statistically significant results spanned from low to high (I^2^ range, 0–98.40; τ^2^ range, 0–325.89) (see Table S1 for results of each meta‐analysis and Figure S1 for forest plots). Potential small‐study effect was found in 2 analyses (preoperative age and European System for Cardiac Operative Risk Evaluation (EuroSCORE)), where trim and fill estimation led to decreases in effect size (see Figure S2 for funnel plots and small study effect investigation).

**Figure 2:**
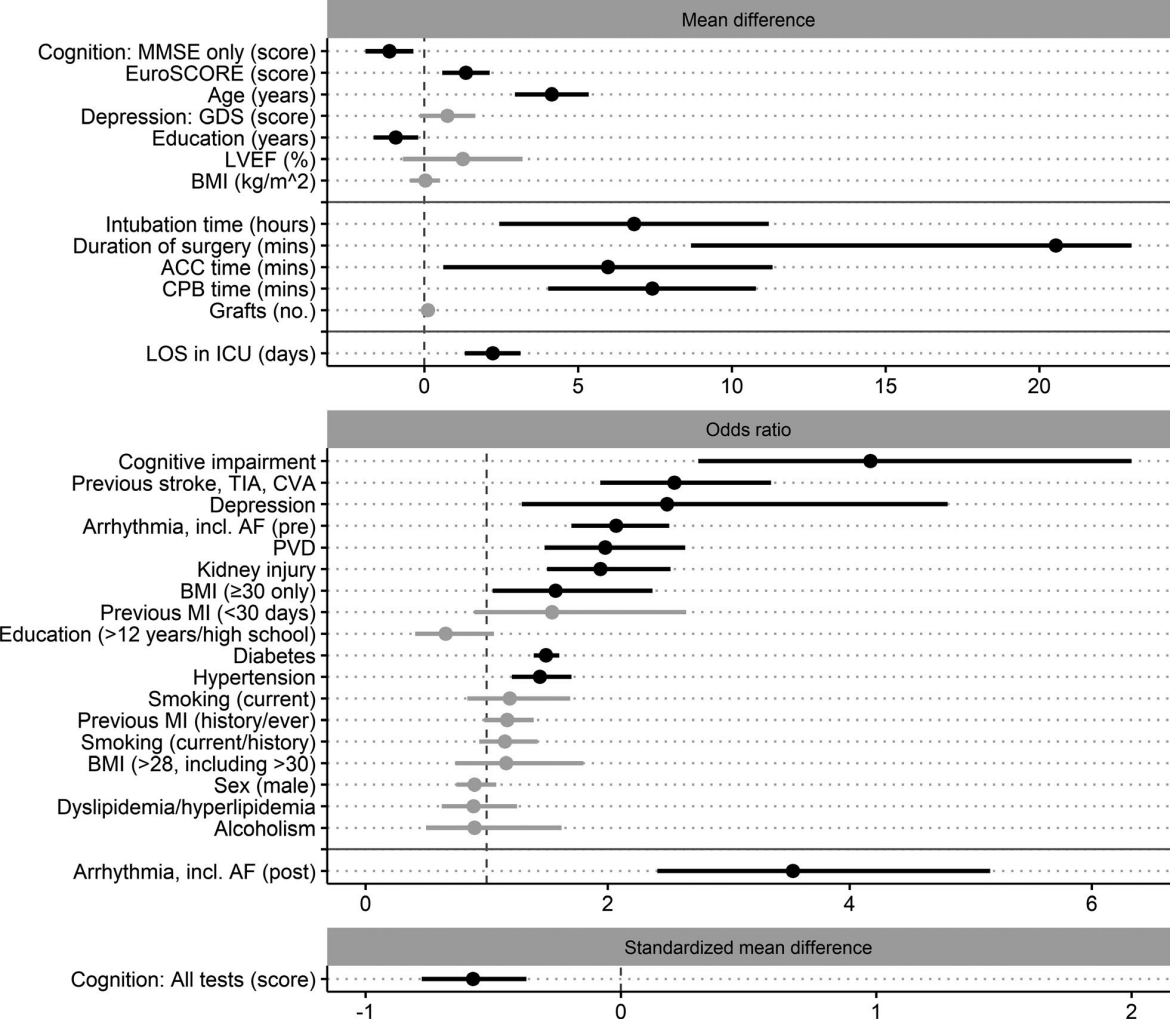
Forest plots of pooled estimates for risk or protective factors of post–coronary artery bypass grafting delirium. Factors grouped according to the primary pooled estimate of the analysis (mean difference [MD], odds ratio, or standardized MD [SMD]), where solid gray horizontal lines indicate separation of preoperative, intraoperative, and postoperative factors and dashed gray vertical lines divide protective (left side) and risk (right side) factor estimates. The pooled estimates are ordered by the common calculated effect size (SMD) from largest to smallest (largest at the top). Estimates that are black represent statistically significant factors; those that are gray did not reach statistical significance. The scale for all continuous variables (MD and SMD plots) is listed within each factor name. The CIs for duration of surgery extend further than the visible portion of the figure. This was not shown to allow appropriate visibility of all pooled estimates. ACC indicates aortic cross‐clamp; AF, atrial fibrillation; BMI, body mass index; CPB, cardiopulmonary bypass; CVA, cerebrovascular attack; GDS, Geriatric Depression Scale; ICU, intensive care unit; LOS, length of stay; LVEF, left ventricular ejection fraction; MI, myocardial infarction; MMSE, Mini‐Mental State Examination; PVD, peripheral vascular disease; and TIA, transient ischemic attack.

Statistically significant preoperative risk factors of developing delirium post‐CABG, from largest to smallest effect size, were: the presence of cognitive impairment, stroke history, depression, arrhythmia, including atrial fibrillation (AF), peripheral vascular disease, kidney injury/disease, body mass index >30 kg/m^2^, diabetes mellitus, and hypertension, along with continuous risk factors of higher EuroSCORE and older age. Statistically significant intraoperative risk factors, from largest to smallest effect size, were increased intubation time (hours), duration of surgery (minutes), aortic cross‐clamp (ACC) time (minutes), and cardiopulmonary bypass (CPB) time (minutes). Statistically significant postoperative risk factors, from largest to smallest effect size, were: increased LOS in the ICU (days) and the presence of arrhythmia, including AF. Statistically significant protective factors for developing delirium post‐CABG were higher preoperative cognition test scores and years of education (Table S1 and Figure 2).

Preoperative factors that did not reach statistical significance were: the presence of alcoholism, body mass index >28 kg/m^2^, dyslipidemia/hyperlipidemia, >12 years of education, male sex, previous myocardial infarction, and previous/current smoking; and continuous factors of higher body mass index, depression score, and left ventricular ejection fraction. With respect to intraoperative factors, number of grafts did not reach statistical significance (Table S1 and Figure 2).

Subgroup analyses investigating the effect of diagnostic criteria for delirium (studies using standardized measurement tool along with diagnostic criteria versus studies using no tool) revealed no meaningful differences for any risk factors, with CIs overlapping for all analyses (Table S7). Meta‐regressions with mean age as a model factor (covariate) revealed statistically significant results for risk factors of ACC time (age: β=−1.33, Z=−2.49, *P*=0.013, *R* ^2^=0.50) and LOS in ICU (age: β=−0.22, Z=−1.99, *P*=0.046, *R* ^2^=0.10). These results suggest that as the mean age of the study sample increases, the delirium risk associated with ACC time and LOS in ICU decreases. The results also suggest that 50% (for ACC time) and 10% (for LOS in ICU) of the variance in delirium presence relating to these risk factors can be attributed to age.

### Acute Cognitive Decline (Immediately to 1‐Month Post‐CABG)

**Figure 3:**
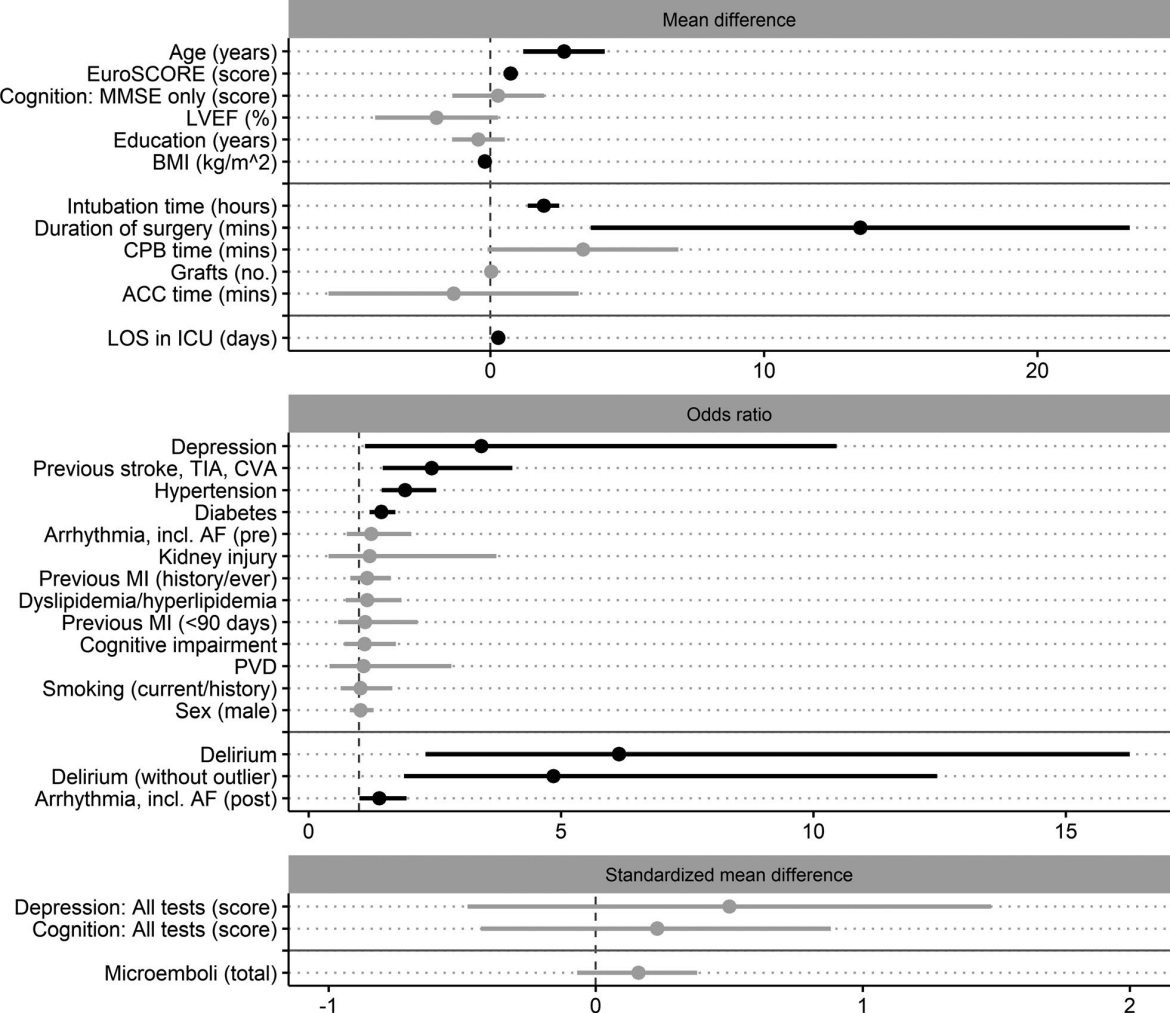
Forest plots of pooled estimates for risk or protective factors of post–coronary artery bypass grafting acute cognitive decline. Factors grouped according to the primary pooled estimate of the analysis (mean difference [MD], odds ratio, or standardized MD [SMD]), where solid gray horizontal lines indicate separation of preoperative, intraoperative, and postoperative factors and dashed gray vertical lines divide protective (left side) and risk (right side) factor estimates. The pooled estimates are ordered by the common calculated effect size (SMD) from largest to smallest (largest at the top). Estimates that are black represent statistically significant factors; those that are gray did not reach statistical significance. The scale for all continuous variables (MD and SMD plots) is listed within each factor name. ACC indicates aortic cross‐clamp; AF, atrial fibrillation; BMI, body mass index; CPB, cardiopulmonary bypass; CVA, cerebrovascular attack; ICU, intensive care unit; LOS, length of stay; LVEF, left ventricular ejection fraction; MI, myocardial infarction; MMSE, Mini‐Mental State Examination; PVD, peripheral vascular disease; and TIA, transient ischemic attack.

Data from 35 individual studies were used within 30 analyses (including subcategory analyses), investigating 25 separate risk or protective factors for the presence of cognitive decline acutely (immediately up to 1 month) post‐CABG. Across the analyses, heterogeneity of statistically significant results spanned from low to high (I^2^ range, 0–92.85; τ^2^ range, 0–32.28) (see Table S2 for results of each meta‐analysis and Figure S3 for forest plots). Potential small study effect was found in 2 analyses. Trim and fill estimation for preoperative age led to a decrease in effect size (see Figure S4 for funnel plots and small study effect investigation). A sensitivity analysis was performed for postoperative delirium (removal of outlier), which resulted in a decrease in effect size (Table S2 and Figure 3).

Statistically significant preoperative risk factors for acute post‐CABG cognitive decline, from largest to smallest effect size, were: the presence of depression, stroke history, hypertension, and diabetes mellitus, along with continuous risk factors of older age and higher EuroSCORE. Statistically significant intraoperative continuous risk factors, from largest to smallest effect size, were increased intubation time (hours) and duration of surgery (minutes). Statistically significant postoperative risk factors, from largest to smallest effect size, were: the presence of delirium and arrhythmia, including AF, and the continuous risk factor of increased LOS in the ICU (days). Higher body mass index was a statistically significant protective factor for acute post‐CABG cognitive decline (Table S2 and Figure 3).

Preoperative factors that did not reach statistical significance were the presence of arrhythmia, including AF, cognitive impairment, dyslipidemia/hyperlipidemia, male sex, kidney injury/disease, previous myocardial infarction, peripheral vascular disease, and previous/current smoking; and continuous factors of higher cognitive test score, depression score, years of education, and lower left ventricular ejection fraction. Intraoperative factors that did not reach statistical significance were increase in ACC time (minutes), CPB time (minutes), number of grafts, and total microemboli count (Table S2 and Figure 3).

Meta‐regressions revealed that 49% of the variance in acute cognitive decline for the risk factor of increased CPB time (age: β=−0.88, Z=−2.24, *P*=0.025, *R* ^2^=0.49) can be attributed to age. These results suggest that as the mean age of the study sample increases, the risk of cognitive decline associated with CPB time decreases.

### Midterm Cognitive Decline (1–6 Months Post‐CABG)

**Figure 4:**
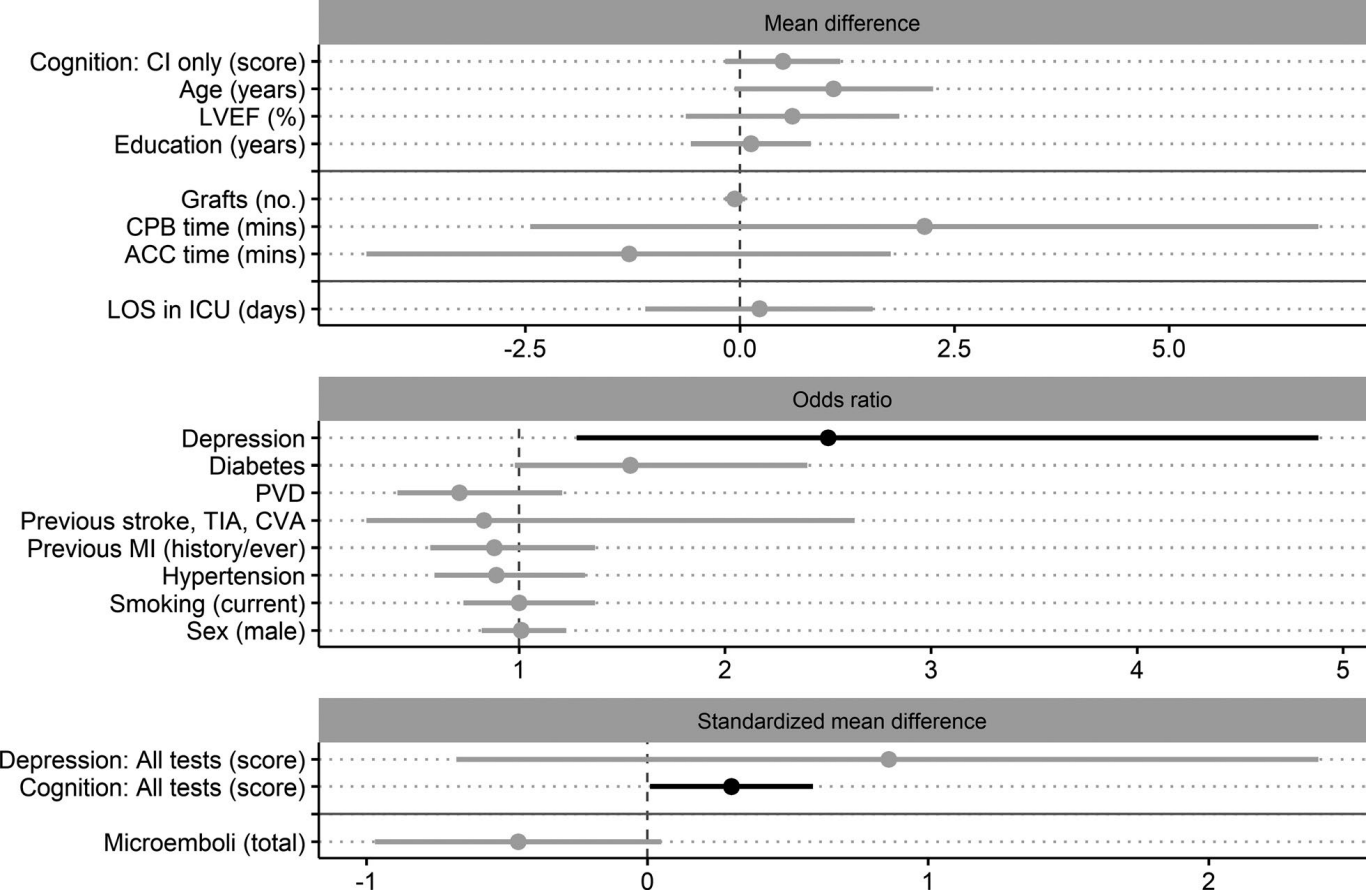
Forest plots of pooled estimates for risk or protective factors of post–coronary artery bypass grafting midterm cognitive decline. Factors grouped according to the primary pooled estimate of the analysis (mean difference [MD], odds ratio, or standardized MD [SMD]), where solid gray horizontal lines indicate separation of preoperative, intraoperative, and postoperative factors and dashed gray vertical lines divide protective (left side) and risk (right side) factor estimates. The pooled estimates are ordered by the common calculated effect size (SMD) from largest to smallest (largest at the top). Estimates that are black represent statistically significant factors; those that are gray did not reach statistical significance. The scale for all continuous variables (MD and SMD plots) is listed within each factor name. ACC indicates aortic cross‐clamp; CI, cognitive index score; CPB, cardiopulmonary bypass; CVA, cerebrovascular attack; ICU, intensive care unit; LOS, length of stay; LVEF, left ventricular ejection fraction; MI, myocardial infarction; PVD, peripheral vascular disease; and TIA, transient ischemic attack.

Data from 24 individual studies were used within 19 analyses (including subcategory analyses), investigating 17 separate risk or protective factors for the presence of cognitive decline in the midterm (1–6 months) post‐CABG. Across the analyses, heterogeneity of statistically significant results spanned from low to moderate (I^2^ range, 0–68.84; τ^2^ range, 0–0.04) (see Table S3 for results of each meta‐analysis and Figure S5 for forest plots). Two analyses revealed statistically significant results, with no indication of small study effect (Figure S6). Preoperative depression and higher cognitive test scores (across all tests) were risk factors for midterm post‐CABG cognitive decline (Table S3 and Figure 4).

Preoperative factors that did not reach statistical significance were the presence of diabetes mellitus, male sex, hypertension, previous myocardial infarction, stroke history, peripheral vascular disease, and current smoking; and continuous factors of higher age, cognitive test score (when using cognitive index), depression score, years of education, and left ventricular ejection fraction. No intraoperative or postoperative factors reached statistical significance, including increase in ACC time (minutes), CPB time (minutes), number of grafts, total microemboli count, and LOS in ICU (days) (Table S3 and Figure 4). No meta‐regressions investigating the influence of age were significant for this time point.

### Long‐Term Cognitive Decline (12–15 Months Post‐CABG)

**Figure 5:**
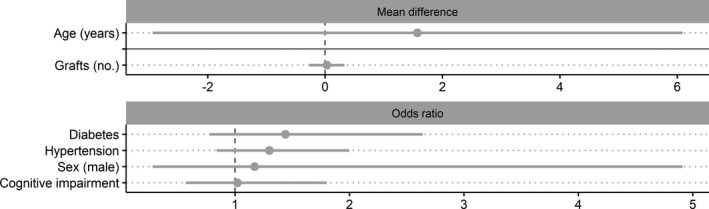
Forest plots of pooled estimates for risk or protective factors of post–coronary artery bypass grafting long‐term cognitive decline. Factors grouped according to the primary pooled estimate of the analysis (mean difference [MD], odds ratio, or standardized MD [SMD]), where solid gray horizontal lines indicate separation of preoperative, intraoperative, and postoperative factors and dashed gray vertical lines divide protective (left side) and risk (right side) factor estimates. The pooled estimates are ordered by the common calculated effect size (SMD) from largest to smallest (largest at the top). Estimates that are black represent statistically significant factors; those that are gray did not reach statistical significance. The scale for all continuous variables (MD and SMD plots) is listed within each factor name.

Data from 5 individual studies were used within 6 separate risk factor analyses for cognitive decline in the long‐term (12–15 months) post‐CABG. No analyses revealed statistically significant results, including presence of preoperative cognitive impairment, diabetes mellitus, male sex, and hypertension, nor older age or higher number of intraoperative grafts (see Table S4 for results of each meta‐analysis, Figure S7 for forest plots, and Figure 5). No meta‐regressions were performed for this time point.

## Discussion

This meta‐analysis quantifies data from >60 000 patients to identify risk and protective factors for the development of cognitive decline, including delirium, immediately following CABG and in the midterm and long‐term. Findings highlight that there are many risk factors for both delirium and cognitive decline following CABG. These factors could be integrated into existing delirium tools or shortlisted in the development of prediction tools for postoperative cognitive decline.^31^, ^32^ Further development of these clinical risk screening tools for both delirium and cognitive decline post‐CABG could lead to more accurate identification of at‐risk patients, improved prognosis, targeting of interventions, and patient management.

Risk prediction for delirium has been discussed at length for nonsurgical patients, with current models generally thought to have inadequate accuracy.^32^ Most published delirium prediction tools are based on individual clinical studies with low statistical power, decreasing their generalizability.^33^, ^34^, ^35^, ^36^ To our knowledge, no tools have been developed for predicting postoperative cognitive decline, nor have they been developed for delirium specifically following CABG. The results of this meta‐analysis can provide a shortlist of risk and protective factors that should be considered in future research for the modeling of prediction tools. Specifically, results should be considered when modifying or developing tools related to post‐CABG cognitive outcomes, as the operative process differs from other surgeries (eg, the use of CPB). Similar risk and protective factors may be applicable to other surgery types (cardiac and noncardiac); however, these factors cannot be ascertained from the current meta‐analysis. The development of CABG‐specific tools (delirium and cognitive decline) may lead to better prognosis, because of earlier identification and risk reduction strategies.

Delirium has been said to be preventable in up to 40% of cases.^9^ Recent editorials^37^, ^38^ have highlighted the importance of decreasing the incidence of delirium and cognitive decline to decrease patient and economic burden. In this meta‐analysis, modifiable risk factors, such as the presence of preoperative depression, diabetes mellitus, and hypertension, were found to increase the risk (ORs, 1.44—3.42) for both delirium and cognitive decline acutely post‐CABG. Future research should investigate the effectiveness of implementing preoperative management strategies of these factors on cognitive outcomes (delirium and cognitive decline) post‐CABG. The presence of cognitive impairment resulted in over a 4‐fold increase in risk of developing post‐CABG delirium. Cognition is known to be modifiable through cognitive training in older populations, including those presenting with heart failure,^39^, ^40^, ^41^ and therefore may be a viable preoperative target of intervention.^42^

In this meta‐analysis, preoperative depression moderately (moderate effect sizes) increased the risk of delirium (OR, 2.49), acute cognitive decline (OR, 3.42), and midterm cognitive decline (OR, 2.50) post‐CABG. In addition, a higher preoperative depression score revealed moderate to large (SMD, 0.50–0.86) increases in the risk of developing acute and midterm cognitive decline post‐CABG, yet these analyses were not statistically significant, possibly because of high heterogeneity (I^2^, 93.32–96.08; τ^2^, 0.92–1.75). Depression in late life is known to occur concurrently with cognitive impairment and can hasten the onset of dementia.^43^ The presence of vascular disease (indicative of undergoing CABG) is considered to have a strong link to the development of depression and dementia.^44^ Therefore, the effects seen across the meta‐analyses in relation to depression may not be independent from other factors. We endeavored to investigate the influence of these factors through meta‐regression, yet it was not possible because of limited studies concurrently reporting data relating to depression, cognitive impairment, and vascular disease (eg, peripheral vascular disease, hypertension, and dyslipidemia).

The presence of delirium following CABG resulted in a near 5‐fold increase (OR, 4.85, following sensitivity analysis) in risk of acute post‐CABG cognitive decline (up to 1 month). This pooled effect size was not adjusted for any preoperative or intraoperative risk factors and, therefore, its independence cannot be assumed and should be interpreted with this in mind. It may be argued that in a short‐term setting, this risk can be inflated because of the cognitive deficits of the delirium episode itself. However, the presence of delirium at this time (acute cognitive decline) is unlikely, as the assessment period for the 3 included studies was between days 7 and 9, whereas we know delirium typically resolves by day 5.^45^, ^46^, ^47^ No studies reported data related to associations between post‐CABG delirium and cognitive decline in the midterm and long‐term. Delirium in late life (not specifically surgery related) is associated with doubling the rate of cognitive decline^37^ and greatly increases the risk of incident dementia.^48^ It should therefore be a priority for surgery‐related research to investigate if post‐CABG delirium has similar impact on long‐term cognitive decline and even dementia incidence.

Only 5 studies assessed cognitive decline in the long‐term (>12 months post‐CABG), restricting risk or protective factors that could be extracted. These analyses revealed no significant results, likely because of smaller sample sizes and study variability. Cognitive decline is seen in nearly 40% of patients 1 to 5 years post‐CABG.^4^ The presence of cognitive decline is associated with decreased quality of life, functional capacity, and increased rates of depression.^7^ In addition, longer‐term cognitive decline can lead to a loss of support networks, such as friends and neighbors, and can strain familial relationships.^49^ Yet, from this meta‐analysis, because of the lack of data at this time point, no possible risk reduction strategies can be suggested.

Meta‐regressions generally found that age was not related to the pooled effect estimates. The 3 significant meta‐regressions (delirium: ACC time and LOS in ICU; acute cognitive decline: CPB time) revealed a negative relationship with age, meaning as mean age of the study sample increased, the effect of the risk factor decreased. For example, as age increased, there was a smaller difference in ACC time between those who developed delirium and those who did not. These results could be influenced by older age increasing the risk of post‐CABG complications (eg, AF, dialysis, reintubation, and stroke).^50^ These complications are likely to increase LOS in the ICU, regardless of the presence of delirium or cognitive decline. In addition, because of increased complications, greater surgical precautions may be taken with older adults (eg, prioritizing dangerously stenosed arteries over complete revascularization of coronary arteries), which may decrease overall ACC and CPB time, minimizing group differences. Although these meta‐regressions reached significance, most of the variance (≥50%) was not explained by age. Therefore, these risk factors should still be considered clinically meaningful.

This meta‐analysis revealed multiple risk factors for post‐CABG delirium and cognitive decline based on group‐level data from included studies. Future research could identify clusters of risk factors by accessing patient‐level data. This investigation could be guided by common risk factors identified in this meta‐analysis, specifically depression, cognitive impairment, stroke history, diabetes mellitus, and vascular factors (hypertension and AF).

This is the only meta‐analysis to investigate risk and protective factors for multiple outcomes (delirium and cognitive decline) across multiple time points in patients undergoing CABG. Although this study is not without limitations, the pooled sample size is >60 000 patients, allowing for greater generalizability of the results. The pooled results of this meta‐analysis cannot be directly compared across time (for cognitive decline), as the same individuals are not represented at all time points. As only studies published in English were included, there may be a geographical bias. All extracted data within this meta‐analysis were unadjusted for covariates, which does not permit investigation of independence. In addition, no temporal adjustments were conducted (eg, adjusting for preoperative depression within the intraoperative and postoperative factor meta‐analyses). Therefore, caution should be used in interpreting study results, especially on the utility of identified intraoperative and postoperative risk factors in risk prediction tools. Within the literature, substantially fewer articles investigated midterm and long‐term cognitive decline (than acute cognitive decline), which means that there may be important risk factors for these time periods that our approach could not identify. Many analyses conducted herein resulted in medium to high heterogeneity. Investigation into small study effect (publication bias) generally did not change the conclusions of this study (Figures S2, S4, and S6). The heterogeneity may be partially driven by the wide range of tests, screening tools, and methods of classifying delirium and cognitive decline within the included studies (Tables S8 and S9), although, notably, our subgroup analyses for delirium diagnosis (when using a diagnostic tool versus no tool) revealed no meaningful differences (Table S7).

## Conclusions

There are many risk factors for delirium and cognitive decline (acutely and in the midterm) following CABG, which could be used in clinical practice, including the development or modification of a clinical prediction tool. Use of a CABG‐specific risk tool could improve prognosis and, in turn, lead to better patient management. This is especially critical for delirium, as it is severely underrecognized and has serious outcomes.^9^ To improve prediction ability of these risk tools, future development could also integrate the results of functional neuroimaging (eg, electroencephalography) and biomarker research, related to CABG.

The most clinically meaningful finding from this meta‐analysis was the identification of modifiable preoperative risk factors for delirium and cognitive decline, of depression, diabetes mellitus, hypertension, and cognitive impairment. Improving the management of depression, diabetes mellitus, and hypertension in a preoperative setting may result in reductions in incident delirium and cognitive decline post‐CABG. Targeting cognitive impairment through cognitive training interventions also has potential. Even if these are small reductions in incidence rates, they will have great impact at scale. Future work should investigate if we can target modifiable risk factors to reduce the incidence of delirium and cognitive decline post‐CABG.

## Sources of Funding

D. Greaves is supported by the Australian Government Research Training Program Scholarship. Dr Keage is supported by a National Health and Medical Research Council Boosting Dementia Research Leadership Fellowship (GNT1135676) and the National Heart Foundation of Australia Vanguard Grant (101758–VG 2017). Dr Psaltis is supported by a National Heart Foundation of Australia Future Leader Fellowship (FLF100412) and a National Health and Medical Research Council Career Development Fellowship (CDF1161506). Dr Davis is supported by a Wellcome Trust Intermediate Clinical Fellowship (WT107467). Dr Lampit is supported by a National Health and Medical Research Council–Australian Research Council Dementia Research Development Fellowship (GNT1108520). Dr Smith is supported by a National Health and Medical Research Council–Australian Research Council Dementia Research Development Fellowship (GNT1097397). This project was supported by a National Heart Foundation of Australia Vanguard Grant (101758–VG 2017).

## Disclosures

None.

## Supporting information


**Data S1**
**Tables S1–S9**
**Figures S1–S7**
**References** **51**, **52**
