# Supplementary material for: Risk Factors for Delirium and Cognitive Decline Following Coronary Artery Bypass Grafting Surgery: A Systematic Review and Meta‐Analysis
Source: J Am Heart Assoc. 2020 Nov 7;9(22):e017275. doi: 10.1161/JAHA.120.017275 (PMC7763731; doi:10.1161/JAHA.120.017275)

SUPPLEMENTAL MATERIAL

Data S1.

Quality Assessment Tool

Critical Appraisal Checklist for Cohort Studies & Studies Reporting Prevalence Data

This checklist and scoring instructions, was developed for the purpose of our meta-analysis using checklists created by the Joanna Briggs Institute [21, 22].

2 points: Sufficiently fulfilled

1 point: Partially fulfilled or unclear

0 points: Unfulfilled or not reported

	2	1	0
1. Were the study participants and the setting described in detail (i.e., sample size, sex proportion, age, recruitment hospital)?			
2. Were valid and reliable methods used for the identification of the condition (i.e., cognitive decline or delirium)?*			
3. Was the condition measured in a standard, reliable way for all participants (i.e., were the assessors who administered the measures adequately trained)? If there was more than one assessor, were they similar in ability/experience?*			
4. Were appropriate procedures in place to minimise attrition?			
5. Was the follow-up time reported?			
6. Was follow up complete, and if not, were the reasons to loss to follow-up described (i.e., was there a clear and justifiable description of why participants dropped out or were excluded from the analysis)?			
Notes:			

*For studies that reported data for more than one outcome (i.e., cognitive impairment and delirium) the point scheme was relative to all outcomes. That is, a study would only score 2 on these items if all outcomes/conditions were identified using valid and reliable tests (item 2), in a valid and reliable manner (item 3).

Overall Appraisal Grade: /12

Scoring Instructions

1. **Were the study participants and the setting described in detail (i.e., sample size, sex proportion, age, recruitment hospital)?**

The study sample should be described in sufficient detail so that other researchers can determine if it is comparable to the population of interest to them. That is, did the researchers provide details on sample size, sex proportion, age and hospital recruited from for either the total sample at baseline, or the sample of participants included in the analyses?

- 2 = All details reported
- 1 = Some details reported
- 0 = No details reported

2. Were valid and reliable methods used for the identification of the condition (i.e., cognitive impairment or delirium)?

Here we are looking for measurement or classification bias. Many health problems are not easily diagnosed or defined and some measures may not be capable of including or excluding appropriate levels or stages of the health problem. If the outcomes were assessed based on existing definitions or diagnostic criteria, then the answer to this question is likely to be yes. If the outcomes were assessed using observer reported, or self-reported scales, the risk of over- or under-reporting is increased, and objectivity is compromised. Importantly, determine if the measurement tools used were validated instruments as this has a significant impact on outcome assessment validity.

Studies that reported only delirium data were assigned 2 points for this item if they used a standardized cognition or delirium assessment, and 1 point if they used a recognized criteria or guidelines (e.g., the Society of Thoracic Surgeons (STS) definition or The Diagnostic and Statistical Manual of Mental Disorders criteria.

- 2 = All measures were standardized and validated
- 1 = Some measures were standardized and validated
- 0 = No measures were standardized and validated

3. Was the condition measured in a standard, reliable way for all participants (i.e., were the assessors who administered the measures adequately trained)? If there was more than one assessor, were they similar in ability/experience?

Were those involved in collecting data trained or educated in the use of the instrument/s? If there was more than one data collector, were they similar in terms of level of education, and clinical or research experience. Overall, was the condition measured in the same way for all participants?

- 2 = The paper states that the assessor or assessors were trained. If the assessors were stated to be psychologists, neuropsychologists or psychometrists it was assumed they were adequately trained.
- 1 = Researchers mention that the tests were administered by assessors/investigators but do not mention their experience or training
- 0 = No clear statement of who conducted the assessments

4. Were appropriate procedures in place to minimize attrition?

Appropriate measures for minimizing attrition are systematic contact strategies (e.g., contacting participants three times; by letter, phone and email). A procedure would be considered inappropriate if it was not systematic (e.g., letting participants contact them, and therefore relying on their motivation).

If there was only one follow-up time that was <10 days and a strategy for minimizing attrition was not mentioned, the study was still assigned 2 points as the strategy was assumed not to be necessary (i.e., the patients were still in hospital). Also, studies that reported only delirium data were assigned 2 points using this same rationale, as the patients are assumed to still be in hospital during the delirium assessments.

- 2 = Studies that utilized a systematic contact strategy that was explicit and would be thought to lead to greater retention at follow-up, or N/A.
- 1 = Procedure mentioned (e.g., called participants) but not clear if the contact process was thorough
- 0 = No strategy mentioned

5. Was the follow up time reported?

The time points for follow-up assessments should be clearly stated. Studies with multiple follow-up assessments were assigned 2 points for this item if all follow-up time points were clearly and precisely stated, 1 point if some of the time-points were clearly and precisely stated, or all time-points were stated, but inexactly (e.g., "6 weeks").

- 2 = When the participant were likely to be out of hospital at time of assessment, studies that reported a mean and SD of the number of days/months for all follow-ups were assigned 2 points. If the follow-up time was likely to be when the patient was in hospital (e.g., 3 days) it was assumed that this was a precise value and the study was awarded 2 points.
- 1 = Studies that report a vague/inexact follow-up time (e.g., 6 months), which is likely to have varied between participants, for some or all follow-ups
- 0 = Follow-up time not reported

6. Was follow up complete, and if not, were the reasons to loss to follow up described?

Reporting of efforts to follow up participants that dropped out may be regarded as an indicator of a well conducted study. Therefore, this item is scored depending on whether a clear and justifiable description of why people were left out, excluded, dropped out, etc. was provided.

- 2 = Follow-up was complete, or if not, there was a statement of how many participants dropped out and for what reasons
- 1 = There is an unclear statement outlining reasons for drop-out and how many participants for each reason (i.e., reasons for drop-out are given but not how many participants for each reason)
- 0 = There was drop out but no mention of reasons why

Table S1. Pooled estimates and corresponding effect size (OR, MD, SMD) for pre, intra, and post-operative variables for delirium (1-7 days) post-CABG.

Variable	Pooled Estimate				Heterogeneity		Common effect size
	k (n)	OR/MD†/ SMD‡	95%CI	p value	I^2	Tau^2	SMD
**Pre-Operative (Categorical)**							
Alcoholism	6 (994)	0.90	0.50—1.62	.721	13.45	0.08	0.06
Arrhythmia, incl. AF	15 (31746)	2.07	1.70—2.51	<.001	25.35	0.03	0.40
BMI >28 (including >30)	7 (16297)	1.16	0.74—1.80	.516	56.28	0.17	0.08
BMI ≥30 only	5 (1786)	1.57	1.05—2.37	.030	0	0	0.25
Cognitive impairment	7 (1039)	4.17	2.75—6.33	<.001	0	0	0.79
Depression	4 (580)	2.49	1.29—4.81	.006	29.16	0.13	0.50
Diabetes	30 (48465)	1.49	1.39—1.60	<.001	0	0	0.22
Dyslipidemia/Hyperlipidemia	13 (6449)	0.89	0.63—1.25	.502	51.79	0.18	0.06
Education>12years/high school	4 (567)	0.66	0.41—1.06	.088	0	0	0.23
Hypertension	27 (38362)	1.44	1.21—1.70	<.001	52.54	0.07	0.20
Sex (male)	35 (37851)	0.90	0.75—1.08	.263	53.35	0.10	0.06
Kidney injury	14 (25264)	1.94	1.50—2.52	<.001	27.49	0.05	0.37
Previous MI <30 days	5 (926)	1.54	0.90—2.65	.116	37.82	0.14	0.24
Previous MI history/ever	11 (10662)	1.17	0.98—1.39	.075	0	0	0.09
Previous stroke, TIA, CVA	15 (27127)	2.55	1.94—3.35	<.001	44.42	0.10	0.52
PVD	14 (16340)	1.98	1.48—2.64	<.001	38.76	0.09	0.38
Smoking current	14 (17825)	1.19	0.84—1.69	.321	72.37	0.24	0.10
Smoking current/history	21 (25813)	1.15	0.94—1.42	.174	56.81	0.09	0.08
**Pre-Operative (Continuous)**							
Age (years) *	28 (9303)	4.14†	2.95—5.34	<.001	78.61	7.14	0.49

BMI	5 (2143)	0.03†	-0.46—0.51	.915	0	0	0.01
Cognition: All tests	9 (887)	-0.58‡	-0.78— -0.37	<.001	34.11	0.03	0.58
Cognition: MMSE only	7 (621)	-1.14†	-1.91— -0.36	.004	77.72	0.68	0.52
Depression GDS	2 (233)	0.75†	-0.15—1.65	.101	0	0	0.30
Education (years)	6 (665)	-0.93†	-1.65— -0.20	.012	19.31	0.16	0.25
EuroSCORE *	10 (11199)	1.35†	0.58—2.12	.001	96.10	1.38	0.51
LVEF (%)	11 (3308)	1.25†	-0.69—3.19	.208	79.34	7.97	0.13
**Intra-Operative (Continuous)**							
ACC time (mins)	16 (7488)	5.97†	0.62— 11.32	.029	90.65	101.19	0.29
CPB time (mins)	21 (12412)	7.41†	4.03—10.78	<.001	51.93	25.72	0.25
Duration of surgery (mins)	13 (3218)	20.53†	8.67—32.38	.001	75.96	325.89	0.35
Intubation time (hours)	11 (6693)	6.82†	2.44—11.20	.002	98.40	52.26	0.75
Number of grafts	8 (2731)	0.11†	-0.02—0.24	.084	34.30	0.01	0.13
**Post-Operative (Categorical)**							
Arrhythmia, incl. AF	16 (8809)	3.53	2.41—5.16	<.001	71.51	0.37	0.70
**Post-Operative (Continuous)**							
LOS in ICU (days)	14 (7177)	2.22†	1.32—3.13	<.001	97.84	2.69	1.20

Note: * indicates potential small-study effect or publication bias, see (**Figure S2)** and for forest plots **(Figure S1)**. Symbols following pooled estimates denote different effect sizes: indicating OR (no symbol), MD† and SMD‡. ACC= aortic cross-clamp, AF= atrial fibrillation, BMI= body mass index, CPB= cardiopulmonary bypass, CVA= cerebrovascular attack, GDS= geriatric depression scale, ICU= intensive care unit, k= number of estimates (number of studies), LOS= length of stay, LVEF= left ventricular ejection fraction, , MD= mean difference, MI= myocardial infarction, MMSE= mini mental state examination, n= pooled sample size, OR= odds ratio, PVD= peripheral vascular disease, SMD= standardized mean difference and TIA= transient ischemic attack..

Table S2. **Pooled estimates and corresponding effect size (OR, MD, SMD) for pre, intra, and post-operative variables for acute cognitive decline (immediately up to 1-month) post-CABG.**

Variable	Pooled Estimate				Heterogeneity		Effect size
	k (n)	OR/MD†/ SMD‡	95%CI	p value	I^2	Tau^2	SMD
**Pre-Operative (Categorical)**							
Arrhythmia, incl. AF	7 (945)	1.24	0.76—2.04	.389	0	0	0.12
Cognitive impairment	4 (714)	1.11	0.71—1.73	.653	0	0	0.06
Depression	2 (330)	3.42	1.12—10.46	.031	61.53	0.40	0.68
Diabetes	17 (3008)	1.44	1.21—1.72	<.001	4.18	0.01	0.20
Dyslipidemia/Hyperlipidemia	6 (836)	1.16	0.74—1.84	.512	35.86	0.11	0.08
Hypertension	15 (2012)	1.91	1.45—2.53	<.001	34.10	0.09	0.36
Sex (male)	18 (2299)	1.03	0.82—1.29	.824	0	0	0.01
Kidney injury	4 (749)	1.21	0.40—3.72	.735	54.22	0.67	0.11
Previous MI <90 days	3 (418)	1.12	0.59—2.16	.724	0	0	0.07
Previous MI history/ever	7 (1011)	1.16	0.83—1.63	.394	25.17	0.05	0.08
Previous stroke, TIA, CVA	5 (745)	2.44	1.47—4.04	.001	0	0	0.49
PVD	4 (856)	1.09	0.42—2.83	.865	57.17	0.50	0.05
Smoking current/history	9 (1400)	1.03	0.64—1.66	.892	68.09	0.31	0.02
**Pre-Operative (Continuous)**							
Age (years) *	22 (2761)	2.69†	1.20—4.18	<.001	92.85	9.17	0.53
BMI	5 (906)	-0.20†	-0.25— -0.14	<.001	0	0	0.10
Cognition: All tests	3 (151)	0.23‡	-0.43—0.88	.492	69.56	0.23	0.23
Cognition: MMSE only	2 (116)	0.28†	-1.39—1.96	.740	82.66	1.21	0.24
Depression: All tests	4 (448)	0.50‡	-0.48—1.48	.316	93.32	0.92	0.50

Education (years)	6 (534)	-0.44†	-1.40—0.53	.377	49.52	0.65	0.11
EuroSCORE	4 (582)	0.74†	0.48—1.01	<.001	0	0	0.46
LVEF %	9 (1126)	-1.97†	-4.21—0.28	.086	72.49	8.28	0.21
**Intra-Operative (Continuous)**							
ACC time (mins)	7 (867)	-1.34†	-5.91—3.23	.566	61.81	20.52	0.07
CPB time (mins)	13 (1699)	3.39†	-0.10—6.88	.057	59.34	16.87	0.15
Duration of surgery (mins)	6 (723)	13.52†	3.67—23.38	.007	21.27	32.28	0.26
Intubation time (hours)	6 (1193)	1.95†	1.37—2.52	<.001	28.29	0.15	0.82
Number of grafts	7 (1113)	0.03†	-0.03—0.09	.400	7.50	0	0.10
Total microemboli	4 (771)	0.16‡	-0.07—0.38	.167	45.77	0.02	0.16
**Post-Operative (Categorical)**							
Arrhythmia, incl. AF	6 (1045)	1.40	1.01—1.94	.042	0	0	0.19
Delirium	3 (355)	6.15	2.32—16.27	<.001	6.32	0.07	1.00
Without outlier	2 (308)	4.85	1.89—12.45	.001	0	0	0.87
**Post-Operative (Continuous)**							
LOS in ICU (days)	7 (1055)	0.29†	0.04—0.55	.025	77.82	0.08	0.77

Note: * indicates potential small-study effect or publication bias, see (**Figure S4)** and for forest plots **(Figure S3)**. Symbols following pooled estimates denote different effect sizes: indicating OR (no symbol), MD† and SMD‡. ACC= aortic cross-clamp, AF= atrial fibrillation, BMI= body mass index, CPB= cardiopulmonary bypass, CVA= cerebrovascular attack, ICU= intensive care unit, k= number of estimates (number of studies), LOS= length of stay, LVEF= left ventricular ejection fraction, , MD= mean difference, MI= myocardial infarction, n= pooled sample size, OR= odds ratio, PVD= peripheral vascular disease, SMD= standardized mean difference and TIA= transient ischemic attack

Table S3. **Pooled estimates and corresponding effect size (OR, MD, SMD) for pre, intra, and post-operative variables for cognitive decline in the mid-term (1 to 6-months) post-CABG.**

Variable	Pooled Estimate				Heterogeneity		Effect size
	k (n)	OR/MD†/ SMD‡	95%CI	p value	I^2	Tau^2	SMD
**Pre-Operative (Categorical)**							
Depression	2 (471)	2.50	1.28—4.88	.007	0	0	0.51
Diabetes	10 (2046)	1.54	0.98—2.40	.059	61.33	0.28	0.24
Sex (male)	12 (2599)	1.01	0.82—1.23	.965	0	0	0.00
Hypertension	12 (2350)	0.89	0.59—1.32	.558	69.32	0.31	0.07
Previous MI history/ever	3 (975)	0.88	0.57—1.37	.580	41.32	0.06	0.07
Previous stroke, TIA, CVA	2 (761)	0.83	0.26—2.63	.748	0	0	0.11
PVD	2 (761)	0.71	0.41—1.21	.209	0	0	0.19
Smoking (current)	5 (1006)	1.00	0.73—1.37	.983	0	0	0.00
**Pre-Operative (Continuous)**							
Age (years)	12 (2093)	1.09†	-0.06—2.25	.063	29.21	1.12	0.13
Cognition: All tests	3 (855)	0.30‡	0.01—0.59	.041	68.84	0.04	0.30
Cognition: CI only	2 (795)	0.50†	-0.17—1.17	.146	89.28	0.21	0.43
Depression: All tests	3 (429)	0.86‡	-0.68—2.39	.273	96.08	1.75	0.86
Education (years)	5 (950)	0.13†	-0.57—0.83	.715	44.26	0.24	0.05
LVEF %	7 (1266)	0.61†	-0.63—1.86	.336	0	0	0.06
**Intra-Operative (Continuous)**							
ACC time (mins)	4 (890)	-1.29†	-4.35—1.76	.407	0	0	0.06
CPB time (mins)	7 (1266)	2.15†	-2.44—6.74	.359	28.63	10.41	0.06
Number of grafts	4 (1124)	-0.06†	-0.17—0.06	.358	0	0	0.06

Total microemboli	4 (542)	-0.46‡	-0.97—0.05	.076	51.03	0.12	0.46
**Post-Operative (Continuous)**							
LOS in ICU (days)	2 (100)	0.23†	-1.10—1.55	.736	88.27	0.80	0.33

Note: * indicates potential small-study effect or publication bias, see (**Figure S6)** and for forest plots **(Figure S5)**. Symbols following pooled estimates denote different effect sizes: indicating OR (no symbol), MD† and SMD‡. ACC= aortic cross-clamp, CI= cognitive index score, CPB= cardiopulmonary bypass, CVA= cerebrovascular attack, ICU= intensive care unit, k= number of estimates (number of studies), LOS= length of stay, LVEF= left ventricular ejection fraction, , MD= mean difference, MI= myocardial infarction, n= pooled sample size, OR= odds ratio, PVD= peripheral vascular disease, SMD= standardized mean difference and TIA= transient ischemic attack.

Table S4. Pooled estimates and corresponding effect size (OR, MD, SMD) for pre, intra, and post-operative variables for cognitive decline in the long-term (12 to 15-months) post-CABG.

Variable	Pooled Estimate				Heterogeneity		Effect size
	k (n)	OR/MD†/ SMD‡	95%CI	p value	I^2	Tau^2	SMD
**Pre-Operative (Categorical)**							
Cognitive impairment	2 (343)	1.02	0.57—1.80	.952	0	0	0.01
Diabetes	2 (504)	1.44	0.78—2.64	.245	0	0	0.20
Sex (male)	2 (301)	1.17	0.28—4.91	.830	28.63	0.49	0.09
Hypertension	2 (504)	1.30	0.84—2.00	.241	0	0	0.14
**Pre-Operative (Continuous)**							
Age (years)	2 (301)	1.57†	-2.94—6.09	.495	46.03	5.30	0.17
**Intra-Operative (Continuous)**							
Number of grafts	2 (301)	0.03†	-0.27—0.33	.832	0	0	0.04

Note: * indicates potential small-study effect or publication bias. See **(Figure S7)** for forest plots. Symbols following pooled estimates denote different effect sizes: indicating OR (no symbol), MD† and SMD‡. k= number of estimates (number of studies), MD= mean difference, n= pooled sample size, OR= odds ratio, SMD= standardized mean difference.

Table S5. Excluded references from full-text screening with associated reason.

Reference	Exclusion Reason
Abner EL, Ding X, Caban-Holt AM, Schmitt FA, Kryscio RJ. Comorbid subjective cognitive decline and sleep apnea significantly increase the risk of incident dementia: Results from the prevention of alzheimer's disease with vitamin e and selenium study. Alzheimer's and Dementia. 2015;11:P733	abstract only
Aghadavoudi Jolfaei O, Bagheri K, Motamedi O, Akbari M. The effect of mean arterial pressure during cardiopulmonary bypass on clinical and para clinical parameters during and after coronary artery bypass graft surgery. European Journal of Anaesthesiology. 2012;29:69-70	abstract only
Al Tmimi L, Sergeant P, Velde M, Meyns B, Coburn M, Rex S. Xenon anaesthesia in patients undergoing off-pump coronary artery bypass graft surgery: A prospective, randomized controlled clinical trial (eudract 2012-002316-12). European Journal of Anaesthesiology. 2014;31:71	abstract only
Alexander W. American college of cardiology meeting highlights. P and T. 2017;42:340-343	abstract only
Andrejaitiene J, Sirvinskas E, Svagzdiene M. Post-cardiac surgery delirium risk factors and clinical outcome. Applied Cardiopulmonary Pathophysiology. 2012;16:251-252	abstract only
Annett Salzwedel A, Heidler MD, Wegscheider K, Schikora M, Jobges M, Zaskia P, Voller H. Cognitive performance in cardiac rehabilitation: Impact on short- and medium-term outcome of patient education. European Journal of Preventive Cardiology. 2017;24 (1 Supplement 1):S10	abstract only
Anonymous. Alzheimer's gene linked with postoperative confusion. Journal of psychosocial nursing and mental health services. 1998;36:13-14	abstract only
Antonitsis P, Anastasiadis K, Argiriadou H, Kosmidis MH, Megari K, Thomaidou E, Aretouli E, Papakonstantinou C. Improved neurocognitive outcome after coronary artery bypass surgery using minimal versus conventional extracorporeal circulation: A randomized controlled study. Journal of the American College of Cardiology. 2011;57:E910	abstract only
Arenson B, Grocott H, MacDonald L, Hiebert BM, Carino M, Freed DH, Arora RC. The effectiveness of implementing a systematic screening tool to improve identification of delirium after cardiac surgery. Canadian Journal of Cardiology. 2012;28:S288	abstract only
Arsenova N, Mosenko S. Neurological complications at the coronary artery bypass grafting. European journal of neurology. 2010;17:169	abstract only
Arthur B, Tan S, Alston P. Is postoperative delirium associated with the use of cardio-pulmonary bypass? A comparison of patients undergoing on- and off-pump coronary artery bypass grafting surgery. Journal of cardiothoracic and vascular anesthesia. 2011;25:S63-S64	abstract only
Arunagirinathan U, Gruning A, Gulbins H, Reichenspurner H. Cognitive outcomes and quality of life after on-pump versus off-pump coronary artery bypass grafting. Thoracic and Cardiovascular Surgeon. 2011;59:no pagination	abstract only
Awe M, Dalyanoglu H, Schipke JD, Lichtenberg A, Korbmacher B. Postoperative delirium: Examination of 1n206 cardio-surgical patients of a single university center during one-year period. Thoracic and Cardiovascular Surgeon. Conference: 46th Annual Meeting of the German Society for Thoracic and Cardiovascular Surgery, DGTHG. 2017;65	abstract only
Baroncelli F, Barile L, D'Agrosa L, Jain A, Lombrano MR, Marini E, Forfori F, Martinelli G, Meraglia A. Adherence to the local guidelines for the management of delirium in a cardiothoracic intensive care unit: A clinical audit. Applied Cardiopulmonary Pathophysiology. 2014;18:102	abstract only
Bendov DV, Gordeev ML. Simultaneous carotid endarterectomy and coronary artery bypass grafting. Interactive cardiovascular and thoracic surgery. 2011;12:S77-S78	abstract only
Brown CH. The effect of remote ischemic preconditioning on delirium after cardiac surgery. Anesthesia and analgesia. 2016;122:S64	abstract only
Caldas J, Panerai R, Camara L, Ferreira G, Bor-Seng-Shu E, Lima M, Galas F, Mian N, Nogueira R, Queiroz De Oliveira G, et al. Dynamic cerebral autoregulation: A marker of post-operative delirium? Critical Care. Conference: 37th International Symposium on Intensive Care and Emergency Medicine. Belgium. 2017;21	abstract only

Caldas J, Panerai R, Ferreira G, Camara L, Zeferino S, Jardim J, Bor-Seng-Shu E, Oliveira M, Norgueira R, Groehs R, et al. Cerebral hemodynamic in high-risk cardiac patients undergoing cardiac surgery with cardiopulmonary bypass: The role of intraaortic balloon. Critical Care. Conference: 37th International Symposium on Intensive Care and Emergency Medicine. Belgium. 2017;21	abstract only
Chugunova J, Chumakova G, Gritzenko O, Veselovskaya N. Neuropsychological dysfuntions among patients with artery bypass grafting. Atherosclerosis. 2015;241:e212	abstract only
Ding S, Feng X, Tao X, Lu S, Su P, Shen Y, Zhao H, Wu Y. Including intra-and post-operative factors in delirium predictive rule after coronary artery bypass graft could increase its accuracy. Circulation. 2015;132:no pagination	abstract only
Dorynska A, Kozela M, Pajak A. Verbal memory and verbal fluency as predictors of cardiovascular disease incidence in polish cohort of the hapiee study. European Journal of Preventive Cardiology. 2018;25 (2 Supplement 1):S31-S32	abstract only
Duara R, Barker WW, Potter E, Appel J, Bhatia N, Greig M, Wu Y, Schofield E, Loewenstein D. Long-term effect of history of coronary artery bypass grafting surgery (cabg) on cognitive diagnosis, mri findings and episodic memory scores. Alzheimer's and Dementia. 2009;5:387-388	abstract only
Dzemali O, Graves K, Loeblein H, Zientara A, Kostorz A, Haussler A, Genoni M. Risk factors and incidence for postop delirium in patients undergoing cardiac surgery with cardiopulmonary bypass support vs. Off pump surgery. Thoracic and Cardiovascular Surgeon. 2013;61:no pagination	abstract only
Ellouze M, Badrudin D, Cartier R. Statins decrease incidence of delirium after opcab surgery. Canadian Journal of Cardiology. 2015;31:S173	abstract only
Eshankulova N, Mirataliyevna N. Aortic atherosclerosis and postoperative neurological dysfunction in elderly coronary surgical patients. Cerebrovascular Diseases. 2017;43 (Supplement 1):41	abstract only
Evered L, Scott D, Silbert B, Ames D. A prospective evaluation of dementia following coronary artery bypass graft surgery. European Journal of Anaesthesiology. 2014;31:121	abstract only
Evered L, Silbert B, Ames D, Maruff P, Scott D. Does general anaesthesia exacerbate alzheimer's disease? Alzheimer's and Dementia. 2012;8:P208-P209	abstract only
Evered L, Silbert B, Scott D, Maruff P, Li QX, Masters CL. Plasma abeta42 levels predict cognitive dysfunction after cardiac surgery. Anaesthesia and intensive care. 2009;37:655	abstract only
Evered L, Silbert B, Scott D, Maruff P. Cardiac surgery accelerates decline in clinical dementia rating scale. Alzheimer's and Dementia. 2011;7:S243	abstract only
Feola M, Menditto E, Nervo E, Lombardo E, Taglieri C, Piccolo S, Peano M, Vallauri P. Efficacy of the cardiovascular rehabilitation in patients with congestive heart failure or after cardiac surgery. European Journal of Heart Failure, Supplement. 2010;9:S270	abstract only
Feola M, Vallauri P, Taglieri C, Lombardo E, Nasi M, Aspromonte N, Valle R. Is there a correlation between cognitive impairment and prognostic parameters in patients with congestive heart failure or after cardiac surgery? European Journal of Heart Failure, Supplement. 2009;8:ii604	abstract only
Freibrodt J, Huppe M, Sedemund-Adib B, Sievers HH, Schmidtke C. Can we predict the occurence of postoperative delirium after cardiac surgery in the elderly by a clinical and frailty assessment? Thoracic and Cardiovascular Surgeon. 2013;61:no pagination	abstract only
Freibrodt J, Huppe M, Sedemund-Adib B, Sievers HH, Schmidtke C. Effect of postoperative delirium on quality of life and daily activities 6 month after elective cardiac surgery in the elderly. Thoracic and Cardiovascular Surgeon. 2013;61:no pagination	abstract only
Galina Chumakova G, Chugunova J, Veselovskaya N. Dynamics of cognitive functions in patient with visceral obesity undergoing coronary artery bypass grafting. European Journal of Preventive Cardiology. 2016;23:S56	abstract only
Gasparovic H, Kopjar T, Anticevic A, Rados M, Malojcic B, Ivancan V, Fabijanic T, Cikes M, Milicic D, Gasparovic V, et al. Remote ischemic preconditioning preceding coronary artery bypass grafting reduces the volume of ischemic brain lesions. Circulation. Conference: Resuscitation Science Symposium, ReSS. 2017;136	abstract only

Gauge N, Salaunkey K, Zhu J, Ferreira N, Aron J, Araujo H, Green D, Amoako D, Ballard C, Kunst G. Optimization of intra-operative depth of anaesthesia and cerebral oxygenation significantly reduces postoperative delirium after coronary artery bypass graft surgery. Applied Cardiopulmonary Pathophysiology. 2014;18:68	abstract only
Girgin S, Aksun M, Karahan N, Golboyu B, Sencan A, Yurekli I, Gunes T, Aran G, Gurbuz A. Effects of intraoperative monitoring of cerebral oximetry on postoperative neurocognitive functions of patients after cabg surgery. Cochrane Central Register of Controlled Trials (CENTRAL). 2012;16:268-269	abstract only
Giubbolini G, Nitti M, Di Volo S, Costoloni G, Durante F, Casolaro I, Morbidelli G, Zanobini F, Biagioli B, Fagiolini A. Psychiatric disorders in intensive care unit post cardio-surgery. European Neuropsychopharmacology. 2014;24:S357-S358	abstract only
Gottesman RF, Grega MA, Bailey MM, Pham LD, Zeger SL, Baumgartner WA, Selnes OA, McKhann GM. Delirium after cabg surgery is associated with higher long-term mortality. Stroke. 2009;40:e261	abstract only
Gottesman RF, Grega MA, James N, Baumgartner WA, Selnes OA, McKhann GM. Impact of cognition on mortality in individuals with coronary artery disease. Annals of neurology. 2010;68:S12	abstract only
Gottesman RF, Grega MA, Selnes OA, Thompson RE, Zhou X, James N, Baumgartner WA, McKhann GM. Stroke risk predicts cognitive performance over 6 years in patients with coronary artery disease. Stroke. 2011;42:e55	abstract only
Graham AA. The association between baseline frailty and delirium or functional status after cardiac surgery. Journal of the American Geriatrics Society. 2016;64:S142-S143	abstract only
Herrmann N, O'Regan J, Schwartz YB, Saleem M, Swardfager W, Lanctot K. Cognitive effects of anticholinergic medications in patients with coronary artery disease. American Journal of Geriatric Psychiatry. 2013;21:S159	abstract only
Holmgaard F, Vedel AG, Rasmussen LS, Langkilde A, Nilsson JC, Ravn HB. Near infrared spectroscopy at two levels of mean arterial pressure during cardiopulmonary bypass. Journal of Cardiothoracic and Vascular Anesthesia. 2017;31 (Supplement 1):S3	abstract only
Hosang S, Bartels C, Hansen M, Herr M, Schilling T, Baraki H, Kutschka I, Hadem J. Short-term cognitive function following coronary artery bypass grafting in patients at high risk of cerebrovascular events - preliminary results from a prospective study. Internist. 2017;58 (Supplement 1):S36	abstract only
Hsieh SJ, Fuster D, D'Alessandro DA, Leff JD, Gong MN. Feasibility and efficacy of intranasal insulin for post-operative delirium: The cns-elders randomized controlled trial. American Journal of Respiratory and Critical Care Medicine. 2015;191:no pagination	abstract only
Ishida K, Yamashita A, Furutani A, Oshibuchi R, Yamagata H, Sakamoto S, Fukuda S, Matsumoto M. Association between change in intraoperative regional cerebral oxygen saturation and development of postoperative cognitive dysfunction after off-pump coronary artery bypass graft surgery. Journal of neurosurgical anesthesiology. 2012;24:450-451	abstract only
Ishida K, Yamashita A, Uchida M, Nakanishi T, Yamashita S, Utada K, Iida Y, Kaneko S, Shiramoto K, Matsumoto M. Evaluation of factors associated with development of postoperative cognitive dysfunction after off-pump coronary artery bypass graft surgery. Journal of neurosurgical anesthesiology. 2015;27:456-457	abstract only
Johansson E, Wohlin S, Andersson EA, Thulesius H. Cognitive function and postoperative delirium in 64 elective cardiac surgery patients. Scandinavian Cardiovascular Journal. 2012;46:42	abstract only
Kacar MB, Kacar SM, Doric VV, Majdevac SM, Zagoricnik MR, Cemerlic-Adic NL. Postoperative cognitive dysfunction after cardiopulmonary bypass demonstrated by psychometric testing - is it really common complication? European Journal of Anaesthesiology. 2011;28:8	abstract only
Kadoi Y. Effects of balloon-induced pulsatile perfusion on postoperative short- and long-term cognitive dysfunction in diabetic patients with impaired cerebrovascular carbon dioxide reactivity. Critical care medicine. 2011;39:58	abstract only
Kastaun S, Gerriets T, Schwarz N, Tschernatsch M, Kaps M, Kraus J, Schoenburg M, Walther T. Charles bonnet syndrome-like pseudo-hallucinations following heart surgery in patients with normal vision. Journal of Neurology. 2011;258:S46	abstract only

Kastaun S, Schwarz N, Schonburg M, Mollmann H, Bachmann G, Sammer G, Hamm C, Walther T, Gerriets T. Post-interventional cognitive dysfunction and ischemic brain lesions after cardiac catheter procedures and cabg. Thoracic and Cardiovascular Surgeon. 2012;60:no pagination	abstract only
Kazmierski J, Banys A, Latek J, Bourke J, Jaszewski R, Kloszewska I, Barbosa A, Cosci F. Raised il-2 and tnf-alpha concentrations are independently associated with delirium after coronary artery bypass graft surgery. Psychotherapy and psychosomatics. 2013;82:47	abstract only
Kazmierski J, Banys A, Latek J, Bourke J, Jaszewski R, Kloszewska I. Cortisol levels and neuropsychiatric diagnosis as markers of postoperative delirium. European Psychiatry. 2013;28:no pagination	abstract only
Keunen RW, Palmbergen WA, Van Sonderen A, Falsafi MA, Duynstee F, Claessen K. Consistent reduction of post coronary artery bypass grafting delirium frequency by the haga braincare strategy. Cerebrovascular Diseases. 2013;35:748	abstract only
Keunen RWM, Palmbergen W, Van Sonderen A, Falsaf PA, Bartels E, Poppert H, Schlachetzki F, Russell D, Csiba L. Improved peri-operative neurological screening reduces postoperative delirium after cabg surgery. Cerebrovascular Diseases. 2011;31:24	abstract only
Kishmaraia T, Rukhadze I, Katsitadze Z, Kaloiani V, Chanishvili T. Heart and brain cerebral disorder and changes of polysomnographic (psg) parameters after cardiac surgery. International Journal of Stroke. 2014;9:198-199	abstract only
Knipp SC, Flesch M, Wilhelm H, Massoudy P, Schlamann M, Assenmacher E, Philipp T, Erdmann E, Diener HC, Jakob H. Effect of candesartan on cognitive and renal function in patients undergoing on-pump coronary artery bypass grafting: The arta trial. Stroke. 2009;40:e273	abstract only
Krysta K, Woznica A, Krzych L, Pawlak A, Skarysz J, Eysmontt Z, Krupka-Matuszczyk I, Bochenek A, Cisowski M, Janas-Kozik M. Analysis of correlation between intensity of anxiety, depression and cognitive functions - before and after coronary artery bypass surgery. European Psychiatry. 2010;25:no pagination	abstract only
Krysta K, Woznica A, Krzych LJ, Pawlak A, Skarysz J, Eysmontt Z, Krupka-Matuszczyk I, Bochenek A, Cisowski M. Cognitive functioning in young, professionally active men after cabg operation. European Psychiatry. 2011;26:no pagination	abstract only
Krzych LJ, Woznica A, Pawlak A, Skarysz J, Eysymontt Z, Krupka-Matuszczyk I, Bochenek A, Cisowski M. Quality of life in young and professionally active men undergoing on-pump coronary artery bypass grafting. European Neuropsychopharmacology. 2009;19:S336	abstract only
Kumpaitiene B, Svagzdiene M, Sirvinskas E, Zakelis R, Petkus V, Chomskis R, Ragauskas A, Benetis R. Disorder of cerebrovascular autoregulation during cardiac surgery with cpb and its relation with the rate of post-operative cognitive dysfunction. Journal of cardiothoracic and vascular anesthesia. 2016;30:S5-S6	abstract only
Kuzma E, Airdrie J, Littlejohns TJ, Lourida I, Thompson-Coon J, Lang IA, Scrobotovici M, Thacker E, Fitzpatrick AL, Kuller LH, et al. Coronaryartery bypass graft surgery and dementia risk in the cardiovascular health study. Alzheimer's and Dementia. 2016;12:P248-P249	abstract only
La Pier T. The predictive value of the timed-up-and-go on disability in older patients 1-year after coronary artery bypass surgery. Physiotherapy (United Kingdom). 2011;97:eS663	abstract only
Langebartels G, Strauch J, Naraghi H, Liakopoulos O, Choi YH, Wahlers T. Only cabg procedures on pump lead to significant early postoperative increase in neurone specific enolase (nse) levels leading to deficits in neurocognitive abilities. Thoracic and Cardiovascular Surgeon. 2010;58:no pagination	abstract only
Lazibat I, Sutlic Z, Brkic K, Rudez I, Baric D, Unic D, Jonjic D. Predictors of the short-term neurocognitive outcome following coronary revascularization depending on the use of cardiopulmonary bypass. Interactive cardiovascular and thoracic surgery. 2011;12:S111	abstract only
Li N, Wang DX, Mu DL, Shan GJ. Impact of early postoperative delirium on long-term survival rate, cognitive function, and quality of life in patients after coronary artery bypass graft surgery. British Journal of Anaesthesia. 2012;108:549p-550p	abstract only
Likhvantsev V, Grebenchikov O, Shaibakova V, Levikov D, Cherpakov R. Neuroprotective effect of volatile induction and maintenance of the anesthesia (vima) vs. Total intravenous anesthesia (tiva) during on-pump coronary artery bypass grafting (cabg). European Journal of Anaesthesiology. 2013;30:54	abstract only
Lin IH, Oldham M, Hawkins K, Scoutt L, Yuh D, Lee H. Depression predicts delirium after coronary artery bypass graft surgery independent of cognitive impairment and cerebrovascular disease: An analysis of the noahs study. American Journal of Geriatric Psychiatry. 2019;27 (3 Supplement):S160-S161	abstract only
Loblein H, Dzemali O, Haab A, Haussler A, Graves K, Schurr U, Odavic D, Seiffert B, Genoni M. The impact of preoperative anaemia on patients undergoing coronary bypass surgery. Thoracic and Cardiovascular Surgeon. 2011;59:no pagination	abstract only

Losenno K, Peterson M, Ouzounian M, Whitlock R, Dagenais F, Boodhwani M, Bhatnagar G, Poostizadeh A, Pozeg Z, Moon M, et al. Early clinical outcomes of a novel frozen elephant trunk prosthesis: The canadian thoracic aortic collaborative experience. Canadian Journal of Cardiology. 2017;33 (10 Supplement 1):S86	abstract only
Mankute A, Usas E, Sirvinskas E, Andrejaitiene J. Assessment of short-term cognitive function in patients undergoing coronary artery bypass graft surgery with or without intraoperative external head cooling technique: Pilot study. European Journal of Anaesthesiology. 2012;29:1	abstract only
Marchioli R, Mozaffarian D, Silletta G, Macchia A, Ferrazzi P, Gardner T, Latini R, Libby P, Lombardi F, O'Gara P, et al. Fish oil for the prevention of post-operative atrial fibrillation - the omega-3 fatty acids for prevention of post-operative atrial fibrillation (opera) trial. Circulation. 2012;126:2781	abstract only
Martinovic I, Lindemann S, Irqsusi M, Mirat J, Vcev A, Wittlinger T, Noutsias M. Promising 12-months clinical results for anaortic multivessel all-arterial minimally invasive direct coronary bypass surgery via distal mini-sternotomy. European Journal of Heart Failure. 2018;20 (Supplement 1):486	abstract only
Menditto E, Nervo E, Lombardo E, Taglieri C, Piccolo S, Vallauri P, De Blasi M, Feola M. The correlation between cognitive impairment and prognostic parameters in patients with congestive heart failure or after cardiac surgery. European Journal of Cardiovascular Prevention and Rehabilitation. 2010;17:S107	abstract only
Merino JG, Latour LL, Tso AR, Lee KY, Kang DW, Davis LA, Warach S. Blood-brain barrier disruption after cardiac surgery. Stroke. 2011;42:e90	abstract only
Mickle A, Budelier T, Burton J, Oberhaus J, Park D, McKinnon S, Avidan M. Effect of intubation duration on postoperative delirium after cardiac surgery. Anesthesia and Analgesia. 2018;126 (4 Supplement 1):283	abstract only
Mishchenko TS, Kharina KV, Dmytriieva OV, Derevetska VG. Dynamic changes of cognitive conditions in patients after different types of cardiac surgery. European Stroke Journal. 2018;3 (1 Supplement 1):476-477	abstract only
Miyazaki S, Yoshitani K, Miura T, Ohnishi Y. Predictors of postoperative derilium and stroke after off-pump coronary artery bypass surgery. Journal of neurosurgical anesthesiology. 2008;20:330	abstract only
Mufti HN, Abidi S, Abidi SR, Hirsch GM. Predictors of post-operative delirium in cardiac surgery patients; a machine learning approach. Canadian Journal of Cardiology. 2014;30:S237-S238	abstract only
Muller Moran H, Maguire D, Kowalski S, Jacobsohn E, Mackenzie G, Grocott H, Arora R. Effect of earlier extubation on postoperative delirium after coronary artery bypass grafting. Canadian Journal of Cardiology. 2017;33 (10 Supplement 1):S92	abstract only
Nervo E, Menditto E, Lombardo E, Taglieri C, Piccolo S, Vallauri P, Peano M, Feola M. Efficacy of the cardiovascular rehabilitation in patients with congestive heart failure or after cardiac surgery. European Journal of Cardiovascular Prevention and Rehabilitation. 2010;17:S21	abstract only
Niemann B, Salzmann M, Giesler T, Rohrbach S, Mirow N, Vogt S, Grieshaber P, Roth P, Boning A. New onset postoperative atrial fibrillation: Relevance of peri- and intraoperative characteristics for incidence of atrial fibrillation and patient outcome? Thoracic and Cardiovascular Surgeon. Conference: 47th Annual Meeting of the German Society for Thoracic and Cardiovascular Surgery, DGTHG. 2018;66	abstract only
Niemeyer-Guimaraes M, Cendoroglo MS, Mello-Almada C. Functional capacity of older adults undergoing coronary artery bypass graft surgery: A 6 months follow-up. Journal of General Internal Medicine. 2013;28:S80	abstract only
Norkiene I, Ivaskevicius J. Impact of preoperative anxiety and depression on postoperative cognitive decline and outcomes after coronary artery bypass grafting. Interactive cardiovascular and thoracic surgery. 2010;10:S201	abstract only
Norkiene I, Samalavicius R, Misiuriene I, Ivaskevicius J, Seeberger M, Jakobsen CJ, Manners J. Asymptomatic carotid artery stenosis and postoperative cognitive outcome in patients undergoing on-pump coronary artery bypass grafting. Journal of cardiothoracic and vascular anesthesia. 2010;24:S34	abstract only
Norkiene I, Samalavieius R, Ivaeius J. Asymptomatic carotid artery stenosis and cognitive outcomes after coronary artery bypass grafting. Journal of vascular surgery. 2012;55:614	abstract only
Oldham M, Lee HB. Mild cognitive impairment predicts post-operative delirium after coronary artery bypass graft surgery: Preliminary data from the neuropsychiatric outcomes after heart surgery study. American Journal of Geriatric Psychiatry. 2015;23:S71-S72	abstract only

O'Neal J, Dighe S, Gray K, Lerner A, Talmor D, Marcantonio E, Subramaniam B. The use of dexmedetomidine and intravenous acetaminophen for the prevention of postoperative delirium in cardiac surgery patients over 60 years of age: A pilot study. Anesthesia and analgesia. 2015;120:S411	abstract only
O'Neal JB, Liu X, Shotwell MS, Liang Y, Shaw A, Shah A, Billings FT. Exposure to cardiopulmonary bypass during coronary artery bypass surgery and postoperative delirium. Anesthesia and analgesia. 2016;122:S56	abstract only
O'Neal JB, Liu X, Shotwell MS, Liang Y, Shaw A, Shah A, Billings FT. Impact of preoperative statin or beta blocker use and delirium following cardiac surgery. Anesthesia and analgesia. 2016;122:S57	abstract only
O'Neill B, Taylor F, Vohra A. Health, survival and quality of life after coronary artery bypass grafting: A ten year follow-up study. Journal of cardiothoracic and vascular anesthesia. 2011;25:S1	abstract only
O'Neill B, Taylor F, Vohra A. Quality of life is improved after coronary artery bypass grafting-a 10-year follow-up study. Interactive cardiovascular and thoracic surgery. 2011;12:S18	abstract only
Ovchinnikov D, Garnyuk V, Vorobiev E, Beltiukov P, Grebennik V, Gordeev M, Barantsevich E. Postoperative cognitive decline after cabg-association between cytokines profile and matrix metalloproteinases 2 and 9. Journal of the Neurological Sciences. 2017;381 (Supplement 1):770-771	abstract only
Pacini D, Di Marco L, Marsilli D, Pettinato C, Tonon C, Fonti C, Lodi R, Di Bartolomeo R. Cerebral function and metabolism after antegrade selective cerebral perfusion in aortic arch surgery. Interactive cardiovascular and thoracic surgery. 2009;9:S91	abstract only
Palotas A, Reis H, Teixeira A, Mukhamedyarov M, Rizvanov A, Janka Z, Kalman J. Introducing post-surgical alzheimer's disease. European journal of neurology. 2010;17:74	abstract only
Parhar H, Reimer-Kent J, Merchant RN. A rapid recovery pathway for cabg surgery: One institutional experience. Canadian Journal of Anesthesia. 2013;60:S97	abstract only
Patel N, Janus J, Banahan C, Horsfield MA, Evans DH, Chung E. Acute neuroimaging cerebral ischemic lesions on fluid-attenuated inversion recovery imaging are not associated with neuropsychological decline after cardiac surgery. International Journal of Stroke. 2014;9:53	abstract only
Patel N, Janus J, Banahan C, Spyt T, Evans DH, Chung EML, Azevedo E, Oliveira V, Sargento-Freitas J, Russell D, et al. The number and timing of cerebral emboli during cardiac surgery in conjunction with post-operative mri scans and neuropsychological tests. Cerebrovascular Diseases. 2013;35:70	abstract only
Pereira M, Jung P, Tangri N, Hiebert B, Freed DH, Arora RC. The impact of frailty on post-operative delirium in patients undergoing cardiac surgery. Canadian Journal of Cardiology. 2013;29:S206	abstract only
Perez-Belmonte LM, San Roman-Teran CM, Jimenez-Navarro M, Barbancho MA, Garcia-Alberca JM, Lara JP. Assessment of long-term cognitive impairment after off-pump coronary-artery bypass grafting and related risk factors. Journal of the American Medical Directors Association. 2015;16:263.e269-211	abstract only
Perthel M, Daum I. Comparison of the neurocognitive sequelae of coronary artery bypass grafting (cabg) using conventional (cecc) or minimized extracorporeal circulation (minecc). Canadian Journal of Cardiology. 2011;27:S96-S97	abstract only
Petkus V, Kumpaitiene B, Svagzdiene M, Sirvinskas E, Zaklelis R, Krakauskaite S, Chomskis R, Benetis R, Ragauskas A. System of real-time non-invasive diagnosis of cerebrovascular autoregulation impairments during cardiac surgery with cardiopulmonary bypass and assessment of postoperative deterioration of cognitive functions. Cerebrovascular Diseases. 2017;43 (Supplement 1):124	abstract only
Petkus V, Zakelis R, Kumpaitiene B, Svagzdiene M, Sirvinskas E, Adomaitiene V, Krakauskaite S, Chomskis R, Ragauskas A, Benetis R. Cerebrovascular autoregulation impairments during cardiac surgery with cardiopulmonary bypass are related to postoperative cognitive deterioration. European Journal of Neurology. 2018;25 (Supplement 1):49-50	abstract only
Pirat A, Komurcu O, Camkiran A, Arslan G, Bayraktar N, Sezgin A. A comparison of hemodynamic effects of etomidatemidazolam and ketamine-midazolam for anesthesia induction in coronary artery bypass grafting surgery. Intensive care medicine. 2013;39:S414	abstract only
Ponea AM, Hsieh SJ, Fuster D, Gong MN. Safety and tolerability of intranasal insulin in older critically ill patients. American Journal of Respiratory and Critical Care Medicine. 2015;191:no pagination	abstract only

Pugsley W, Klinger L, Newman S, Harrison M, Paschalis C. The role of microemboli in the neuropsychological sequelae of coronary artery bypass surgery (abstract). J Neurol Neurosurg Psych. 1994;57:249-250	abstract only
Qiubo Q, Qi B. Study the correlation between evaluation of cerebrovascular function by 320 dynamic volume ct and neurological complications after off-pump coronary artery bypass graft. Journal of the American College of Cardiology. 2014;64:C201	abstract only
Rasmussen LA, Jakobsen CJ. Cognitive dysfunction after cardiac anaesthesia is associated with prolonged hospital stay. Intensive care medicine. 2012;38:S97	abstract only
Regieli JJ, Sauer AMC, Dieleman JM, Dijk DV, Jansen EW, Grobbee DE, Stella PS, De Jaegere PP, Doevendans PA, Nathoe HM. Long-term neurocognitive outcome after off-pump coronary artery bypass grafting versus percutaneous coronary intervention: The octopus study. Circulation. 2010;120:2155	abstract only
Reineke D, Konig T, Meszaros K, Sodeck G, Erdoes G, Englberger L, Czerny M, Carrel T. Cognitive brain function after coronary bypass grafting with minimimal invasive extracorporeal circulation. Interactive cardiovascular and thoracic surgery. 2014;19:S722	abstract only
Ringaitiene D, Norkiene I, Sipylaite J. Increasesed incidence of postoperative delirium in patients with risk of malnutrition undergoing coronary artery bypass grafting. Acta Anaesthesiologica Scandinavica, Supplement. 2013;57:20	abstract only
Rothenhausler HB. The effects of cardiac surgical procedures on health-related quality of life, cognitive performance, and emotional status outcomes: A prospective 6-month follow-up study. Psychiatria Danubina. 2010;22:135-136	abstract only
Russell D. Monitoring the brain during invasive cardiovascular examinations and surgery. European Journal of Neurology. 2018;25 (Supplement 1):1-2	abstract only
Rustenbach C, Baumbach H, Nagib R, Michaelsen J, Hipp G, Pressmar M, Leinweber M, Franke U, Pohl U, Sperandio M. Minimally invasive versus conventional extracorporeal circulation in minimally invasive cardiac valve surgery. Journal of Vascular Research. 2011;48:321-322	abstract only
Ryhammer PK, Rasmussen LA, Jakobsen CJ. Early extubation after cardiac surgery is associated with better postoperative cognitive function. Applied Cardiopulmonary Pathophysiology. 2013;17:151	abstract only
Saager L, You J, Kurz A, Turan A. Can the memorial delirium assessment scale be utilized for early identification of delirium onset? Critical care medicine. 2011;39:133	abstract only
Sabashnikov A, Choi YH, Rahmanian PB, Zeriouh M, Mehler TO, Wahlers T, Wittwer T. Outcomes in patients undergoing surgery with a minimized extracorporeal circulation compared to off pump revascularization. Thoracic and Cardiovascular Surgeon. 2012;60:no pagination	abstract only
Sahan C, Sivrikoz N, Sungur Z, Gurvit H, Senturk M, Camci E. Effects of near-infrared spectroscopy on cognitive dysfunction for patients undergoing elective coronary surgery. Applied Cardiopulmonary Pathophysiology. 2014;18:99	abstract only
Sayilgan C, Erdotan C, Yuceyar L, Erolcay H, Catlar S, Konukotlu D, Omeroglu S, Firtina S, Balciotlu I, Ipek G. Prophylactic use of pentoxifylline on cognitive function and biochemical markers for brain demage in on-pump cabg patients. European Journal of Anaesthesiology. 2011;28:63	abstract only
Scheeren TWL, van Harten AE, Kok WF, Absalom AR. Intraoperative cerebral tissue oxygenation and postoperative cognitive dysfunction after onand off-pump coronary artery bypass surgery - a randomized study. Applied Cardiopulmonary Pathophysiology. 2012;16:347-349	abstract only
Schonburg M, Schwarz N, Bachmann G, Kaps M, Klovekorn W, Sammer G, Tschernatsch M, Nottbohm R, Blaes F, Gerriets T. Early prediction of long-term neurobehavioral outcome after coronary artery bypass surgery. Thoracic and Cardiovascular Surgeon. nd;58:no pagination	abstract only
Schwarz N, Schonburg M, Mollmann H, Kastaun S, Kaps M, Bachmann G, Sammer G, Hamm C, Walter T, Gerriets T. Cognitive decline and ischemic micro-lesions after coronary catheterization. A comparison to coronary artery bypass grafting and healthy volunteers. Cerebrovascular Diseases. 2011;31:295	abstract only
Selnes OA, McKhann GM, Grega MA, Baumgartner WA, Pham L, Zeger S. Impact of coronary artery disease on long-term cognitive outcome. Annals of neurology. 2009;66:S40-S41	abstract only
Sharka I, Quka A, Sotiri E, Gjyli L, Myftiu S. Poor sleep quality and its influence on the development of acute cognitive disorders (delirium) in acute cardiac patients. European Heart Journal: Acute Cardiovascular Care. 2013;2:85-86	abstract only

Shmyrev V, Ponomarev D, Lomivorotov V. Effects of remote ischaemic preconditioning on cognitive function and neurologic injury in cardiac surgery. Applied Cardiopulmonary Pathophysiology. 2014;18:59	abstract only
Shonbin AN, Zavolozhin AS, Bystrov DO, Elisarov MV, Yarkovoy MA. 'Don't touch aorta' technique in off-pump coronary artery bypass grafting reduces the incidence of postoperative neurological complications. Interactive cardiovascular and thoracic surgery. 2011;12:S92-S93	abstract only
Siepe M, Pfeiffer T, Gieringer A, Zemann S, Benk C, Schlensak C, Beyersdorf F. Increased systemic perfusion pressure during cardiopulmonary bypass protects against postoperative cognitive dysfunction. Interactive cardiovascular and thoracic surgery. 2010;11:S85	abstract only
Silva FCP, Schmidt AP, Oses JP, Pinto KO, Carmona MJC, Auler Jr J. Increased serum levels of s100b protein and neuron-specific enolase (nse) in patients after cabg surgery: Is there any correlation with postoperative cognitive dysfunction? European Journal of Anaesthesiology. 2012;29:105	abstract only
Singh D, Lohchab SS. Nomothermic cardiopulmonary bypass: Our experience-dharmveer singh. Journal of Extra-Corporeal Technology. 2009;41:A2	abstract only
Skitek M, Jerin A, Kristl J, Marc J, Kikelj D. Serum biomarkers in ischemic neurological complications. European Journal of Pharmaceutical Sciences. 2011;44:60-61	abstract only
Skoloudik D, Hurtikova E, Brat R, Herzig R. Sonolysis in prevention of brain infarction during cardiac surgery (sonorescue): A randomized, controlled trial. Journal of the Neurological Sciences. 2015;357:e411	abstract only
Skoloudik D, Kuliha M, Hurtikova E, Roubec M, Herzig R, Sanak D, Kral M, Prochazka V, Hrbac T, Brat R. On-going clinical trials effect of sonolysis on a risk reduction of brain infarction during cardiac or carotid interventions: A prospective study. International Journal of Stroke. 2014;9:253	abstract only
Skoloudik D, Kuliha M, Roubec M, Hurtikova E, Herzig R, Kral M, Goldirova A, Prochazka V, Hrbac T, Brat R, et al. Effect of sonolysis on a risk reduction of brain infarction during cardiac or carotid interventions. A prospective study. Cerebrovascular Diseases. 2014;37:25	abstract only
Skoloudik D, Kuliha M, Roubec M, Hurtikova E, Herzig R, Kral M, Goldirova A, Prochazka V, Hrbac T, Brat R. Effect of sonolysis on a risk reduction of brain infarction during cardiac or carotid interventions. Cerebrovascular Diseases. 2014;38:95	abstract only
Soinne L, Rantanen K, Hietanen M, Vento A, Ramo J, Roine RO. Long-term cognitive decline after coronary surgery is primarily patientrelated. European journal of neurology. 2012;19:542	abstract only
Sorrentino F, Franciosi G, Torre T, Faeli M, Riva Muzio S, Siclari F. Low perioperative mortality, good survival and quality of life following cabg surgery in octogenarians. Kardiovaskulare Medizin. 2011;14:29S	abstract only
Spence J, Bosch J, Sharma M, Cukierman-Yaffe T, Canavan M, Belley-Cote E, Whitlock R, Devereaux PJ, Lamy A. Predictors of cognitive decline after cardiac surgery: An evaluation of the cabg off or on pump revascularization study (coronary) cohort. European Heart Journal. 2017;38 (Supplement 1):445-446	abstract only
Strauch J, Langebartels G, Wittwer T, Liakopoulos O, Wahlers T. On-pump cabg procedures lead to significant early postoperative increase in neurone specific enolase (nse) levels and show deficits in neurocognitive abilities. Innovations: Technology and Techniques in Cardiothoracic and Vascular Surgery. 2010;5:215-216	abstract only
Sun L, Ding S, Feng X, Wu F, Su P, Shen Y, Zhao H, Wu Y. Higher serum interieukin-6 is a strong postoperative predictor of delirium after isolated coronary artery bypass graft surgery. Circulation. 2014;130:no pagination	abstract only
Svagzdiene M, Kumpaitiene B, Sirvinskas E, Zakelis R, Petkus V, Ragauskas A, Benetis R. Cerebrovascular autoregulation impairment during cardiac surgery is related to postoperative cognitive dysfunction. Journal of Cardiothoracic and Vascular Anesthesia. 2017;31 (Supplement 1):S3-S4	abstract only
Szwed K, Bielinski M, Kaszewska A, Hoffmann A, Borkowska A. Neuropsychological assessment of cardiac rehabilitation following cardiac surgery - preliminary results. European Psychiatry. 2010;25:no pagination	abstract only
Tabaee AS, Rostami A. Comparing neurocognitive disturbances after off-pump and on-pump coronary artery bypass graft. Cardiology in the Young. 2010;20:261	abstract only
Tarasova I, Maleva O, Chernobay A, Barbarash O, Barbarash L. Impact of mild cognitive impairment on the changes in bioelectrical brain activity in patients undergoing on-pump coronary artery bypass grafting. Neurodegenerative Diseases. 2015;15:1696	abstract only

Tarasova I, Trubnikova O, Barbarash O, Barbarash L. The changes of brain activity associated with postoperative cognitive dysfunction after coronary bypass surgery. International Journal of Psychophysiology. 2018;131 (Supplement):S167	abstract only
Tarasova IV, Trubnikova OA, Syrova ID, Barbarash OL. Eeg dynamics in patients with coronary artery disease undergoing on-pump coronary bypass surgery. Interactive cardiovascular and thoracic surgery. 2011;12:S86-S87	abstract only
Thomaidou E, Megari K, Argiriadou E, Anastasiadis K, Kosmidou M, Paroutsidou G, Kiourtzieva E, Grosomanidis V, Ghalvatzoulis O, Taskos N, et al. Intraoperative cerebral desaturation as prognostic factor for the neurocognitive outcome and the prolonged hospitalization after coronary artery by pass grafting surgery. Intensive care medicine. 2010;36:S328	abstract only
Thomaidou E, Megari K, Argiriadou H, Antonitsis P, Sarlis G, Anastasiadis K, Kosmidou M, Taskos N, Bretzakis G, Stamatiou G, et al. Erythromycin and neuroprotection in coronary artery bypass surgery (cabg). Journal of cardiothoracic and vascular anesthesia. 2011;25:S40	abstract only
Toeg HD, Nathan H, Rubens F, Wozny D, Boodhwani M. Clinical impact of neurocognitive deficits following cardiac surgery. Canadian Journal of Cardiology. 2012;28:S287	abstract only
Trubnikova O, Argunova Y, Mamontova A, Barbarash O. Effect of 3 weeks of aerobic exercise training on neuropsychological status of patients undergoing coronary artery bypass grafting. European Heart Journal. 2014;35:899-900	abstract only
Trubnikova O, Tarasova I, Maleva O, Syrova I, Barbarash O. Cognitive outcomes in patients after coronary artery bypass grafting at five-year follow-up. International Journal of Psychophysiology. 2018;131 (Supplement):S169	abstract only
Trubnikova OA, Tarasova IV, Artamonova AI, Syrova ID, Barbarash OL. Dynamics of neurophysiologic parameters in coronary heart disease patients with internal carotid artery stenosis undergoing coronary bypass surgery. Interactive cardiovascular and thoracic surgery. 2011;12:S89	abstract only
Trubnikova OA, Tarasova IV, Maleva OV, Kagan ES, Barbarash OL, Barbarash LS. Factors for the development of persistent postoperative cognitive dysfunction in patients undergoing coronary artery bypass surgery under extracorporeal circulation. [russian]. Terapevticheskii Arkhiv. 2017;89:41-47	abstract only
Tsaousi G, Pitsis A, Deliaslani D, Karakoulas K, Vasilakos D. Cerebral oxygen indices impairment during off-pump coronary revascularization and its relevance to early intellectual dysfunction. European Journal of Anaesthesiology. 2009;26:60	abstract only
Tsitlidze E, Rukhadze I, Chanishvili T. Changes of polysomnography (psg) parameters in patients with cognitive disorders after cardiac surgery. Journal of Sleep Research. 2016;25:136	abstract only
Tsygan N, Andreev R, Golokhvastov S, Karpova O, Peleshok A, Kurasov E, Odinak M. Prevention of postoperative cerebral dysfunction after coronary artery bypass surgery with cardiopulmonary bypass. Cerebrovascular Diseases. 2018;45 (Supplement 1):385	abstract only
Tsygan N, Trashkov A, Andreev R, Yakovleva V, Pometko D, Khubulava G, Litvinenko I, Odinak M. Perioperative stroke and other clinical types of postoperative cerebral dysfunction in coronary artery bypass surgery. Cerebrovascular Diseases. 2018;45 (Supplement 1):53	abstract only
Vedel AG, Holmgaard F, Rasmussen L, Langkilde A, Paulson OB, Olsen PS, Lange T, Ravn HB, Nilsson JC. The influence of mean arterial pressure during cardiopulmonary bypass on cerebral complications. Journal of Cardiothoracic and Vascular Anesthesia. 2017;31 (Supplement 1):S14	abstract only
Vorobyeva M, Villevalde S, Kobalava Z. Intensive statin therapy has no impact on cognition in high cardiovascular risk patients. European Journal of Heart Failure. 2017;19 (Supplement 1):100-101	abstract only
Widmann C, Semmler A, Okulla T, Urbach H, Kaiser M, Widman G, Mormann F, Weide J, Fliessbach K, Klockgether T, et al. Sepsis and cognition. Journal of Nutrition, Health and Aging. 2012;16:866	abstract only
Wittwer T, Sabashnikov A, Choi YH, Rahmanian P, Zeriouh M, Wahlers T. Superior outcomes in patients undergoing surgery with a minimized extracorporeal circulation compared to off-pump revascularization. Innovations: Technology and Techniques in Cardiothoracic and Vascular Surgery. 2012;7:99	abstract only
Woznica A, Krzych LJ, Pawlak A, Skarysz J, Eysymontt Z, Krupka-Matuszczyk I, Krysta K, Bochenek A, Cisowski M. Evaluation of cognitive functioning in young, professionally active men after coronary bypass. European Neuropsychopharmacology. 2009;19:S359	abstract only

Yamada U, Yokota K, Ohta D, Furukawa K. Delirium could be an indicator of sepsis in patients under 65 years old with urinary tract infections. Critical Care. 2012;16:S121	abstract only
Yassin A, Wood M, Lowther C. Master follower cardioplegia versus standard cardioplegia in coronary artery bypass graft surgery. Critical care medicine. 2013;41:A72	abstract only
Yulia Argunova Y, Maleva O, Syrova I, Barbarash O. Exercise training as a rehabilitation method for early postoperative cognitive dysfunction in patients undergoing coronary artery bypass grafting. European Journal of Preventive Cardiology. 2015;22:S79	abstract only
Yulia Argunova Y, Trubnikova OA, Kagan ES, Barbarash OL. Cerebroprotective effects of physical training in patients undergoing coronary artery bypass grafting. European Journal of Preventive Cardiology. 2017;24 (1 Supplement 1):S149	abstract only
Yulia Argunova Y, Trubnikova OA, Kagan ES, Barbarash OL. High adherence to treatment as a promising method of effective prevention of postoperative cognitive dysfunction in patients after coronary artery bypass grafting. European Journal of Preventive Cardiology. 2018;25 (2 Supplement 1):S126	abstract only
Zakharov V, Hussain A, Smetkin A, Kirov M. Comparison of sevoflurane and propofol anesthesia during off-pump coronary artery bypass grafting: Effects on cerebral oxygenation and cognitive function. European Journal of Anaesthesiology. 2014;31:120	abstract only
Zllami B, Basha E, Cekrezi B, Kruja J. Correlation between white matter lesions and neurological complication after cardiac surgery. European journal of neurology. 2012;19:182	abstract only
Chernov VI, Efimova NY, Efimova IY, Akhmedov SD, Lishmanov YB. Cognitive function and cerebral perfusion in off-pump and on-pump coronary artery bypass patients. Research focus on cognitive disorders. 2007:71-85	book/chapter
Mattlar C-E, Knuts L-R, Engblom E, Vanttinen E, Willner AERG. Neuropsychological findings and personality structure associated with coronary artery bypass surgery (cabs): An eight month follow-up study. Impact of cardiac surgery on the quality of life: Neurological and psychological aspects. 1990:211-219	book/chapter
Newman S, Klinger L, Venn G, Smith P, Harrison M, Treasure T, Willner AERG. The persistence of neuropsychological deficits twelve months after coronary artery bypass surgery. Impact of cardiac surgery on the quality of life: Neurological and psychological aspects. 1990:173-179	book/chapter
Newman S, Smith P, Treasure T, Joseph P, Ell P, Harrison M, Johnston MMT. Acute neuropsychological consequences of coronary bypass artery surgery. Applications in health psychology. 1989:121-130	book/chapter
Futterman LG, Lemberg L. Encephalopathies following cardiac surgery. American journal of critical care : an official publication, American Association of Critical-Care Nurses. 1998;7:450-453	case study
Anon. Effect of remote ischemic preconditioning on cognitive function after off-pump coronary artery bypass graft. ClinicalTrials.gov 2009	clinical trial registration
Awad H, Essandoh M. Goal-directed oxygen delivery during cardiopulmonary bypass: Can this perfusion strategy improve biochemical and clinical neurologic outcomes? Journal of Cardiothoracic and Vascular Anesthesia. 2018;32:2493-2494	comment/letter
Barbero C, Centofanti P, D'Agata F, Mortara P, Rinaldi M. Late neuropsychologic outcome after hypothermic circulatory arrest: Is there cause for concern? Journal of Cardiothoracic & Vascular Anesthesia. 2018;32:e1-e3	comment/letter
Blauth C, Griffin S, Harrison M, Klinger L, Newman S, Pugsley W, Smith P, Taylor K, Treasure T, Venn G, et al. Neuropsychologic alterations after cardiac operation. Journal of Thoracic and Cardiovascular Surgery. 1989;98:454-455	comment/letter
Forcillo J, Perrault LP. If too frail, functional benefit following cardiac surgery may fail: A role for prehabilitation? Journal of Thoracic and Cardiovascular Surgery. 2017;154:2000-2001	comment/letter
Grocott HP. Anesthetic agents and postoperative delirium after off-pump coronary bypass graft surgery: An insufficiently complex approach to a complex problem. Journal of Cardiothoracic & Vascular Anesthesia. 2018;32:e87-e88	comment/letter
Hung W. Prolonged cognitive impairment after delirium among patients who had cardiac surgery. Journal of Clinical Outcomes Management. 2012;19:395-397	comment/letter

King N, Mann V. Why do on-pump patients live longer? Aging. 2018;10:2553-2554	comment/letter
Leslie M. The post-op brain: Surgery can cure-but it may take a toll on cognition. Some scientists blame a body-wide inflammatory response. Science. 2017;356:898-900	comment/letter
Lewis C, Hogue CW. Lack of benefit of near-infrared spectroscopy monitoring for improving patient outcomes. Case closed? British Journal of Anaesthesia. 2017;119:347-349	comment/letter
Li WA, Geng X, Ding Y. Stroke is a global epidemic: New developments in clinical and translational cerebrovascular diseases research. Neurological Research. 2017;39:475-476	comment/letter
Lo B, Fijnheer R, Nierich AP, Kalkman CJ, Van Dijk D. Activation of hemostasis is associated with early cognitive decline after off-pump coronary artery bypass surgery [8]. Journal of Thrombosis and Haemostasis. 2005;3:2114-2117	comment/letter
Mackenzie M, Hall R. Mental decline and cardiac surgery - should we go there? Critical Care Medicine. 2017;45:1411-1412	comment/letter
Pulido JN. Cardiac surgery blues: The midterm impact of postoperative delirium and the association with mood disorders. Journal of Thoracic and Cardiovascular Surgery. 2018;155:668-669	comment/letter
Sugita J, Fujiu K. Systemic inflammatory stress response during cardiac surgery. International Heart Journal. 2018;59:457-459	comment/letter
Ainsworth CR. Examination of post-operative cognitive decline within a cardiac sample: Analysis of the semantic clustering index on the hopkins verbal learning test-revised. Dissertation Abstracts International: Section B: The Sciences and Engineering. 2011;71:5779	dissertation
Browne SM. Neuropsychiatric complications of coronary artery bypass graft surgery. Dissertation Abstracts International: Section B: The Sciences and Engineering. 2002;63:1260	dissertation
Geishardt S. Objective and subjective neuropsychological impairment and the relationship to depression, in randomized cpb and off-pump patients, following heart surgery. Dissertation Abstracts International: Section B: The Sciences and Engineering. 2006;67:2833	dissertation
Hedges C. Sleep, mood, memory and verbal learning in off-pump coronary artery bypass patients. Dissertation Abstracts International: Section B: The Sciences and Engineering. 2004;65:157	dissertation
Kamper JE. Predictors of neuropsychological status in cardiac patients. Dissertation Abstracts International: Section B: The Sciences and Engineering. 2014;74:No-Specified	dissertation
Legendre SA. The influence of cognitive reserve on neuropsychological functioning after coronary artery bypass grafting. Dissertation Abstracts International: Section B: The Sciences and Engineering. 2004;64:5789	dissertation
Patel S. The impact of pomegranate on memory dysfunction after cardiac surgery. Dissertation Abstracts International: Section B: The Sciences and Engineering. 2011;72:3749	dissertation
Sy MJ. Neuropsychological changes after open heart surgery. Dissertation Abstracts International. 1981;42:2088	dissertation
Thompson ES. Effect of two general anesthetic agents on the cognitive function of neurologically normal adults following extracorporeal circulation for coronary artery revascularization. Dissertation Abstracts International: Section B: The Sciences and Engineering. 1997;58:2359	dissertation
Al Tmimi L, Velde M, Meyns B, Meuris B, Sergeant P, Milisen K, Pottel H, Poesen K, Rex S. Serum protein s100 as marker of postoperative delirium after off-pump coronary artery bypass surgery: Secondary analysis of two prospective randomized controlled trials. Clinical chemistry and laboratory medicine. 2017;54:1671-1680	duplicate
Alan Hall R, Fordyce DJ, Lee ME, Eisenberg B, Lee RF, Holmes IJH, Campbell WG. Brain spect imaging and neuropsychological testing in coronary artery bypass patients. Annals of Thoracic Surgery. 1999;68:2082-2088	duplicate
Bily B, Artemiou P, Sabol F, Bilecova-Rabajdova M, Kolarcik P, Torok P. The role of dexmedetomidine in the prevention of postoperative delirium in cardiac surgery patients. Cardiology Letters. 2015;24:435-444	duplicate

Engelhardt W, Dierks T, Pause M, Hartung E, Haaland K, Temkin N, Randahl G, Dikmen S. Early cerebral functional outcome after coronary artery bypass surgery using different acid-base management during hypothermic cardiopulmonary bypass. Recovery of simple motor skills after head injury. Acta anaesthesiologica Scandinavica. 1996;40:457-465	duplicate
Gorna R, Kustrzycki W, Kiejna A, Rymaszewska J. Assessment of short-term neuropsychchologic changes after normathermic versus hypothermic coronary artery bypass grafting. Ocena bezposrednich zmian funkcji poznawczych u pacjentow poddanych chirurgicznej rewaskularyzacji wiencowej przeprowadzonej w hipotermii i normotermii. 2001;35:781-795	duplicate
Kastaun S, Gerriets T, Schwarz N, Tschernatsch M, Kaps M, Walther T, Schoenburg M. Pseudo-hallucinations following heart surgery in patients with normal vision. Thoracic and Cardiovascular Surgeon. 2011;59:no pagination	duplicate
Khosravi A, Skrabal C, Westphal B, Kundt G, Greim B, Kunesch E, Liebold A, Steinhoff G. Evaluation of coated oxygenators in cardiopulmonary-bypass systems and their impact on neurocognitive function. Perfusion. 2012;20:249-254	duplicate
koloudík D, Hurtíková E, Brát R, Herzig R. Sonolysis in prevention of brain infarction during cardiac surgery (sonorescue): Randomized, controlled trial. Medicine (Baltimore). 2017;95:e3615	duplicate
Meybohm P, Renner J, Broch O, Caliebe D, Albrecht M, Cremer J, Haake N, Scholz J, Zacharowski K, Bein B. Postoperative neurocognitive dysfunction in patients undergoing cardiac surgery after remote ischemic preconditioning: A double-blind randomized controlled pilot study. PloS one. 2016;8:e64743	duplicate
Mongero L, Beck, Jr., Manspeizer H, Heyer E, Lee K, Spanier T, Smith C. Cardiac surgical patients exposed to heparin-bonded circuits develop less postoperative cerebral dysfunction than patients exposed to non-heparin-bonded circuits. Perfusion. 2012;16:107-111	duplicate
Rothenhausler HB, Stepan A, Kapfhammer HP. The effects of cardiac surgical procedures on health-related quality of life, cognitive performance, and emotional status outcomes: A prospective 6-month follow-up study. Journal of Psychosomatic Research. 2010;68:660	duplicate
Sauër A, Nathoe H, Hendrikse J, Peelen L, Regieli J, Veldhuijzen D, Kalkman C, Grobbee D, Doevendans P, Dijk D. Cognitive outcomes 7.5 years after angioplasty compared with off-pump coronary bypass surgery. Ann Thorac Surg. 2013;96:1294-1300	duplicate
Selenes OA, Royall RM, Grega MA, Borowicz LM, Jr., Quaskey S, McKhann G, Aberg BBBBBBBCCdlTdLDDFFFGGGGHHKLLMM. Cognitive changes 5 years after coronary artery bypass grafting. Archives of neurology. 2001;58:598-604	duplicate
Shroyer AL, Grover FL, Hattler B, Collins JF, McDonald GO, Kozora E, Lucke JC, Baltz JH, Novitzky D. On-pump versus off-pump coronary-artery bypass surgery. New England Journal of Medicine. 2009;361:1827-1837	duplicate
Spence J, Bosch J, Sharma M, Cukierman-Yaffe T, Canavan M, Belley-Cote E, Whitlock R, Devereaux P, Lamy A. Predictors of cognitive decline after cardiac surgery: An evaluation of the cabg off or on pump revascularization study (coronary) cohort. Canadian Journal of Cardiology. 2017;33 (10 Supplement 1):S36	duplicate
Stevens R, Gersbach P, Ruchat P, Hurni M, Stumpe F, Fischer A, Sadeghi H. Open-heart surgery in octogenarians. Schweizerische Medizinische Wochenschrift. 1995;125:2084-2089	duplicate
Whitaker D, Green A, Stygall J, Harrison M, Newman S. Evaluation of an alternative s100b assay for use in cardiac surgery: Relationship with microemboli and neuropsychological outcome. Perfusion. 2012;22:267-272	duplicate
Abu-Omar Y, Cifelli A, Matthews PM, Taggart DP. The role of microembolisation in cerebral injury as defined by functional magnetic resonance imaging. European journal of cardio-thoracic surgery : official journal of the European Association for Cardio-thoracic Surgery. 2004;26:586-591	inappropriate data
Ahlgren E, Lundqvist A, Nordlund A, Aren C, Rutberg H. Neurocognitive impairment and driving performance after coronary artery bypass surgery. European journal of cardio-thoracic surgery : official journal of the European Association for Cardio-thoracic Surgery. 2003;23:334-340	inappropriate data
Al TL, Hemelrijck J, Velde M, Sergeant P, Meyns B, Missant C, Jochmans I, Poesen K, Coburn M, Rex S. Xenon anaesthesia for patients undergoing off-pump coronary artery bypass graft surgery: A prospective randomized controlled pilot trial. Br J Anaesth. 2015;115:550-559	inappropriate data

Al Tmimi L, Devroe S, Dewinter G, Van De Velde M, Poortmans G, Meyns B, Meuris B, Coburn M, Rex S. Xenon as an adjuvant to propofol anesthesia in patients undergoing off-pump coronary artery bypass graft surgery: A pragmatic randomized controlled clinical trial. Anesthesia and Analgesia. 2017;125:1118-1128	inappropriate data
Alex J, Laden G, Cale ARJ, Bennett S, Flowers K, Madden L, Gardiner E, McCollum PT, Griffin SC. Pretreatment with hyperbaric oxygen and its effect on neuropsychometric dysfunction and systemic inflammatory response after cardiopulmonary bypass: A prospective randomized double-blind trial. The Journal of thoracic and cardiovascular surgery. 2005;130:1623-1630	inappropriate data
Anastasiadis K, Argiriadou H, Kosmidis M, Megari K, Antonitsis P, Thomaidou E, Aretouli E, Papakonstantinou C. Neurocognitive outcome after coronary artery bypass surgery using minimal versus conventional extracorporeal circulation: A randomised controlled pilot study. Heart. 2011;97:1082-1088	inappropriate data
Andrew MJ, Baker RA, Bennetts J, Kneebone AC, Knight JL. A comparison of neuropsychologic deficits after extracardiac and intracardiac surgery. Journal of cardiothoracic and vascular anesthesia. 2001;15:9-14	inappropriate data
Andrew MJ, Baker RA, Kneebone AC, Knight JL. Neuropsychological dysfunction after minimally invasive direct coronary artery bypass grafting. The Annals of thoracic surgery. 1998;66:1611-1617	inappropriate data
Antony SP, Jamuna R, Kini SM, Chakravarthy M, Alexander BBBBBBBBBCCCCDDEF-SGGGHHKKLLMMM. Neuropsychological deficits in patients with myocardial infarction. Neuropsychological Trends. 2010;7:37-50	inappropriate data
Arrowsmith J, Harrison M, Newman S, Stygall J, Timberlake N, Pugsley W. Neuroprotection of the brain during cardiopulmonary bypass: A randomized trial of remacemide during coronary artery bypass in 171 patients. Stroke. 1998;29:2357-2362	inappropriate data
Askar FZ, Cetin HY, Kumral E, Cetin O, Acarer A, Kosova B, Yagdi T. Apolipoprotein e epsilon4 allele and neurobehavioral status after on-pump coronary artery bypass grafting. Journal of Cardiac Surgery. 2005;20:501-505	inappropriate data
Aykut K, Albayrak G, Guzeloglu M, Hazan E, Tufekci M, Erdogan I. Pulsatile versus non-pulsatile flow to reduce cognitive decline after coronary artery bypass surgery: A randomized prospective clinical trial. Journal of Cardiovascular Disease Research. 2013;4:127-129	inappropriate data
Baker R, Andrew M, Ross I, Knight J. The octopus ii stabilizing system: Biochemical and neuropsychological outcomes in coronary artery bypass surgery. Heart Surg Forum. 2001;4 Suppl 1:S19-23	inappropriate data
Bappu NJ, Venugopal P, Bisoi AK, Mankad PS. Troponin-i release after cardiac surgery with different surgical techniques and post-operative neurological outcomes. McGill journal of medicine : MJM : an international forum for the advancement of medical sciences by students. 2006;9:88-94	inappropriate data
Barbut D, Hinton RB, Szatrowski TP, Hartman GS, Bruefach M, Williams-Russo P, Charlson ME, Gold JP. Cerebral emboli detected during bypass surgery are associated with clamp removal. Stroke. 1994;25:2398-2402	inappropriate data
Bar-Yosef S, Anders M, Mackensen GB, Ti LK, Mathew JP, Phillips-Bute B, Messier RH, Grocott HP. Aortic atheroma burden and cognitive dysfunction after coronary artery bypass graft surgery. Annals of Thoracic Surgery. 2004;78:1556-1562	inappropriate data
Bassano C, Bovio E, Uva F, Iacobelli S, Iasevoli N, Farinaccio A, Ruvolo G. Partially anaortic clampless off-pump coronary artery bypass prevents neurologic injury compared to on-pump coronary surgery: A propensity score-matched study on 286 patients. Heart and vessels. 2016;31:1412-1417	inappropriate data
Bayindir O, Akpinar B, Can E, Guden M, Sonmez B, Demiroglu C. The use of the 5-ht3-receptor antagonist ondansetron for the treatment of postcardiotomy delirium. Journal of cardiothoracic and vascular anesthesia. 2000;14:288-292	inappropriate data
Bendszus M, Reents W, Franke D, Mullges W, Babin-Ebell J, Koltzenburg M, Warmuth-Metz M, Solymosi L. Brain damage after coronary artery bypass grafting. Archives of neurology. 2002;59:1090-1095	inappropriate data
Bi Q, Jun-Yu L, Xiao-Qing L, Qin L, Di L, Qiu-Bo Q. The impact of intracranial artery disease and prior cerebral infarction on central nervous system complications after off-pump coronary artery bypass grafting. Journal of Neurological Sciences. 2014;31:247-256	inappropriate data
Bi Q, Li JY, Li XQ, Li Q, Luo D, Qiao QB. Impact of intracranial artery disease and prior cerebral infarction on central nervous system complications after off-pump coronary artery bypass grafting. Neurophysiology. 2015:no pagination	inappropriate data

Bonacchi M, Prifti E, Maiani M, Bartolozzi F, Di Eusanio M, Leacche M. Does off-pump coronary revascularization reduce the release of the cerebral markers, s-100beta and nse? Heart, Lung & Circulation. 2006;15:314-319	inappropriate data
Boodhwani M, Rubens F, Wozny D, Rodriguez R, Nathan HJ. Effects of sustained mild hypothermia on neurocognitive function after coronary artery bypass surgery: A randomized, double-blind study. The Journal of thoracic and cardiovascular surgery. 2007;134:1443-1442	inappropriate data
Borger MA, Peniston CM, Weisel RD, Vasiliou M, Green RE, Feindel CM. Neuropsychologic impairment after coronary bypass surgery: Effect of gaseous microemboli during perfusionist interventions. The Journal of thoracic and cardiovascular surgery. 2001;121:743-749	inappropriate data
Borger MA, Rao V. Temperature management during cardiopulmonary bypass: Effect of rewarming rate on cognitive dysfunction. Seminars in cardiothoracic and vascular anesthesia. 2002;6:17-20	inappropriate data
Browne SM, Halligan PW, Wade DT, Taggart DP. Cognitive performance after cardiac operation: Implications of regression toward the mean. The Journal of thoracic and cardiovascular surgery. 1999;117:481-485	inappropriate data
Bruce KM, Yelland GW, Smith JA, Robinson SR. Recovery of cognitive function after coronary artery bypass graft operations. The Annals of thoracic surgery. 2013;95:1306-1313	inappropriate data
Bruggemans EF, Van de Vijver FJ, Huysmans HA. Assessment of cognitive deterioration in individual patients following cardiac surgery: Correcting for measurement error and practice effects. Journal of clinical and experimental neuropsychology. 1997;19:543-559	inappropriate data
Bucerius J, Gummert JF, Borger MA, Walther T, Doll N, Falk V, Schmitt DV, Mohr FW. Predictors of delirium after cardiac surgery delirium: Effect of beating-heart (off-pump) surgery. The Journal of thoracic and cardiovascular surgery. 2004;127:57-64	inappropriate data
Butterworth J, Legault C, Stump DA, Coker L, Hammon JW, Jr., Troost BT, Royster RL, Prough DS. A randomized, blinded trial of the antioxidant pegorgotein: No reduction in neuropsychological deficits, inotropic drug support, or myocardial ischemia after coronary artery bypass surgery. Journal of cardiothoracic and vascular anesthesia. 1999;13:690-694	inappropriate data
Butterworth J, Wagenknecht LE, Legault C, Zaccaro DJ, Kon ND, Hammon Jr JW, Rogers AT, Troost BT, Stump DA, Furberg CD, et al. Attempted control of hyperglycemia during cardiopulmonary bypass fails to improve neurologic or neurobehavioral outcomes in patients without diabetes mellitus undergoing coronary artery bypass grafting. Journal of Thoracic and Cardiovascular Surgery. 2005;130:1319	inappropriate data
Buziashvili II, Ambat'ello SG, Aleksakhina IA, Pashchenkov MV. Influence of cardiopulmonary bypass on cognitive functions in patients with ischemic heart disease. Zhurnal nevrologii i psikhiatrii imeni S.S. Korsakova / Ministerstvo zdravookhraneniia i meditsinskoi promyshlennosti Rossiiskoi Federatsii, Vserossiiskoe obshchestvo nevrologov [i] Vserossiiskoe obshchestvo psikhiatrov. 2005;105:30-35	inappropriate data
Carella F, Travaini G, Contri P, Guzzetti S, Botta M, Pieri E, Mangoni A. Cerebral complications of coronary by-pass surgery. A prospective study. Acta neurologica Scandinavica. 1988;77:158-163	inappropriate data
Chakravarthy M, Manjunath V, Jawali V, Patil T, Jayaprakash K, Kalligudd P, Prabhakumar D, Kolar S, Manohar M, Basavaraj G, et al. Neurocognitive behaviour changes in patients undergoing off pump coronary artery bypass surgery- a prospective observational study. Journal of Anaesthesiology Clinical Pharmacology. 2008;24:49-52	inappropriate data
Chandarana PC, Cooper AJ, Goldbach MM, Coles JC, Vesely MA. Perceptual and cognitive deficit following coronary artery bypass surgery. Stress Medicine. 1988;4:163-171	inappropriate data
Chaudhury S, Sharma S, Pawar AA, Kumar BK, Srivastava K, Sudarsanan S, Singh D. Psychological correlates of outcome after coronary artery bypass graft. Medical Journal Armed Forces India. 2006;62:220-223	inappropriate data
Cheng DC, Karski J, Peniston C, Asokumar B, Raveendran G, Carroll J, Nierenberg H, Roger S, Mickle D, Tong J, et al. Morbidity outcome in early versus conventional tracheal extubation after coronary artery bypass grafting: A prospective randomized controlled trial. The Journal of thoracic and cardiovascular surgery. 1996;112:755-764	inappropriate data

Chernov VI, Efimova NY, Efimova IY, Akhmedov SD, Lishmanov YB. Short-term and long-term cognitive function and cerebral perfusion in off-pump and on-pump coronary artery bypass patients. European journal of cardio-thoracic surgery : official journal of the European Association for Cardio-thoracic Surgery. 2006;29:74-81	inappropriate data
Chokron S, Helft G, Perez C. Effects of age and cardiovascular disease on selective attention. Cardiovascular psychiatry and neurology. 2013;2013:185385	inappropriate data
Dabrowski W, Rzecki Z, Czajkowski M, Pilat J, Biernacka J, Kotlinska E, Pasternak K, Stazka K, Sztanke M, Sztanke K. Plasma matrix metalloproteinase 9 correlates with disorders of brain magnesium homeostasis in patients undergoing coronary artery bypass surgery. Magnesium research. 2010;23:169-179	inappropriate data
de Baar M, Diephuis JC, Moons KGM, Holtkamp J, Hijman R, Kalkman CJ. The effect of zero-balanced ultrafiltration during cardiopulmonary bypass on s100b release and cognitive function. Perfusion. 2003;18:9-14	inappropriate data
de Tournay-Jette E, Dupuis G, Denault A, Cartier R, Bherer L. The benefits of cognitive training after a coronary artery bypass graft surgery. Journal of Behavioral Medicine. 2012;35:557-568	inappropriate data
Djaiani G, Ali M, Borger MA, Woo A, Carroll J, Feindel C, Fedorko L, Karski J, Rakowski H. Epiaortic scanning modifies planned intraoperative surgical management but not cerebral embolic load during coronary artery bypass surgery. Anesthesia and analgesia. 2008;106:1611-1618	inappropriate data
Djaiani G, Fedorko L, Borger MA, Green R, Carroll J, Marcon M, Karski J. Continuous-flow cell saver reduces cognitive decline in elderly patients after coronary bypass surgery. Circulation. 2007;116:1888-1895	inappropriate data
Djaiani G, Katznelson R, Fedorko L, Rao V, Green R, Carroll J, Katski J. Early benefit of preserved cognitive function is not sustained at one-year after cardiac surgery: A longitudinal follow-up of the randomized controlled trial. Canadian journal of anaesthesia = Journal canadien d'anesthesie. 2012;59:449-455	inappropriate data
Dowd NP, Karski JM, Cheng DC, Gajula S, Seneviratne P, Munro JA, Fiducia D. Fast-track cardiac anaesthesia in the elderly: Effect of two different anaesthetic techniques on mental recovery. British Journal of Anaesthesia. 2001;86:68-76	inappropriate data
Dumas A, Dupuis GH, Searle N, Cartier R. Early versus late extubation after coronary artery bypass grafting: Effects on cognitive function. Journal of cardiothoracic and vascular anesthesia. 1999;13:130-135	inappropriate data
Ebert AD, Walzer TA, Huth C, Herrmann M. Early neurobehavioral disorders after cardiac surgery: A comparative analysis of coronary artery bypass graft surgery and valve replacement. Journal of cardiothoracic and vascular anesthesia. 2001;15:15-19	inappropriate data
Ellis RJ, Wisniewski A, Potts R, Calhoun C, Loucks P, Wells MR. Reduction of flow rate and arterial pressure at moderate hypothermia does not result in cerebral dysfunction. Journal of Thoracic and Cardiovascular Surgery. 1980;79:173-180	inappropriate data
Engelhardt W, Dierks T, Pause M, Hartung E, Haaland K, Temkin N, Randahl G, Dikmen S. Early cerebral functional outcome after coronary artery bypass surgery using different acid-base management during hypothermic cardiopulmonary bypass. Acta anaesthesiologica Scandinavica. 1996;40:457-465	inappropriate data
Ernest C, Worcester M, Tatoulis J, Elliott P, Murphy B, Higgins R, Grande M, Goble A. Neurocognitive outcomes in off-pump versus on-pump bypass surgery: A randomized controlled trial. Ann Thorac Surg. 2006;81:2105-2114	inappropriate data
Eryomina OV, Petrova MM, Prokopenko SV, Mozheyko EY, Kaskaeva DS, Gavrilyuk OA. The effectiveness of the correction of cognitive impairment using computer-based stimulation programs for patients with coronary heart disease after coronary bypass surgery. Journal of the Neurological Sciences. 2015;358:188-192	inappropriate data
Evered L, Scott DA, Silbert B, Maruff P. Postoperative cognitive dysfunction is independent of type of surgery and anesthetic. Anesthesia and analgesia. 2011;112:1179-1185	inappropriate data
Evered LA, Silbert BS, Scott DA, Maruff P, Ames D. Prevalence of dementia 7.5 years after coronary artery bypass graft surgery. Anesthesiology. 2016;125:62-71	inappropriate data
Evered LA, Silbert BS, Scott DA, Maruff P, Laughton KM, Volitakis I, Cowie T, Cherny RA, Masters CL, Li QX. Plasma amyloid beta42 and amyloid beta40 levels are associated with early cognitive dysfunction after cardiac surgery. Annals of Thoracic Surgery. 2009;88:1426-1432	inappropriate data

Evered LA, Silbert BS, Scott DA. Postoperative cognitive dysfunction and aortic atheroma. The Annals of thoracic surgery. 2010;89:1091-1097	inappropriate data
Farhoudi M, Mehrvar K, Afrasiabi A, Parvizi R, Khalili AA, Nasiri B, Hashemzadeh K, Ghabili K. Neurocognitive impairment after off-pump and on-pump coronary artery bypass graft surgery - an iranian experience. Neuropsychiatric disease and treatment. 2010;6:775-778	inappropriate data
Farsak B, Gunaydin S, Yorgancioglu C, Zorlutuna Y. Elevated levels of s-100beta correlate with neurocognitive outcome after cardiac surgery. The Journal of cardiovascular surgery. 2003;44:31-35	inappropriate data
Fitch JCK, Rollins S, Matis L, Alford B, Aranki S, Collard CD, Dewar M, Elefteriades J, Hines R, Kopf G, et al. Pharmacology and biological efficacy of a recombinant, humanized, single-chain antibody c5 complement inhibitor in patients undergoing coronary artery bypass graft surgery with cardiopulmonary bypass. Circulation. 1999;100:2499-2506	inappropriate data
Folks DG, Freeman AM, 3rd, Sokol RS, Govier AV, Reves JG, Baker DM. Cognitive dysfunction after coronary artery bypass surgery: A case-controlled study. Southern medical journal. 1988;81:202-206	inappropriate data
Formica F, Tata G, Singh G, Mariani S, D'Alessandro S, Messina LA, Sangalli F, Paolini G. Incidence of perioperative stroke in clampless aortic anastomosis during off-pump coronary artery bypass grafting. Heart & Vessels. 2018;33:595-604	inappropriate data
Forrest CM, Mackay GM, Oxford L, Millar K, Darlington LG, Higgins MJ, Stone TW. Kynurenine metabolism predicts cognitive function in patients following cardiac bypass and thoracic surgery. Journal of Neurochemistry. 2011;119:136-152	inappropriate data
Gasparovic H, Borojevic M, Malojcic B, Gasparovic K, Biocina B. Single aortic clamping in coronary artery bypass surgery reduces cerebral embolism and improves neurocognitive outcomes. Vascular medicine (London, England). 2013;18:275-281	inappropriate data
Gasparovic H, Kopjar T, Rados M, Anticevic A, Rados M, Malojcic B, Ivancan V, Fabijanic T, Cikes M, Milicic D, et al. Impact of remote ischemic preconditioning preceding coronary artery bypass grafting on inducing neuroprotection. Journal of Thoracic & Cardiovascular Surgery. 2018;06:06	inappropriate data
Gerriets T, Schwarz N, Bachmann G, Kaps M, Kloevekorn WP, Sammer G, Tschernatsch M, Nottbohm R, Blaes F, Schonburg M. Evaluation of methods to predict early long-term neurobehavioral outcome after coronary artery bypass grafting. American Journal of Cardiology. 2010;105:1095-1101	inappropriate data
Gold J, Charlson M, Williams-Russo P, Szatrowski T, Peterson J, Pirraglia P, Hartman G, Yao F, Hollenberg J, Barbut D. Improvement of outcomes after coronary artery bypass. A randomized trial comparing intraoperative high versus low mean arterial pressure. J Thorac Cardiovasc Surg. 1995;110:1302-1311; discussion 1311	inappropriate data
Goto T, Baba T, Honma K, Shibata Y, Arai Y, Uozumi H, Okuda T. Magnetic resonance imaging findings and postoperative neurologic dysfunction in elderly patients undergoing coronary artery bypass grafting. The Annals of thoracic surgery. 2001;72:137-142	inappropriate data
Goto T, Baba T, Ito A, Maekawa K, Koshiji T. Gender differences in stroke risk among the elderly after coronary artery surgery. Anesthesia and analgesia. 2007;104:1016-contents	inappropriate data
Goto T, Baba T, Matsuyama K, Honma K, Ura M, Koshiji T. Aortic atherosclerosis and postoperative neurological dysfunction in elderly coronary surgical patients. The Annals of thoracic surgery. 2003;75:1912-1918	inappropriate data
Griffin S, Klinger L, Newman S, Hothersall J, McLean P, Harrison M, Sturridge M, Treasure T. The effect of substrate load and blood glucose management on cerebral dysfunction following cardiopulmonary bypass. Vascular Surgery. 1992;26:656-664	inappropriate data
Grigore AM, Grocott HP, Mathew JP, Phillips-Bute B, Stanley TO, Butler A, Reves JG, Blumenthal JA, Newman MF, Clements FM, et al. The rewarming rate and increased peak temperature alter neurocognitive outcome after cardiac surgery. Anesthesia and analgesia. 2002;94:4-10	inappropriate data
Grigore AM, Mathew J, Grocott HP, Reves JG, Blumenthal JA, White WD, Smith PK, Jones RH, Kirchner JL, Mark DB, et al. Prospective randomized trial of normothermic versus hypothermic cardiopulmonary bypass on cognitive function after coronary artery bypass graft surgery. Anesthesiology. 2001;95:1110-1119	inappropriate data
Grimm M, Czerny M, Baumer H, Kilo J, Madl C, Kramer L, Rajek A, Wolner E. Normothermic cardiopulmonary bypass is beneficial for cognitive brain function after coronary artery bypass grafting - a prospective randomized trial. European Journal of Cardio-Thoracic Surgery. 2000;18:270-275	inappropriate data

Grocott HP, Mackensen GB, Grigore AM, Mathew J, Reves JG, Phillips-Bute B, Smith PK, Newman MF. Postoperative hyperthermia is associated with cognitive dysfunction after coronary artery bypass graft surgery. Stroke. 2002;33:537-541	inappropriate data
Gunaydin B, Babacan A. Cerebral hypoperfusion after cardiac surgery and anesthetic strategies: A comparative study with high dose fentanyl and barbiturate anesthesia. Annals of thoracic and cardiovascular surgery : official journal of the Association of Thoracic and Cardiovascular Surgeons of Asia. 1998;4:12-17	inappropriate data
Haljan G, Maitland A, Buchan A, Arora RC, King M, Haigh J, Culleton B, Faris P, Zygun D. The erythropoietin neuroprotective effect: Assessment in cabg surgery (tenpeaks): A randomized, double-blind, placebo controlled, proof-of-concept clinical trial. Stroke. 2009;40:2769-2775	inappropriate data
Hall MW, Hopkins RO, Long JW, Mohammad SF, Solen KA. Hypothermia-induced platelet aggregation and cognitive decline in coronary artery bypass surgery: A pilot study. Perfusion. 2005;20:157-167	inappropriate data
Hammeke TA, Hastings JE. Neuropsychologic alterations after cardiac operation. The Journal of thoracic and cardiovascular surgery. 1988;96:326-331	inappropriate data
Hammon J, Stump D, Butterworth J, Moody D, Rorie K, Deal D, Kincaid E, Oaks T, Kon N. Coronary artery bypass grafting with single cross-clamp results in fewer persistent neuropsychological deficits than multiple clamp or off-pump coronary artery bypass grafting. Ann Thorac Surg. 2007;84:1174-1178; discussion 1178	inappropriate data
Hammon J, Stump D, Butterworth J, Moody D, Rorie K, Deal D, Kincaid E, Oaks T, Kon N. Single crossclamp improves 6-month cognitive outcome in high-risk coronary bypass patients: The effect of reduced aortic manipulation. J Thorac Cardiovasc Surg. 2006;131:114-121	inappropriate data
Harrison MJG, Schneidau A, Ho R, Smith PLC, Newman S, Treasure T. Cerebrovascular disease and functional outcome after coronary artery bypass surgery. Stroke. 1989;20:235-237	inappropriate data
Hedges C. Sleep, memory, and learning in off-pump coronary artery bypass patients. Research in nursing & health. 2005;28:462-473	inappropriate data
Hernandez F, Jr., Brown JR, Likosky DS, Clough RA, Hess AL, Roth RM, Ross CS, Whited CM, O'Connor GT, Klemperer JD. Neurocognitive outcomes of off-pump versus on-pump coronary artery bypass: A prospective randomized controlled trial. The Annals of thoracic surgery. 2007;84:1897-1903	inappropriate data
Herrmann M, Ebert AD, Tober D, Hann J, Huth C. A contrastive analysis of release patterns of biochemical markers of brain damage after coronary artery bypass grafting and valve replacement and their association with the neurobehavioral outcome after cardiac surgery. European journal of cardio-thoracic surgery : official journal of the European Association for Cardio-thoracic Surgery. 1999;16:513-518	inappropriate data
Heyer EJ, Adams DC, Delphin E, McMahon DJ, Steneck SD, Oz MC, Miehler RE, Rose EA. Cerebral dysfunction after coronary artery bypass grafting done with mild or moderate hypothermia. Journal of Thoracic and Cardiovascular Surgery. 1997;114:270-277	inappropriate data
Heyer EJ, Lee KS, Manspeizer HE, Mongero L, Spanier TB, Caliste X, Esrig B, Smith C. Heparin-bonded cardiopulmonary bypass circuits reduce cognitive dysfunction. Journal of cardiothoracic and vascular anesthesia. 2002;16:37-42	inappropriate data
Hlatky MA, Bacon C, Boothroyd D, Mahanna E, Reves JG, Newman MF, Johnstone I, Winston C, Brooks MM, Rosen AD, et al. Cognitive function 5 years after randomization to coronary angioplasty or coronary artery bypass graft surgery. Circulation. 1997;96:II-15	inappropriate data
Ho PM, Arciniegas DB, Grigsby J, McCarthy M, Jr., McDonald GO, Moritz TE, Shroyer AL, Sethi GK, Henderson WG, London MJ, et al. Predictors of cognitive decline following coronary artery bypass graft surgery. The Annals of thoracic surgery. 2004;77:597-603	inappropriate data
Hofland J, Ouattara A, Fellahi JL, Gruenewald M, Hazebroucq J, Ecoffey C, Joseph P, Heringlake M, Steib A, Coburn M, et al. Effect of xenon anesthesia compared to sevoflurane and total intravenous anesthesia for coronary artery bypass graft surgery on postoperative cardiac troponin release an international, multicenter, phase 3, single-blinded, randomized noninferiority trial. Anesthesiology. 2017;127:918-933	inappropriate data
Hudetz JA, Gandhi SD, Iqbal Z, Patterson KM, Pagel PS. Elevated postoperative inflammatory biomarkers are associated with short- and medium-term cognitive dysfunction after coronary artery surgery. Journal of Anesthesia. 2011;25:1-9	inappropriate data

Hudetz JA, Iqbal Z, Gandhi SD, Patterson KM, Byrne AJ, Pagel PS. Postoperative delirium and short-term cognitive dysfunction occur more frequently in patients undergoing valve surgery with or without coronary artery bypass graft surgery compared with coronary artery bypass graft surgery alone: Results of a pilot study. Journal of cardiothoracic and vascular anesthesia. 2011;25:811-816	inappropriate data
Ito A, Goto T, Maekawa K, Baba T, Mishima Y, Ushijima K. Postoperative neurological complications and risk factors for pre-existing silent brain infarction in elderly patients undergoing coronary artery bypass grafting. Journal of Anesthesia. 2012;26:405-411	inappropriate data
Jensen BO, Hughes P, Rasmussen LS, Pedersen PU, Steinbruchel DA. Cognitive outcomes in elderly high-risk patients after off-pump versus conventional coronary artery bypass grafting: A randomized trial. Circulation. 2006;113:2790-2795	inappropriate data
Jensen BO, Rasmussen LS, Steinbruchel DA. Cognitive outcomes in elderly high-risk patients 1 year after off-pump versus on-pump coronary artery bypass grafting. A randomized trial. European journal of cardio-thoracic surgery : official journal of the European Association for Cardio-thoracic Surgery. 2008;34:1016-1021	inappropriate data
Jonsson H, Johnsson P, Ailing C, Backstrom M, Bergh C, Blomquist S. S100beta after coronary artery surgery: Release pattern, source of contamination, and relation to neuropsychological outcome. Annals of Thoracic Surgery. 1999;68:2202-2208	inappropriate data
Jonsson H, Johnsson P, Backstrom M, Alling C, Dautovic-Bergh C, Blomquist S. Controversial significance of early s100b levels after cardiac surgery. BMC neurology. 2004;4:24	inappropriate data
Joung K-W, Rhim J-H, Chin J-H, Kim W-J, Choi D-K, Lee E-H, Hahm K-D, Sim J-Y, Choi I-C. Effect of remote ischemic preconditioning on cognitive function after off-pump coronary artery bypass graft: A pilot study. Korean Journal of Anesthesiology. 2013;65:418-424	inappropriate data
Kadoi Y, Saito S, Fujita N, Mizutani A. Effects of balloon-induced pulsatile perfusion on postoperative short- and long-term cognitive dysfunction in diabetic patients with impaired cerebrovascular carbon dioxide reactivity. Journal of cardiothoracic and vascular anesthesia. 2013;27:238-244	inappropriate data
Kanbak M, Saricaoglu F, Avci A, Ocal T, Koray Z, Aypar U. Propofol offers no advantage over isoflurane anesthesia for cerebral protection during cardiopulmonary bypass: A preliminary study of s-100beta protein levels. Canadian journal of anaesthesia = Journal canadien d'anesthesie. 2004;51:712-717	inappropriate data
Kaukinen L, Porkkala H, Kaukinen S, Pehkonen E, Karkela J, Aaran RK, Tarkka M. Release of brain-specific creatine kinase and neuron-specific enolase into cerebrospinal fluid after hypothermic and normothermic cardiopulmonary bypass in coronary artery surgery. Acta anaesthesiologica Scandinavica. 2000;44:361-368	inappropriate data
Kazmierski J, Banys A, Latek J, Bourke J, Jaszewski R. Cortisol levels and neuropsychiatric diagnosis as markers of postoperative delirium: A prospective cohort study. Critical Care (London, England). 2013;17:R38	inappropriate data
Keizer AMA, Hijman R, Kalkman CJ, Kahn RS, Van Dijk D. The incidence of cognitive decline after (not) undergoing coronary artery bypass grafting: The impact of a controlled definition. Acta anaesthesiologica Scandinavica. 2005;49:1232-1235	inappropriate data
Kempfert J, Opfermann UT, Richter M, Bossert T, Mohr FW, Gummert JF. Twelve-month patency with the pas-port proximal connector device: A single center prospective randomized trial. The Annals of thoracic surgery. 2008;85:1579-1584	inappropriate data
Kinnunen EM, De Feo M, Reichart D, Tauriainen T, Gatti G, Onorati F, Maschietto L, Bancone C, Fiorentino F, Chocron S, et al. Incidence and prognostic impact of bleeding and transfusion after coronary surgery in low-risk patients. Transfusion. 2017;57:178-186	inappropriate data
Kneebone AC, Andrew MJ, Baker RA, Knight JL. Neuropsychologic changes after coronary artery bypass grafting: Use of reliable change indices. The Annals of thoracic surgery. 1998;65:1320-1325	inappropriate data
Kneebone AC, Luszcz MA, Baker RA, Knight JL. A syndromal analysis of neuropsychological outcome following coronary artery bypass graft surgery. Journal of neurology, neurosurgery, and psychiatry. 2005;76:1121-1127	inappropriate data
Knipp SC, Matatko N, Wilhelm H, Schlamann M, Thielmann M, Losch C, Diener HC, Jakob H. Cognitive outcomes three years after coronary artery bypass surgery: Relation to diffusion-weighted magnetic resonance imaging. The Annals of thoracic surgery. 2008;85:872-879	inappropriate data

Knipp SC, Weimar C, Schlamann M, Schweter S, Wendt D, Thielmann M, Benedik J, Jakob H. Early and long-term cognitive outcome after conventional cardiac valve surgery. Interactive Cardiovascular & Thoracic Surgery. 2017;24:534-540	inappropriate data
Kohn A. Magnetic resonance imaging registration and quantitation of the brain before and after coronary artery bypass graft surgery. The Annals of thoracic surgery. 2002;73:S363-365	inappropriate data
Kok WF, Van Harten AE, Koene BMJA, Mariani MA, Koerts J, Tucha O, Absalom AR, Scheeren TWL. A pilot study of cerebral tissue oxygenation and postoperative cognitive dysfunction among patients undergoing coronary artery bypass grafting randomised to surgery with or without cardiopulmonary bypass. Anaesthesia. 2014;69:613-622	inappropriate data
Kong RS, Butterworth J, Aveling W, Stump DA, Harrison MJG, Hammon J, Stygall J, Rorie KD, Newman SP. Clinical trial of the neuroprotectant clomethiazole in coronary artery bypass graft surgery: A randomized controlled trial. Anesthesiology. 2002;97:585-591	inappropriate data
Kozora E, Kongs S, Collins J, Hattler B, Baltz J, Hampton M, Grover F, Novitzky D, Shroyer A. Cognitive outcomes after on- versus off-pump coronary artery bypass surgery. Ann Thorac Surg. 2010;90:1134-1141	inappropriate data
Kumpaitiene B, Svagzdiene M, Drigotiene I, Sirvinskas E, Sepetiene R, Zakelis R, Benetis R. Correlation among decreased regional cerebral oxygen saturation, blood levels of brain injury biomarkers, and cognitive disorder. Journal of International Medical Research. 2018;46:3621-3629	inappropriate data
Kuzma E, Airdrie J, Littlejohns TJ, Lourida I, Thompson-Coon J, Lang IA, Scrobotovici M, Thacker EL, Fitzpatrick A, Kuller LH, et al. Coronary artery bypass graft surgery and dementia risk in the cardiovascular health study. Alzheimer disease and associated disorders. 2017:no pagination	inappropriate data
Lanctot KL, O'Regan J, Schwartz Y, Swardfager W, Saleem M, Oh PI, Herrmann N. Assessing cognitive effects of anticholinergic medications in patients with coronary artery disease. Psychosomatics. 2014;55:61-68	inappropriate data
LaPier TK. Functional status of patients during subacute recovery from coronary artery bypass surgery. Heart & lung : the journal of critical care. 2007;36:114-124	inappropriate data
Lazibat I, Sutlic Z, Brkic K, Nevajda B, Sikic J, Mestrovic AH. Predictors of short-term neurocognitive outcome following coronary revascularisation (cabg) depending on the use of cardiopulmonary bypass. Collegium Antropologicum. 2012;36:827-833	inappropriate data
Leacche M, Carrier M, Bouchard D, Pellerin M, Perrault LP, Paga P, Hebert Y, Cartier R. Improving neurologic outcome in off-pump surgery: The "no touch" technique. The heart surgery forum. 2003;6:169-175	inappropriate data
Lee JD, Lee SJ, Tsushima WT, Yamauchi H, Lau WT, Popper J, Stein A, Johnson D, Lee D, Petrovitch H, et al. Benefits of off-pump bypass on neurologic and clinical morbidity: A prospective randomized trial. The Annals of thoracic surgery. 2003;76:18-16	inappropriate data
Lewis M, Maruff P, Silbert B, Evered L, Scott D. The sensitivity and specificity of three common statistical rules for the classification of post-operative cognitive dysfunction following coronary artery bypass graft surgery. Acta Anaesthesiol Scand. 2006;50:50-57	inappropriate data
Lewis MS, Maruff P, Silbert BS, Evered LA, Scott DA. Detection of postoperative cognitive decline after coronary artery bypass graft surgery is affected by the number of neuropsychological tests in the assessment battery. The Annals of thoracic surgery. 2006;81:2097-2104	inappropriate data
Lewis MS, Maruff P, Silbert BS, Evered LA, Scott DA. The influence of different error estimates in the detection of postoperative cognitive dysfunction using reliable change indices with correction for practice effects (doi:10.1016/j.Acn.2006.05.004). Archives of Clinical Neuropsychology. 2007;22:249-257	inappropriate data
Lund C, Hol PK, Lundblad R, Fosse E, Sundet K, Tennoe B, Brucher R, Russell D. Comparison of cerebral embolization during off-pump and on-pump coronary artery bypass surgery. The Annals of thoracic surgery. 2003;76:765-770	inappropriate data
Lund C, Sundet K, Tennoe B, Hol PK, Rein KA, Fosse E, Russell D. Cerebral ischemic injury and cognitive impairment after off-pump and on-pump coronary artery bypass grafting surgery. The Annals of thoracic surgery. 2005;80:2126-2131	inappropriate data
Maekawa K, Goto T, Baba T, Yoshitake A, Katahira K, Yamamoto T. Impaired cognition preceding cardiac surgery is related to cerebral ischemic lesions. Journal of Anesthesia. 2011;25:330-336	inappropriate data

Mahanna EP, Blumenthal JA, White WD, Croughwell ND, Clancy CP, Smith LR, Newman MF. Defining neuropsychological dysfunction after coronary artery bypass grafting. The Annals of thoracic surgery. 1996;61:1342-1347	inappropriate data
Malheiros SMF, Brucki SMD, Gabbai AA, Bertolucci PHF, Juliano Y, Carvalho AC, Buffolo E. Neurological outcome in coronary artery surgery with and without cardiopulmonary bypass. Acta neurologica Scandinavica. 1995;92:256-260	inappropriate data
Martens S, Dietrich M, Herzog C, Doss M, Schneider G, Moritz A, Wimmer-Greinecker G. Automatic connector devices for proximal anastomoses do not decrease embolic debris compared with conventional anastomoses in cabg. European journal of cardio-thoracic surgery : official journal of the European Association for Cardio-thoracic Surgery. 2004;25:993-1000	inappropriate data
Mathew JP, Grocott HP, McCurdy JR, Ti LK, Davis RD, Laskowitz DT, Podgoreanu MV, Swaminathan M, Lynch J, Stafford-Smith M, et al. Preoperative statin therapy does not reduce cognitive dysfunction after cardiopulmonary bypass. Journal of cardiothoracic and vascular anesthesia. 2005;19:294-299	inappropriate data
Mathew JP, Grocott HP, Phillips-Bute B, Stafford-Smith M, Laskowitz DT, Rossignol D, Blumenthal JA, Newman MF, Bennett MJ, Booth JV, et al. Lower endotoxin immunity predicts increased cognitive dysfunction in elderly patients after cardiac surgery. Stroke. 2003;34:508-513	inappropriate data
Mattlar CE, Engblom E, Vesala P, Vanttinen E, Knuts LR. The proportion of patients with cognitive impairment after coronary artery bypass surgery: An 8-month follow-up study. Psychotherapy and psychosomatics. 1991;55:145-150	inappropriate data
McKenzie LH, Simpson J, Stewart M. The impact of depression on activities of daily living skills in individuals who have undergone coronary artery bypass graft surgery. Psychology, health & medicine. 2009;14:641-653	inappropriate data
McKhann GM, Borowicz LM, Goldsborough MA, Enger C, Selnes OA. Depression and cognitive decline after coronary artery bypass grafting. Lancet (London, England). 1997;349:1282-1284	inappropriate data
McKhann GM, Goldsborough MA, Borowicz Jr LM, Selnes OA, Mellits ED, Enger C, Quaskey SA, Baumgartner WA, Cameron DE, Stuart RS, et al. Cognitive outcome after coronary artery bypass: A one-year prospective study. Annals of Thoracic Surgery. 1997;63:510-515	inappropriate data
McKhann GM, Grega MA, Borowicz Jr LM, Bailey MM, Barry SJE, Zeger SL, Baumgartner WA, Selnes OA. Is there cognitive decline 1 year after cabg? Comparison with surgical and nonsurgical controls. Neurology. 2005;65:991-999	inappropriate data
Molstrom S, Nielsen TH, Andersen C, Nordstrom CH, Toft P. Bedside monitoring of cerebral energy state during cardiac surgery-a novel approach utilizing intravenous microdialysis. Journal of cardiothoracic and vascular anesthesia. 2016:no pagination	inappropriate data
Mongero LB, Beck JR, Manspeizer HE, Heyer EJ, Lee K, Spanier TA, Smith CR. Cardiac surgical patients exposed to heparin-bonded circuits develop less postoperative cerebral dysfunction than patients exposed to non-heparin-bonded circuits. Perfusion. 2001;16:107-111	inappropriate data
Motallebzadeh R, Bland JM, Markus HS, Kaski JC, Jahangiri M. Neurocognitive function and cerebral emboli: Randomized study of on-pump versus off-pump coronary artery bypass surgery. The Annals of thoracic surgery. 2007;83:475-482	inappropriate data
Mullges W, Babin-Ebell J, Reents W, Toyka KV. Cognitive performance after coronary artery bypass grafting: A follow-up study. Neurology. 2002;59:741-743	inappropriate data
Murkin J, Martzke J, Buchan A, Bentley C, Wong C. A randomized study of the influence of perfusion technique and ph management strategy in 316 patients undergoing coronary artery bypass surgery. Ii. Neurologic and cognitive outcomes. J Thorac Cardiovasc Surg. 1995;110:349-362	inappropriate data
Musumeci F, Feccia M, MacCarthy PA, Ellis GR, Mammana L, Brinn F, Penny WJ. Prospective randomized trial of single clamp technique versus intermittent ischaemic arrest: Myocardial and neurological outcome. European journal of cardio-thoracic surgery : official journal of the European Association for Cardio-thoracic Surgery. 1998;13:702-709	inappropriate data
Nathan HJ, Rodriguez R, Wozny D, Dupuis J-Y, Rubens FD, Bryson GL, Wells G. Neuroprotective effect of mild hypothermia in patients undergoing coronary artery surgery with cardiopulmonary bypass: Five-year follow-up of a randomized trial. The Journal of thoracic and cardiovascular surgery. 2007;133:1206-1211	inappropriate data
Newman MF, Kirchner JL, Phillips-Bute B, Gaver V, Grocott H, Jones RH, Mark DB, Reves JG, Blumenthal JA. Longitudinal assessment of neurocognitive function after coronary-artery bypass surgery. The New England journal of medicine. 2001;344:395-402	inappropriate data

Norkiene I, Samalavicius R, Misiuriene I, Paulauskiene K, Budrys V, Ivaskevicius J. Incidence and risk factors for early postoperative cognitive decline after coronary artery bypass grafting. Medicina (Kaunas, Lithuania). 2010;46:460-464	inappropriate data
Notzold A, Michel K, Khattab AA, Sievers HH, Huppe M. Diabetes mellitus increases adverse neurocognitive outcome after coronary artery bypass grafting surgery. The Thoracic and cardiovascular surgeon. 2006;54:307-312	inappropriate data
Oelofsen N, Fullard JPP, Foxcroft CD. Health-related quality of life after coronary revascularisation surgery. South African Journal of Psychology. 1998;28:85-91	inappropriate data
O'Neal JB, Billings FTt, Liu X, Shotwell MS, Liang Y, Shah AS, Ehrenfeld JM, Wanderer JP, Shaw AD. Risk factors for delirium after cardiac surgery: A historical cohort study outlining the influence of cardiopulmonary bypass. Canadian Journal of Anaesthesia. 2017;64:1129-1137	inappropriate data
Owens SG, Agnew J, Curbow B, Selnes O, Fitzgerald S, Ades AA-RBBBEGHHHHHMMMMNP-BRRRSSSSST. The association of neurocognitive decline and other variables with return to work, hobbies, and activities of daily living after coronary artery bypass graft surgery. Physical & Occupational Therapy in Geriatrics. 2010;28:348-359	inappropriate data
Ozturk S, Sacar M, Baltalarli A, Ozturk I. Effect of the type of cardiopulmonary bypass pump flow on postoperative cognitive function in patients undergoing isolated coronary artery surgery. Anatolian Journal of Cardiology. 2016;16:875-880	inappropriate data
Palotasa A, Reis HJ, Bogats G, Babik B, Racsmany M, Engvau L, Kecskemeti E, Juhasz A, Vieira LB, Teixeira AL, et al. Coronary artery bypass surgery provokes alzheimer's disease-like changes in the cerebrospinal fluid. Journal of Alzheimer's Disease. 2010;21:1153-1164	inappropriate data
Panagopoulou E, Maes S, Tyrodimos E, Benos A. Symptoms of traumatic stress after coronary artery bypass grafting. International journal of behavioral medicine. 2008;15:227-231	inappropriate data
Panday GFV, Fischer S, Bauer A, Metz D, Schubel J, El Shouki N, Eberle T, Hausmann H. Minimal extracorporeal circulation and off-pump compared to conventional cardiopulmonary bypass in coronary surgery. Interactive cardiovascular and thoracic surgery. 2009;9:832-836	inappropriate data
Paolin A, Michielon P, Betetto M, Sartori G, Valfre C, Rodriguez G, Murkin JM. Lower perfusion pressure during hypothermic cardiopulmonary bypass is associated with decreased cerebral blood flow and impaired memory performance 6 months postoperatively. The heart surgery forum. 2010;13:E7-12	inappropriate data
Patel RL, Turtle MR, Chambers DJ, James DN, Newman S, Venn GE. Alpha-stat acid-base regulation during cardiopulmonary bypass improves neuropsychologic outcome in patients undergoing coronary artery bypass grafting. The Journal of thoracic and cardiovascular surgery. 1996;111:1267-1279	inappropriate data
Patel RL, Turtle MR, Chambers DJ, Newman S, Venn GE. Hyperperfusion and cerebral dysfunction. Effect of differing acid-base management during cardiopulmonary bypass. European journal of cardio-thoracic surgery : official journal of the European Association for Cardio-thoracic Surgery. 1993;7:457-464	inappropriate data
Peterson JC, Pirraglia PA, Wells MT, Charlson ME. Attrition in longitudinal randomized controlled trials: Home visits make a difference. BMC Medical Research Methodology. 2012;12:178	inappropriate data
Petrovitch H, White L, Masaki KH, Ross GW, Abbott RD, Rodriguez BL, Lu G, Burchfiel CM, Blanchette PL, Curb JD. Influence of myocardial infarction, coronary artery bypass surgery, and stroke on cognitive impairment in late life. The American journal of cardiology. 1998;81:1017-1021	inappropriate data
Phillips-Bute B, Mathew JP, Blumenthal JA, Grocott HP, Laskowitz DT, Jones RH, Mark DB, Newman MF. Association of neurocognitive function and quality of life 1 year after coronary artery bypass graft (cabg) surgery. Psychosomatic medicine. 2006;68:369-375	inappropriate data
Potter GG, Plassman BL, Helms MJ, Steffens DC, Welsh-Bohmer KA. Age effects of coronary artery bypass graft on cognitive status change among elderly male twins. Neurology. 2004;63:2245-2249	inappropriate data
Puskas F, Grocott HP, White WD, Mathew JP, Newman MF, Bar-Yosef S. Intraoperative hyperglycemia and cognitive decline after cabg. The Annals of thoracic surgery. 2007;84:1467-1473	inappropriate data
Puskas JD, Stringer A, Hwang SN, Hatfield B, Smith AS, Kilgo PD, Williams WH. Neurocognitive and neuroanatomic changes after off-pump versus on-pump coronary artery bypass grafting: Long-term follow-up of a randomized trial. The Journal of thoracic and cardiovascular surgery. 2011;141:1116-1127	inappropriate data

Rasmussen LS, Christiansen M, Eliasen K, Sander-Jensen K, Moller JT. Biochemical markers for brain damage after cardiac surgery - time profile and correlation with cognitive dysfunction. Acta anaesthesiologica Scandinavica. 2002;46:547-551	inappropriate data
Rasmussen LS, Christiansen M, Hansen PB, Moller JT. Do blood levels of neuron-specific enolase and s-100 protein reflect cognitive dysfunction after coronary artery bypass? Acta anaesthesiologica Scandinavica. 1999;43:495-500	inappropriate data
Rasmussen LS, Sperling B, Abildstrom HH, Moller JT. Neuron loss after coronary artery bypass detected by spect estimation of benzodiazepine receptors. The Annals of thoracic surgery. 2002;74:1576-1580	inappropriate data
Raymond PD, Hinton-Bayre AD, Radel M, Ray MJ, Marsh NA. Assessment of statistical change criteria used to define significant change in neuropsychological test performance following cardiac surgery. European journal of cardio-thoracic surgery : official journal of the European Association for Cardio-thoracic Surgery. 2006;29:82-88	inappropriate data
Raymond PD, Radel M, Ray MJ, Hinton-Bayre AD, Marsh NA. Investigation of factors relating to neuropsychological change following cardiac surgery. Perfusion. 2007;22:27-33	inappropriate data
Reynolds JD, Amory DW, Grocott HP, White WD, Newman MF. Change in plasma glutamate concentration during cardiac surgery is a poor predictor of cognitive outcome. Journal of cardiothoracic and vascular anesthesia. 2002;16:431-436	inappropriate data
Robson MJA, Alston RP, Deary IJ, Andrews PJD, Souter MJ. Jugular bulb oxyhemoglobin desaturation, s100beta, and neurologic and cognitive outcomes after coronary artery surgery. Anesthesia and analgesia. 2001;93:839-845	inappropriate data
Ropacki SA, Bert AA, Ropacki MT, Rogers BL, Stern RA. The influence of cognitive reserve on neuropsychological functioning following coronary artery bypass grafting (cabg). Archives of clinical neuropsychology : the official journal of the National Academy of Neuropsychologists. 2007;22:73-85	inappropriate data
Rosengart TK, Sweet JJ, Finnin E, Wolfe P, Cashy J, Hahn E, Marymont J, Sanborn T. Stable cognition after coronary artery bypass grafting: Comparisons with percutaneous intervention and normal controls. The Annals of thoracic surgery. 2006;82:597-607	inappropriate data
Sader MA, Miller LA, Caine D, McCredie RJ, Corr MJ, Robertson M, Watson JDG, Celermajer DS. Neuropsychological and psychiatric outcomes following coronary surgery or angioplasty: A comparative study. Heart, Lung & Circulation. 2002;11:95-101	inappropriate data
Salmon P, Stanford SC, Mikhail G, Zielinski S, Pepper JR. Hemodynamic and emotional responses to a psychological stressor after cardiac transplantation. Psychosomatic medicine. 2001;63:289-299	inappropriate data
Santos FS, Velasco IT. Clinical features of elderly patients submitted to coronary artery bypass graft. Gerontology. 2005;51:234-241	inappropriate data
Sauer AMC, Nathoe HM, Hendrikse J, Peelen LM, Regieli J, Veldhuijzen DS, Kalkman CJ, Grobbee DE, Doevendans PA, Van Dijk D. Cognitive outcomes 7.5 years after angioplasty compared with off-pump coronary bypass surgery. Annals of Thoracic Surgery. 2013;96:1294-1300	inappropriate data
Schindler BA, Shook J, Schwartz GM. Beneficial effects of psychiatric intervention on recovery after coronary artery bypass graft surgery. General Hospital Psychiatry. 1989;11:358-364	inappropriate data
Schmitz C, Weinreich S, Schneider R, Schneider D, Speth I, Schulze-Rauschenbach C, Pohl C, Welz A. Off-pump versus on-pump coronary artery bypass: Can opcab reduce neurologic injury? The heart surgery forum. 2003;6:127-130	inappropriate data
Sellman M, Holm L, Ivert T, Semb BKH. A randomized study of neuropsychological function in patients undergoing coronary bypass surgery. Thoracic and Cardiovascular Surgeon. 1993;41:349-354	inappropriate data
Selnes OA, Goldsborough MA, Borowicz LM, Jr., Enger C, Quaskey SA, McKhann GM. Determinants of cognitive change after coronary artery bypass surgery: A multifactorial problem. The Annals of thoracic surgery. 1999;67:1669-1676	inappropriate data
Semrau JS, Scott SH, Hamilton AG, Petsikas D, Payne DM, Bisleri G, Saha T, Boyd JG. The relationship between cerebral oxygen saturation and quantitative metrics of neurological function after coronary bypass surgery: A feasibility study. Journal of Cardiovascular Surgery. 2018;59:716-728	inappropriate data
Sendelbach S, Lindquist R, Watanuki S, Savik K. Correlates of neurocognitive function of patients after off-pump coronary artery bypass surgery. American journal of critical care : an official publication, American Association of Critical-Care Nurses. 2006;15:290-298	inappropriate data

Shaaban-Ali M, Harmer M, Vaughan RS, Dunne JA, Latto IP, Haaverstad R, Kulatilake ENP, Butchart EG. Changes in serum s100beta protein and mini-mental state examination after cold (28degreec) and warm (34degreec) cardiopulmonary bypass using different blood gas strategies (alpha-stat and ph-stat). Acta anaesthesiologica Scandinavica. 2002;46:10-16	inappropriate data
Shaw PJ, Bates D, Cartlidge NE, French JM, Heaviside D, Julian DG, Shaw DA. Long-term intellectual dysfunction following coronary artery bypass graft surgery: A six month follow-up study. The Quarterly journal of medicine. 1987;62:259-268	inappropriate data
Shaw PJ, Bates D, Cartlidge NEF. Early intellectual dysfunction following coronary bypass surgery. Quarterly Journal of Medicine. 1986;58:59-68	inappropriate data
Shaw PJ, Bates D, Cartlidge NEF. Neurologic and neuropsychological morbidity following major surgery: Comparison of coronary artery bypass and peripheral vascular surgery. Stroke. 1987;18:700-707	inappropriate data
Shi Z, Song J, Chang H, Zhang Y. Effects of preoperative psychological intervention on early postoperative cognitive dysfunction after off-pump coronary artery bypass surgery. Biomedical Research (India). 2017;28:2909-2912	inappropriate data
Silbert B, Maruff P, Evered L, Scott D, Kalpokas M, Martin K, Lewis M, Myles P. Detection of cognitive decline after coronary surgery: A comparison of computerized and conventional tests. Br J Anaesth. 2004;92:814-820	inappropriate data
Silbert BS, Evered LA, Scott DA, Cowie TF. The apolipoprotein e epsilon{lunate}4 allele is not associated with cognitive dysfunction in cardiac surgery. Annals of Thoracic Surgery. 2008;86:841-847	inappropriate data
Silbert BS, Scott DA, Evered LA, Lewis MS, Maruff PT. Preexisting cognitive impairment in patients scheduled for elective coronary artery bypass graft surgery. Anesthesia and analgesia. 2007;104:1023-contents	inappropriate data
Silva FP, Schmidt AP, Valentin LS, Pinto KO, Zeferino SP, Oses JP, Wiener CD, Otsuki DA, Tort ABL, Portela LV, et al. S100b protein and neuron-specific enolase as predictors of cognitive dysfunction after coronary artery bypass graft surgery: A prospective observational study. European Journal of Anaesthesiology. 2016;33:681-689	inappropriate data
Sirvinskas E, Usas E, Mankute A, Raliene L, Jakuska P, Lenkutis T, Benetis R. Effects of intraoperative external head cooling on short-term cognitive function in patients after coronary artery bypass graft surgery. Perfusion. 2014;29:124-129	inappropriate data
Smith PL. The cerebral complications of coronary artery bypass surgery. Annals of the Royal College of Surgeons of England. 1988;70:212-216	inappropriate data
Song Z, Fu P, Chen M, Bi Q. Association of ct perfusion and postoperative cognitive dysfunction after off-pump coronary artery bypass grafting. Neurological Research. 2016;38:533-537	inappropriate data
Sousa Uva M, Cavaco S, Oliveira AG, Matias F, Silva C, Mesquita A, Aguiar P, Bau J, Pedro A, Magalhaes MP. Early graft patency after off-pump and on-pump coronary bypass surgery: A prospective randomized study. European Heart Journal. 2010;31:2492-2499	inappropriate data
Stanley TO, Mackensen GB, Grocott HP, White WD, Blumenthal JA, Laskowitz DT, Landolfo KP, Reves JG, Mathew JP, Newman MF. The impact of postoperative atrial fibrillation on neurocognitive outcome after coronary artery bypass graft surgery. Anesthesia and analgesia. 2002;94:290-295	inappropriate data
Steed L, Kong R, Stygall J, Acharya J, Bolla M, Harrison MJG, Humphries SE, Newman SP. The role of apolipoprotein e in cognitive decline after cardiac operation. Annals of Thoracic Surgery. 2001;71:823-826	inappropriate data
Stroobant N, Van Nooten G, Belleghem YV, Vingerhoets G. Short-term and long-term neurocognitive outcome in on-pump versus off-pump cabg. European Journal of Cardio-Thoracic Surgery. 2002;22:559-564	inappropriate data
Stroobant N, van Nooten G, De Bacquer D, Van Belleghem Y, Vingerhoets G. Neuropsychological functioning 3-5 years after coronary artery bypass grafting: Does the pump make a difference? European journal of cardio-thoracic surgery : official journal of the European Association for Cardio-thoracic Surgery. 2008;34:396-401	inappropriate data
Stroobant N, Van Nooten G, Van Belleghem Y, Vingerhoets G. Relation between neurocognitive impairment, embolic load, and cerebrovascular reactivity following on- and off-pump coronary artery bypass grafting. Chest. 2005;127:1967-1976	inappropriate data

Stroobant N, Vingerhoets G. Depression, anxiety, and neuropsychological performance in coronary artery bypass graft patients: A follow-up study. Psychosomatics. 2008;49:326-331	inappropriate data
Svenmarker S, Engström K, Karlsson T, Jansson E, Lindholm R, Aberg T. Influence of pericardial suction blood retransfusion on memory function and release of protein s100b. Perfusion. 2012;19:337-343	inappropriate data
Sweet JJ, Finnin E, Wolfe PL, Beaumont JL, Hahn E, Marymont J, Sanborn T, Rosengart TK. Absence of cognitive decline one year after coronary bypass surgery: Comparison to nonsurgical and healthy controls. The Annals of thoracic surgery. 2008;85:1571-1578	inappropriate data
Szwed K, Pawliszak W, Anisimowicz L, Bucinski A, Borkowska A. Short-term outcome of attention and executive functions from aorta no-touch and traditional off-pump coronary artery bypass surgery. The world journal of biological psychiatry : the official journal of the World Federation of Societies of Biological Psychiatry. 2014;15:397-403	inappropriate data
Tabatabaie O, Matin N, Heidari A, Tabatabaie A, Hadaegh A, Yazdanynejad S, Tabatabaie K. Spinal anesthesia reduces postoperative delirium in opium dependent patients undergoing coronary artery bypass grafting. Acta Anaesthesiologica Belgica. 2015;66:49-54	inappropriate data
Thomaidou E, Argiriadou H, Vretzakis G, Megari K, Taskos N, Chatzigeorgiou G, Anastasiadis K. Perioperative use of erythromycin reduces cognitive decline after coronary artery bypass grafting surgery: A pilot study. Clinical Neuropharmacology. 2017;40:195-200	inappropriate data
Thornton EW, Groom C, Fabri BM, Fox MA, Hallas C, Jackson M. Quality of life outcomes after coronary artery bypass graft surgery: Relationship to neuropsychologic deficit. The Journal of thoracic and cardiovascular surgery. 2005;130:1022-1027	inappropriate data
Toner I, Peden CJ, Hamid SK, Newman S, Taylor KM, Smith PLC. Magnetic resonance imaging and neuropsychological changes after coronary artery bypass graft surgery: Preliminary findings. Journal of neurosurgical anesthesiology. 1994;6:163-169	inappropriate data
Toner I, Taylor KM, Lockwood G, Newman S, Smith PLC. Eeg changes during cardiopulmonary bypass surgery and postoperative neuropsychological deficit: The effect of bubble and membrane oxygenators. European Journal of Cardio-Thoracic Surgery. 1997;11:312-319	inappropriate data
Toner I, Taylor KM, Newman S, Smith PLC. Cerebral functional changes following cardiac surgery: Neuropsychological and eeg assessment. European Journal of Cardio-Thoracic Surgery. 1998;13:13-20	inappropriate data
Tully PJ, Baker RA, Kneebone AC, Knight JL. Neuropsychologic and quality-of-life outcomes after coronary artery bypass surgery with and without cardiopulmonary bypass: A prospective randomized trial. Journal of cardiothoracic and vascular anesthesia. 2008;22:515-521	inappropriate data
Tully PJ, Baker RA, Knight JL, Turnbull DA, Winefield HR. Neuropsychological function 5 years after cardiac surgery and the effect of psychological distress. Archives of clinical neuropsychology : the official journal of the National Academy of Neuropsychologists. 2009;24:741-751	inappropriate data
van Dijk D, Diephuis J, Nierich A, Keizer A, Kalkman C. Beating heart versus conventional cardiopulmonary bypass: The octopus experience: A randomized comparison of 281 patients undergoing coronary artery bypass surgery with or without cardiopulmonary bypass. Semin Cardiothorac Vasc Anesth. 2006;10:167-170	inappropriate data
van Dijk D, Jansen E, Hijman R, Nierich A, Diephuis J, Moons K, Lahpor, Jr., Borst C, Keizer A, Nathoe H, et al. Cognitive outcome after off-pump and on-pump coronary artery bypass graft surgery: A randomized trial. JAMA. 2002;287:1405-1412	inappropriate data
van Dijk D, Moons KGM, Nathoe HM, van Aarnhem EHL, Borst C, Keizer AMA, Kalkman CJ, Hijman R, Octopus Study G. Cognitive outcomes five years after not undergoing coronary artery bypass graft surgery. The Annals of thoracic surgery. 2008;85:60-64	inappropriate data
van Dijk D, Spoor M, Hijman R, Nathoe H, Borst C, Jansen E, Grobbee D, Jaegere P, Kalkman C. Cognitive and cardiac outcomes 5 years after off-pump vs on-pump coronary artery bypass graft surgery. JAMA. 2007;297:701-708	inappropriate data
Vanninen R, Aikia M, Kononen M, Partanen K, Tulla H, Hartikainen P, Partanen J, Manninen H, Enberg P, Hippelainen M. Subclinical cerebral complications after coronary artery bypass grafting: Prospective analysis with magnetic resonance imaging, quantitative electroencephalography, and neuropsychological assessment. Archives of neurology. 1998;55:618-627	inappropriate data

Vedin J, Nyman H, Ericsson A, Hylander S, Vaage J. Cognitive function after on or off pump coronary artery bypass grafting. European journal of cardio-thoracic surgery : official journal of the European Association for Cardio-thoracic Surgery. 2006;30:305-310	inappropriate data
Venn GE, Patel RL, Chambers DJ. Cardiopulmonary bypass: Perioperative cerebral blood flow and postoperative cognitive deficit. The Annals of thoracic surgery. 1995;59:1331-1335	inappropriate data
Wahrborg P, Booth JE, Clayton T, Nugara F, Pepper J, Weintraub WS, Sigwart U, Stables RH, So S. Neuropsychological outcome after percutaneous coronary intervention or coronary artery bypass grafting: Results from the stent or surgery (sos) trial. Circulation. 2004;110:3411-3417	inappropriate data
Wang D, Wu X, Li J, Xiao F, Liu X, Meng M. The effect of lidocaine on early postoperative cognitive dysfunction after coronary artery bypass surgery. Anesthesia and analgesia. 2002;95:1134-contents	inappropriate data
Westaby S, Saatvedt K, White S, Katsumata T, Van Oeveren W, Bhatnagar NK, Brown S, Halligan PW. Is there a relationship between serum s-100beta protein and neuropsychologic dysfunction after cardiopulmonary bypass? Journal of Thoracic and Cardiovascular Surgery. 2000;119:132-137	inappropriate data
Whitaker D, Newman S, Stygall J, Hope-Wynne C, Harrison M, Walesby R. The effect of leucocyte-depleting arterial line filters on cerebral microemboli and neuropsychological outcome following coronary artery bypass surgery. Eur J Cardiothorac Surg. 2004;25:267-274	inappropriate data
Whitaker D, Stygall J, Harrison M, Newman S. Relationship between white cell count, neuropsychologic outcome, and microemboli in 161 patients undergoing coronary artery bypass surgery. The Journal of thoracic and cardiovascular surgery. 2006;131:1358-1363	inappropriate data
Whitaker D, Stygall J, Hope-Wynne C, Walesby R, Harrison M, Newman S. A prospective clinical study of cerebral microemboli and neuropsychological outcome comparing vent-line and auto-venting arterial line filters: Both filters are equally safe. Perfusion. 2006;21:83-86	inappropriate data
Whitaker DC, Green AJE, Stygall J, Harrison MJG, Newman SP. Evaluation of an alternative s100b assay for use in cardiac surgery: Relationship with microemboli and neuropsychological outcome. Perfusion. 2007;22:267-272	inappropriate data
Xu B, Qiao Q, Chen M, Rastogi R, Luo D, Bi Q. Relationship between neurological complications, cerebrovascular and cerebral perfusion following off-pump coronary artery bypass grafting. Neurological Research. 2015;37:421-426	inappropriate data
Yin Y-q, Luo A-l, Guo X-y, Li L-h, Huang Y-g. Postoperative neuropsychological change and its underlying mechanism in patients undergoing coronary artery bypass grafting. Chinese medical journal. 2007;120:1951-1957	inappropriate data
Yoon BW, Bae HJ, Kang DW, Lee SH, Hong KS, Kim KB, Park BJ, Roh JK. Intracranial cerebral artery disease as a risk factor for central nervous system complications of coronary artery bypass graft surgery. Stroke. 2001;32:94-99	inappropriate data
Zamvar V, Williams D, Hall J, Payne N, Cann C, Young K, Karthikeyan S, Dunne J. Assessment of neurocognitive impairment after off-pump and on-pump techniques for coronary artery bypass graft surgery: Prospective randomised controlled trial. BMJ (Clinical research ed.). 2002;325:1268	inappropriate data
Zhang W, Hu W, Shen M, Ye X, Huang Y, Sun Y. Profiles of delirium and the clinical outcomes of patients who underwent coronary artery bypass grafting: A prospective study from china. Journal of Clinical Nursing. 2016;25:631-641	inappropriate data
Zhang W, Sun Y, Liu Y, Qiu W, Ye X, Zhang G, Zhang L. A nursing protocol targeting risk factors for reducing postoperative delirium in patients following coronary artery bypass grafting: Results of a prospective before-after study. International Journal of Nursing Sciences. 2017;4:81-87	inappropriate data
Zimpfer D, Czerny M, Vogt F, Schuch P, Kramer L, Wolner E, Grimm M. Neurocognitive deficit following coronary artery bypass grafting: A prospective study of surgical patients and nonsurgical controls. The Annals of thoracic surgery. 2004;78:513-519	inappropriate data
Abrahamov D, Levran O, Naparstek S, Refaeli Y, Kaptson S, Abu Salah M, Ishai Y, Sahar G. Blood-brain barrier disruption after cardiopulmonary bypass: Diagnosis and correlation to cognition. Annals of Thoracic Surgery. 2017:no pagination	measure not operationalised
Abrahamov D, Levran O, Naparstek S, Refaeli Y, Kaptson S, Abu Salah M, Ishai Y, Sahar G. Blood-brain barrier disruption after cardiopulmonary bypass: Diagnosis and correlation to cognition. Annals of Thoracic Surgery. 2017;104:161-169	measure not operationalised

Abu-Omar Y, Cader S, Guerrieri Wolf L, Pigott D, Matthews PM, Taggart DP. Short-term changes in cerebral activity in on-pump and off-pump cardiac surgery defined by functional magnetic resonance imaging and their relationship to microembolization. The Journal of thoracic and cardiovascular surgery. 2006;132:1119-1125	measure not operationalised
Al Jawad MA, Taha S. Nadir oxygen delivery to the brain as a risk factor for post-operative neurocognitive impairment in patients undergoing coronary artery bypass grafting: A myth or fact. Journal of the Egyptian Society of Cardio-Thoracic Surgery. 2018;26:49-56	measure not operationalised
Al-Ruzzeh S, George S, Bustami M, Wray J, Ilsley C, Athanasiou T. Effect of off-pump coronary artery bypass surgery on clinical, angiographic, neurocognitive, and quality of life outcomes: Randomised controlled trial. BMJ. 2006;332:1365-1368	measure not operationalised
Andrell P, Jensen C, Norrsell H, Ekre O, Ekholm S, Norrsell U, Eliasson T, Mannheimer C, Blomstrand C. White matter disease in magnetic resonance imaging predicts cerebral complications after coronary artery bypass grafting. The Annals of thoracic surgery. 2005;79:74-80	measure not operationalised
Bak E, Marcisz C. Quality of life in elderly patients following coronary artery bypass grafting. Patient preference and adherence. 2014;8:289-299	measure not operationalised
Baufreton C, Allain P, Chevailler A, Etcharry-Bouyx F, Corbeau JJ, Legall D, de Brux JL. Brain injury and neuropsychological outcome after coronary artery surgery are affected by complement activation. The Annals of thoracic surgery. 2005;79:1597-1605	measure not operationalised
Bayram H, Hidiroglu M, Cetin L, Kucuker A, Iriz E, Uguz E, Saglam F, Sener E. Comparing s-100 beta protein levels and neurocognitive functions between patients undergoing on-pump and off-pump coronary artery bypass grafting. The Journal of surgical research. 2013;182:198-202	measure not operationalised
Bergh C, Backstrom M, Jonsson H, Havinder L, Johnsson P. In the eye of both patient and spouse: Memory is poor 1 to 2 years after coronary bypass and angioplasty. The Annals of thoracic surgery. 2002;74:689-694	measure not operationalised
Bernet F, Baykut D, Reineke D, Matt P, Zerkowski HR. Impact of female gender on the early outcome in off-pump coronary artery bypass surgery. European journal of medical research. 2006;11:114-118	measure not operationalised
Blumenthal JA, Madden DJ, Burker EJ, Croughwell N, Schniebolk S, Smith R, White WD, Hlatky M, Reves JG. A preliminary study of the effects of cardiac procedures on cognitive performance. International journal of psychosomatics : official publication of the International Psychosomatics Institute. 1991;38:13-16	measure not operationalised
Bokeriia LA, Golukhova EZ, Breskina NY, Polunina AG, Davydov DM, Begachev AV, Kazanovskaya SN. Asymmetric cerebral embolic load and postoperative cognitive dysfunction in cardiac surgery. Cerebrovascular diseases (Basel, Switzerland). 2007;23:50-56	measure not operationalised
Borde DP, Futane SS, Asegaonkar B, Apsingekar P, Khade S, Khodve B, Puranik M, George A, Joshi S. Effect of perioperative pregabalin on postoperative quality of recovery in patients undergoing off-pump coronary artery bypass grafting (opcabg): A prospective, randomized, double-blind trial. Journal of Cardiothoracic and Vascular Anesthesia. 2017;31:1241-1245	measure not operationalised
Breuer AC, Furlan AJ, Hanson MR, Lederman RJ, Loop FD, Cosgrove DM, Greenstreet RL, Estafanous FG. Central nervous system complications of coronary artery bypass graft surgery: Prospective analysis of 421 patients. Stroke. 1983;14:682-687	measure not operationalised
Brillman J, Davis D, Clark RE, Price TRP, Lovell MR, Benckart DA. Increased middle cerebral artery flow velocity during the initial phase of cardiopulmonary bypass may cause neurological dysfunction. Journal of Neuroimaging. 1995;5:135-141	measure not operationalised
Browne SM, Halligan PW, Wade DT, Taggart DP. Postoperative hypoxia is a contributory factor to cognitive impairment after cardiac surgery. The Journal of thoracic and cardiovascular surgery. 2003;126:1061-1064	measure not operationalised
Bruggemans EF, Van Dijk JG, Huysmans HA. Residual cognitive dysfunctioning at 6 months following coronary artery bypass graft surgery. European journal of cardio-thoracic surgery : official journal of the European Association for Cardio-thoracic Surgery. 1995;9:636-643	measure not operationalised
Bull NJ, Turner A, Levi C, Hunter M. Effect of core temperature and embolic load during cardiac surgery on motion perception. Heart, Lung & Circulation. 2016;25:512-519	measure not operationalised
Buschbeck D, Riess FC, Dobritzsch B, Dahme B, Bleese N. Short-term neuropsychologic differences after normothermic versus hypothermic cardiopulmonary bypass. The Journal of thoracic and cardiovascular surgery. 1998;116:350-353	measure not operationalised

Bute BP, Mathew J, Blumenthal JA, Welsh-Bohmer K, White WD, Mark D, Landolfo K, Newman MF, Neurological Outcome Research Group CAREIotDHCDNCUS, Allen ABBBCCCCDEEFF-SGHHHHHHJKKLMMMNN. Female gender is associated with impaired quality of life 1 year after coronary artery bypass surgery. Psychosomatic medicine. 2003;65:944-951	measure not operationalised
Camboni D, Schmidt S, Philipp A, Rupprecht L, Haneya A, Puehler T, Arlt M, Hilker M, Schmid C. Microbubble activity in miniaturized and in conventional extracorporeal circulation. ASAIO journal (American Society for Artificial Internal Organs : 1992). 2009;55:58-62	measure not operationalised
Can MG, Ulugol H, Gunes I, Aksu U, Tosun M, Karduz G, Vardar K, Toraman F. Effects of alprazolam and melatonin used for premedication on oxidative stress, glicocalyx integrity and neurocognitive functions. Turk Anestezi Ve Reanimasyon Dergisi. 2018;46:233-237	measure not operationalised
Cavalcante ES, Magario R, Conforti CA, Junior GC, Arena R, Carvalho ACC, Buffolo E, Filho BL. Impact of intensive physiotherapy on cognitive function after coronary artery bypass graft surgery. Arquivos Brasileiros de Cardiologia. 2014;103:391-397	measure not operationalised
Chang JC, Shipstone A, Llenado-Lee MA. Postoperative thrombotic thrombocytopenic purpura following cardiovascular surgeries. American journal of hematology. 1996;53:11-17	measure not operationalised
Charlson ME, Peterson JC, Krieger KH, Hartman GS, Hollenberg JP, Briggs WM, Segal AZ, Parikh M, Thomas SJ, Donahue RG, et al. Improvement of outcomes after coronary artery bypass ii: A randomized trial comparing intraoperative high versus customized mean arterial pressure. Journal of Cardiac Surgery. 2007;22:465-472	measure not operationalised
Cheng D, Newman M, Duke P, Wong D, Finegan B, Howie M, Fitch J, Bowdle T, Hogue C, Hillel Z, et al. The efficacy and resource utilization of remifentanil and fentanyl in fast-track coronary artery bypass graft surgery: A prospective randomized, double-blinded controlled, multi-center trial. Anesth Analg. 2001;92:1094-1102	measure not operationalised
Clark RE, Brillman J, Davis DA, Lovell MR, Price TR, Magovern GJ. Microemboli during coronary artery bypass grafting. Genesis and effect on outcome. The Journal of thoracic and cardiovascular surgery. 1995;109:249-248	measure not operationalised
Clough RA, Leavitt BJ, Morton JR, Plume SK, Hernandez F, Nugent W, Lahey SJ, Ross CS, O'Connor GT. The effect of comorbid illness on mortality outcomes in cardiac surgery. Archives of Surgery. 2002;137:428-433	measure not operationalised
Cox ML, Gulack BC, Thibault DP, He X, Williams ML, Thourani VH, Jacobs JP, Brennan JM, Daneshmand MA, Acharya D. Outcomes after coronary artery bypass grafting in patients with myocardial infarction, cardiogenic shock and unresponsive neurological state: Analysis of the society of thoracic surgeons database. European Journal of Cardio-Thoracic Surgery. 2018;54:710-716	measure not operationalised
Das S, Nanda SK, Bisoi AK, Wadhawan AN. Effect of preoperative statin therapy on early postoperative memory impairment after off-pump coronary artery bypass surgery. Annals of Cardiac Anaesthesia. 2016;19:38-44	measure not operationalised
de Queiroz Guimaraes MNM, de Mello Almada Filho C. Functional status change in older adults undergoing coronary artery bypass surgery. Sao Paulo Medical Journal. 2011;129:99-106	measure not operationalised
Delphin E, Jackson D, Gubenko Y, Botea A, Esrig B, Fritz W, Mavridis S. Sevoflurane provides earlier tracheal extubation and assessment of cognitive recovery than isoflurane in patients undergoing off-pump coronary artery bypass surgery. J Cardiothorac Vasc Anesth. 2007;21:690-695	measure not operationalised
Demir G, Cukurova Z, Eren G, Hergunsel O. Comparison of the effects of on-pump and off-pump coronary artery bypass surgery on cerebral oxygen saturation using near-infrared spectroscopy. Korean Journal of Anesthesiology. 2014;67:391-397	measure not operationalised
Diegeler A, Hirsch R, Schneider F, Schilling LO, Falk V, Rauch T, Mohr FW. Neuromonitoring and neurocognitive outcome in off-pump versus conventional coronary bypass operation. The Annals of thoracic surgery. 2000;69:1162-1166	measure not operationalised
Dogan S, Graubitz K, Aybek T, Khan MF, Kessler P, Moritz A, Wimmer-Greinecker G. How safe is the port access technique in minimally invasive coronary artery bypass grafting? The Annals of thoracic surgery. 2002;74:1537-1543	measure not operationalised
Doganci S, Gunaydin S, Kocak OM, Yilmaz S, Demirkilic U. Impact of the intensity of microemboli on neurocognitive outcome following cardiopulmonary bypass. Perfusion. 2013;28:256-262	measure not operationalised

Doraiswamy PM, Babyak MA, Hennig T, Trivedi R, White WD, Mathew JP, Newman MF, Blumenthal JA. Donepezil for cognitive decline following coronary artery bypass surgery: A pilot randomized controlled trial. Psychopharmacology bulletin. 2007;40:54-62	measure not operationalised
Dupuis G, Kennedy E, Lindquist R, Barton FB, Terrin ML, Hoogwerf BJ, Czajkowski SM, Herd JA, Post CBSI, Ayanian BBBBBCDGGHHKKKLLLLMMNNNNNPRRSS. Coronary artery bypass graft surgery and cognitive performance. American Journal of Critical Care. 2006;15:471-478	measure not operationalised
Engelhardt W, Dierks T, Pause M, Sold M, Hartung E, Silber R. P300-mapping--a neurophysiological tool to quantify cerebral dysfunction after coronary artery bypass grafting. European journal of cardio-thoracic surgery : official journal of the European Association for Cardio-thoracic Surgery. 1995;9:12-17	measure not operationalised
Engelman RM, Pleet AB, Rousou JA, Flack JE, 3rd, Deaton DW, Pekow PS, Gregory CA. Influence of cardiopulmonary bypass perfusion temperature on neurologic and hematologic function after coronary artery bypass grafting. The Annals of thoracic surgery. 1999;67:1547-1556	measure not operationalised
Eremina DA, Shchelkova OY. The dynamics of the cognitive functioning and emotional state of cardiac patients during rehabilitation after coronary revascularization. Psychology in Russia: State of the Art. 2017;10:201-214	measure not operationalised
Erol DD, Ibis HA. Glutamate/aspartate supplementation during cardiopulmonary bypass: Effect on postoperative neurocognitive function. The heart surgery forum. 2008;11:E17-20	measure not operationalised
Farhoudi M, Parvizi R, Bilehjani E, Tarzamni MK, Mehrvar K, Safaiyan A. Preoperative transcranial and carotid doppler study in coronary artery bypass graft patients. Neurosciences (Riyadh, Saudi Arabia). 2007;12:42-45	measure not operationalised
Fearn SJ, Pole R, Wesnes K, Faragher EB, Hooper TL, McCollum CN. Cerebral injury during cardiopulmonary bypass: Emboli impair memory. The Journal of thoracic and cardiovascular surgery. 2001;121:1150-1160	measure not operationalised
Finkelmeier BA, Kaye GM, Saba YS, Parker MA. Influence of age on postoperative course in coronary artery bypass patients. The Journal of cardiovascular nursing. 1993;7:38-46	measure not operationalised
Fish KJ, Helms KN, Sarnquist FH, van Steennis C, Linet OI, Hilberman M, Mitchell RS, Jamieson SW, Miller DC, Tinklenberg JS. A prospective, randomized study of the effects of prostacyclin on neuropsychologic dysfunction after coronary artery operation. The Journal of thoracic and cardiovascular surgery. 1987;93:609-615	measure not operationalised
Flesch M, Knipp S, Kessler G, Geissler H-J, Massoudy P, Wilhelm H, Philipp T, Erdmann E. Arta: At1-receptor blocker therapy in patients undergoing coronary artery bypass grafting. Clinical research in cardiology : official journal of the German Cardiac Society. 2009;98:33-43	measure not operationalised
Folks DG, Franceschini J, Sokol RS, Freeman 3rd AM, Folks DM. Coronary artery bypass surgery in older patients: Psychiatric morbidity. Southern medical journal. 1986;79:303-306	measure not operationalised
Freeman AM, 3rd, Folks DG, Sokol RS, Govier AV, Reves JG, Fleece EL, Hall KR, Zorn GL, Karp RB. Cognitive function after coronary bypass surgery: Effect of decreased cerebral blood flow. The American journal of psychiatry. 1985;142:110-112	measure not operationalised
Freundt M, Ried M, Philipp A, Diez C, Kolat P, Hirt SW, Schmid C, Haneya A. Minimized extracorporeal circulation is improving outcome of coronary artery bypass surgery in the elderly. Perfusion. 2016;31:143-148	measure not operationalised
Gasparovic H, Malojcic B, Borojevic M, Vojkovic J, Gabelica R, Milicic D, Biocina B. Reduction of microembolic signals with a single-clamp strategy in coronary artery bypass surgery: A pilot study. The heart surgery forum. 2009;12:E357-361	measure not operationalised
Gerriets T, Schwarz N, Sammer G, Baehr J, Stolz E, Kaps M, Kloevekorn W, Bachmann G, Schönburg M. Protecting the brain from gaseous and solid micro-emboli during coronary artery bypass grafting: A randomized controlled trial. Eur Heart J. 2010;31:360-368	measure not operationalised
Geyik S, Yigiter R, Akcali A, Deniz H, Murat Geyik A, Ali Elci M, Hafiz E. The effect of circadian melatonin levels on inflammation and neurocognitive functions following coronary bypass surgery. Annals of thoracic and cardiovascular surgery : official journal of the Association of Thoracic and Cardiovascular Surgeons of Asia. 2015;21:466-473	measure not operationalised

Ghafari R, Baradari A, Firouzian A, Nouraei M, Aarabi M, Zamani A, Emami ZA. Cognitive deficit in first-time coronary artery bypass graft patients: A randomized clinical trial of lidocaine versus procaine hydrochloride. Perfusion. 2012;27:320-325	measure not operationalised
Gokgoz L, Gunaydin S, Sinci V, Unlu M, Boratav C, Babacan A, Soncul H, Halit V, Inanir S, Ersoz A. Psychiatric complications of cardiac surgery postoperative delirium syndrome. Scandinavian Cardiovascular Journal. 1997;31:217-222	measure not operationalised
Gong Z, Li J, Zhong Y, Guan X, Huang A, Ma L. Effects of dexmedetomidine on postoperative cognitive function in patients undergoing coronary artery bypass grafting. Experimental and Therapeutic Medicine. 2018;16:4685-4689	measure not operationalised
Gonzalez-Scarano F, Hurtig HI. Neurologic complications of coronary artery bypass grafting: Case-control study. Neurology. 1981;31:1032-1035	measure not operationalised
Gottesman RF, Hillis AE, Grega MA, Borowicz Jr LM, Selnes OA, Baumgartner WA, McKhann GM. Early postoperative cognitive dysfunction and blood pressure during coronary artery bypass graft operation. Archives of neurology. 2007;64:1111-1114	measure not operationalised
Grega MA, Borowicz LM, Baumgartner WA. Impact of single clamp versus double clamp technique on neurologic outcome. The Annals of thoracic surgery. 2003;75:1387-1391	measure not operationalised
Grothusen C, Friedrich C, Attmann T, Meinert J, Ohnewald E, Ulbricht U, Huenges K, Haneya A, Frank D, Graesner JT, et al. Coronary artery bypass surgery within 48 hours after cardiac arrest due to acute myocardial infarction. European Journal of Cardio-Thoracic Surgery. 2017;52:297-302	measure not operationalised
Habib S, Khan AR, Iqbal Afridi M, Saeed A, Jan AF, Amjad N. Frequency and predictors of cognitive decline in patients undergoing coronary artery bypass graft surgery. Journal of the College of Physicians and Surgeons Pakistan. 2014;24:543-548	measure not operationalised
Halici U, Acipayam M, Uncu H, Altinay L, Ketenciler S, Ozsoyler I. A retrospective comparison of early results of isolated coronary artery bypass surgery in young (<40 years) and middle aged (40-60 years) patients. Acta Medica Mediterranea 2015;31:807-811	measure not operationalised
Halkos ME, Anderson A, Binongo JNG, Stringer A, Lasanajak Y, Thourani VH, Lattouf OM, Guyton RA, Baio KT, Sarin E, et al. Operative strategies to reduce cerebral embolic events during on- and off-pump coronary artery bypass surgery: A stratified, prospective randomized trial. Journal of Thoracic and Cardiovascular Surgery. 2017;154:1278-1285.e1271	measure not operationalised
Hassani S, Alipour A, Darvishi Khezri H, Firouzian A, Emami Zeydi A, Gholipour Baradari A, Ghafari R, Habibi W-A, Tahmasebi H, Alipour F, et al. Can valeriana officinalis root extract prevent early postoperative cognitive dysfunction after cabg surgery? A randomized, double-blind, placebo-controlled trial. Psychopharmacology. 2015;232:843-850	measure not operationalised
Hempe S, Moza A, Goetzenich A, Tewarie L, Bleilevens C, Autschbach R, Schnoering H, Zayat R. Synchronous or staged carotid endarterectomy and coronary artery bypass grafting? Propensity score matched study. Heart Surgery Forum. 2018;21:E359-E364	measure not operationalised
Holinski S, Claus B, Alaaraj N, Dohmen PM, Kirilova K, Neumann K, Uebelhack R, Konertz W. Cerebroprotective effect of piracetam in patients undergoing coronary bypass surgery. Medical Science Monitor. 2008;14:PI53-PI57	measure not operationalised
Holinski S, Claus B, Barajas T, Neumann K, Uebelhack R, Konertz W. Cerebroprotective effect of preoperative dual antiplatelet therapy in patients undergoing coronary bypass surgery. Annals of thoracic and cardiovascular surgery : official journal of the Association of Thoracic and Cardiovascular Surgeons of Asia. 2014;20:38-43	measure not operationalised
Holinski S, Claus B, Haeger N, Neumann K, Uebelhack R, Konertz W. Effect of different pump heads for cpb on early cognitive outcome after coronary artery bypass surgery. Annals of thoracic and cardiovascular surgery : official journal of the Association of Thoracic and Cardiovascular Surgeons of Asia. 2013;19:273-278	measure not operationalised
Issitt RW, Harvey I, Walsh B, Voegeli D. Quantification of lipid filtration and the effects on cerebral injury during cardiopulmonary bypass. Annals of Thoracic Surgery. 2017;104:884-890	measure not operationalised
Ji F, Li Z, Young N, Moore P, Liu H. Perioperative dexmedetomidine improves mortality in patients undergoing coronary artery bypass surgery. Journal of cardiothoracic and vascular anesthesia. 2014;28:267-273	measure not operationalised

Jonsson H, Johnsson P, Alling C, Westaby S, Blomquist S. Significance of serum s100 release after coronary artery bypass grafting. The Annals of thoracic surgery. 1998;65:1639-1644	measure not operationalised
Joudi M, Fathi M, Harati H, Joudi M, Izanloo A, Rahdari A, Soltani G. Evaluating the incidence of cognitive disorder following off-pump coronary artery bypasses surgery and its predisposing factors. Anesthesiology and pain medicine. 2014;4:e18545	measure not operationalised
Kadoi Y, Goto F. Factors associated with postoperative cognitive dysfunction in patients undergoing cardiac surgery. Surgery today. 2006;36:1053-1057	measure not operationalised
Kanbak M, Saricaoglu F, Akinci SB, Oc B, Balci H, Celebioglu B, Aypar U. The effects of isoflurane, sevoflurane, and desflurane anesthesia on neurocognitive outcome after cardiac surgery: A pilot study. The heart surgery forum. 2007;10:E36-41	measure not operationalised
Kara I, Erkin A, Sacli H, Demirtas M, Percin B, Diler MS, Kirali K. The effects of near-infrared spectroscopy on the neurocognitive functions in the patients undergoing coronary artery bypass grafting with asymptomatic carotid artery disease: A randomized prospective study. Annals of thoracic and cardiovascular surgery : official journal of the Association of Thoracic and Cardiovascular Surgeons of Asia. 2015;21:544-550	measure not operationalised
Kawahara F, Kadoi Y, Saito S, Goto F, Fujita N. Slow rewarming improves jugular venous oxygen saturation during rewarming. Acta anaesthesiologica Scandinavica. 2003;47:419-424	measure not operationalised
Keizer AMA, Hijman R, van Dijk D, Kalkman CJ, Kahn RS. Cognitive self-assessment one year after on-pump and off-pump coronary artery bypass grafting. The Annals of thoracic surgery. 2003;75:835-839	measure not operationalised
Khosravi A, Skrabal CA, Westphal B, Kundt G, Greim B, Kunesch E, Liebold A, Steinhoff G. Evaluation of coated oxygenators in cardiopulmonary bypass systems and their impact on neurocognitive function. Perfusion. 2005;20:249-254	measure not operationalised
Kilo J, Czerny M, Gorlitzer M, Zimpfer D, Baumer H, Wolner E, Grimm M. Cardiopulmonary bypass affects cognitive brain function after coronary artery bypass grafting. The Annals of thoracic surgery. 2001;72:1926-1932	measure not operationalised
Klonoff H, Clark C, Kavanagh-Gray D, Mizgala H, Munro I. Two-year follow-up study of coronary bypass surgery. Psychologic status, employment status, and quality of life. The Journal of thoracic and cardiovascular surgery. 1989;97:78-85	measure not operationalised
Klonoff H, Fleetham J, Tayor R, Clark C. Treatment outcome of obstructive sleep apnea: Physiological and neuropsychological concomitants. Journal of Nervous and Mental Disease. 1987;175:208-212	measure not operationalised
Knapik P, Ciesla D, Wawrzynczyk M, Knapik M, Borkowski J, Zembala M. Incidence and prediction of permanent neurological deficits after cardiac surgery - are the existing models of prediction truly global? European journal of cardio-thoracic surgery : official journal of the European Association for Cardio-thoracic Surgery. 2010;37:717-723	measure not operationalised
Knipp SC, Matatko N, Wilhelm H, Schlamann M, Massoudy P, Forsting M, Diener HC, Jakob H. Evaluation of brain injury after coronary artery bypass grafting. A prospective study using neuropsychological assessment and diffusion-weighted magnetic resonance imaging. European journal of cardio-thoracic surgery : official journal of the European Association for Cardio-thoracic Surgery. 2004;25:791-800	measure not operationalised
Kobayashi T, Hamano K, Mikamo A, Okada H, Gohra H, Miyamoto M, Oda T, Esato K. Perioperative features of coronary artery bypass grafting in patients aged 75 years or older. The Japanese journal of thoracic and cardiovascular surgery : official publication of the Japanese Association for Thoracic Surgery = Nihon Kyobu Geka Gakkai zasshi. 2002;50:152-157	measure not operationalised
Koenig MA, Grega MA, Bailey MM, Pham LD, Zeger SL, Baumgartner WA, McKhann GM. Statin use and neurologic morbidity after coronary artery bypass grafting: A cohort study. Neurology. 2009;73:2099-2106	measure not operationalised
Kopp M, Knuenz A, Oberascher F, Pfaffenberger N, Lucic N, Ruedl G, Holzner B, Bonaros N, Johannes B. Psychomotor car-driving abilities after robotically assisted totally endoscopic coronary artery bypass grafting. The Thoracic and cardiovascular surgeon. 2012;60:462-467	measure not operationalised
Kornfeld DS, Heller SS, Frank KA, Edie RN, Barsa J. Delirium after coronary artery bypass surgery. The Journal of thoracic and cardiovascular surgery. 1978;76:93-96	measure not operationalised

Kotfis K, Szylinska A, Listewnik M, Lechowicz K, Kosiorowska M, Drozdzal S, Brykczynski M, Rotter I, Zukowski M. Balancing intubation time with postoperative risk in cardiac surgery patients - a retrospective cohort analysis. Therapeutics & Clinical Risk Management. 2018;14:2203-2212	measure not operationalised
Krannich J-H, Tobias T, Broscheit J, Leyh R, Mullges W. Diabetes severely affects attentional performance after coronary artery bypass grafting. Journal Of Cardiothoracic Surgery. 2012;7:115	measure not operationalised
Krzych LJ, Szurlej D, Kooldziej T, Machej L, Weglarzy A, Balch A, Wilczynski M, Wos S, Bochenek A. Diagnostic accuracy of pre-operative nt-probnp level in predicting short-term outcomes in coronary surgery: A pilot study. Kardiologia Polska. 2011;69:1121-1127	measure not operationalised
Kunihara T, Tscholl D, Langer F, Heinz G, Sata F, Schafers HJ. Cognitive brain function after hypothermic circulatory arrest assessed by cognitive p300 evoked potentials. European Journal of Cardio-Thoracic Surgery. 2007;32:507-513	measure not operationalised
Kuzma E, Airdrie J, Littlejohns TJ, Lourida I, Thompson-Coon J, Lang IA, Scrobotovici M, Thacker EL, Fitzpatrick A, Kuller LH, et al. Coronary artery bypass graft surgery and dementia risk in the cardiovascular health study. Alzheimer Disease & Associated Disorders. 2017;31:120-127	measure not operationalised
Lamy A, Devereaux PJ, Prabhakaran D, Taggart DP, Hu S, Paolasso E, Straka Z, Piegas LS, Akar AR, Jain AR, et al. Effects of off-pump and on-pump coronary-artery bypass grafting at 1 year. New England Journal of Medicine. 2013;368:1179-1188	measure not operationalised
Lee TA, Wolozin B, Weiss KB, Bednar MM. Assessment of the emergence of alzheimer's disease following coronary artery bypass graft surgery or percutaneous transluminal coronary angioplasty. Journal of Alzheimer's disease : JAD. 2005;7:319-324	measure not operationalised
Lelis RGB, Krieger JE, Pereira AC, Schmidt AP, Carmona MJ, Oliveira SA, Auler JOC, Jr. Apolipoprotein e4 genotype increases the risk of postoperative cognitive dysfunction in patients undergoing coronary artery bypass graft surgery. The Journal of cardiovascular surgery. 2006;47:451-456	measure not operationalised
Lewis M, Maruff P, Silbert B. Examination of the use of cognitive domains in postoperative cognitive dysfunction after coronary artery bypass graft surgery. Ann Thorac Surg. 2005;80:910-916	measure not operationalised
Loberman D, Consalvi C, Healey A, Rivera B, Poulin K, Mohr R, Ziv-Baran T. Adverse cerebral outcomes after coronary artery bypass surgery-more than a decade of experience in a single center. Thoracic and Cardiovascular Surgeon. 2018;66:452-456	measure not operationalised
Loladze G, Kuehnel RU, Claus T, Hartrumpf M, Kuepper F, Pohl M, Albes JM. Double-wire versus single-wire sternal closure in obese patients: A randomized prospective study. Thoracic & Cardiovascular Surgeon. 2017;65:332-337	measure not operationalised
Luo D, Yip J, Song Z, Xu B, Bi Q. Ct perfusion in predicting the morbidity and prognosis of hypoxic-ischemic encephalopathy after off-pump coronary artery bypass grafting. Neurological Research. 2017;39:521-529	measure not operationalised
Lyketsos CG, Toone L, Tschanz J, Corcoran C, Norton M, Zandi P, Munger R, Breitner JCS, Welsh-Bohmer K, Cache County Study G, et al. A population-based study of the association between coronary artery bypass graft surgery (cabg) and cognitive decline: The cache county study. International Journal of Geriatric Psychiatry. 2006;21:509-518	measure not operationalised
Martin TD, Craver JM, Gott JP, Weintraub WS, Ramsay J, Mora CT, Guyton RA, Weisel RD, Engelman RM, Vaughn CC, et al. Prospective, randomized trial of retrograde warm blood cardioplegia: Myocardial benefit and neurologic threat. Annals of Thoracic Surgery. 1994;57:298-304	measure not operationalised
Martinovic I, Lindemann S, Irqsusi M, Mirat J, Vcev A, Wittlinger T, Noutsias M. Minimally invasive direct coronary bypass surgery via distal mini-sternotomy : Promising clinical results with anaortic, multivessel, all-arterial technique. Herz. 2018;10:10	measure not operationalised
Mathisen L, Lingaas PS, Andersen MH, Hol PK, Fredriksen PM, Sundet K, Rokne B, Wahl AK, Fosse E. Changes in cardiac and cognitive function and self-reported outcomes at one year after coronary artery bypass grafting. The Journal of thoracic and cardiovascular surgery. 2010;140:122-128	measure not operationalised
McKhann GM, Grega MA, Borowicz Jr LM, Bechamps M, Selnes OA, Baumgartner WA, Royall RM. Encephalopathy and stroke after coronary artery bypass grafting: Incidence, consequences, and prediction. Archives of neurology. 2002;59:1422-1428	measure not operationalised
McLean RF, Wong BI, Naylor CD, Snow WG, Harrington EM, Gawel M, Fremes SE. Cardiopulmonary bypass, temperature, and central nervous system dysfunction. Circulation. 1994;90:II250-255	measure not operationalised

Merwin SL, Abram HS. Psychologic response to coronary artery bypass. Southern medical journal. 1977;70:153-155	measure not operationalised
Mesgerani M, Begum K. Body mass index (bmi) as a factor influencing post surgical events in patients with coronary artery bypass grafting (cabg). JPMI - Journal of Postgraduate Medical Institute. 2009;23:296-303	measure not operationalised
Millar K, Asbury AJ, Murray GD. Pre-existing cognitive impairment as a factor influencing outcome after cardiac surgery. British Journal of Anaesthesia. 2001;86:63-67	measure not operationalised
Miyairi T, Takamoto S, Kotsuka Y, Takeuchi A, Yamanaka K, Sato H. Comparison of neurocognitive results after coronary artery bypass grafting and thoracic aortic surgery using retrograde cerebral perfusion. European journal of cardio-thoracic surgery : official journal of the European Association for Cardio-thoracic Surgery. 2005;28:97-93	measure not operationalised
Moazen-Zadeh E, Abbasi SH, Safi-Aghdam H, Shahmansouri N, Arjmandi-Beglar A, Hajhosseinn Talasaz A, Salehiomran A, Forghani S, Akhondzadeh S. Effects of saffron on cognition, anxiety, and depression in patients undergoing coronary artery bypass grafting: A randomized double-blind placebo-controlled trial. Journal of Alternative & Complementary Medicine. 2018;24:361-368	measure not operationalised
Morino Y, Hara K, Tanabe K, Kuroda Y, Ayabe S, Kozuma K, Kigawa I, Fukuda S, Wanibuchi Y, Tamura T. Retrospective analysis of cerebral complications after coronary artery bypass grafting in elderly patients. Japanese circulation journal. 2000;64:46-50	measure not operationalised
Mullges W, Berg D, Schmidtke A, Weinacker B, Toyka KV. Early natural course of transient encephalopathy after coronary artery bypass grafting. Critical care medicine. 2000;28:1808-1811	measure not operationalised
Mullges W, Franke D, Reents W, Babin-Ebell J, Toyka KV. Reduced rate of microembolism by optimized aortic cannula position does not influence early postoperative cognitive performance in cabg patients. Cerebrovascular diseases (Basel, Switzerland). 2003;15:192-198	measure not operationalised
Mutch WAC, Fransoo RR, Campbell BI, Chateau DG, Sirski M, Warrian RK. Dementia and depression with ischemic heart disease: A population-based longitudinal study comparing interventional approaches to medical management. PloS one. 2011;6:e17457	measure not operationalised
Newman S, Klinger L, Venn G, Smith P, Harrison M, Treasure T. Subjective reports of cognition in relation to assessed cognitive performance following coronary artery bypass surgery. Journal of Psychosomatic Research. 1989;33:227-233	measure not operationalised
Nina VJdS, Rocha MIdA, Rodrigues RF, Oliveira VCd, Teixeira JLL, Figueredo ED, Nina RVdAH, Sousa CAC. Assesment of cabdeal score as predictor of neurological dysfunction after on-pump coronary artery bypass grafting surgery. Revista brasileira de cirurgia cardiovascular : orgao oficial da Sociedade Brasileira de Cirurgia Cardiovascular. 2012;27:429-435	measure not operationalised
Pacini D, Di Marco L, Leone A, Tonon C, Pettinato C, Fonti C, Manners DN, Di Bartolomeo R. Cerebral functions and metabolism after antegrade selective cerebral perfusion in aortic arch surgery. European journal of cardio-thoracic surgery : official journal of the European Association for Cardio-thoracic Surgery. 2010;37:1322-1331	measure not operationalised
Palanzo DA, Zarro DL. The effect of fast-tracking on neurological complications post-cardiopulmonary bypass. Perfusion. 1996;11:451-453	measure not operationalised
Park YJ, Yoon JW, Kim KI, Lee YJ, Kim KW, Choi SH, Lim S, Choi DJ, Park K-H, Choh JH, et al. Subclinical hypothyroidism might increase the risk of transient atrial fibrillation after coronary artery bypass grafting. The Annals of thoracic surgery. 2009;87:1846-1852	measure not operationalised
Prapas SN, Panagiotopoulos IA, Pentchev DN, Ayyad MAS, Protogeros DA, Kotsis VN, Linardakis IN, Tzanavaras TP, Stratigi PT. Aorta no-touch off-pump coronary artery revascularization in octogenarians: 5 years' experience. The heart surgery forum. 2009;12:E349-353	measure not operationalised
Puehler T, Haneya A, Philipp A, Zausig YA, Kobuch R, Diez C, Birnbaum DE, Schmid C. Minimized extracorporeal circulation system in coronary artery bypass surgery: A 10-year single-center experience with 2243 patients. European journal of cardio-thoracic surgery : official journal of the European Association for Cardio-thoracic Surgery. 2011;39:459-464	measure not operationalised

Rankin KP, Kochamba GS, Boone KB, Petitti DB, Buckwalter JG. Presurgical cognitive deficits in patients receiving coronary artery bypass graft surgery. Journal of the International Neuropsychological Society : JINS. 2003;9:913-924	measure not operationalised
Reineke D, Winkler B, Konig T, Meszaros K, Sodeck G, Schonhoff F, Erdoes G, Czerny M, Carrel T. Minimized extracorporeal circulation does not impair cognitive brain function after coronary artery bypass grafting. Interactive cardiovascular and thoracic surgery. 2015;20:68-73	measure not operationalised
Ried M, Kobuch R, Rupprecht L, Keyser A, Hilker M, Schmid C, Diez C. Reduced 30-day mortality in men after elective coronary artery bypass surgery with minimized extracorporeal circulation-a propensity score analysis. BMC Cardiovascular Disorders. 2012;12:17	measure not operationalised
Roach GW, Kanchuger M, Mangano CM, Newman M, Nussmeier N, Wolman R, Aggarwal A, Marschall K, Graham SH, Ley C, et al. Adverse cerebral outcomes after coronary bypass surgery. The New England journal of medicine. 1996;335:1857-1863	measure not operationalised
Sabban MA, Jalal A, Bakir BM, Alshaer AA, Abbas OA, Abdal-Aal MM, Awadallah YA, Al-Saddique AA, Fouda MA. Comparison of neurological outcomes in patients undergoing conventional coronary artery bypass grafting, on-pump beating heart coronary bypass, and off-pump coronary bypass. Neurosciences. 2007;12:35-41	measure not operationalised
Sabik JF, Gillinov AM, Blackstone EH, Vacha C, Houghtaling PL, Navia J, Smedira NG, McCarthy PM, Cosgrove DM, Lytle BW, et al. Does off-pump coronary surgery reduce morbidity and mortality? Journal of Thoracic and Cardiovascular Surgery. 2002;124:698-707	measure not operationalised
Sahu B, Chauhan S, Kiran U, Bisoi A, Lakshmy R, Selvaraj T, Nehra A. Neurocognitive function in patients undergoing coronary artery bypass graft surgery with cardiopulmonary bypass: The effect of two different rewarming strategies. Journal of cardiothoracic and vascular anesthesia. 2009;23:14-21	measure not operationalised
Saito A, Kumamaru H, Ono M, Miyata H, Motomura N. Propensity-matched analysis of a side-clamp versus an anastomosis assist device in cases of isolated coronary artery bypass grafting. European journal of cardio-thoracic surgery : official journal of the European Association for Cardio-thoracic Surgery. 2018;54:889-895	measure not operationalised
Santana Santos F, Tadeu Velasco I. Clinical features in elderly patients submitted to coronary artery bypass graft (cabg). Revista Brasileira de Medicina. 2005;62:96-102	measure not operationalised
Schmidt R, Fazekas F, Offenbacher H, Machler H, Freidl W, Payer F, Rigler B, Harrison MIG, Lechner H. Brain magnetic resonance imaging in coronary artery bypass grafts: A pre- and postoperative assessment. Neurology. 1993;43:775-778	measure not operationalised
Schuch V, Moysidis T, Weiland D, Santosa F, Kroger K. Dementia and amputation. Interventional Medicine & Applied Science. 2012;4:175-180	measure not operationalised
Schwarz N, Schoenburg M, Mollmann H, Kastaun S, Kaps M, Bachmann G, Sammer G, Hamm C, Walther T, Gerriets T. Cognitive decline and ischemic microlesions after coronary catheterization. A comparison to coronary artery bypass grafting. American Heart Journal. 2011;162:756-763	measure not operationalised
Selnes O, Grega M, Bailey M, Pham L, Zeger S, Baumgartner W, McKhann G. Cognition 6 years after surgical or medical therapy for coronary artery disease. Ann Neurol. 2008;63:581-590	measure not operationalised
Selnes O, Grega M, Borowicz L, Royall R, McKhann G, Baumgartner W. Cognitive changes with coronary artery disease: A prospective study of coronary artery bypass graft patients and nonsurgical controls. Ann Thorac Surg. 2003;75:1377-1384; discussion 1384	measure not operationalised
Selnes OA, Grega MA, Bailey MM, Pham L, Zeger S, Baumgartner WA, McKhann GM. Neurocognitive outcomes 3 years after coronary artery bypass graft surgery: A controlled study. The Annals of thoracic surgery. 2007;84:1885-1896	measure not operationalised
Selnes OA, Grega MA, Borowicz Jr LM, Barry S, Zeger S, Baumgartner WA, McKhann GM. Cognitive outcomes three years after coronary artery bypass surgery: A comparison of on-pump coronary artery bypass graft surgery and nonsurgical controls. Annals of Thoracic Surgery. 2005;79:1201-1209	measure not operationalised
Selnes OA, Royall RM, Grega MA, Borowicz LM, Jr., Quaskey S, McKhann GM. Cognitive changes 5 years after coronary artery bypass grafting: Is there evidence of late decline? Archives of neurology. 2001;58:598-604	measure not operationalised
Sendelbach S, Lindquist R, Watanuki S, Roman DD, Savik K. Early neurocognitive function of patients after off-pump coronary artery bypass surgery. Heart & lung : the journal of critical care. 2005;34:367-374	measure not operationalised

Shi Y, Wang W. Application of different anesthetic methods in coronary artery bypass grafting and the effect on postoperative outcome. Experimental & Therapeutic Medicine. 2019;17:695-700	measure not operationalised
Shroyer AL, Grover FL, Hattler B, Collins JF, McDonald GO, Kozora E, Lucke JC, Baltz JH, Novitzky D, Veterans Affairs Randomized On/Off Bypass Study G, et al. On-pump versus off-pump coronary-artery bypass surgery. The New England journal of medicine. 2009;361:1827-1837	measure not operationalised
Shroyer ALW, Hattler B, Wagner TH, Baltz JH, Collins JF, Carr BM, Almassi GH, Quin JA, Hawkins RB, Kozora E, et al. Comparing off-pump and on-pump clinical outcomes and costs for diabetic cardiac surgery patients. Annals of Thoracic Surgery. 2014;98:38-45	measure not operationalised
Skrabal CA, Khosravi A, Westphal B, Steinhoff G, Liebold A. Effects of poly-2-methoxyethylacrylate (pmea)-coating on cpb circuits. Scandinavian cardiovascular journal : SCJ. 2006;40:224-229	measure not operationalised
Srinivasan K, Ravikumar S, Chandra SR, Ganapathy S, Ravi GS. Cerebral and coronary vasculature in disease associations and dissociations in the south indian population. Journal of Neurosciences in Rural Practice. 2017;8:352-356	measure not operationalised
Stamou SC, Bafi AS, Boyce SW, Pfister AJ, Dullum MKC, Hill PC, Zaki S, Garcia JM, Corso PJ. Coronary revascularization of the circumflex system: Different approaches and long-term outcome. Annals of Thoracic Surgery. 2000;70:1371-1377	measure not operationalised
Stazka J, Szepietowska EM, Baranska E, Dudka P, Bedynska BSBBBBBBDDEGGJ-RJJJJKKKMP-ZRSSSSS. The cognitive functioning of patients with heart disease treated cardiosurgically-assessment before and after surgery. Preliminary study. Psychiatria Polska. 2012;46:757-768	measure not operationalised
Stewart A, Katznelson R, Kraeva N, Carroll J, Pickworth T, Rao V, Djaiani G. Genetic variation and cognitive dysfunction one year after cardiac surgery. Anaesthesia. 2013;68:571-575	measure not operationalised
Stockard JJ, Bickford RG, Schauble JF. Pressure-dependent cerebral ischemia during cardiopulmonary bypass. Neurology. 1973;23:521-529	measure not operationalised
Strauss B, Paulsen G, Strenge H, Graetz S, Regensburger D, Speidel H. Preoperative and late postoperative psychosocial state following coronary artery bypass surgery. The Thoracic and cardiovascular surgeon. 1992;40:59-64	measure not operationalised
Stygall J, Newman SP, Fitzgerald G, Steed L, Mulligan K, Arrowsmith JE, Pugsley W, Humphries S, Harrison MJ. Cognitive change 5 years after coronary artery bypass surgery. Health psychology : official journal of the Division of Health Psychology, American Psychological Association. 2003;22:579-586	measure not operationalised
Stygall J, Suvarna S, Harrington J, Hayward M, Walesby RK, Newman SP. Effect on the brain of two techniques of myocardial protection. Asian Cardiovascular & Thoracic Annals. 2009;17:259-265	measure not operationalised
Svenmarker S, Sandström E, Karlsson T, Jansson E, Häggmark S, Lindholm R, Appelblad M, Aberg T. Clinical effects of the heparin coated surface in cardiopulmonary bypass. Eur J Cardiothorac Surg. 1997;11:957-964	measure not operationalised
Szalma I, Kiss A, Kardos L, Horvath G, Nyitrai E, Tordai Z, Csiba L. Piracetam prevents cognitive decline in coronary artery bypass: A randomized trial versus placebo. The Annals of thoracic surgery. 2006;82:1430-1435	measure not operationalised
Tagarakis GI, Tsolaki-Tagaraki F, Tsolaki M, Diegeler A, Kazis D, Rouska E, Papassotiropoulos A. The role of soat-1 polymorphisms in cognitive decline and delirium after bypass heart surgery. Clinical research in cardiology : official journal of the German Cardiac Society. 2007;96:600-603	measure not operationalised
Taggart DP, Browne SM, Halligan PW, Wade DT, Murkin JM. Is cardiopulmonary bypass still the cause of cognitive dysfunction after cardiac operations? Journal of Thoracic and Cardiovascular Surgery. 1999;118:414-421	measure not operationalised
Taggart DP, Browne SM, Wade DT, Halligan PW. Neuroprotection during cardiac surgery: A randomised trial of a platelet activating factor antagonist. Heart (British Cardiac Society). 2003;89:897-900	measure not operationalised
Tarasova IV, Volf NV, Trubnikova OA, Barbarash OL. Electroencephalogram changes in patients undergoing on-pump coronary artery bypass grafting. Neuroscience and Behavioral Physiology. 2013;43:577-581	measure not operationalised
Trubnikova O, Tarasova I, Barbarash O. The influence of low and moderate carotid stenosis on neurophysiologic status of patients undergoing on-pump coronary artery bypass grafting. Frontiers in neurology. 2012;3:1	measure not operationalised

Trubnikova OA, Tarasova IV, Artamonova AI, Syrova ID, Barbarash OL. Age as a risk factor for cognitive impairments in patients undergoing coronary bypass. Neuroscience and Behavioral Physiology. 2013;43:89-92	measure not operationalised
van den Goor JM, Saxby BK, Tijssen JG, Wesnes KA, de Mol BA, Nieuwland R. Improvement of cognitive test performance in patients undergoing primary cabg and other cpb-assisted cardiac procedures. Perfusion. 2008;23:267-273	measure not operationalised
Vlahou A, Diplaris K, Ampatzidou F, Karagounnis L, Drossos G. The role of blood transfusion in the development of atrial fibrillation after coronary artery bypass grafting. The Thoracic and cardiovascular surgeon. 2016;64:688-692	measure not operationalised
Walzer T, Herrmann M, Wallesch CW. Neuropsychological disorders after coronary bypass surgery. Journal of neurology, neurosurgery, and psychiatry. 1997;62:644-648	measure not operationalised
Westaby S, Saatvedt K, White S, Katsumata T, van Oeveren W, Halligan PW. Is there a relationship between cognitive dysfunction and systemic inflammatory response after cardiopulmonary bypass? The Annals of thoracic surgery. 2001;71:667-672	measure not operationalised
Wimmer-Greinecker G, Matheis G, Brieden M, Dietrich M, Oremek G, Westphal K, Winkelmann BR, Moritz A. Neuropsychological changes after cardiopulmonary bypass for coronary artery bypass grafting. The Thoracic and cardiovascular surgeon. 1998;46:207-212	measure not operationalised
Wittwer T, Sabashnikov A, Rahmanian PB, Choi Y-H, Zeriouh M, Mehler TO, Wahlers T. Less invasive coronary artery revascularization with a minimized extracorporeal circulation system: Preliminary results of a comparative study with off-pump-procedures. Journal Of Cardiothoracic Surgery. 2013;8:75	measure not operationalised
Wu M-Y, Lee M-Y, Lin C-C, Chang Y-S, Tsai F-C, Lin P-J. Resuscitation of non-postcardiotomy cardiogenic shock or cardiac arrest with extracorporeal life support: The role of bridging to intervention. Resuscitation. 2012;83:976-981	measure not operationalised
Wu M-Y, Tseng Y-H, Chang Y-S, Tsai F-C, Lin P-J. Using extracorporeal membrane oxygenation to rescue acute myocardial infarction with cardiopulmonary collapse: The impact of early coronary revascularization. Resuscitation. 2013;84:940-945	measure not operationalised
Yamamuro M, Lytle BW, Sapp SK, Cosgrove DM, 3rd, Loop FD, McCarthy PM. Risk factors and outcomes after coronary reoperation in 739 elderly patients. The Annals of thoracic surgery. 2000;69:464-474	measure not operationalised
Yoda M, Nonoyama M, Shimakura T. Cerebral perfusion during off-pump coronary artery bypass grafting. Surgery today. 2004;34:501-505	measure not operationalised
Zhai W, Liu J, Si Y, Han J. Changes in postoperative cognitive function during off-pump coronary artery bypass graft surgery: Dose response of propofol. International Journal of Clinical and Experimental Medicine. 2016;9:10939-10946	measure not operationalised
Zhang TZ, Zhou J, Jin Q, Sun YJ, Diao YG, Zhang YN, Zhang Z. Protective effects of remifentanil preconditioning on cerebral injury during pump-assisted coronary artery bypass graft. Genetics and molecular research : GMR. 2014;13:7658-7665	measure not operationalised
Zhang X, Schmitt FA, Caban-Holt AM, Ding X, Kryscio RJ, Abner E. Diabetes mitigates the role of memory complaint in predicting dementia risk: Results from the prevention of alzheimer's disease with vitamin e and selenium study. Jpad. 2017;4:143-148	measure not operationalised
Zhao G. Effect of continuous nursing intervention on rehabilitation after coronary artery bypass grafting in elderly patients with coronary heart disease. Acta Medica Mediterranea. 2019;35:467-473	measure not operationalised
Zimpfer D, Czerny M, Kilo J, Kasimir M-T, Madl C, Kramer L, Wieselthaler GM, Wolner E, Grimm M. Cognitive deficit after aortic valve replacement. The Annals of thoracic surgery. 2002;74:407-412	measure not operationalised
Anonymous. Two studies: Long-term cognitive decline after bypass surgery; alzheimer's gene linked to earlier bypass. Comprehensive therapy. 1999;25:300-301	news article
Agarwal R, Kalita J, Pandey S, Agarwal SK, Misra UK. Evaluation of cognitive function and p300 in patients undergoing cardiac surgery. Electromyography and clinical neurophysiology. 2010;50:259-264	no CABG results
Andrew MJ, Baker RA, Kneebone AC, Knight JL. Mood state as a predictor of neuropsychological deficits following cardiac surgery. Journal of Psychosomatic Research. 2000;48:537-546	no CABG results

Arenson BG, MacDonald LA, Grocott HP, Hiebert BM, Arora RC. Effect of intensive care unit environment on in-hospital delirium after cardiac surgery. The Journal of thoracic and cardiovascular surgery. 2013;146:172-178	no CABG results
Atalan N, Efe Sevim M, Akgun S, Fazliogullari O, Basaran C. Morphine is a reasonable alternative to haloperidol in the treatment of postoperative hyperactive-type delirium after cardiac surgery. Journal of cardiothoracic and vascular anesthesia. 2013;27:933-938	no CABG results
Baranyi A, Rothenhausler HB. The impact of s100b and persistent high levels of neuron-specific enolase on cognitive performance in elderly patients after cardiopulmonary bypass. Brain Injury. 2013;27:417-424	no CABG results
Barber PA, Hach S, Tippett LJ, Ross L, Merry AF, Milsom P. Cerebral ischemic lesions on diffusion-weighted imaging are associated with neurocognitive decline after cardiac surgery. Stroke. 2008;39:1427-1433	no CABG results
Bashein G, Townes B, Nessly M, Bledsoe S, Hornbein T, Davis K, Goldstein D, Coppel D. A randomized study of carbon dioxide management during hypothermic cardiopulmonary bypass. Anesthesiology. 1990;72:7-15	no CABG results
Basile AM, Fusi C, Conti AA, Paniccia R, Trefoloni G, Pracucci G, Di Carlo A, Noferi D, Carbonetto F, Pretelli P, et al. S-100 protein and neuron-specific enolase as markers of subclinical cerebral damage after cardiac surgery: Preliminary observation of a 6-month follow-up study. European neurology. 2001;45:151-159	no CABG results
Bhudia SK, Cosgrove DM, Naugle RI, Rajeswaran J, Lam B-K, Walton E, Petrich J, Palumbo RC, Gillinov AM, Apperson-Hansen C, et al. Magnesium as a neuroprotectant in cardiac surgery: A randomized clinical trial. The Journal of thoracic and cardiovascular surgery. 2006;131:853-861	no CABG results
Bily B, Artemiou P, Sabol F, Bilecova-Rabajdova M, Kolarcik P, Torok P. The role of dexmedetomidine in the prevention of postoperative delirium in cardiac surgery patients. [slovak]. Cardiology letters,. 2015;24:435-444	no CABG results
Breu A, Stransky M, Metterlein T, Werner T, Trabold B. Subsyndromal delirium after cardiac surgery. Scandinavian Cardiovascular Journal. 2015;49:207-212	no CABG results
Brown CHIV, Morrissey C, Ono M, Yenokyan G, Selnes OA, Walston J, Max L, LaFlam A, Neufeld K, Gottesman RF, et al. Impaired olfaction and risk of delirium or cognitive decline after cardiac surgery. Journal of the American Geriatrics Society. 2015;63:16-23	no CABG results
Brown CHt, Laflam A, Max L, Lymar D, Neufeld KJ, Tian J, Shah AS, Whitman GJ, Hogue CW. The impact of delirium after cardiac surgical procedures on postoperative resource use. The Annals of thoracic surgery. 2016;101:1663-1669	no CABG results
Brown CHt, Probert J, Healy R, Parish M, Nomura Y, Yamaguchi A, Tian J, Zehr K, Mandal K, Kamath V, et al. Cognitive decline after delirium in patients undergoing cardiac surgery. Anesthesiology. 2018;129:406-416	no CABG results
Browndyke JN, Berger M, Harshbarger TB, Smith PJ, White W, Bisanar TL, Alexander JH, Gaca JG, Welsh-Bohmer K, Newman MF, et al. Resting-state functional connectivity and cognition after major cardiac surgery in older adults without preoperative cognitive impairment: Preliminary findings. Journal of the American Geriatrics Society. 2017;65:e6-e12	no CABG results
Browndyke JN, Moser DJ, Cohen RA, O'Brien DJ, Algina JJ, Haynes WG, Staples ED, Alexander J, Davies LK, Bauer RM. Acute neuropsychological functioning following cardiosurgical interventions associated with the production of intraoperative cerebral microemboli. The Clinical neuropsychologist. 2002;16:463-471	no CABG results
Burker EJ, Blumenthal JA, Feldman M, Thyrum E, Mahanna E, White W, Smith LR, Lewis J, Croughwell N, Schell R. The mini mental state exam as a predictor of neuropsychological functioning after cardiac surgery. International Journal of Psychiatry in Medicine. 1995;25:263-276	no CABG results
Buziashvili II, Aleksakhina IA, Ambat'ello SG, Matskeplishvili ST. Use of cognitive evoked potentials p300 in diagnosis of affection of the higher mental functions after surgery on the heart in conditions of cardiopulmonary bypass. Zhurnal nevrologii i psikhiatrii imeni S.S. Korsakova / Ministerstvo zdravookhraneniia i meditsinskoi promyshlennosti Rossiiskoi Federatsii, Vserossiiskoe obshchestvo nevrologov [i] Vserossiiskoe obshchestvo psikhiatrov. 2005;105:51-54	no CABG results
Carrascal Y, Casquero E, Gualis J, Di Stefano S, Florez S, Fulquet E, Echevarria JR, Fiz L. Cognitive decline after cardiac surgery: Proposal for easy measurement with a new test. Interactive cardiovascular and thoracic surgery. 2005;4:216-221	no CABG results

Cereghetti C, Siegemund M, Schaedelin S, Fassl J, Seeberger MD, Eckstein FS, Steiner LA, Goettel N. Independent predictors of the duration and overall burden of postoperative delirium after cardiac surgery in adults: An observational cohort study. Journal of Cardiothoracic and Vascular Anesthesia. 2017;31:1966-1973	no CABG results
Chabot RJ, Gugino LD, Aglio LS, Maddi R, Cote W. Qeeg and neuropsychological profiles of patients after undergoing cardiopulmonary bypass surgical procedures. Clinical EEG (electroencephalography). 1997;28:98-105	no CABG results
Cheng H, Li Z, Young N, Boyd D, Atkins Z, Ji F, Liu H. The effect of dexmedetomidine on outcomes of cardiac surgery in elderly patients. Journal of cardiothoracic and vascular anesthesia. 2016;30:1502-1508	no CABG results
Chuich T, Cropsey CL, Shi Y, Johnson D, Shotwell MS, Henson CP. Perioperative sedation in mechanically ventilated cardiac surgery patients with dexmedetomidine-based versus propofol-based regimens. Annals of Pharmacotherapy. 2019;53:5-12	no CABG results
Cook DJ, Huston J, 3rd, Trenerry MR, Brown RD, Jr., Zehr KJ, Sundt TM, 3rd. Postcardiac surgical cognitive impairment in the aged using diffusion-weighted magnetic resonance imaging. The Annals of thoracic surgery. 2007;83:1389-1395	no CABG results
Croughwell ND, Newman MF, Blumenthal JA, White WD, Lewis JB, Frasco PE, Smith LR, Thyrum EA, Hurwitz BJ, Leone BJ. Jugular bulb saturation and cognitive dysfunction after cardiopulmonary bypass. The Annals of thoracic surgery. 1994;58:1702-1708	no CABG results
de Vries AJ, Vermeijden WJ, van Pelt LJ, van den Heuvel ER, van Oeveren W. Additional filtering of blood from a cell salvage device is not likely to show important additional benefits in outcome in cardiac surgery. Transfusion. 2019;59:989-994	no CABG results
Degirmenci B, Durak H, Hazan E, Karabay O, Derebek E, Yilmaz M, Ozbilek E, Oto O. The effect of coronary artery bypass surgery on brain perfusion. Journal of nuclear medicine : official publication, Society of Nuclear Medicine. 1998;39:587-591	no CABG results
Di Carlo A, Perna AM, Pantoni L, Basile AM, Bonacchi M, Pracucci G, Trefoloni G, Bracco L, Sangiovanni V, Piccini C, et al. Clinically relevant cognitive impairment after cardiac surgery: A 6-month follow-up study. Journal of the Neurological Sciences. 2001;188:85-93	no CABG results
Diab MS, Bilkhu R, Soppa G, Edsell M, Fletcher N, Heiberg J, Royse C, Jahangiri M. The influence of prolonged intensive care stay on quality of life, recovery, and clinical outcomes following cardiac surgery: A prospective cohort study. Journal of Thoracic and Cardiovascular Surgery. 2018;156:1906-1915.e1903	no CABG results
Eghtesadi-Araghi P, Sohrabpour A, Vahedi H, Saberi-Firoozi M. Halothane hepatitis in iran: A review of 59 cases. World journal of gastroenterology. 2008;14:5322-5326	no CABG results
Eifert S, Reichenspurner H, Pfefferkorn T, Baur B, von Schlippenbach C, Mayer TE, Hamann G, Reichart B. Neurological and neuropsychological examination and outcome after use of an intra-aortic filter device during cardiac surgery. Perfusion. 2003;18 Suppl 1:55-60	no CABG results
Eizadi-Mood N, Aghadavoudi O, Najarzadegan MR, Fard MM. Prevalence of delirium in opium users after coronary artery bypass graft surgery. International Journal of Preventive Medicine. 2014;5:900-906	no CABG results
Eljezi V, Imhoff E, Bourdeaux D, Pereira B, Farhat M, Schoeffler P, Azarnoush K, Duale C. Bilateral sternal infusion of ropivacaine and length of stay in icu after cardiac surgery with increased respiratory risk a randomised controlled trial. European Journal of Anaesthesiology. 2017;34:56-65	no CABG results
Eljezi V, Imhoff E, Bourdeaux D, Pereira B, Farhat M, Schoeffler P, Azarnoush K, Duale C. Bilateral sternal infusion of ropivacaine and length of stay in icu after cardiac surgery with increased respiratory risk: A randomised controlled trial. European Journal of Anaesthesiology. 2017;34:56-65	no CABG results
Fontes MT, McDonagh DL, Phillips-Bute B, Welsby IJ, Podgoreanu MV, Fontes ML, Stafford-Smith M, Newman MF, Mathew JP. Arterial hyperoxia during cardiopulmonary bypass and postoperative cognitive dysfunction. Journal of cardiothoracic and vascular anesthesia. 2014;28:462-466	no CABG results
Gandhi GY, Nuttall GA, Abel MD, Mullany CJ, Schaff HV, Williams BA, Schrader LM, Rizza RA, McMahon MM. Intraoperative hyperglycemia and perioperative outcomes in cardiac surgery patients. Mayo Clinic proceedings. 2005;80:862-866	no CABG results
Ganguly G, Dixit V, Patrikar S, Venkatraman R, Gorthi SP, Tiwari N. Carbon dioxide insufflation and neurocognitive outcome of open heart surgery. Asian Cardiovascular and Thoracic Annals. 2015;23:774-780	no CABG results

Georgiadis D, Berger A, Kowatschev E, Lautenschlager C, Borner A, Lindner A, Schulte-Mattler W, Zerkowski HR, Zierz S, Deufel T. Predictive value of s-100beta and neuron-specific enolase serum levels for adverse neurologic outcome after cardiac surgery. The Journal of thoracic and cardiovascular surgery. 2000;119:138-147	no CABG results
Ghaffary S, Ghaeli P, Talasaz AH, Karimi A, Noroozian M, Salehiomran A, Jalali A. Effect of memantine on post-operative cognitive dysfunction after cardiac surgeries: A randomized clinical trial. DARU, Journal of Pharmaceutical Sciences. 2017;25 (1) (no pagination)	no CABG results
Grieco G, d'Hollosy M, Culliford AT, Jonas S. Evaluating neuroprotective agents for clinical anti-ischemic benefit using neurological and neuropsychological changes after cardiac surgery under cardiopulmonary bypass. Methodological strategies and results of a double-blind, placebo-controlled trial of gm1 ganglioside. Stroke. 1996;27:858-874	no CABG results
Grondin CM, Meere C, Castonguay Y, Grondin P, Lepage G. Cardiac surgery in the aged: A review of 120 cases. Cardiovascular clinics. 1971;2:233-245	no CABG results
Gugino LD, Chabot RJ, Aglio LS, Aranki S, Dekkers R, Maddi R. Qeeg changes during cardiopulmonary bypass: Relationship to postoperative neuropsychological function. Clinical EEG Electroencephalography. 1999;30:53-63	no CABG results
Gugino LD, Chabot RJ, Aglio LS, Maddi R, Gosnell J, Aranki S. Qeeg and neuropsychological profiles of patients prior to undergoing cardiopulmonary bypass surgical procedures. Clinical EEG (electroencephalography). 1997;28:87-97	no CABG results
Gunstad J, Benitez A, Hoth KF, Spitznagel MB, McCaffery J, McGeary J, Kakos LS, Poppas A, Paul RH, Jefferson AL, et al. P-selectin 1087g/a polymorphism is associated with neuropsychological test performance in older adults with cardiovascular disease. Stroke. 2009;40:2969-2972	no CABG results
Harciarek M, Williamson JB, Biedunkiewicz B, Lichodziejewska-Niemierko M, Debska-Slizien A, Rutkowski B. Memory performance in adequately dialyzed patients with end-stage renal disease: Is there an association with coronary artery bypass grafting? Journal of clinical and experimental neuropsychology. 2010;32:881-889	no CABG results
Hayashi K, Oshima H, Shimizu M, Kobayashi K, Matsui S, Nishida Y, Usui A. Preoperative 6-minute walk distance is associated with postoperative cognitive dysfunction. Annals of Thoracic Surgery. 2018;106:505-512	no CABG results
Herrmann M, Ebert AD, Galazky I, Wunderlich MT, Kunz WS, Huth C. Neurobehavioral outcome prediction after cardiac surgery: Role of neurobiochemical markers of damage to neuronal and glial brain tissue. Stroke. 2000;31:645-650	no CABG results
Hogue CW, Fucetola R, Hershey T, Freedland K, Davila-Roman VG, Goate AM, Thompson RE. Risk factors for neurocognitive dysfunction after cardiac surgery in postmenopausal women. The Annals of thoracic surgery. 2008;86:511-516	no CABG results
Hogue CW, Jr., Freedland K, Hershey T, Fucetola R, Nassief A, Barzilai B, Thomas B, Birge S, Dixon D, Schechtman KB, et al. Neurocognitive outcomes are not improved by 17beta-estradiol in postmenopausal women undergoing cardiac surgery. Stroke. 2007;38:2048-2054	no CABG results
Holinski S, Claus B, Alaaraj N, Dohmen PM, Neumann K, Uebelhack R, Konertz W. Cerebroprotective effect of piracetam in patients undergoing open heart surgery. Annals of Thoracic and Cardiovascular Surgery. 2011;17:137-142	no CABG results
Hudetz JA, Hoffmann RG, Patterson KM, Byrne AJ, Iqbal Z, Gandhi SD, Warltier DC, Pagel PS. Preoperative dispositional optimism correlates with a reduced incidence of postoperative delirium and recovery of postoperative cognitive function in cardiac surgical patients. Journal of cardiothoracic and vascular anesthesia. 2010;24:560-567	no CABG results
Hudetz JA, Patterson KM, Iqbal Z, Gandhi SD, Pagel PS. Metabolic syndrome exacerbates short-term postoperative cognitive dysfunction in patients undergoing cardiac surgery: Results of a pilot study. Journal of cardiothoracic and vascular anesthesia. 2011;25:282-287	no CABG results
Ille R, Lahousen T, Schweiger S, Hofmann P, Kapfhammer HP. Influence of patient-related and surgery-related risk factors on cognitive performance, emotional state, and convalescence after cardiac surgery. Cardiovascular Revascularization Medicine. 2007;8:166-169	no CABG results
Isgro F, Schmidt C, Pohl P, Saggau W. A predictive parameter in patients with brain related complications after cardiac surgery? European journal of cardio-thoracic surgery : official journal of the European Association for Cardio-thoracic Surgery. 1997;11:640-644	no CABG results

Jacobs A, Neveling M, Horst M, Ghaemi M, Kessler J, Eichstaedt H, Rudolf J, Model P, Bonner H, de Vivie ER, et al. Alterations of neuropsychological function and cerebral glucose metabolism after cardiac surgery are not related only to intraoperative microembolic events. Stroke. 1998;29:660-667	no CABG results
Jarvela K, Porkkala H, Karlsson S, Martikainen T, Selander T, Bendel S. Postoperative delirium in cardiac surgery patients. Journal of Cardiothoracic and Vascular Anesthesia. 2018;32:1597-1602	no CABG results
Jenkins CD, Jono RT, Stanton B-A, Stroup-Benham CA. The measurement of health-related quality of life: Major dimensions identified by factor analysis. Social Science & Medicine. 1990;31:925-931	no CABG results
Ji F, Li Z, Nguyen H, Young N, Shi P, Fleming N, Liu H. Perioperative dexmedetomidine improves outcomes of cardiac surgery. Circulation. 2013;127:1576-1584	no CABG results
Jones RN, Rudolph JL, Inouye SK, Yang FM, Fong TG, Milberg WP, Tommet D, Metzger ED, Cupples LA, Marcantonio ER. Development of a unidimensional composite measure of neuropsychological functioning in older cardiac surgery patients with good measurement precision. Journal of clinical and experimental neuropsychology. 2010;32:1041-1049	no CABG results
Jung P, Pereira MA, Hiebert B, Song X, Rockwood K, Tangri N, Arora RC. The impact of frailty on postoperative delirium in cardiac surgery patients. Journal of Thoracic and Cardiovascular Surgery. 2015;149:869	no CABG results
Kaukuntla H, Walker A, Harrington D, Jones T, Bonser RS, Study G. Differential brain and body temperature during cardiopulmonary bypass--a randomised clinical study. European journal of cardio-thoracic surgery : official journal of the European Association for Cardio-thoracic Surgery. 2004;26:571-579	no CABG results
Kazmierski J, Kowman M, Banach M, Fendler W, Okonski P, Banys A, Jaszewski R, Rysz J, Mikhailidis DP, Sobow T, et al. Incidence and predictors of delirium after cardiac surgery: Results from the ipdacs study. Journal of Psychosomatic Research. 2010;69:179-185	no CABG results
Khan E, Brieger D, Amerena J, Atherton JJ, Chew DP, Farshid A, Ilton M, Juergens CP, Kangaharan N, Rajaratnam R, et al. Differences in management and outcomes for men and women with st-elevation myocardial infarction. Medical Journal of Australia. 2018;209:118-123	no CABG results
Kilminster S, Treasure T, McMillan T, Holt DW. Neuropsychological change and s-100 protein release in 130 unselected patients undergoing cardiac surgery. Stroke. 1999;30:1869-1874	no CABG results
Kim DH, Huybrechts KF, Patorno E, Marcantonio ER, Park Y, Levin R, Abdurrob A, Bateman BT. Adverse events associated with antipsychotic use in hospitalized older adults after cardiac surgery. Journal of the American Geriatrics Society. 2017;65:1229-1237	no CABG results
Kolkka R, Hilberman M. Neurologic dysfunction following cardiac operation with low-flow, low-pressure cardiopulmonary bypass. The Journal of thoracic and cardiovascular surgery. 1980;79:432-437	no CABG results
Krzych LJ, Wybraniec MT, Krupka-Matuszczyk I, Skrzypek M, Bolkowska A, Wilczynski M, Bochenek AA. Detailed insight into the impact of postoperative neuropsychiatric complications on mortality in a cohort of cardiac surgery subjects: A 23,000-patient-year analysis. Journal of cardiothoracic and vascular anesthesia. 2014;28:448-457	no CABG results
Lahariya S, Grover S, Bagga S, Sharma A. Delirium in patients admitted to a cardiac intensive care unit with cardiac emergencies in a developing country: Incidence, prevalence, risk factor and outcome. General Hospital Psychiatry. 2014;36:156-164	no CABG results
Lee A, Mu JL, Chiu CH, Gin T, Underwood MJ, Joynt GM. Effect of motor subtypes of delirium in the intensive care unit on fast-track failure after cardiac surgery. Journal of Thoracic and Cardiovascular Surgery. 2018;155:268-275.e261	no CABG results
Lee J, Jung J, Noh J, Yoo S, Hong Y. Perioperative psycho-educational intervention can reduce postoperative delirium in patients after cardiac surgery: A pilot study. International Journal of Psychiatry in Medicine. 2013;45:143-158	no CABG results
Mardani D, Bigdelian H. Prophylaxis of dexamethasone protects patients from further post-operative delirium after cardiac surgery: A randomized trial. Journal of research in medical sciences : the official journal of Isfahan University of Medical Sciences. 2013;18:137-143	no CABG results
Mardani D, Bigdelian H. The effect of dexamethasone prophylaxis on postoperative delirium after cardiac surgery: A randomized trial. Journal of Research in Medical Sciences. 2012;17:S113-s119	no CABG results

Mathew J, Shernan S, White W, Fitch J, Chen J, Bell L, Newman M. Preliminary report of the effects of complement suppression with pexelizumab on neurocognitive decline after coronary artery bypass graft surgery. Stroke. 2004;35:2335-2339	no CABG results
Mathew JP, Fontes ML, Tudor IC, Ramsay J, Duke P, Mazer CD, Barash PG, Hsu PH, Mangano DT. A multicenter risk index for atrial fibrillation after cardiac surgery. Journal of the American Medical Association. 2004;291:1720-1729	no CABG results
Mathew JP, White WD, Schinderle DB, Podgoreanu MV, Berger M, Milano CA, Laskowitz DT, Stafford-Smith M, Blumenthal JA, Newman MF. Intraoperative magnesium administration does not improve neurocognitive function after cardiac surgery. Stroke. 2013;44:3407-3413	no CABG results
McDaid CM, Lewis SA, McMurray T, Phillips SA. Heart surgery: What are the neuropsychological consequences? Special Issue: Heart disease: The psychological challenge. 1994;15:110-125	no CABG results
Meybohm P, Renner J, Broch O, Caliebe D, Albrecht M, Cremer J, Haake N, Scholz J, Zacharowski K, Bein B. Postoperative neurocognitive dysfunction in patients undergoing cardiac surgery after remote ischemic preconditioning: A double-blind randomized controlled pilot study. PloS one. 2013;8:e64743	no CABG results
Mitchell SJ, Merry AF, Frampton C, Davies E, Grieve D, Mills BP, Webster CS, Milsom FP, Willcox TW, Gorman DF. Cerebral protection by lidocaine during cardiac operations: A follow-up study. The Annals of thoracic surgery. 2009;87:820-825	no CABG results
Moser DJ, Cohen RA, Clark MM, Aloia MS, Tate BA, Stefanik S, Forman DE, Tilkemeier PL. Neuropsychological functioning among cardiac rehabilitation patients. Journal of cardiopulmonary rehabilitation. 1999;19:91-97	no CABG results
Mufti HN, Hirsch GM. Perioperative prediction of agitated (hyperactive) delirium after cardiac surgery in adults - the development of a practical scorecard. Journal of Critical Care. 2017;42:192-199	no CABG results
Nathan HJ, Munson J, Wells G, Mundi C, Balaa F, Wynands JE. The management of temperature during cardiopulmonary bypass: Effect on neuropsychological outcome. Journal of Cardiac Surgery. 1995;10:481-487	no CABG results
Nguyen Q, Uminski K, Hiebert BM, Tangri N, Arora RC. Midterm outcomes after postoperative delirium on cognition and mood in patients after cardiac surgery. Journal of Thoracic & Cardiovascular Surgery. 2018;155:660-667.e662	no CABG results
Nussmeier NA, Miao Y, Roach GW, Wolman RL, Mora-Mangano C, Fox M, Szekely A, Tommasino C, Schwann NM, Mangano DT. Predictive value of the national institutes of health stroke scale and the mini-mental state examination for neurologic outcome after coronary artery bypass graft surgery. Journal of Thoracic and Cardiovascular Surgery. 2010;139:901-912	no CABG results
Ogawa M, Izawa KP, Satomi-Kobayashi S, Tsuboi Y, Komaki K, Gotake Y, Sakai Y, Tanaka H, Okita Y. Impact of delirium on postoperative frailty and long term cardiovascular events after cardiac surgery. PLoS ONE [Electronic Resource]. 2017;12 (12) (no pagination)	no CABG results
O'Neal JB, Billings FT, Liu X, Shotwell MS, Liang Y, Shah AS, Ehrenfeld JM, Wanderer JP, Shaw AD. Effect of preoperative beta-blocker use on outcomes following cardiac surgery. American Journal of Cardiology. 2017;120:1293-1297	no CABG results
Osse RJ, Fekkes D, Tulen JHM, Wierdsma AI, Bogers AJJC, van der Mast RC, Hengeveld MW. High preoperative plasma neopterin predicts delirium after cardiac surgery in older adults. Journal of the American Geriatrics Society. 2012;60:661-668	no CABG results
Patel N, Horsfield MA, Banahan C, Janus J, Masters K, Morlese J, Egan V, Chung EML. Impact of perioperative infarcts after cardiac surgery. Stroke. 2015;46:680-686	no CABG results
Pesonen A, Suojaranta-Ylinen R, Hammarn E, Kontinen VK, Raivio P, Tarkkila P, Rosenberg PH. Pregabalin has an opioid-sparing effect in elderly patients after cardiac surgery: A randomized placebo-controlled trial. British Journal of Anaesthesia. 2011;106:873-881	no CABG results
Poole L, Ronaldson A, Kidd T, Leigh E, Jahangiri M, Steptoe A. Pre-operative cognitive functioning and inflammatory and neuroendocrine responses to cardiac surgery. Annals of behavioral medicine : a publication of the Society of Behavioral Medicine. 2016;50:545-553	no CABG results
Prakanrattana U, Prapaitrakool S. Efficacy of risperidone for prevention of postoperative delirium in cardiac surgery. Anaesthesia and intensive care. 2007;35:714-719	no CABG results

Ramlawi B, Otu H, Rudolph JL, Mieno S, Kohane IS, Can H, Libermann TA, Marcantonio ER, Bianchi C, Sellke FW. Genomic expression pathways associated with brain injury after cardiopulmonary bypass. The Journal of thoracic and cardiovascular surgery. 2007;134:996-1005	no CABG results
Ramlawi B, Rudolph JL, Mieno S, Feng J, Boodhwani M, Khabbaz K, Levkoff SE, Marcantonio ER, Bianchi C, Sellke FW. C-reactive protein and inflammatory response associated to neurocognitive decline following cardiac surgery. Surgery. 2006;140:221-226	no CABG results
Ramlawi B, Rudolph JL, Mieno S, Khabbaz K, Sodha NR, Boodhwani M, Levkoff SE, Marcantonio ER, Sellke FW. Serologic markers of brain injury and cognitive function after cardiopulmonary bypass. Annals of surgery. 2006;244:593-601	no CABG results
Rasmussen LA, Ryhammer PK, Greisen J, Bhavsar RR, Lorentzen A-G, Jakobsen C-J. Ultrashort acting remifentanil is not superior to long-acting sufentanil in preserving cognitive function-a randomized study. Journal of Clinical Anesthesia. 2016;33:127-134	no CABG results
Regragui I, Birdi I, Izzat MB, Black AM, Lopatatzidis A, Day CJ, Gardner F, Bryan AJ, Angelini GD. The effects of cardiopulmonary bypass temperature on neuropsychologic outcome after coronary artery operations: A prospective randomized trial. The Journal of thoracic and cardiovascular surgery. 1996;112:1036-1045	no CABG results
Richebe P, Picard W, Rivat C, Jelacic S, Branchard O, Leproust S, Cahana A, Janvier G. Effects of nefopam on early postoperative hyperalgesia after cardiac surgery. Journal of cardiothoracic and vascular anesthesia. 2013;27:427-435	no CABG results
Rogers CA, Stoica S, Ellis L, Stokes EA, Wordsworth S, Dabner L, Clayton G, Downes R, Nicholson E, Bennett S, et al. Randomized trial of near-infrared spectroscopy for personalized optimization of cerebral tissue oxygenation during cardiac surgery. British Journal of Anaesthesia. 2017;119:384-393	no CABG results
Ropacki SA, Patel SM, Hartman RE. Pomegranate supplementation protects against memory dysfunction after heart surgery: A pilot study. Evidence-based complementary and alternative medicine : eCAM. 2013;2013:932401	no CABG results
Rosengart T, Sweet J, Finnin E, Wolfe P, Cashy J, Hahn E, Marymont J, Sanborn T. Neurocognitive functioning in patients undergoing coronary artery bypass graft surgery or percutaneous coronary intervention: Evidence of impairment before intervention compared with normal controls. Ann Thorac Surg. 2005;80:1327-1334; discussion 1334	no CABG results
Rothenhausler H-B, Grieser B, Nollert G, Reichart B, Schelling G, Kapfhammer H-P. Psychiatric and psychosocial outcome of cardiac surgery with cardiopulmonary bypass: A prospective 12-month follow-up study. General Hospital Psychiatry. 2005;27:18-28	no CABG results
Rudolph JL, Inouye SK, Jones RN, Yang FM, Fong TG, Levkoff SE, Marcantonio ER. Delirium: An independent predictor of functional decline after cardiac surgery. Journal of the American Geriatrics Society. 2010;58:643-649	no CABG results
Saczynski JS, Marcantonio ER, Quach L, Fong TG, Gross A, Inouye SK, Jones RN. Cognitive trajectories after postoperative delirium. The New England journal of medicine. 2012;367:30-39	no CABG results
Sakurai M, Takahara Y, Takeuchi S, Mogi K. Cognitive dysfunction following cardiovascular surgery. The Japanese journal of thoracic and cardiovascular surgery : official publication of the Japanese Association for Thoracic Surgery = Nihon Kyobu Geka Gakkai zasshi. 2005;53:251-254	no CABG results
Salzwedel A, Heidler MD, Meng K, Schikora M, Wegscheider K, Reibis R, Voller H. Impact of cognitive performance on disease-related knowledge six months after multi-component rehabilitation in patients after an acute cardiac event. European Journal of Preventive Cardiology. 2019;26:46-55	no CABG results
Savageau JA, Stanton BA, Jenkins CD, Frater RWM. Neuropsychological dysfunction following elective cardiac operation. Ii. A six-month reassessment. Journal of Thoracic and Cardiovascular Surgery. 1982;84:595-600	no CABG results
Savageau JA, Stanton BA, Jenkins CD, Klein MD. Neuropsychological dysfunction following elective cardiac operation. I. Early assessment. The Journal of thoracic and cardiovascular surgery. 1982;84:585-594	no CABG results
Schoen J, Husemann L, Tiemeyer C, Lueloh A, Sedemund-Adib B, Berger KU, Hueppe M, Heringlake M. Cognitive function after sevoflurane- vs propofol-based anaesthesia for on-pump cardiac surgery: A randomized controlled trial. British Journal of Anaesthesia. 2011;106:840-850	no CABG results
Schricker T, Sato H, Beaudry T, Codere T, Hatzakorzian R, Pruessner JC. Intraoperative maintenance of normoglycemia with insulin and glucose preserves verbal learning after cardiac surgery. PloS one. 2014;9:no pagination	no CABG results

Selnes OA, Grega MA, Bailey MM, Pham LD, Zeger SL, Baumgartner WA, McKhann GM. Do management strategies for coronary artery disease influence 6-year cognitive outcomes? The Annals of thoracic surgery. 2009;88:445-454	no CABG results
Shadvar K, Baastani F, Mahmoodpoor A, Bilehjani E. Evaluation of the prevalence and risk factors of delirium in cardiac surgery icu. Journal of Cardiovascular and Thoracic Research. 2013;5:157-161	no CABG results
Shehabi Y, Grant P, Wolfenden H, Hammond N, Bass F, Campbell M, Chen J. Prevalence of delirium with dexmedetomidine compared with morphine based therapy after cardiac surgery: A randomized controlled trial (dexmedetomidine compared to morphine-dexcom study). Anesthesiology. 2009;111:1075-1084	no CABG results
Shioiri A, Kurumaji A, Takeuchi T, Nemoto K, Arai H, Nishikawa T. A decrease in the volume of gray matter as a risk factor for postoperative delirium revealed by an atlas-based method. American Journal of Geriatric Psychiatry. 2016;24:528-536	no CABG results
Skoloudik D, Hurtikova E, Brat R, Herzig R. Sonolysis in prevention of brain infarction during cardiac surgery (sonorescue). Medicine (United States). 2016;95:no pagination	no CABG results
Smulter N, Lingehall HC, Gustafson Y, Olofsson B, Engstrom KG, Appelblad M, Svenmarker S. Disturbances in oxygen balance during cardiopulmonary bypass: A risk factor for postoperative delirium. Journal of Cardiothoracic and Vascular Anesthesia. 2018;32:684-690	no CABG results
Snyder-Ramos SA, Gruhlke T, Bauer H, Bauer M, Luntz AP, Motsch J, Martin E, Vahl CF, Missler U, Wiesmann M, et al. Cerebral and extracerebral release of protein s100b in cardiac surgical patients. Anaesthesia. 2004;59:344-349	no CABG results
Soehle M, Dittmann A, Ellerkmann RK, Baumgarten G, Putensen C, Guenther U. Intraoperative burst suppression is associated with postoperative delirium following cardiac surgery: A prospective, observational study. BMC Anesthesiology. 2015;15:no pagination	no CABG results
Sponholz C, Schuwirth C, Koenig L, Hoyer H, Coldewey SM, Schelenz C, Doenst T, Kortgen A, Bauer M. Intraoperative reduction of vasopressors using processed electroencephalographic monitoring in patients undergoing elective cardiac surgery: A randomized clinical trial. Journal of Clinical Monitoring & Computing. 2019;19:19	no CABG results
Susheela AT, Packiasabapathy S, Gasangwa DV, Patxot M, O'Neal J, Marcantonio E, Subramaniam B. The use of dexmedetomidine and intravenous acetaminophen for the prevention of postoperative delirium in cardiac surgery patients over 60 years of age: A pilot study. F1000Research. 2017;6:1842	no CABG results
Svensson LG, Nadolny EM, Penney DL, Jacobson J, Kimmel WA, Entrup MH, D'Agostino RS. Prospective randomized neurocognitive and s-100 study of hypothermic circulatory arrest, retrograde brain perfusion, and antegrade brain perfusion for aortic arch operations. Annals of Thoracic Surgery. 2001;71:1905-1912	no CABG results
Tan MC, Felde A, Kuskowski M, Ward H, Kelly RF, Adabag AS, Dysken M. Incidence and predictors of post-cardiotomy delirium. American Journal of Geriatric Psychiatry. 2008;16:575-583	no CABG results
Tardiff BE, Newman MF, Saunders AM, Strittmatter WJ, Blumenthal JA, White WD, Croughwell ND, Davis Jr RD, Roses AD, Reves JG. Preliminary report of a genetic basis for cognitive decline after cardiac operations. Annals of Thoracic Surgery. 1997;64:715-720	no CABG results
ten Broeke M, Koster S, Konings T, Hensens AG, van der Palen J. Can we predict a delirium after cardiac surgery? A validation study of a delirium risk checklist. European Journal of Cardiovascular Nursing. 2018;17:255-261	no CABG results
Terazawa S, Oshima H, Narita Y, Fujimoto K, Mutsuga M, Tokuda Y, Yoshizumi T, Ito H, Uchida W, Usui A. Strategy of cardiovascular surgery for patients with dementia as evaluated by mini-mental state examination. Circulation Journal. 2018;82:2998-3004	no CABG results
Townes BD, Bashein G, Hornbein TF, Coppel DB, Goldstein DE, Davis KB, Nessly ML, Bledsoe SW, Veith RC, Ivey TD, et al. Neurobehavioral outcomes in cardiac operations. A prospective controlled study. Journal of Thoracic and Cardiovascular Surgery. 1989;98:774-782	no CABG results
Trubnikova OA, Tarasova IV, Syrova ID, Mamontova AS, Kovalenko AV, Barbarash OL, Barbarash LS. Neuropsychological status of patients with minor and moderate carotid artery stenosis undergoing coronary bypass. Neuroscience and Behavioral Physiology. 2014;44:625-630	no CABG results
Tse L, Schwarz SKW, Bowering JB, Moore RL, Barr AM. Incidence of and risk factors for delirium after cardiac surgery at a quaternary care center: A retrospective cohort study. Journal of cardiothoracic and vascular anesthesia. 2015;29:1472-1479	no CABG results

Uebelhack R, Vohs K, Zytowski M, Schewe HJ, Koch C, Konertz W. Effect of piracetam on cognitive performance in patients undergoing bypass surgery. Pharmacopsychiatry. 2003;36:89-93	no CABG results
Uysal S, Mazzeffi M, Lin HM, Fischer GW, Griepp RB, Adams DH, Reich DL. Internet-based assessment of postoperative neurocognitive function in cardiac and thoracic aortic surgery patients. Journal of Thoracic and Cardiovascular Surgery. 2011;141:777-781	no CABG results
Vedel AG, Holmgaard F, Siersma V, Langkilde A, Paulson OB, Ravn HB, Nilsson JC, Rasmussen LS. Domain-specific cognitive dysfunction after cardiac surgery. A secondary analysis of a randomized trial. Acta Anaesthesiologica Scandinavica. 2019;19:19	no CABG results
Veliz-Reissmuller G, Torres HA, Van der Linden J, Lindblom D, Jonhagen ME. Pre-operative mild cognitive dysfunction predicts risk for post-operative delirium after elective cardiac surgery. Aging - Clinical and Experimental Research. 2007;19:172-177	no CABG results
Vingerhoets G, de Soete G, Jannes C. Subjective complaints versus neuropsychological test performance after cardiopulmonary bypass. Journal of Psychosomatic Research. 1995;39:843-853	no CABG results
Vingerhoets G, Jannes C, De Soete G, Van Nooten G. Prospective evaluation of verbal memory performance after cardiopulmonary bypass surgery. Journal of clinical and experimental neuropsychology. 1996;18:187-196	no CABG results
Vingerhoets G, Van Nooten G, Jannes C. Effect of asymptomatic carotid artery disease on cognitive outcome after cardiopulmonary bypass. Journal of the International Neuropsychological Society : JINS. 1996;2:236-239	no CABG results
Wanat M, Fitousis K, Boston F, Masud F. Comparison of dexmedetomidine versus propofol for sedation in mechanically ventilated patients after cardiovascular surgery. Methodist DeBakey cardiovascular journal. 2014;10:111-117	no CABG results
Whitlock RP, Devereaux PJ, Teoh KH, Lamy A, Vincent J, Pogue J, Paparella D, Sessler DI, Karthikeyan G, Villar JC, et al. Methylprednisolone in patients undergoing cardiopulmonary bypass (sirs): A randomised, double-blind, placebo-controlled trial. The Lancet. 2015;386:1243-1253	no CABG results
Willner AE, Rabiner CJ, Wisoff BG, Fishman J, Rosen B, Hartstein M, Klein DF. Analogy tests and psychopathology at follow-up after open heart surgery. Biological psychiatry. 1976;11:687-696	no CABG results
Willner AE, Rabiner CJ. Psychopathology and cognitive dysfunction five years after open-heart surgery. Comprehensive psychiatry. 1979;20:409-418	no CABG results
Zhang W, Sun Y, Liu Y, Qiu W, Ye X, Zhang G, Zhang L. A nursing protocol targeting risk factors for reducing postoperative delirium in patients following coronary artery bypass grafting: Results of a prospective before-after study. International Journal of Nursing Sciences. 2017:no pagination	no CABG results
Zhang X, Yan X, Gorman J, Hoffman SN, Zhang L, Boscarino JA. Perioperative hyperglycemia is associated with postoperative neurocognitive disorders after cardiac surgery. Neuropsychiatric disease and treatment. 2014;10:361-370	no CABG results
Abdallah I, Ayad AE, Afifi W, Amin ME. Lidocaine versus magnesium infusion for cerebral protection during cardiopulmonary bypass. Egyptian Journal of Anaesthesia. 2003;19:195-204	no full text
Bi Q, Zhang WW, Li JY, Wu H. Relationship between impaired baseline cerebral perfusion and cognitive dysfunction after off-pump coronary artery bypass grafting. Experimental and Clinical Cardiology. 2014;20:497-516	no full text
Brusa G, Montano V, Raiteri U, Rissotto R, Grasso MA, Pergolo A, Crosetti S, Ottonello GA. Tcd monitoring in subjects submitted to aorto-coronary by-pass: Correlations with cognitive impairment. Rivista di Neurobiologia. 1997;43:109-114	no full text
Capdeville M, Lee JH. Off-pump coronary artery bypass surgery and anesthesia. Progress in Anesthesiology. 2003;17:163-179	no full text
Newman MF, Croughwell ND, Blumenthal JA, White WD, Lewis JB, Smith LR, Frasco P, Towner EA, Schell RM, Hurwitz BJ, et al. Effect of aging on cerebral autoregulation during cardiopulmonary bypass: Association with postoperative cognitive dysfunction. Circulation. 1994;90:II243-II249	no full text
Zwolinski R, Marcinkiewicz A, Kedzia K, Susik M, Ostrowski S, Jaszewski R. Delirium after surgical myocardial revascularization- identifying the target patients. Experimental and Clinical Cardiology. 2014;20:145-154	no full text

Akgul Erdil F, Durak P, Iyican D, Erdemli O, Kapadar H, Karakas S, Emir M, Sener E. Does fast-track anesthesia technique have advantage on the short term memory functions and depression in coronary artery bypass surgery? Gogus-Kalp-Damar Anestezi ve Yogun Bakim Dernegi Dergisi. 2004;10:111-116	not in english
Alekseevich GY, Rodikov MV, Marchenko AV, Myalyuk PA, Alekseevich GV. An analysis of postoperative cognitive dysfunction in different methods of coronary artery bypass. [russian]. Zhurnal Nevrologii i Psihiatrii imeni S.S. 2017;Korsakova. 117:16-20	not in english
Argunova YA, Trubnikova OA, Mamontova AS, Syrova ID, Kuhareva IN, Maleva OV, Barbarash OL. The influence of three-week aerobic exercise program on neurodynamic parameters of patients underwent coronary bypass grafting. Russian Journal of Cardiology. 2016;130:30-36	not in english
Asenbaum S, Zeitlhofer J, Spiss C, Wolner E, Deecke L. [neurologic and psychiatric complications after heart surgery]. Neurologische und psychiatrische Komplikationen nach Herzoperationen. 1991;69:368-373	not in english
Bagheri K, Honarmand A, Hosseini-Moosa SJ. Association of mean arterial pressure during cardiopulmonary pump and incidence of delirium after coronary artery bypass graft surgery. [persian]. Journal of Isfahan Medical School. 2017;35:1358-1363	not in english
Bagheri K, Motamedi O, Aghadavoudi O, Akbari M. The effects of mean arterial pressure during cardiopulmonary bypass on clinical and paraclinical parameters during and after coronary artery bypass graft surgery. Journal of Isfahan Medical School. 2012;29:no pagination	not in english
Benetis R, Girdauskas E, Kinduris S. Neuropsychological outcomes after coronary artery bypass grafting. Medicina (Kaunas, Lithuania). 2004;40 Suppl 1:30-34	not in english
Benetis R, Kinduris S, Girdauskas E, Ereminiene E. Central nervous system complications after coronary artery bypass grafting. Medicina (Kaunas, Lithuania). 2002;38 Suppl 2:247-250	not in english
Bi Q, Li Q, Zhang ZQ, Gu CX, Ma XH. A study of neurological complications after coronary artery bypass grafting. Zhonghua nei ke za zhi [Chinese journal of internal medicine]. 2008;47:202-205	not in english
Boning A, Bein B, Dircksen C, Carstensen S, Friedrich C, Hedderich J, Cremer J. The use of a sutureless anastomotic system for aortic vein graft anastomoses does not lead to an improvement in neuropsychological outcome. Zeitschrift fur Herz-, Thorax- und Gefasschirurgie. 2004;18:281-289	not in english
Caldas J, Panerai RB, Robinson TG, Camara L, Ferreira G, Borg-Seng-Shu E, De Lima Oliveira M, Mian NC, Santos L, Nogueira R, et al. Impaired dynamic cerebral autoregulation after coronary artery bypass grafting and association with postoperative delirium. Critical Care. 2016;20:no pagination	not in english
Cao L, Li Q, Bi Q. [neurologic injury after off-pump coronary artery bypass grafting in elder patients with a history of cerebral infarction]. Zhonghua nei ke za zhi [Chinese journal of internal medicine]. 2011;50:201-204	not in english
Chernov VI, Efimova NI, Efimova II, Akhmedov SD, Lishmanov IB. Central hemodynamic and cognitive function in the short- and long-term periods after coronary bypass grafting. Angiologiia i sosudistaia khirurgiia = Angiology and vascular surgery. 2004;10:114-124	not in english
Chernov VI, Efimova NI, Efimova II, Akhmedov SD, Lishmanov IB. Scintigraphic evaluation of cerebral circulation in prognosis of cerebrovascular complications of coronary artery bypass grafting. Klinicheskaia Meditsina. 2009;87:33-37	not in english
Chugunova YV, Chumakova GA, Ermolin PA, Baranov AS. The influence of visceral obesity on cognition in patients after coronary artery bypass surgery. Russian Journal of Cardiology. 2016;132:19-24	not in english
Colak A, Arar C, Turan E, Soker A, Kargi M, Gunday I, Turan N. The effect of normal and low hematocrit levels on cognitive functions during cardiopulmonary bypass. Gogus-Kalp-Damar Anestezi ve Yogun Bakim Dernegi Dergisi. 2008;14:119-125	not in english
Cui QT, Fu QL, Han PL, Zhang J. Risk factors of cognitive impairment after off-pump coronary artery bypass grafting. Chinese Journal of Cardiology. 2012;40:104-107	not in english
Durmus M, Karaaslan K, But AK, Toprak HI, Teksan H, Ersoy MO. The effects of acute normovolemic hemodilution on postoperative cognitive functions in coronary artery bypass surgery. Gogus-Kalp-Damar Anestezi ve Yogun Bakim Dernegi Dergisi. 2003;9:119-123	not in english
Efimova N, Chernov V, Efimova I, Akhmedov S, Lishmanov Y. [changes in cerebral blood flow and cognitive function in patients undergoing coronary bypass surgery with cardiopulmonary bypass]. Kardiologiia. 2015;55:40-46	not in english

Efimova NI, Chernov VI, Efimova II, Akhmedov SD, Podoksenov IK, Lishmanov IB. Pharmacological prevention of cerebrovascular complications in cardiac patients after coronary artery bypass surgery. Zhurnal nevrologii i psikhiatrii imeni S.S. Korsakova / Ministerstvo zdravookhraneniia i meditsinskoi promyshlennosti Rossiiskoi Federatsii, Vserossiiskoe obshchestvo nevrologov [i] Vserossiiskoe obshchestvo psikhiatrov. 2003:58-63	not in english
Ge Y, Ma Z, Shi H, Zhao Y, Gu X, Wei H. [incidence and risk factors of postoperative cognitive dysfunction in patients underwent coronary artery bypass grafting surgery]. Zhong Nan da Xue Xue Bao. Yi Xue Ban = Journal of Central South University. Medical Sciences. 2014;39:1049-1055	not in english
Ge Y, Shi H, Zhu W, Ma ZL, Wei H, Gu X, Bao H. Effects of ulinastatin on cognitive function in patients with coronary artery bypass grafting. Zhejiang da xue xue bao. Yi xue ban = Journal of Zhejiang University. Medical sciences. 2015;44:532-538	not in english
Goksu S, Kocoglu H, Daglar B, Ustunsoy H, Celkan MA, Kazaz H, Uyar A, Oner U. Early postoperative period in coronary revascularization: Comparison of on-pump and off-pump techniques. Gogus-Kalp-Damar Anestezi ve Yogun Bakim Dernegi Dergisi. 2003;9:162-166	not in english
Gorna R, Kiejna A. Assessment of short-term neuropsychologic changes after normothermic versus hypothermic coronary artery bypass grafting. Archives of Psychiatry and Psychotherapy. 2003;5:31-43	not in english
Gorna R, Kustrzycki W, Kiejna A, Rymaszewska J. Assessment of short-term neuropsychologic changes after normothermic versus hypothermic coronary artery bypass grafting. Psychiatria Polska. 2001;35:781-795	not in english
Goruleva MV, Ganenko OS, Kovaltcova RS, Kutuzova A, Petrova NN, Demchemnko EA, Nedoshivin AO. Quality of life and psycho-cognitive condition in patients after coronary artery bypass graft surgery. Russian Journal of Cardiology. 2014;113:68-71	not in english
Hamada H, Kuroda M, Nakanuno R, Kawamoto M. Changes in serum s-100 beta protein and mini-mental state examination in patients undergoing coronary artery bypass grafting or valve replacement surgery. Anesthesia and Resuscitation. 2007;43:47-50	not in english
Hamada H, Kuroda M, Nakanuno R, Kawamoto M. Orbital ultrasound monitoring during cardiopulmonary bypass - relevance to postoperative neurocognitive outcome. Anesthesia and Resuscitation. 2007;43:67-71	not in english
Ieva N, Samalavicius R, Misiuriene I, Valaikiene J, Baublys A, Uzdavinys G. [disorders of neurocognitive function after coronary artery bypass grafting]. Pooperacines neurokognityvines disfunkcijos daznis po miokardo revaskulizavimo operaciju. 2004;40 Suppl 1:66-69	not in english
Jensen BO, Hughes P, Rasmussen LS, Pedersen PU, Steinbruchel DA. Cognitive functions in elderly high-risk patients after off-pump coronary artery bypass grafting versus conventional bypass grafting - a randomised study - secondary publication. Ugeskrift for Laeger. 2006;168:3820-3822	not in english
Kamenskaya OV, Klinkova AS, Meshkov IO, Lomivorotov VV, Cherniavsky AM. Predictors of cardiorespiratory complications in patients with ischemic heart disease after coronary artery bypass grafting. [russian]. Kardiologiia. 2017;57:5-9	not in english
Kertsman VP, Kuperberg EB, Mergasov AG, Sonkin VG, Soboleva ER, Shvarts IP, Botnar IM. The risk of neurological complications following aortocoronary bypass in patients with multifocal atherosclerosis. Anesteziologiia i reanimatologiia. 1991:19-22	not in english
Koc M, Unver S, Aydinli B, Yildirim Guclu C, Kazanci D, Balaban F, Ozgok A. The effects of three different anesthetic techniques on cerebral oxygenation and postoperative neurocognitive function in heart surgery. Gogus-Kalp-Damar Anestezi ve Yogun Bakim Dernegi Dergisi. 2014;20:77-84	not in english
Kolesnykov V, Loskutov O, Druzhyna O, Maruniak S, Todurov B. Features of cerebral blood flow dynamics in patients with arterial hypertension during cardiac surgery. [russian]. Georgian Medical News. 2019:13-19	not in english
Krenkel GL. Structure and dynamics of mental disorders in patients before heart surgery and in the early postoperative period. Zhurnal nevrologii i psikhiatrii imeni S.S. Korsakova / Ministerstvo zdravookhraneniia i meditsinskoi promyshlennosti Rossiiskoi Federatsii, Vserossiiskoe obshchestvo nevrologov [i] Vserossiiskoe obshchestvo psikhiatrov. 2007;107:18-25	not in english
Kurnaz P, Sungur Z, Camci E, Sivrikoz N, Orhun G, Senturk M, Sayin O, Tireli E, Gurvit H. The effect of two different glycemic management protocols on postoperative cognitive dysfunction in coronary artery bypass surgery. Brazilian Journal of Anesthesiology. 2016:no pagination	not in english
Kurnaz P, Sungur Z, Camci E, Sivrikoz N, Orhun G, Senturk M, Sayin O, Tireli E, Gurvit H. The effect of two different glycemic management protocols on postoperative cognitive dysfunction in coronary artery bypass surgery. Revista Brasileira de Anestesiologia. 2017;67:258-265	not in english

Li J-y, Bi Q. [the influence of carotid artery stenosis on neurological outcomes in patients undergoing off-pump coronary artery bypass grafting]. Zhonghua nei ke za zhi. 2012;51:687-689	not in english
Li X, Ma W, Jiang J, Yuan P, Bi Q. Morbidity and related risk factors of postoperative delirium in the patients undergoing coronary artery bypass grafting. Chinese Journal of Neurology. 2015;48:1069-1073	not in english
Malinovskii NN, Beliaev AA, Khomskaia ED, Oleinikova EN. Analysis of mental functions in patients after coronary bypass surgery. Khirurgiia. 2000:17-22	not in english
Mansoori M, Boroumand AB. The effect of zolpidem or promethazine premedication on agitation, sedation and cognitive disorders before and after coronary artery bypass graft surgery. Journal of Isfahan Medical School. 2015;33:no pagination	not in english
Mansouri M, Masoumi SGR, Izadi E. The preventive effect of donepezil on post-operative cognitive disorder in patients undergoing coronary artery bypass graft (cabg) surgery. [persian]. Journal of Isfahan Medical School. 2017;35:228-234	not in english
Matsumiya G, Ohtake S, Sawa Y, Takahashi T, Nishimura M, Kagizaki K, Katsura T, Matsue H, Matsuda H. [is routine application of off-pump coronary artery bypass grafting warranted?]. Kyobu geka. The Japanese journal of thoracic surgery. 2001;54:315-320	not in english
Mortasawi A, Rosendahl U, Schroder T, Albert A, Ennker IC, Ennker J. Isolated coronary artery bypass grafting in the 9th decade of life. Zeitschrift fur Gerontologie und Geriatrie. 2000;33:381-387	not in english
Mu D-l, Wang D-x, Li L-h, Shan G-j, Su Y, Yu Q-j, Shi C-x. [postoperative delirium is associated with cognitive dysfunction one week after coronary artery bypass grafting surgery]. Beijing da xue xue bao. Yi xue ban = Journal of Peking University. Health sciences. 2011;43:242-249	not in english
Mullges W, Misoph M, Berg D, Hickethier T, Babin-Ebell J. Aprotinin and cognitive performance after coronary artery bypass surgery. Zeitschrift fur Herz-, Thorax- und Gefasschirurgie. 2001;15:29-34	not in english
Ohnishi Y, Uchida O, Hayashi Y, Kuro M, Sugimoto K, Kuriyama Y. [relationship between retained microbubbles and neuropsychologic alterations after cardiac operation]. Masui. The Japanese journal of anesthesiology. 1995;44:1327-1333	not in english
Ovchinnikov DA, Amosov DD, Vorobyov EA, Garnyuk VV, Beltiukov PP, Grebennik VK, Gordeev ML, Barantsevich ER. [cognitive dysfunction and content of inflammatory markers in patients after coronary artery bypass graft]. Zhurnal Nevrologii i Psikhiatrii Imeni S.S. Korsakova. 2017;117:5-10	not in english
Ozturk T, Kocan AA, Yildirim F, Alp D, Kurdal T. The effect of beta-blocking agents on morbidity und mortality following coronary artery bypass surgery. Gogus-Kalp-Damar Anestezi ve Yogun Bakim Dernegi Dergisi. 2013;19:80-85	not in english
Petrova MM, Prokopenko SV, Eremina OV, Matjushin GV, Sakovich VA, Drobot DB, Mozhejko EY, Kaskaeva DS. No title. Rational Pharmacotherapy in Cardiology. 2015;11:391-397	not in english
Petrova MM, Prokopenko SV, Eremina OV, Mozheiko EY, Kaskaeva DS, Gankin MI. Cerebral circulation and cognitive status of coronary heart disease patients after bypass surgery. [russian]. Russian Journal of Cardiology. 2017;149:34-41	not in english
Petrova MM, Prokopenko SV, Eremina OV, Mozhejko EY, Kaskaeva DS. Correction of postoperative cognitive dysfunction in cardiosurgery using computer-based stimulation programs. Zhurnal Nevrologii i Psihiatrii imeni S.S. Korsakova. 2016;116:35-41	not in english
Petrova MM, Prokopenko SV, Eremina OV, Mozheyko EY, Kaskaeva DS, Gankin MI. Cerebral circulation and cognition in patients with coronary heart disease underwent coronary bypass operation. [russian]. Russian Journal of Cardiology. 2017;143:77-84	not in english
Petrova MM, Shprakh VV, Kaskaeva DS, Eremina OV, Narkevich AN, Eremina SS. Prognostic methods of postoperative cognitive dysfunction in patients with ischemic heart disease after coronary bypass surgery under extracorporeal circulation. [russian]. Zhurnal nevrologii i psikhiatrii imeni S.S. 2018;Korsakova. 118:81-86	not in english
Pfeiffer T, Siepe M, Benk C, Gieringer A, Zemann S, Schlensak C, Beyersdorf F. Influence of a changed perfusion management with increased perfusion pressure in heart-lung machine on the postoperative neuropsychological capabilities of the patient. Kardiotechnik. 2011;20:3-7	not in english
Polushin AY, Yanishevskiy SN, Maslevtsov DV, Krivov VO, Beskrovnaya OV, Molchan NS. The efficacy of prevention of postoperative cognitive dysfunction in cardiac surgeries with the use of the cerebrolysin. [russian]. Zhurnal Nevrologii i Psihiatrii imeni S.S. 2017;Korsakova. 117:37-45	not in english

Rodig G, Taeger K. Memory function in the early postoperative period after cardiac surgery - impact of the anaesthetic procedure and comparison with memory function after vascular surgery. Anasthesiologie und Intensivmedizin. 2002;43:431-455	not in english
Rothenhausler HB, Stepan A, Hetterle R, Trantina-Yates A. The effects of coronary artery bypass graft surgery on health-related quality of life, cognitive performance, and emotional status outcomes: A prospective 6-month follow-up consultation-liaison psychiatry study. Fortschritte der Neurologie Psychiatrie. 2010;78:343-354	not in english
Sezer O, Karlidag R, Karabulut AB, Ozcan C, Nisanoglu V, Turkoz Y, But A, Unal S. Relationship between nitric oxide levels and delirium in patients with coronary bypass operation. Klinik Psikofarmakoloji Bulteni. 2004;14:185-190	not in english
Solodukhin AV, Bezzubova VA, Kuhareva IN, Inozemtseva AA, Seryy AV, Yanitskiy MS, Trubnikova OA, Barbarash OL. The relationship between psychological characteristics of the attitude to the disease, coping behavior of patients with ischemic heart disease, and their cognitive status. RUDN Journal of Psychology and Pedagogics. 2017;14:178-189	not in english
Stevens R, Gersbach P, Ruchat P, Hurni M, Stumpe F, Fischer A, Sadeghi H. [cardiac surgery in octogenarians]. Chirurgie cardiaque chez l'octogenaire. 1995;125:2084-2089	not in english
Sugimoto K, Ohata A, Terada H, Kuriyama Y. Changes in neuropsychological functions following cardiovascular surgery. Clinical Neurology. 1995;35:606-610	not in english
Thiel A, Zimmer M, Stertmann WA, Kaps M, Hempelmann G. Microembolisations during cardiac surgery under extracorporeal circulation. Anasthesiologie Intensivmedizin Notfallmedizin Schmerztherapie. 1997;32:715-720	not in english
Trubnikova O, Mamontova A, Maleva O, Kuhareva I, Barbarash O. Factors determining the development of long-term postoperative cognitive dysfunction in patients with type 2 diabetes undergoing coronary artery bypass grafting. European Journal of Preventive Cardiology. 2015;22:S189	not in english
Trubnikova OA, Maleva OV, Tarasova IV, Mamontova AS, Uchasova EG, Barbarash OL. Effect of statins on development of early cognitive dysfunction after coronary artery bypass grafting. Kardiologiia. 2015;55:49-56	not in english
Trubnikova OA, Mamontova AS, Maleva OV, Tarasova IV, Kukhareva IN, Kuzmina AA, Kagan ES, Barbarash OL. Predictors of persistant post-operation cognitive dysfunction in 2 type diabetes patients after coronary bypass grafting. Russian Journal of Cardiology. 2016;130:12-18	not in english
Trubnikova OA, Mamontova AS, Syrova ID, Kukhareva IN, Maleva OV, Barbarash OL. The cognitive status of patients with type 2 diabetes mellitus after coronary bypass surgery. Klinicheskaia Meditsina. 2015;93:39-44	not in english
Trubnikova OA, Mamontova AS, Tarasova IV, Maleva OV, Kuz'mina AA, Barbarash OL. Association of cognitive impairments with carbohydrate and lipid metabolic parameters in type 2 diabetes mellitus patients undergoing coronary bypass surgery. Terapevticheskii Arkhiv. 2015;87:69-75	not in english
Trubnikova OA, Tarasova IV, Mamontova AS, Syrova ID, Maleva OV, Barbarash OL. A role of carotid stenoses in the structure of early postoperative cognitive dysfunction in patients underwent coronary artery bypass grafting. Zhurnal Nevrologii i Psikhiatrii Imeni S.S. Korsakova. 2014;114:36-42	not in english
Trubnikova OA, Tarasova IV, Mamontova AS, Syrova ID, Maleva OV, Barbarash OL. Structure of cognitive disorders and dynamics of bioelectric activity of the brain in patients after direct myocardial revascularization. Russian Journal of Cardiology. 2014;112:57-62	not in english
Trubnikova OA, Tarasova IV, Syrova ID, Mamontova AS, Kovalenko AV, Barbarash OL, Barbarash LS. Neuropsychological status of patients with low and moderate carotid artery stenoses after the coronary artery bypass surgery. Zhurnal Nevrologii i Psihiatrii imeni S.S. Korsakova. 2013;113:28-33	not in english
Tsygan NV, Odinak MM, Khubulava GG, Tsygan VN, Peleshok AS, Andreev RV, Kurasov ES, Litvinenko IV. [postoperative cerebral dysfunction]. Zhurnal Nevrologii i Psikhiatrii Imeni S.S. Korsakova. 2017;117:34-39	not in english
Valentini M, Spezzaferri R, Brambilla G, Tavanelli M, Sangiuliano M, Majorino G, Racca V, Ferratini M. Complexities of psychological disorders observable after surgical myocardial revascularization in male subjects. Italian Heart Journal Supplement. 2005;6:375-381	not in english
Wos S, Opala G, Jasinski M, Janas P, Bachowski R, Kus H, Domaradzki W, Gemel M, Deja M, Dyaczynska-Herman A. The incidence of early central nervous system complications following cardiac surgery with cardiopulmonary bypass. Kardiologia Polska. 1997;47:115-119	not in english

Xu BL, Bi Q, Chen MY, Luo D. Ct cerebral perfusion parameters in prediction of postoperative cognition disorders of off-pump coronary artery bypass grafting. Chinese Journal of Interventional Imaging and Therapy. 2015;12:298-302	not in english
Xu D, Liu F, Hua Y, Zhang K-f, Liu Y-h, Shang X-b, Li H-l, Yao Q, Li X-f, Zhang R, et al. Increasing cardiopulmonary bypass flow volume improves outcome of patient with carotid stenosis undergoing coronary artery bypass grafting. Zhonghua wai ke za zhi [Chinese journal of surgery]. 2009;47:577-579	not in english
Yilmaz E, Aksun M, Girgin S, Gulseren S, Kuru V, Sencan A, Koroglu L, Aran G, Karahan N. The comparison of the effects of the off-pump and cardiopulmonary by-pass techniques upon postoperative delirium at elective coronary by-pass greft surgery. Gogus-Kalp-Damar Anestezi ve Yogun Bakim Dernegi Dergisi. 2013;19:67-75	not in english
Yilmaz M, Aydin U, Kilic Yilmaz V, Yavuz Y, Denizalti TB, Canik S. The effect of magnesium on neurocognitive functions after cardiopulmonary by-pass surgery. Turkiye Klinikleri Cardiovascular Sciences. 2014;26:105-110	not in english
Yin Y-q, Luo A-l, Guo X-y, Li L-h, Ren H-z, Ye T-h, Huang Y-g. Perioperative cortisol circadian secretion and neuropsychological states in patients undergoing coronary artery bypass grafting surgery. Zhonghua wai ke za zhi [Chinese journal of surgery]. 2005;43:463-467	not in english
Zhang Y, Cheng H, Xu C, Bao H, Shi H, Ge Y, Wei H. [effects of ultrasound-guided stellate ganglion block on cerebral oxygen metabolism and postoperative cognitive dysfunction in the elderly]. Sheng wu yi xue gong cheng xue za zhi = Journal of biomedical engineering = Shengwu yixue gongchengxue zazhi. 2014;31:1107-1110	not in english
Zhang Y, Qian Y, Bao H, Shi H, Zhou J. Effect of stellate ganglion block on bilateral regional cerebral oxygen saturation and postoperative cognitive function. Sheng wu yi xue gong cheng xue za zhi = Journal of biomedical engineering = Shengwu yixue gongchengxue zazhi. 2016;33:132-135	not in english
Zhao YH, Wen DX. Analysis of related factors of postoperative cognitive dysfunction for patients undergoing off-pump coronary artery bypass graft surgery. Journal of Shanghai Jiaotong University (Medical Science). 2016;36:100-104	not in english
Zoll A, Degirmenci U, Bleich S, Richter-Schmidinger T, Kornhuber J, Fischlein T, Weih M. Neuropsychological complications after coronary bypass grafting. Fortschritte der Neurologie-Psychiatrie. 2009;77:97-101	not in english
Ernest CS, Elliott PC, Murphy BM, Le Grande MR, Goble AJ, Higgins RO, Worcester MUC, Tatoulis J. Predictors of cognitive function in candidates for coronary artery bypass graft surgery. Journal of the International Neuropsychological Society : JINS. 2007;13:257-266	only baseline data
Ernest CS, Murphy BM, Worcester MUC, Higgins RO, Elliott PC, Goble AJ, Le Grande MR, Genardini N, Tatoulis J. Cognitive function in candidates for coronary artery bypass graft surgery. The Annals of thoracic surgery. 2006;82:812-818	only baseline data
Hudetz JA, Patterson KM, Pagel PS. Comparison of pre-existing cognitive impairment, amnesic mild cognitive impairment, and multiple domain mild cognitive impairment in men scheduled for coronary artery surgery. European Journal of Anaesthesiology. 2012;29:320-325	only baseline data
Tsushima WT, Johnson DB, Lee JD, Matsukawa JM, Fast KMS. Depression, anxiety and neuropsychological test scores of candidates for coronary artery bypass graft surgery. Archives of clinical neuropsychology : the official journal of the National Academy of Neuropsychologists. 2005;20:667-673	only baseline data
Aldea GS, O'Gara P, Shapira OM, Treanor P, Osman A, Patalis E, Arkin C, Diamond R, Babikian V, Lazar HL, et al. Effect of anticoagulation protocol on outcome in patients undergoing cabg with heparin-bonded cardiopulmonary bypass circuits. The Annals of thoracic surgery. 1998;65:425-433	other
Barry SJE, Zeger SL, Selnes OA, Grega MA, Borowicz LM, Jr., McKhann GM. Quantitative methods for tracking cognitive change 3 years after coronary artery bypass surgery. The Annals of thoracic surgery. 2005;79:1104-1109	other
Bhamidipati D, Goldhammer JE, Sperling MR, Torjman MC, McCarey MM, Whellan DJ. Cognitive outcomes after coronary artery bypass grafting. Journal of Cardiothoracic & Vascular Anesthesia. 2017;31:707-718	other
Cockburn J, Hildick-Smith D, Trivedi U, De Belder A. Coronary revascularisation in the elderly. Heart. 2017;103:316-324	other
Indja B, Fanning JP, Maller JJ, Fraser JF, Bannon PG, Vallely M, Grieve SM. Neural network imaging to characterize brain injury in cardiac procedures: The emerging utility of connectomics. British Journal of Anaesthesia. 2017;118:680-688	other

Lamy A, Devereaux PJ, Prabhakaran D, Hu S, Piegas LS, Straka Z, Paolasso E, Taggart D, Lanas F, Akar AR, et al. Rationale and design of the coronary artery bypass grafting surgery off or on pump revascularization study: A large international randomized trial in cardiac surgery. American Heart Journal. 2012;163:1-6	other
Wu M, Liang Y, Dai Z, Wang S. Perioperative dexmedetomidine reduces delirium after cardiac surgery: A meta-analysis of randomized controlled trials. Journal of Clinical Anesthesia. 2018;50:33-42	other
Yuan SM, Lin H. Postoperative cognitive dysfunction after coronary artery bypass grafting. Brazilian Journal of Cardiovascular Surgery. 2019;34:76-84	other
Maggio M, Nicolini F, Cattabiani C, Beghi C, Gherli T, Schwartz RS, Valenti G, Ceda GP. Effects of testosterone supplementation on clinical and rehabilitative outcomes in older men undergoing on-pump cabg. Contemporary Clinical Trials. 2012;33:730-738	protocol paper
Uva MS, Matias F, Cavaco S, Magalhaes MP. Rationale, design and methodology for a prospective randomized study of graft patency in off-pump and on-pump multi-vessel coronary artery bypass surgery (promiss) using multidetector computed tomography. Trials. 2008;9:44	protocol paper
Whitlock R, Teoh K, Vincent J, Devereaux PJ, Lamy A, Paparella D, Zuo Y, Sessler DI, Shah P, Villar JC, et al. Rationale and design of the steroids in cardiac surgery trial. American Heart Journal. 2014;167:660-665	protocol paper

Table S6. **Demographic data, presence in statistical analyses and quality assessment scores for included studies within risk and protective factor meta-analyses for delirium and cognitive-decline post CABG.**

Ref. No.	Lead Author, Year	Total No. of Patients	Total No. of Males	Mean/Median Age	SD/Range/IQR of Age	Cognitive Decline C1	C2	C3	Del	QA (/12)
51	Al Tmimi, 2016	92	78	67	R: 46-86	-	-	-	x	12
52	Baba, 2007	218	152	71.25	5.5	x	-	-	-	10
53	Boodhwani, 2006*	448	390	68.3	0.4	x	-	-	-	12
54	Braekken, 1998	14	14	N/A	N/A	-	x	-	-	7
55	Bucerius, 2005	9682	7500	N/A	N/A	-	-	-	x	6
56	Caldas 2019	67	51	64.3	9.5	-	-	-	x	12
57	Chen, 2017	136	104	60.85	7.76	-	-	-	x	10
58	Christiansen, 2016	8	7	63.38	10.69	x	-	-	-	7
59	Coffey, 1983	1669	1384	52.15	SEM: 8	-	-	-	x	7
60	Colak, 2015	190	148	62.66	7.96	x	-	-	-	7
61	Cumurcu, 2008	50	37	59.62	10.66	-	-	-	x	9

Ref. No.	Lead Author, Year	Total No. of Patients	Total No. of Males	Mean/Median Age	SD/Range/IQR of Age	Cognitive Decline C1	C2	C3	Del	QA (/12)
62	deTournay-Jette, 2011	61	51	70.39	4.69	x	x	-	-	10
63	Dieleman, 2009	281	192	61.3	9	-	x	x	-	10
64	Djaiani, 2003	417	293	60.34	10	-	x	-	-	9
65	Dong, 2014	108	83	63	7.9	x	-	-	x	10
66	Eriksson, 2002	52	40	70.27	5.53	-	-	-	x	12
67	Goto, 2000	177	117	70.26	4.99	x	-	-	-	11
68	Gottesman, 2010	5052	3682	63.92	N/A	-	-	-	x	7
69	Hall, 1999	35	27	65.9	9.1	x	-	-	-	12
70	Harmon, 2004†	35	28	61.7	7.51	x	x	-	-	9
71	Harmon, 2005†	36	30	64.07	N/A	x	-	-	-	12
72	Humphreys, 2016	173	148	63.47	10.1	-	-	-	x	7
73	Kadoi, 2001‡	185	138	N/A	N/A	x	x	-	-	6
74	Kadoi, 2002§	60	53	62.75	8.5	-	x	-	-	9

Ref. No.	Lead Author, Year	Total No. of Patients	Total No. of Males	Mean/Median Age	SD/Range/IQR of Age	Cognitive Decline C1	C2	C3	Del	QA (/12)
[75]	Kadoi, 2003[‡]	180	136	65	9	-	x	-	-	9
[76]	Kadoi, 2005[§]	280	210	65.07	9.93	x	x	-	-	9
[77]	Kadoi, 2007[§]	106	53	62.55	10.45	-	x	-	-	9
[78]	Kadoi, 2011 (a)[§]	124	89	61.29	5.39	x	-	-	-	12
[79]	Kadoi, 2011 (b)[§]	90	68	65	9	x	x	-	-	9
[80]	Kazmierski, 2014 (a)[|¶]	113	90	64	R: 59-71	-	-	-	x	12
[81]	Kazmierski, 2014 (b)[¶]	102	N/A	N/A	N/A	-	-	-	x	12
[82]	Kazmierski, 2014 (c) [|¶]	113	90	Med: 64	R: 59-71	-	-	-	x	12
[83]	Khan, 2014	735	577	55.64	9.65	-	-	-	x	10
[84]	Khatri, 1999	170	127	61	10	-	x	-	-	7
[85]	Kok, 2017	57	N/A	N/A	N/A	-	-	x	-	5
[86]	Kumpaitiene, 2019	59	34	66.49	8.04	x	-	-	-	11
[87]	Lachmann, 2018	252	180	61.0	9.1	-	-	x	-	8

Ref. No.	Lead Author, Year	Total No. of Patients	Total No. of Males	Mean/Median Age	SD/Range/IQR of Age	Cognitive Decline C1	C2	C3	Del	QA (/12)
88	Leenders, 2018	357	304	66.20	8.84	-	-	-	x	9
89	Li, 2015	38	34	62.4	11.8	-	-	-	x	10
90	Liu, 2009	227	209	60	8	x	x	-	-	9
91	Loponen, 2008	300	237	66.17	8.89	-	-	-	x	8
92	Mardani, 2012	196	183	61.84	11.83	-	-	-	x	9
93	Mariscalco, 2012	4079	3220	67.8	9.2	-	-	-	x	12
94	Martin, 2010#	14262	10912	N/A	N/A	-	-	-	x	5
95	Martin, 2012#	8474	6391	N/A	N/A	-	-	-	x	7
96	Mathew, 2006**	121	N/A	N/A	N/A	-	x	-	-	8
97	Mathew, 2007**	677	471	61.7	10.5	-	x	-	-	9
98	Miyazaki, 2011	768	N/A	N/A	N/A	-	-	-	x	6
99	Mu, 2010	243	200	61	8.3	-	-	-	x	12
100	Mu, 2013	166	141	60	8.9	x	-	-	-	12

Ref. No.	Lead Author, Year	Total No. of Patients	Total No. of Males	Mean/Median Age	SD/Range/IQR of Age	Cognitive Decline C1	C2	C3	Del	QA (/12)
101	Newman, 1987	67	62	55.0	7.8	x	-	-	-	10
102	Nikolic, 2012	370	271	N/A	N/A	-	-	-	x	7
103	Norkiene, 2007	1367	1035	64.98	9.14	-	-	-	x	10
104	Norkiene, 2011	127	103	60.91	7.24	x	-	-	-	11
105	Oh, 2008	46	36	63	5.5	x	-	-	-	11
106	Oh, 2017	292	211	N/A	N/A	-	-	-	x	10
107	Oldham, 2015	102	76	65.1	9	-	-	-	x	11
108	Oldham, 2019	131	96	65.8	9.2	-	-	-	x	12
109	Omiya, 2015	88	N/A	69	7	-	-	-	x	10
110	Otomo, 2013	153	109	72	7	-	-	-	x	12
111	Palmbergen, 2012	642	452	68.5	9.79	-	-	-	x	11
112	Plaschke, 2010	114	89	68.98	8.39	-	-	-	x	12
113	Reents, 2002	47	41	60.4	8	x	-	-	-	10

Ref. No.	Lead Author, Year	Total No. of Patients	Total No. of Males	Mean/Median Age	SD/Range/IQR of Age	Cognitive Decline C1	C2	C3	Del	QA (/12)
114	Restrepo, 2002	13	10	65	9	x	-	-	-	10
115	Ringaitiene, 2015	99	70	67.6	7.78	-	-	-	x	10
116	Robson, 2000	124	N/A	59.44	9.25	-	x	-	-	7
117	Rodriguez, 2010	356	325	63	9	x	x	-	-	5
118	Rolfson, 1999 (a)††	75	59	N/A	N/A	-	-	-	x	12
119	Rolfson, 1999 (b)††	71	57	71	N/A	-	-	-	x	11
120	Royse, 2000	47	37	64.22	1.78	x	x	-	-	8
121	Royse, 2011	180	153	62.79	10.5	-	-	-	x	10
122	Rudolph, 2005	36	36	68.8	9.2	-	-	-	x	12
123	Rudolph, 2006‡‡	80	62	74.9	6.2	-	-	-	x	11
124	Rudolph, 2009‡‡	68	67	70.7	8.2	-	-	-	x	12
125	Sahan, 2018	40	34	65.85	6.02	x	x	-	-	9
6	Santos, 2004	220	142	70.71	5.48	-	-	-	x	11

Ref. No.	Lead Author, Year	Total No. of Patients	Total No. of Males	Mean/Median Age	SD/Range/IQR of Age	Cognitive Decline C1	C2	C3	Del	QA (/12)
126	Scott, 2002	103	84	64.77	1.3	x	-	-	-	12
127	Sevuk, 2015	200	128	70.65	3.95	-	-	-	x	12
128	Siepe, 2011	92	74	66.87	8.98	-	-	-	x	12
129	Silbert, 2006[§§]	326	252	67.9	7.6	x	x	-	-	9
130	Silbert, 2008[§§]	264	203	67.8	7.7	x	-	x	-	7
131	Slater, 2009	240	201	64.74	9.96	x	x	-	-	7
132	Smith, 1986	55	51	54.7	R: 37-74	x	-	-	-	9
133	Smith, 2000	381	308	N/A	N/A	-	x	-	-	8
134	Stump, 1996	167	138	61	10	x	-	-	-	8
135	Subramaniam, 2019	120	101	Med: 69	IQR: 63-76	-	-	-	x	12
136	Suksompong, 2002	110	110	61.95	7.58	x	-	-	-	10
137	Swaminathan, 2002	282	201	61	10.44	-	x	-	-	8
138	Sylivris, 1998	41	31	69.8	6.9	x	-	-	-	10

						Cognitive Decline				
**Ref. No.**	**Lead Author, Year**	**Total No. of Patients**	**Total No. of Males**	**Mean/Median Age**	**SD/Range/IQR of Age**	**C1**	**C2**	**C3**	**Del**	**QA (/12)**
139	Tagarakis, 2007	137	99	69.55	7.63	-	-	-	x	7
140	Tamura, 2019	88	76	69.3	2.5	-	-	-	x	10
141	Toeg, 2013[*]	652	576	64.37	9	x	-	-	-	10
142	Trubnikova, 2014	101	101	56.6	5.85	x	-	x	-	4
143	Tully, 2010	158	125	64.68	10.56	-	-	-	x	10
144	van Dijk, 2004	281	191	61.2	9.0	-	x	-	-	7
145	Yilmaz, 2016	137	105	61.02	7.83	-	-	-	x	10
146	Zhang, 2015	249	197	62.9	9.34	-	-	-	x	12

[†, ‡, §, ¶, ‡‡] Suspected overlap of samples; [*, |, #, **, ††, §§] Known overlap of sample

Ref No. = supplementary reference list number; C1= acute cognitive decline (immediately post-operatively up to 1-month); C2= mid-term cognitive decline (1 to 6-months post-operatively); C3= long-term cognitive decline (12 to 15-months post-operatively). Del = delirium; Med = median; IQR= inter quartile range; QA = quality assessment; SD = standard deviation.

Table S7. **Subgroup meta-analyses of diagnostic tool, for pre, intra and post-operative variables for the development of delirium following CABG.**

Variable	Delirium Diagnosis	Pooled Estimate				Heterogeneity	
		k (n)	OR/MD*/ SMD†	95%CI	p value	I^2	Tau^2
**Pre-Operative (Categorical)**							
Alcoholism	No Tool	Insufficient Data					
	Tool	5 (694)	0.77	0.46—1.29	.317	0	0
Arrhythmia, incl. AF	No Tool	7 (31550)	2.05	1.77—2.37	<.001	0	0
	Tool	8 (1252)	1.91	1.15—3.16	<.001	45.09	0.21
BMI >28 (including >30)	No Tool	2 (15629)	0.86	0.51—1.47	.587	62.68	0.10
	Tool	5 (668)	1.46	0.89—2.41	.133	11.02	0.04
BMI ≥30 only	No Tool	Insufficient Data					
	Tool	4 (419)	1.85	1.09—3.14	.023	0.00	0
Cognitive Impairment	No Tool	Insufficient Data					
	Tool	6 (790)	4.11	2.59—6.53	<.001	0	0
Depression	No Tool	2 (378)	2.06	0.75—5.67	.162	66.06	0.35
	Tool	2 (202)	4.14	1.37—12.51	.012	0	0
Diabetes	No Tool	12 (42736)	1.46	1.33—1.60	<.001	13.34	0
	Tool	18 (5419)	1.57	1.32—1.87	<.001	0	0
Dyslipidaemia/Hyperlipidaemia	No Tool	4 (2283)	0.70	.033—1.49	.355	66.26	0.39
	Tool	9 (4166)	0.99	0.67—1.45	.943	46.78	0.14
Education>12years/high school	No Tool	Insufficient Data					
	Tool	3 (347)	0.78	0.45—1.35	.374	0	0
Hypertension	No Tool	11 (33054)	1.65	1.38—1.98	<.001	50.98	0.04
	Tool	16 (5308)	1.18	0.88—1.57	.267	38.51	0.12
Sex (male)	No Tool	10 (30814)	1.10	0.87—1.40	.415	62.44	0.06
	Tool	25 (6639)	0.78	0.60—1.01	.056	41.95	0.16
Kidney injury	No Tool	6 (23602)	1.91	1.40—2.60	<.001	36.40	0.05

Variable	Delirium Diagnosis	Pooled Estimate k (n)	OR/MD*/ SMD†	95%CI	p value	Heterogeneity I^2	Tau^2
	Tool	8 (1662)	1.96	1.18—3.25	.009	29.13	0.14
Previous MI <30 days	No Tool	3 (101)	1.98	0.88—4.49	.100	52.09	0.27
	Tool	2 (200)	1.04	0.56—1.93	.909	0	0
Previous MI history/ever	No Tool	3 (6877)	1.04	0.72—1.51	.822	45.06	0.05
	Tool	8 (3785)	1.24	0.92—1.67	.160	0	0
Previous stroke, TIA, CVA	No Tool	6 (22297)	2.73	1.92—3.88	<.001	56.18	0.09
	Tool	9 (4830)	2.37	1.50—3.70	<.001	35.96	0.16
PVD	No Tool	4 (11604)	2.11	1.73—2.58	<.001	0.90	0
	Tool	10 (4736)	2.01	1.28—3.15	.003	49.82	0.24
Smoking current	No Tool	5 (16780)	1.19	1.07—3.53	.030	85.56	0.33
	Tool	9 (1045)	0.83	0.60—1.15	.265	17.36	0.04
Smoking current/history	No Tool	8 (24122)	1.41	1.04—1.92	.029	76.07	0.12
	Tool	13 (1691)	0.92	0.73—1.15	.458	0	0
**Pre-Operative (Continuous)**							
Age (years)	No Tool	8 (3118)	3.11*	1.50—4.72	<.001	51.30	2.31
	Tool	20 (6185)	4.52*	2.95—6.09	<.001	82.93	9.67
BMI	No Tool		Insufficient Data				
	Tool	4 (776)	0.023*	-0.6—0.65	.653	15.02	0.07
Cognition: All tests	No Tool		Insufficient Data				
	Tool	9 (887)	-0.58†	-0.78— -0.37	<.001	34.11	0.03
Cognition: MMSE only	No Tool		Insufficient Data				
	Tool	7 (621)	1.14*	-1.91— -0.36	.004	77.72	0.68
Depression GDS	No Tool		Insufficient Data				
	Tool	2 (233)	0.75*	-0.15—1.65	.101	0	0
Education (years)	No Tool		Insufficient Data				
	Tool	6 (665)	-0.93*	-1.65— -0.20	.012	19.31	0.16
EuroSCORE	No Tool	3 (1058)	3.06*	0.28— 5.83	0.31	95.87	5.71

Variable	Delirium Diagnosis	k (n)	OR/MD*/ SMD†	95%CI	p value	I^2	Tau^2
		Pooled Estimate				Heterogeneity	
	Tool	7 (10141)	0.65*	0.14—1.16	.012	89.23	0.37
LVEF (%)	No Tool	4 (2518)	1.91*	-1.94—5.77	.330	91.24	13.83
	Tool	7 (790)	0.82*	-1.24—2.89	.435	57.05	4.24
**Intra-Operative (Continuous)**							
ACC time (mins)	No Tool	7 (3026)	9.88*	-0.52— 20.29	.063	94.19	176.11
	Tool	9 (4462)	3.61*	-0.97—8.18	.123	75.77	34.07
CPB time (mins)	No Tool	8 (7693)	4.98*	2.33—7.63	<.001	0	0
	Tool	13 (4719)	7.91*	2.37—13.45	.005	60.91	52.60
Duration of surgery (mins)	No Tool	Insufficient Data					
	Tool	12 (1851)	19.66*	7.18—32.14	.002	77.61	342.16
Intubation time (hours)	No Tool	3 (2194)	7.391*	1.78—13.00	.010	94.02	22.48
	Tool	8 (4499)	6.62*	1.25—12.00	.016	98.50	57.31
Number of grafts	No Tool	3 (1863)	0.06*	-0.27—0.38	.738	58.79	0.05
	Tool	5 (868)	0.15*	0.04—0.27	.009	0	0
**Post-Operative (Categorical)**							
Arrhythmia, incl. AF	No Tool	7 (4423)	4.26	2.16—8.40	<.001	82.46	0.65
	Tool	9 (4386)	2.98	1.93—4.61	<.001	52.46	0.20
**Post-Operative (Continuous)**							
LOS in ICU (days)	No Tool	4 (2390)	3.39*	-0.16—6.94	.061	99.04	12.59
	Tool	10 (4787)	1.69*	1.06—2.31	<.001	94.03	0.86

Note: “Delirium Diagnosis” indicates analyses conducted by categorization of diagnostic method, where “No Tool” represents studies that did not utilize a specific instrument and “Tool” represents studies utilizing a standardized instrument e.g. Confusion Assessment Method (CAM) or the Delirium Rating Scale (DRS) to inform the reference standard. ACC= aortic cross-clamp, AF= atrial fibrillation, BMI= body mass index, CI= cognitive index score, CPB= cardiopulmonary bypass, CVA= cerebrovascular attack, GDS= geriatric depression scale, k= number of estimates (number of studies), LOS= length of stay, LVEF= left ventricular ejection fraction, MD= mean difference, MI= myocardial infarction, MMSE= mini mental state examination, n= pooled sample size, OR= odds ratio, PVD= peripheral vascular disease, SMD= standardised mean difference and TIA= transient ischemic attack. Symbols following pooled estimates denote different effect sizes: indicating OR (no symbol), MD* and SMD†.

Table S8. **Study specific information regarding instruments utilized and method of classification/diagnosis utilized.**

Reference No.	Lead Author, Year	Instruments	Definitions of Cognitive Decline and Delirium (Standardized Delirium Measurement Tool: Y/N)
51	Al Tmimi, 2016	Delirium: CAM or CAM-ICU	Delirium: Positive CAM/CAM-ICU score (Y)
52	Baba, 2007	Cognition: HDS, Kana pick-out test, digit symbol, digit span (forward & backward)	Cognition: 20% method
53	Boodhwani, 2006	Cognition: Total learning free recall, consistent long-term retrieval, long-term retrieval, long-term storage, delayed recall, digit span (forward & backward), trails A & B, grooved pegboard, symbol digit modalities, RAVLT, Buschke selective reminding, WMS-III/mental control	Cognition: 0.5 SD method
54	Braekken, 1998	Cognition: WAIS vocabulary, WAIS picture completion, RCPM, CVLT-L, CVLT-S, CVLT-L, serial digit learning, WMS drawing, trails A & B, letter cancellation task, WAIS	Cognition: 1 SD method

Reference No.	Lead Author, Year	Instruments	Definitions of Cognitive Decline and Delirium (Standardized Delirium Measurement Tool: Y/N)
		digit symbol, computerized RT, COWAT, grooved pegboard	
55	Bucerius, 2005	Delirium: APA guidelines	Delirium: According to APA guidelines (N)
56	Caldas, 2019	Delirium: CAM-ICU	Delirium: Positive CAM-ICU score (Y)
57	Chen, 2017	Delirium: CAM-ICU	Delirium: Positive CAM-ICU score (Y)
58	Christiansen, 2016	Cognition: VVLT, CST, stroop test, LDCT	Cognition: Decline of >20% in ≥2 tests
59	Coffey, 1983	Delirium: DSM-III criteria	Delirium: DSM-III diagnostic criteria (N)
60	Colak, 2015	Cognition: MMSE, color trail test, grooved pegboard	Cognition: Miscellaneous
		Delirium: DSS	Delirium: Patient met criteria specific to study (N)
61	Cumurcu, 2008	Delirium: DRS (for severity), DSM-IV-TR criteria, MMSE	Delirium: DSM-IV-TR diagnostic criteria (N)

Reference No.	Lead Author, Year	Instruments	Definitions of Cognitive Decline and Delirium (Standardized Delirium Measurement Tool: Y/N)
62	de Tournay-Jette, 2011	Cognition: MMSE (pre-screen, excluded if <24 pre-surgery), logical memory subtest (of the Rivermead battery), RAVLT, digit symbol, trails A & B, stroop test, verbal fluency test	Cognition: 1 SD method
63	Dieleman, 2009	Cognition: RAVLT-L, RAVLT-R, grooved pegboard, trails A & B, Sternberg memory comparison, line orientation test, stroop test, continuous performance task, self-ordering tasks, visuospatial working memory, symbol digit modalities	Cognition: RCI method
64	Djaiani, 2003	Cognition: Randt short story, WAIS digit span, WMS figural memory, WAIS digit symbol, Trails B, RAVLT	Cognition: 1 SD method
65	Dong, 2014	Cognition: 12 neuropsychological tests used to assess cognitive functions including attention, memory and executive function	Cognition: RCI method

Reference No.	Lead Author, Year	Instruments	Definitions of Cognitive Decline and Delirium (Standardized Delirium Measurement Tool: Y/N)
		Delirium: CAM-ICU	Delirium: Positive CAM-ICU score (Features 1 and 2 are present and either Feature 3 or 4 is present) (Y)
		Delirium: DSM-III-R	Delirium: DSM-III-R diagnostic criteria (N)
66	Eriksson, 2002	Delirium: CAM and OBS scale	Delirium: Positive CAM score and fulfilled DSM-IV criteria (Y)
67	Goto, 2000	Cognition: HDS	Cognition: Cutoff method
68	Gottesman, 2010	Delirium: DSS	Delirium: Charts reviewed for delirium in those with neurologic injury (N)
69	Hall, 1999	Cognition: Trails A & B, digit span (forward & backward), COWAT	Cognition: Z-score method
70	Harmon, 2004	Cognition: RAVLT, trails A & B, grooved pegboard, COWAT, digit symbol	Cognition: RCI method

Reference No.	Lead Author, Year	Instruments	Definitions of Cognitive Decline and Delirium (Standardized Delirium Measurement Tool: Y/N)
		Delirium: DSM-III-R, MMSE	Delirium: Diagnosis based on the DSM-III-R criteria and the MMSE (N)
71	Harmon, 2005	Cognition: RAVLT, trails A & B, grooved pegboard, COWAT, digit symbol	Cognition: RCI method
		Delirium: DSM-III-R, MMSE	Delirium: Diagnosis based on the DSM-III-R criteria and the MMSE (N)
		Delirium: ICDSC	Delirium: ICDSC score ≥ 4 (Y)
72	Humphreys, 2016	Delirium: DSI, SPMSQ	Delirium: Positive DSI score (had any one of the critical symptoms of delirium: disorientation, disturbance of consciousness, or perceptual disturbance) (Y)
73	Kadoi, 2001	Cognition: MMSE, RAVLT, trails A & B, digit span (forward), grooved pegboard	Cognition: 1 SD method

Reference No.	Lead Author, Year	Instruments	Definitions of Cognitive Decline and Delirium (Standardized Delirium Measurement Tool: Y/N)
74	Kadoi, 2002	Cognition: MMSE, RAVLT, trails A & B, digit span (forward), grooved pegboard	Cognition: 1 SD method
75	Kadoi, 2003	Cognition: MMSE, RAVLT, trails A & B, digit span (forward), grooved pegboard	Cognition: 1 SD method
76	Kadoi, 2005	Cognition: MMSE, RAVLT, trails A & B, digit span (forward), grooved pegboard	Cognition: 1 SD method
77	Kadoi, 2007	Cognition: MMSE, RAVLT, trails A & B, digit span (forward), grooved pegboard	Cognition: 1 SD method
79	Kadoi, 2011 (a)	Cognition: MMSE, RAVLT, trails A & B, digit span (forward), grooved pegboard	Cognition: 1 SD method
78	Kadoi, 2011 (b)	Cognition: MMSE, RAVLT, trails A & B, digit span (forward), grooved pegboard	Cognition: 1 SD method
80	Kazmierski, 2014 (a)	Cognition: MoCA, trails B	Cognition: Cutoff method

Reference No.	Lead Author, Year	Instruments	Definitions of Cognitive Decline and Delirium (Standardized Delirium Measurement Tool: Y/N)
		Delirium: CAM or CAM-ICU, RASS	Delirium: If RASS was above –4 (–3 through +4), assessment with the CAM-ICU was administered (Y)
81	Kazmierski, 2014 (b)	Delirium: CAM-ICU, MDAS (for severity)	Delirium: Positive CAM-ICU score (Feature 1 and Feature 2 and either Feature 3 or 4 are present) (Y)
82	Kazmierski, 2014 (c)	Delirium: CAM-ICU	Delirium: If RASS was above –4 (–3 through +4), assessment with the CAM-ICU was administered (Y)
83	Khan, 2014	Delirium: DSM-IV	Delirium: Diagnosed using DSM-IV criteria (N)
84	Khatri, 1999	Cognition: Randt short story, WAIS-R digit span, WAIS-R digit symbol, trails B, figural memory	Cognition: 20% method
85	Kok, 2017	Cognition: CogState brief computerized test battery (detection task, identification task, one card learning task and one back task)	Cognition: RCI method

Reference No.	Lead Author, Year	Instruments	Definitions of Cognitive Decline and Delirium (Standardized Delirium Measurement Tool: Y/N)
86	Kumpaitiene, 2019	Cognition: MMSE, RAVLT, WAIS digit span, WAIS digit symbol, Shulte table	Cognition: Pre-post change of >2 points in combined studentized score, or >2 points in ≥2 individual studentized test scores.
87	Lachmann, 2018	Motor choice RT, grooved pegboard, Trails A & B, symbol digit modalities, stroop test, continuous performance task, RAVLT, self-ordering tasks, visual/spatial working memory, Sternberg memory comparison, line orientation	Cognition: Decrease of ≥20% on ≥3 tests
88	Leenders, 2018	Delirium: CAM, CAM-ICU, multidisciplinary consultation	Delirium: Administration of haloperidol in addition to positive CAM or CAM-ICU score and multidisciplinary consultation (Y)
89	Li, 2015	Delirium: CAM	Delirium: Positive CAM score (Features 1 and 2 are present and either Feature 3 or 4 is present) (Y)
90	Liu, 2009	Cognition: WMS mental control, WMS visual retention, WMS paired-associate verbal learning, digit span (forward and backward),	Cognition: RCI method

Reference No.	Lead Author, Year	Instruments	Definitions of Cognitive Decline and Delirium (Standardized Delirium Measurement Tool: Y/N)
		WAIS-R digit symbol, trails A, grooved pegboard (dom & non-dom)	
91	Loponen, 2008	Delirium: DSS (clinically diagnosed)	Delirium: Clinically diagnosed with requirement that temporary medication, i.e. diazepam or haloperidol, was needed to sedate the delirious patient (N)
92	Mardani, 2012	Delirium: DSM-IV, MMSE	Delirium: DSM-IV criteria interviews conducted on patients with a MMSE score ≤ 23 (N)
93	Mariscalco, 2012	Delirium: CAM-ICU	Delirium: At least 2 positive assessments on CAM-ICU (Features 1 and 2 are present and either Feature 3 or 4 is present) (Y)
94	Martin, 2010	Delirium: STS	Delirium: Defined according to STS definition (N)
95	Martin, 2012	Delirium: STS	Delirium: Defined according to STS definition (N)

Reference No.	Lead Author, Year	Instruments	Definitions of Cognitive Decline and Delirium (Standardized Delirium Measurement Tool: Y/N)
96	Mathew, 2006	Cognition: Randt short story, WMS modified visual reproduction test, WAIS-R digit span, WAIS-R digit symbol, trails B	Cognition: 1 SD method (domain)
97	Mathew, 2007	Cognition: Randt short story, WMS modified visual reproduction test, WAIS-R digit span, WAIS-R digit symbol, trails B	Cognition: 1 SD method (domain)
98	Miyazaki, 2011	Delirium: DSM-IV	Delirium: Diagnosed according to DSM-IV criteria or administering antipsychotic agents by reviewing medical records during the ICU stay (N)
99	Mu, 2010	Delirium: CAM-ICU, RASS	Delirium: If RASS was above –4 (–3 through +4), assessment with the CAM-ICU was administered (4-step algorithm) (Y)
100	Mu, 2013	Cognition: Trails A, grooved pegboard (dom & non-dom), the WMS-Chinese edn. of the mental control subtest, digit span subtest (forward & backward), visual retention subtest,	Cognition: 1 SD method (preop) / RCI method

Reference No.	Lead Author, Year	Instruments	Definitions of Cognitive Decline and Delirium (Standardized Delirium Measurement Tool: Y/N)
		paired associate verbal learning subtest, digit symbol subtest	
101	Newman, 1987	Cognition: RAVLT, non-verbal recognition memory test (computer-administered), Trails A & B, WAIS block design, Purdue Pegboard (left, right, and both hands), letter cancellation test, symbol digit replacement (computer-based), choice RT (computer-based)	Cognition: Decrease of ≥1SD in >3 tests
102	Nikolic, 2012	Delirium: CAM	Delirium: Positive CAM score (Features 1 and 2 are present and either Feature 3 or 4 is present) (Y)
103	Norkiene, 2007	Delirium: DSM-IV	Delirium: Clinician diagnosis according to DSM-IV criteria (N)
104	Norkiene, 2011	Cognition: MMSE, RAVLT, trails A & B, digit span, digit symbol, cube drawing	Cognition: 1 SD method
		Delirium: DSM-IV	Delirium: Defined according to DSM-IV criteria (N)

Reference No.	Lead Author, Year	Instruments	Definitions of Cognitive Decline and Delirium (Standardized Delirium Measurement Tool: Y/N)
105	Oh, 2008	Cognition: MMSE, trails A, grooved pegboard	Cognition: 20% method
106	Oh, 2017	Delirium: DSS (psychiatric consultation & DSM-IV)	Delirium: Diagnosed by psychiatric consultation according to DSM-IV criteria (N)
107	Oldham, 2015	Cognition: CDR, MMSE, digit span, HVLT, WMS-IV, progressive digit sequencing, three word fluency tasks, NAB mazes subtest, trails A & B, digit symbol	Cognition: Cutoff method, 1 SD method
		Delirium: aDST, CAM, DI, MMSE	Delirium: Determined based on CAM (Y)
108	Oldham, 2019	Delirium: CAM, MMSE, abbreviated digit span test, DI	Delirium: Psychiatrist diagnosis based on CAM, MMSE, digit span test and delirium index (Y)
109	Omiya, 2015	Delirium: DRS-R-98	Delirium: DRS-R-98 score ≥ 8 (Y)
110	Otomo, 2013	Delirium: DSM-IV, DRS	Delirium: Diagnosed according to DSM-IV criteria & DRS score (Y)

Reference No.	Lead Author, Year	Instruments	Definitions of Cognitive Decline and Delirium (Standardized Delirium Measurement Tool: Y/N)
111	Palmbergen, 2012	Delirium: DOS scale, confirmed by geriatrician or internist	Delirium: DOS scale for screening. If suspected, confirmed by geriatrician or internist (Y)
112	Plaschke, 2010	Delirium: CAM-ICU (German), RASS	Delirium: Positive CAM-ICU score (Y)
113	Reents, 2002	Cognition: d2-letter cancellation test, trails B, Benton‘s visual retention test, WAIS block design, WAIS digit span	Cognition: 1 SD method
		Delirium: DSM-IV	Delirium: Defined according to DSM-IV criteria (N)
114	Restrepo, 2002	Cognition: Trails B, oral and written naming test, oral reading tests, line cancellation, Bells tests	Cognition: Z-score method
115	Ringaitiene, 2015	Delirium: CAM-ICU	Delirium: Positive CAM-ICU score (Y)
116	Robson, 2000	Cognition: RAVLT, trails A & B, PASAT, grooved pegboard, COWAT, NART, block design, object assembly test, digit symbol, picture completion test	Cognition: 1 SD method (<20% tests), 0.5 SD method (<20% tests)

Reference No.	Lead Author, Year	Instruments	Definitions of Cognitive Decline and Delirium (Standardized Delirium Measurement Tool: Y/N)
117	Rodriguez, 2010	Cognition: Group 1: RAVLT, trails A & B, grooved pegboard, symbol digit modalities, WAIS-R digit span, WMS mental control, letter (FAS test), category fluency (animal naming), finger tapping; Group 2: RAVLT, trails A & B, grooved pegboard, symbol digit modalities, WAIS-R digit span, verbal fluency (FAS test), categories (animal naming)	Cognition: Z-score method
118	Rolfson, 1999 (a)	Delirium: CAM, MMSE, DSM-III-R	Delirium: Diagnosed according to DSM-III-R criteria, based on results from standardized measures (e.g. CAM) and consultation with nurses, family members and hospital records (Y)
119	Rolfson, 1999 (b)	Delirium: DSM-III-R on clinical grounds (CAM, CAM-MD, CAM-RN, MMSE, clock drawing test, MD chart review, RN chart review - used to determine clinical diagnosis)	Delirium: Clinically diagnosed according to DSM-III-R criteria, based on results from standardized measures (Y)

Reference No.	Lead Author, Year	Instruments	Definitions of Cognitive Decline and Delirium (Standardized Delirium Measurement Tool: Y/N)
120	Royse, 2000	Cognition: Recall (short-term & delayed), COWAT, trails A & B, grooved pegboard (dom & non-dom), digit symbol, digit span (forward & backward)	Cognition: 20% method
121	Royse, 2011	Cognition: Trails A & B, COWAT, stroop test, letter cancellation, grooved pegboard (dom & non-dom), RAVLT, digit span (forward & backward), symbol digit modalities	Cognition: 1 SD method (<20% tests)
		Delirium: CAM	Delirium: Positive CAM score (Y)
122	Rudolph, 2005	Delirium: CAM, digit span, DSI, MDAS, MMSE	Delirium: Positive CAM score (Features 1 and 2 are present and either Feature 3 or 4 is present) (Y)
123	Rudolph, 2006	Delirium: CAM (CAM-ICU for postoperatively intubated patients), digit span, DSI, MDAS, MMSE	Delirium: Positive CAM score (Features 1 and 2 are present and either Feature 3 or 4 is present) (Y)

Reference No.	Lead Author, Year	Instruments	Definitions of Cognitive Decline and Delirium (Standardized Delirium Measurement Tool: Y/N)
124	Rudolph, 2009	Delirium: CAM, digit span, DSI, MDAS, MMSE	Delirium: Positive CAM score (Features 1 and 2 are present and either Feature 3 or 4 is present) (Y)
125	Sahan, 2018	Cognition: WMS logical memory, clock drawing test, word list generation test, digit span, visuomotor spatial skills test	Cognition: 1 SD method (≥2 tests)
6	Santos, 2004	Delirium: DSM-IV	Delirium: Diagnosed by geriatrician based on DSM-IV criteria, in addition to notes from nurses and physicians (N)
126	Scott, 2002	Cognition: WMS-R logical memory (I & II), altered form of WMS-R digit span, trails A & B, COWAT	Cognition: 1 SD method (<20% tests), 1 SD method
127	Sevuk, 2015	Delirium: DRS-R-98 (for severity), ICDSC	Delirium: ICDSC score ≥ 4 (Y)
128	Siepe, 2011	Delirium: MMSE, psychologist assessment	Delirium: 10 point drop or more on MMSE from pre-op and a positive assessment by a psychologist (N)

Reference No.	Lead Author, Year	Instruments	Definitions of Cognitive Decline and Delirium (Standardized Delirium Measurement Tool: Y/N)
129	Silbert, 2006	Cognition: CERAD AVLT, digit symbol, Trails A & B, COWAT, semantic fluency test, grooved pegboard test (dom & non-dom)	Cognition: 1 SD method and 20% method (≥2 tests)
130	Silbert, 2008	Cognition: CERAD AVLT, Digit symbol, Trails A & B, COWAT, semantic fluency test, grooved pegboard test (dom & non-dom)	Cognition: 1 SD method (≥2 tests)
131	Slater, 2009	Cognition: MMSE, Trails A & B, HVLT (trials 1, 2, 3, B & C), grooved pegboard (dom & non-dom), stroop test (part C & CW)	Cognition: 1 SD method (<20% tests)
		Delirium: DRS	Delirium: Based on DRS (Y)
132	Smith, 1986	Cognition: WAIS vocab and picture completion subtests, RAVLT, block design, grooved pegboard, trails A & B, letter cancellation, digit symbol replacement, two-choice RT	Cognition: 1 SD method

Reference No.	Lead Author, Year	Instruments	Definitions of Cognitive Decline and Delirium (Standardized Delirium Measurement Tool: Y/N)
133	Smith, 2000	Cognition: RAVLT, Rey auditory nonverbal memory, Trails A & B, letter cancellation, symbol-digit replacement, visual RT, grooved pegboard (dom & non-dom), finger tapping (dom & non-dom)	Cognition: 20% method (≥2 tests)
134	Stump, 1996	Cognition: Trails A & B, grooved pegboard (dom & non-dom), finger tapping (dom & non-dom), symbol digit, letter cancellation, visual RT, verbal and nonverbal memory	Cognition: 20% method (≥2 tests)
135	Subramaniam, 2019	Delirium: CAM, CAM-ICU	Delirium: Positive CAM or CAM-ICU score (Y)
136	Suksompong, 2002	Cognition: Thai Mental State Exam	Cognition: Miscellaneous
137	Swaminathan, 2002	Cognition: Randt short story (immediate & delay), digit symbol, trails B, digit span (forward & backward), figural memory (immediate & delayed)	Cognition: 1 SD method (domain)

Reference No.	Lead Author, Year	Instruments	Definitions of Cognitive Decline and Delirium (Standardized Delirium Measurement Tool: Y/N)
138	Sylivris, 1998	Cognition: WAIS-R general information questionnaire, digit span, digit symbol, RAVLT, COWAT	Cognition: Miscellaneous
139	Tagarakis, 2007	Delirium: DRS	Delirium: Based on DRS, which was performed on patients suspected to develop delirium (Y)
140	Tamura, 2019	Delirium: ICDSC	Delirium: ICDSC >3
141	Toeg, 2013	Cognition: Buschke selective reminding or RAVLT, WAIS-R digit span, finger tapping task, letter and category fluency, trails A & B, grooved pegboard, symbol digit modalities	Cognition: 1 SD method (domain)
142	Trubnikova, 2014	Cognition: Complex visuomotor reaction (reaction time, number of errors), functional mobility of nervous processes and performance of brain responses to feedback (reaction time, number of errors, missed signals), Bourdons test, visual short-term memory tests	Cognition: 20% method

Reference No.	Lead Author, Year	Instruments	Definitions of Cognitive Decline and Delirium (Standardized Delirium Measurement Tool: Y/N)
		(memorisation of 10 numbers, 10 words, 10 nonsense syllables)	
143	Tully, 2010	Delirium: DSI, DSM-IV-TR, SPMSQ	Delirium: Classification based on DSM-IV-TR criteria. Evidence of perceptual disturbance and/or language disturbance was requisite for a delirium diagnosis. Neurology assessments, SPMSQ results and medical notes also evaluated. (N)
144	van Dijk, 2004	Cognition: RAVLT-L, RAVLT-R, grooved pegboard, trails A & B, Sternberg memory comparison, line orientation test, stroop test	Cognition: 20% method
145	Yilmaz, 2016	Delirium: CAM-ICU	Delirium: Positive CAM-ICU score (Features 1 and 2 are present and either Feature 3 or 4 is present) (Y)
146	Zhang, 2015	Delirium: CAM-ICU, RASS	Delirium: Positive CAM-ICU score (Features 1 and 2 are present and either Feature 3 or 4 is present) (Y)

Reference No.	Lead Author, Year	Instruments	Definitions of Cognitive Decline and Delirium (Standardized Delirium Measurement Tool: Y/N)

Note. *see Supplementary Table 9 for glossary of instrument acronyms*

Table S9. **Glossary of abbreviations.**

Cognitive Impairment	
**AVLT**	Auditory Verbal Learning Test
**CDR**	Clinical Dementia Rating scale
**CERAD**	The Consortium to Establish a Registry for Alzheimer's Disease
**COWAT**	Controlled Oral Word Association Test
**CST**	Concept Shifting Test
**CVLT**	California Verbal Learning Test
**Digit symbol**	Digit Symbol Substitution Task
**HDS**	Hasegawa Dementia Scale
**HVLT**	Hopkins Verbal Learning Test
**LDCT**	Letter-Digit Coding Test
**MMSE**	Mini Mental State Examination
**MoCA**	Montreal Cognitive Assessment
**NAB**	Neuropsychological Assessment Battery
**NART**	National Adult Reading Test
**PASAT**	Paced Auditory Serial Addition Task
**Randt short story**	Randt Memory Test Short-Story Module
**RAVLT**	Rey Auditory Verbal Learning Test
**RAVLT-L**	Rey Auditory-Verbal Learning – Learning Trial
**RAVLT-R**	Rey Auditory-Verbal Learning – Recognition Trial
**RCPM**	Raven Coloured Progressive Matrices
**RT**	Reaction Time
**Stroop test**	Stroop Colour Word Interference Test
**Trails A & B**	Halstead-Reitan Trail-making tests A & B

**VVLT**	Visual Verbal Learning Test
**WAIS**	Wechsler Adult Intelligence Scale
**WMS**	Wechsler Memory Scale
**Delirium**	
**aDST**	abbreviated Digit Span Test
**APA**	American Psychiatric Association
**CAM**	Confusion Assessment Method
**CAM-ICU**	Confusion Assessment Method for the ICU
**DI**	Delirium Index
**DOS**	Delirium Observation Screening scale
**DRS**	Delirium Rating Scale
**DRS-R-98**	Delirium Rating Scale Revised-98
**DSI**	Delirium Symptom Interview
**DSM-III-R**	Diagnostic and Statistical Manual of Mental Disorders 3rd ed., Revised.
**DSM-IV**	Diagnostic and Statistical Manual of Mental Disorders 4th ed.
**DSM-IV-TR**	Diagnostic and Statistical Manual of Mental Disorders 4th ed., Text Revision
**DSS**	Definition that is specific to the study
**ICDSC**	The Intensive Care Delirium Screening Checklist
**MDAS**	Memorial Delirium Assessment Scale
**MMSE**	Mini Mental State Examination
**OBS**	Organic Brain Syndrome scale
**RASS**	The Richmond Agitation Sedation Scale
**SPMSQ**	Short Portable Mental Status Questionnaire
**STS**	Accordance with Society of Thoracic Surgeons

Figure S1. Forest plots for delirium post-CABG analyses.

Variable	Forest Plot
**Pre-Operative (Categorical)**	
Alcoholism	See plot below
Arrhythmia, incl. AF	See plot below
BMI >28 (including >30)	See plot below

Alcoholism

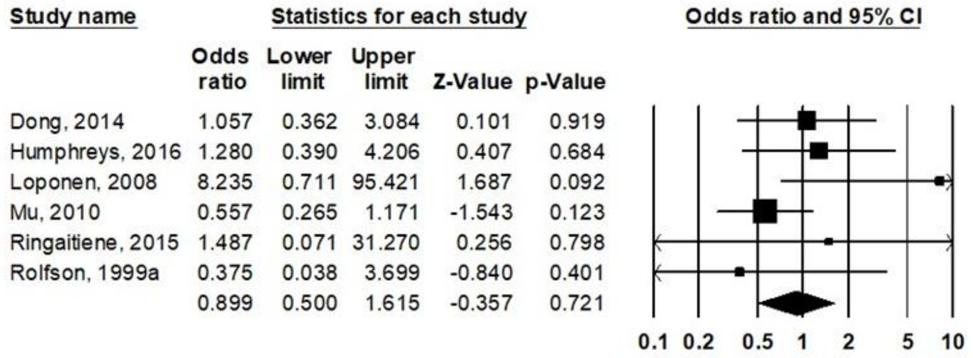

Study name	Odds ratio	Lower limit	Upper limit	Z-Value	p-Value
Dong, 2014	1.057	0.362	3.084	0.101	0.919
Humphreys, 2016	1.280	0.390	4.206	0.407	0.684
Loponen, 2008	8.235	0.711	95.421	1.687	0.092
Mu, 2010	0.557	0.265	1.171	-1.543	0.123
Ringaitiene, 2015	1.487	0.071	31.270	0.256	0.798
Rolfson, 1999a	0.375	0.038	3.699	-0.840	0.401
	0.899	0.500	1.615	-0.357	0.721

Arrhythmia, incl. AF

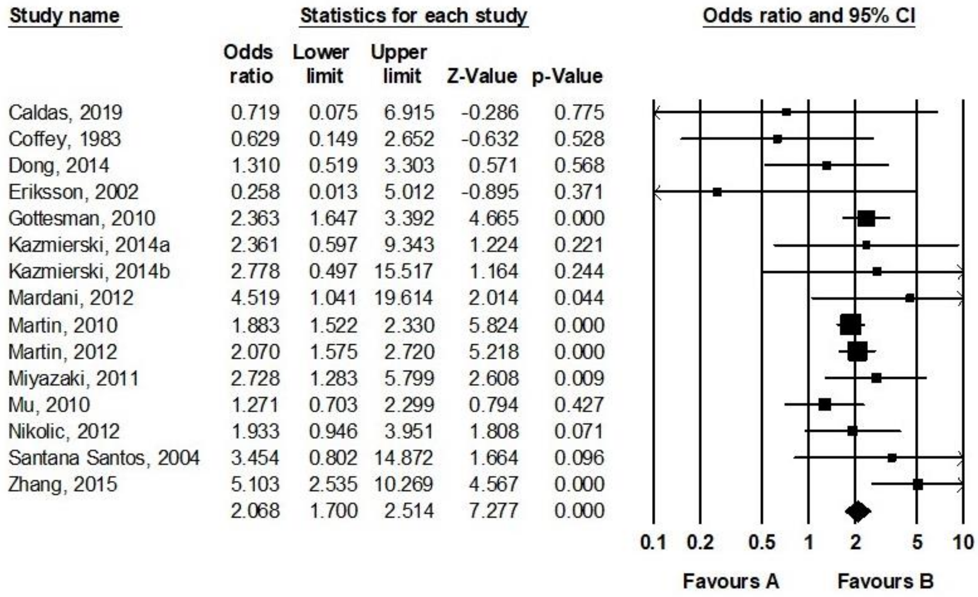

Study name	Odds ratio	Lower limit	Upper limit	Z-Value	p-Value
Caldas, 2019	0.719	0.075	6.915	-0.286	0.775
Coffey, 1983	0.629	0.149	2.652	-0.632	0.528
Dong, 2014	1.310	0.519	3.303	0.571	0.568
Eriksson, 2002	0.258	0.013	5.012	-0.895	0.371
Gottesman, 2010	2.363	1.647	3.392	4.665	0.000
Kazmierski, 2014a	2.361	0.597	9.343	1.224	0.221
Kazmierski, 2014b	2.778	0.497	15.517	1.164	0.244
Mardani, 2012	4.519	1.041	19.614	2.014	0.044
Martin, 2010	1.883	1.522	2.330	5.824	0.000
Martin, 2012	2.070	1.575	2.720	5.218	0.000
Miyazaki, 2011	2.728	1.283	5.799	2.608	0.009
Mu, 2010	1.271	0.703	2.299	0.794	0.427
Nikolic, 2012	1.933	0.946	3.951	1.808	0.071
Santana Santos, 2004	3.454	0.802	14.872	1.664	0.096
Zhang, 2015	5.103	2.535	10.269	4.567	0.000
	2.068	1.700	2.514	7.277	0.000

BMI >28 (including >30)

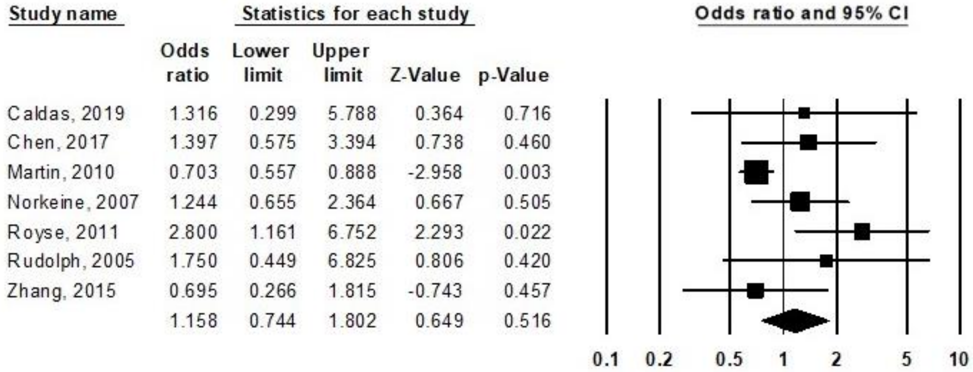

Study name	Odds ratio	Lower limit	Upper limit	Z-Value	p-Value
Caldas, 2019	1.316	0.299	5.788	0.364	0.716
Chen, 2017	1.397	0.575	3.394	0.738	0.460
Martin, 2010	0.703	0.557	0.888	-2.958	0.003
Norkeine, 2007	1.244	0.655	2.364	0.667	0.505
Royse, 2011	2.800	1.161	6.752	2.293	0.022
Rudolph, 2005	1.750	0.449	6.825	0.806	0.420
Zhang, 2015	0.695	0.266	1.815	-0.743	0.457
	1.158	0.744	1.802	0.649	0.516

BMI ≥30 only

Study name	Odds ratio	Lower limit	Upper limit	Z-Value	p-Value
Caldas, 2019	1.316	0.299	5.788	0.364	0.716
Chen, 2017	1.397	0.575	3.394	0.738	0.460
Norkeine, 2007	1.244	0.655	2.364	0.667	0.505
Royse, 2011	2.800	1.161	6.752	2.293	0.022
Rudolph, 2005	1.750	0.449	6.825	0.806	0.420
	1.573	1.045	2.368	2.172	0.030

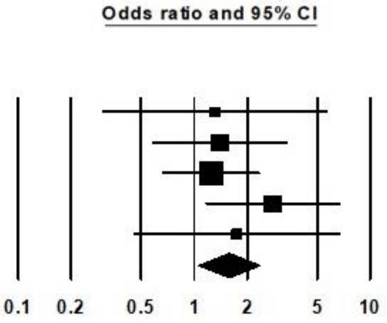

Cognitive Impairment

Study name	Odds ratio	Lower limit	Upper limit	Z-Value	p-Value
Leenders, 2018	4.081	1.446	11.520	2.656	0.008
Oldham, 2018	3.000	1.123	8.013	2.192	0.028
Kazmierski, 2014a	7.619	2.927	19.832	4.160	0.000
Oldham, 2015	6.111	1.056	35.363	2.021	0.043
Rudolph, 2005	4.000	0.981	16.311	1.933	0.053
Zhang, 2015	4.446	1.676	11.797	2.997	0.003
Rolfson, 1999b	2.311	0.752	7.106	1.462	0.144
	4.170	2.746	6.332	6.700	0.000

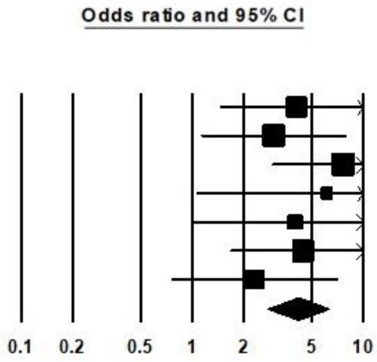

Depression

Study name	Odds ratio	Lower limit	Z-Value	Upper limit	p-Value
Oldham, 2018	3.984	1.158	2.193	13.705	0.028
Santana Santos, 2004	1.242	0.553	0.525	2.787	0.600
Tully, 2010	3.490	1.477	2.850	8.245	0.004
Rolfson, 1999a	4.846	0.408	1.250	57.544	0.211
	2.493	1.291	2.722	4.811	0.006

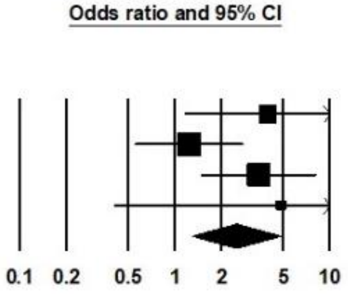

Diabetes

Study name	Odds ratio	Lower limit	Upper limit	Z-Value	p-Value
Caldas, 2019	2.333	0.746	7.301	1.456	0.145
Chen, 2017	1.421	0.690	2.926	0.954	0.340
Leenders, 2018	1.456	0.722	2.939	1.050	0.294
Dong, 2014	2.718	1.122	6.584	2.215	0.027
Eriksson, 2002	1.889	0.394	9.067	0.795	0.427
Gottesman, 2010	1.285	1.011	1.632	2.050	0.040
Bucerius, 2005	1.616	1.390	1.879	6.239	0.000
Coffey, 1983	1.564	0.597	4.098	0.910	0.363
Humphreys, 2016	2.210	1.110	4.400	2.257	0.024
Kazmierski, 2014a	1.154	0.517	2.574	0.350	0.727
Kazmierski, 2014b	1.000	0.363	2.751	0.000	1.000
Khan, 2014	1.237	0.872	1.755	1.191	0.234
Loponen, 2008	0.345	0.077	1.535	-1.398	0.162
Mardani, 2012	0.661	0.143	3.052	-0.531	0.596
Mariscalco, 2012	1.398	0.910	2.150	1.529	0.126
Martin, 2010	1.481	1.299	1.689	5.869	0.000
Martin, 2012	1.549	1.290	1.860	4.687	0.000
Miyazaki, 2011	1.539	1.021	2.320	2.059	0.039
Mu, 2010	1.767	1.041	2.998	2.109	0.035
Nikolic, 2012	1.997	1.152	3.461	2.465	0.014
Norkeine, 2007	1.792	0.887	3.619	1.626	0.104
Omiya, 2015	0.354	0.036	3.455	-0.893	0.372
Otomo, 2013	1.555	0.548	4.415	0.830	0.407
Ringaitiene, 2015	1.882	0.416	8.516	0.821	0.412
Rudolph, 2005	2.286	0.586	8.914	1.191	0.234
Santana Santos, 2004	1.436	0.804	2.564	1.224	0.221
Sevuk, 2015	1.124	0.611	2.069	0.375	0.707
Tully, 2010	0.718	0.345	1.495	-0.886	0.376
Yilmaz, 2016	0.820	0.284	2.371	-0.366	0.714
Zhang, 2015	1.755	0.988	3.116	1.920	0.055
	1.491	1.390	1.599	11.183	0.000

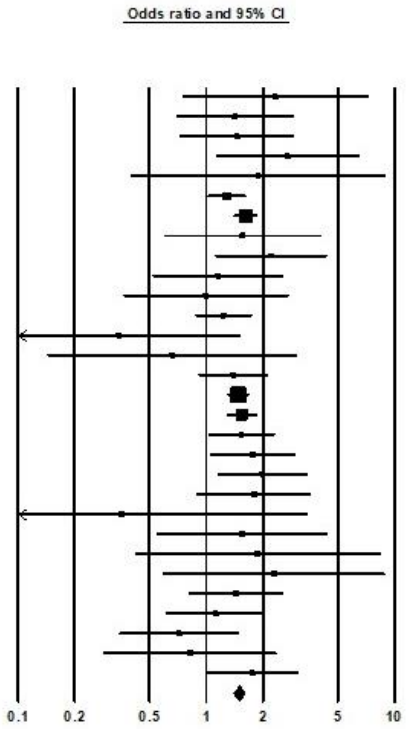

Dyslipidemia/Hyperlipidemia

Study name	Odds ratio	Lower limit	Upper limit	Z-Value	p-Value
Caldas, 2019	0.395	0.126	1.240	-1.592	0.111
Dong, 2014	1.399	0.640	3.055	0.842	0.400
Coffey, 1983	0.212	0.074	0.606	-2.898	0.004
Humphreys, 2016	2.560	0.909	7.206	1.780	0.075
Loponen, 2008	1.066	0.368	3.085	0.118	0.906
Mariscalco, 2012	0.630	0.411	0.965	-2.123	0.034
Mu, 2010	1.028	0.615	1.720	0.107	0.915
Omiya, 2015	4.000	0.217	73.618	0.933	0.351
Otomo, 2013	0.461	0.162	1.314	-1.449	0.147
Rudolph, 2005	1.474	0.121	17.913	0.304	0.761
Santana Santos, 2004	0.639	0.332	1.233	-1.334	0.182
Sevuk, 2015	1.442	0.784	2.652	1.176	0.240
Tully, 2010	1.481	0.658	3.336	0.948	0.343
	0.890	0.633	1.251	-0.672	0.502

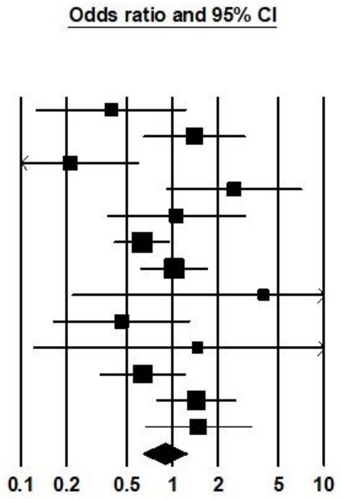

Education>12years/high school

Study name	Odds ratio	Lower limit	Upper limit	Z-Value	p-Value
Chen, 2017	0.568	0.211	1.535	-1.115	0.265
Oldham, 2018	0.779	0.291	2.086	-0.497	0.619
Rudolph, 2006	1.000	0.416	2.405	0.000	1.000
Santana Santos, 2004	0.368	0.134	1.009	-1.942	0.052
	0.658	0.407	1.064	-1.708	0.088

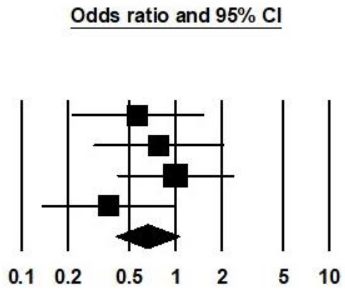

Hypertension

Study name	Odds ratio	Lower limit	Upper limit	Z-Value	p-Value
Caldas, 2019	2.368	0.473	11.871	1.048	0.294
Chen, 2017	4.440	1.794	10.987	3.225	0.001
Dong, 2014	1.446	0.658	3.179	0.919	0.358
Eriksson, 2002	1.800	0.420	7.712	0.792	0.428
Gottesman, 2010	1.391	1.048	1.847	2.285	0.022
Coffey, 1983	1.649	0.836	3.255	1.442	0.149
Humphreys, 2016	0.750	0.374	1.505	-0.810	0.418
Kazmierski, 2014a	1.285	0.448	3.687	0.467	0.641
Khan, 2014	1.683	1.176	2.410	2.844	0.004
Loponen, 2008	1.593	0.581	4.365	0.905	0.365
Mardani, 2012	6.067	2.536	14.512	4.051	0.000
Mariscalco, 2012	0.671	0.435	1.037	-1.797	0.072
Martin, 2010	1.511	1.302	1.754	5.434	0.000
Martin, 2012	1.706	1.386	2.100	5.040	0.000
Miyazaki, 2011	2.705	1.526	4.796	3.406	0.001
Mu, 2010	1.395	0.818	2.379	1.221	0.222
Nikolic, 2012	2.240	0.766	6.548	1.474	0.141
Norkeine, 2007	0.965	0.456	2.040	-0.094	0.925
Omiya, 2015	0.850	0.135	5.366	-0.173	0.863
Otomo, 2013	0.487	0.164	1.447	-1.294	0.196
Ringaitiene, 2015	0.874	0.043	17.656	-0.088	0.930
Rudolph, 2005	2.600	0.445	15.177	1.062	0.288
Santana Santos, 2004	2.228	1.115	4.451	2.268	0.023
Sevuk, 2015	0.867	0.424	1.773	-0.392	0.695
Tully, 2010	0.811	0.404	1.631	-0.587	0.557
Yilmaz, 2016	0.650	0.213	1.986	-0.756	0.450
Zhang, 2015	1.319	0.762	2.283	0.989	0.323
	1.436	1.212	1.702	4.174	0.000

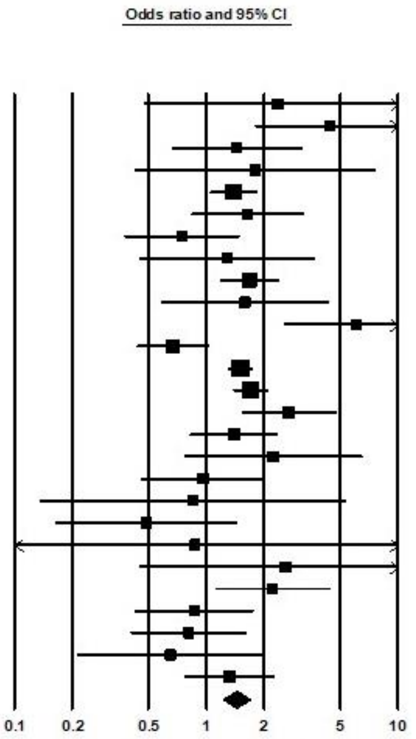

Sex (male)

Study name	Odds ratio	Lower limit	Upper limit	Z-Value	p-Value
Caldas, 2019	1.026	0.281	3.747	0.039	0.969
Chen, 2017	0.180	0.077	0.421	-3.960	0.000
Leenders, 2018	2.525	0.752	8.482	1.499	0.134
Oldham, 2018	0.267	0.102	0.702	-2.678	0.007
Cumurcu, 2008	0.373	0.093	1.495	-1.392	0.164
Dong, 2014	0.928	0.377	2.287	-0.162	0.871
Eriksson, 2002	0.583	0.136	2.506	-0.725	0.469
Gottesman, 2010	0.839	0.651	1.079	-1.367	0.172
Al Tmimi, 2016	0.595	0.164	2.160	-0.789	0.430
Humphreys, 2016	0.550	0.229	1.323	-1.335	0.182
Kazmierski, 2014a	0.436	0.172	1.104	-1.751	0.080
Khan, 2014	1.978	1.288	3.039	3.116	0.002
Li, 2015	2.273	0.097	53.391	0.510	0.610
Loponen, 2008	4.727	0.621	35.954	1.500	0.133
Mardani, 2012	0.441	0.128	1.526	-1.292	0.196
Mariscalco, 2012	0.900	0.543	1.491	-0.410	0.682
Martin, 2010	1.095	0.937	1.280	1.140	0.254
Martin, 2012	1.123	0.905	1.395	1.053	0.292
Mu, 2010	0.974	0.504	1.882	-0.079	0.937
Nikolic, 2012	0.970	0.533	1.762	-0.102	0.919
Norkeine, 2007	1.958	0.818	4.689	1.508	0.132
Oldham, 2015	0.275	0.086	0.862	-2.171	0.030
Otomo, 2013	1.851	0.501	6.842	0.923	0.356
Palmbergen, 2012	1.320	0.718	2.427	0.893	0.372
Ringaitiene, 2015	0.667	0.148	2.993	-0.529	0.597
Rudolph, 2009	2.913	0.115	74.063	0.648	0.517
Rudolph, 2006	0.297	0.094	0.934	-2.076	0.038
Santana Santos, 2004	0.659	0.370	1.173	-1.417	0.156
Sevuk, 2015	0.785	0.429	1.438	-0.783	0.434
Tagarakis, 2007	0.898	0.400	2.017	-0.262	0.794
Tully, 2010	1.880	0.754	4.685	1.364	0.176
Yilmaz, 2016	0.620	0.164	2.346	-0.704	0.481
Zhang, 2015	1.410	0.704	2.827	0.969	0.332
Tamura, 2019	2.350	0.242	22.855	0.736	0.462
Rolfson, 1999a	2.231	0.568	8.759	1.150	0.250
	0.903	0.755	1.080	-1.119	0.263

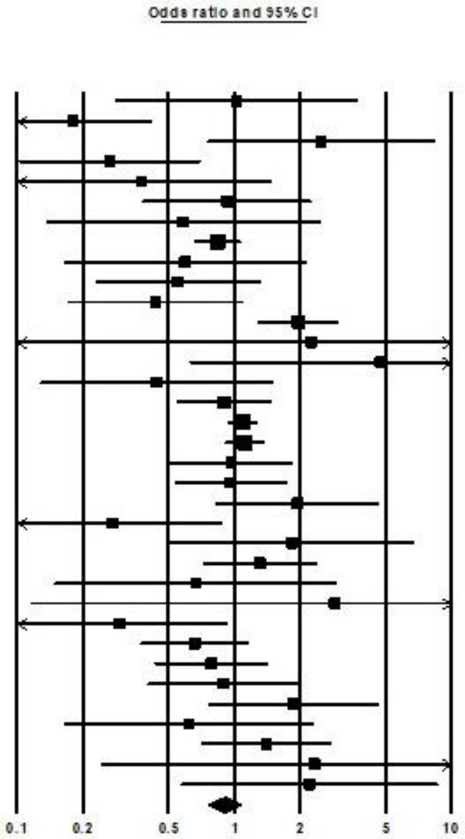

Kidney injury

Study name	Odds ratio	Lower limit	Upper limit	Z-Value	p-Value
Leenders, 2018	4.744	2.256	9.979	4.104	0.000
Humphreys, 2016	1.010	0.282	3.611	0.015	0.988
Mardani, 2012	1.973	0.824	4.723	1.525	0.127
Martin, 2010	1.918	1.487	2.473	5.019	0.000
Martin, 2012	1.607	1.147	2.252	2.755	0.006
Mu, 2010	1.229	0.322	4.691	0.301	0.763
Nikolic, 2012	1.045	0.476	2.293	0.109	0.913
Omiya, 2015	4.000	0.217	73.618	0.933	0.351
Otomo, 2013	1.814	0.361	9.126	0.723	0.470
Ringaitiene, 2015	2.106	0.093	47.538	0.468	0.640
Santana Santos, 2004	5.547	1.677	18.352	2.806	0.005
Tully, 2010	0.473	0.098	2.275	-0.935	0.350
Zhang, 2015	2.492	1.030	6.032	2.025	0.043
Oh, 2017	3.060	1.120	8.360	2.181	0.029
	1.944	1.502	2.517	5.044	0.000

Odds ratio and 95% CI

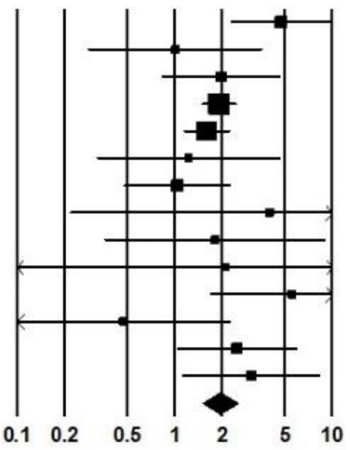

Previous MI <30 days

Study name	Odds ratio	Lower limit	Upper limit	Z-Value	p-Value
Humphreys, 2016	0.940	0.476	1.855	-0.178	0.858
Loponen, 2008	1.825	0.498	6.687	0.907	0.364
Mardani, 2012	4.224	1.552	11.496	2.820	0.005
Ringaitiene, 2015	1.675	0.372	7.547	0.672	0.502
Tully, 2010	1.172	0.574	2.394	0.435	0.663
	1.543	0.898	2.653	1.570	0.116

Odds ratio and 95% CI

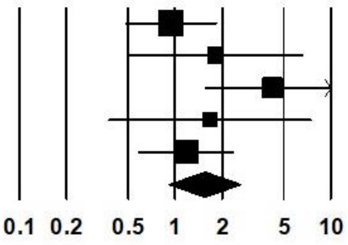

Previous MI history/ever

Study name	Odds ratio	Lower limit	Upper limit	Z-Value	p-Value
Caldas, 2019	1.024	0.243	4.326	0.033	0.974
Chen, 2017	2.037	0.959	4.324	1.852	0.064
Dong, 2014	1.478	0.669	3.264	0.966	0.334
Eriksson, 2002	1.222	0.336	4.448	0.304	0.761
Gottesman, 2010	1.241	0.978	1.575	1.773	0.076
Coffey, 1983	1.135	0.575	2.242	0.364	0.716
Mariscalco, 2012	1.056	0.692	1.610	0.253	0.801
Omiya, 2015	2.933	0.383	22.463	1.036	0.300
Ringaitiene, 2015	0.784	0.185	3.332	-0.329	0.742
Santana Santos, 2004	0.675	0.379	1.203	-1.332	0.183
Yilmaz, 2016	0.860	0.179	4.129	-0.188	0.851
	1.169	0.984	1.388	1.779	0.075

Odds ratio and 95% CI

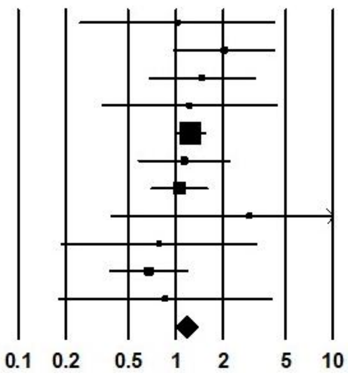

Previous stroke, TIA, CVA

Study name	Odds ratio	Lower limit	Upper limit	Z-Value	p-Value
Caldas, 2019	0.719	0.075	6.915	-0.286	0.775
Chen, 2017	0.914	0.363	2.300	-0.192	0.848
Leenders, 2018	1.765	0.760	4.100	1.321	0.186
Dong, 2014	2.603	0.714	9.490	1.449	0.147
Eriksson, 2002	9.486	1.475	60.991	2.370	0.018
Gottesman, 2010	2.309	1.683	3.168	5.190	0.000
Khan, 2014	18.365	2.130	158.346	2.648	0.008
Mardani, 2012	2.709	1.102	6.662	2.171	0.030
Mariscalco, 2012	2.545	1.389	4.666	3.022	0.003
Martin, 2010	3.895	2.869	5.290	8.709	0.000
Miyazaki, 2011	2.150	1.365	3.386	3.304	0.001
Mu, 2010	1.924	0.879	4.212	1.637	0.102
Norkeine, 2007	1.400	0.422	4.644	0.550	0.582
Palmbergen, 2012	7.781	2.063	29.354	3.029	0.002
Rolfson, 1999a	6.769	1.178	38.898	2.144	0.032
	2.546	1.936	3.350	6.680	0.000

Odds ratio and 95% CI

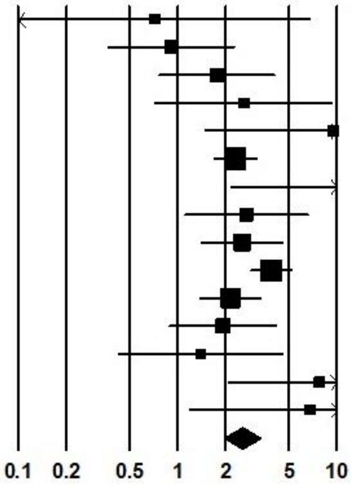

PVD

Study name	Odds ratio	Lower limit	Upper limit	Z-Value	p-Value
Caldas, 2019	0.458	0.051	4.107	-0.697	0.486
Leenders, 2018	1.982	0.881	4.459	1.653	0.098
Coffey, 1983	0.982	0.232	4.160	-0.025	0.980
Humphreys, 2016	1.130	0.417	3.065	0.240	0.810
Kazmierski, 2014a	2.295	0.905	5.816	1.751	0.080
Kazmierski, 2014b	1.871	0.616	5.683	1.105	0.269
Mariscalco, 2012	1.498	0.910	2.465	1.589	0.112
Martin, 2012	2.159	1.765	2.642	7.478	0.000
Nikolic, 2012	10.733	3.963	29.068	4.669	0.000
Norkeine, 2007	2.790	1.303	5.970	2.643	0.008
Otomo, 2013	3.314	1.088	10.094	2.109	0.035
Ringaitiene, 2015	2.130	0.468	9.689	0.978	0.328
Sevuk, 2015	0.854	0.253	2.881	-0.254	0.799
Tully, 2010	1.128	0.397	3.203	0.226	0.821
	1.977	1.482	2.637	4.636	0.000

Odds ratio and 95% CI

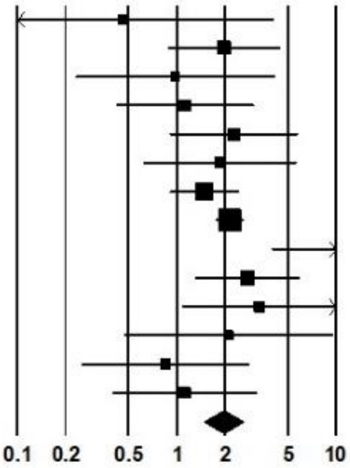

Smoking current

Study name	Odds ratio	Lower limit	Upper limit	Z-Value	p-Value
Caldas, 2019	0.343	0.069	1.697	-1.312	0.190
Chen, 2017	0.442	0.212	0.921	-2.179	0.029
Dong, 2014	0.974	0.449	2.113	-0.066	0.948
Kazmierski, 2014b	0.467	0.155	1.405	-1.355	0.175
Khan, 2014	2.886	2.016	4.132	5.789	0.000
Mardani, 2012	8.358	1.850	37.764	2.759	0.006
Martin, 2010	1.147	0.971	1.354	1.611	0.107
Norkeine, 2007	1.053	0.438	2.536	0.116	0.908
Omiya, 2015	1.600	0.237	10.809	0.482	0.630
Otomo, 2013	2.012	0.617	6.562	1.159	0.246
Rudolph, 2005	1.545	0.318	7.502	0.540	0.589
Santana Santos, 2004	1.962	0.935	4.118	1.781	0.075
Sevuk, 2015	0.748	0.413	1.356	-0.958	0.338
Zhang, 2015	1.115	0.649	1.918	0.395	0.693
	1.194	0.841	1.694	0.992	0.321

Odds ratio and 95% CI

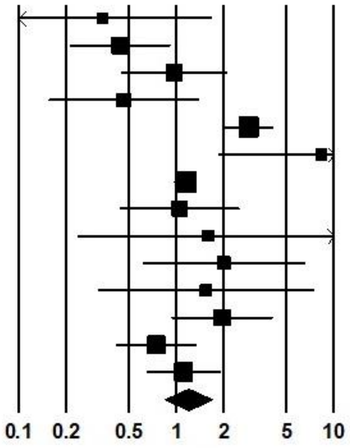

Smoking current/history

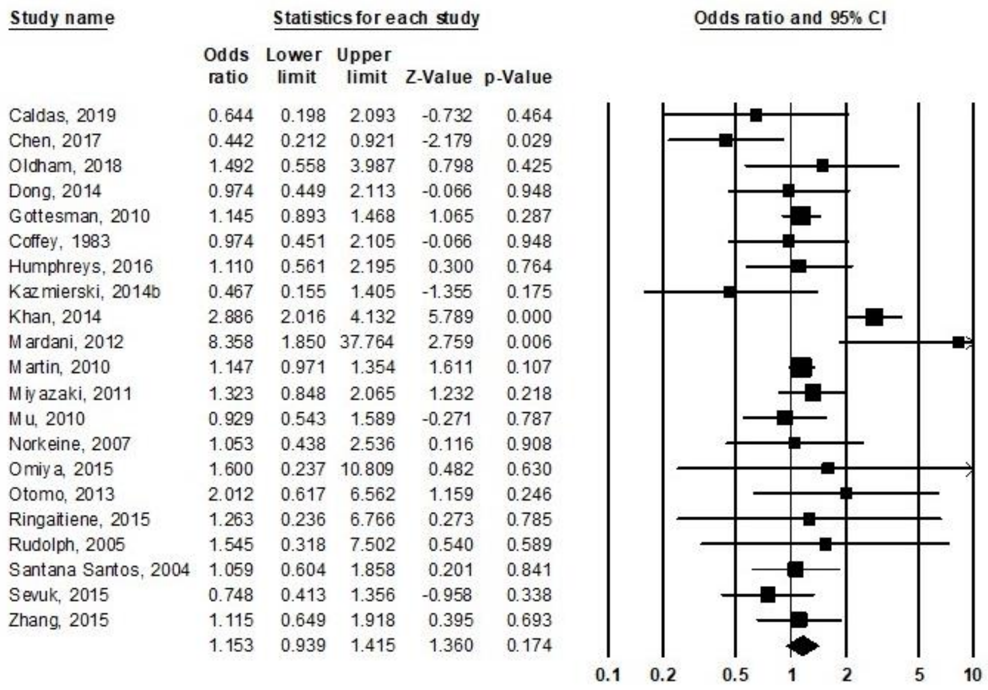

Study name	Odds ratio	Lower limit	Upper limit	Z-Value	p-Value
Caldas, 2019	0.644	0.198	2.093	-0.732	0.464
Chen, 2017	0.442	0.212	0.921	-2.179	0.029
Oldham, 2018	1.492	0.558	3.987	0.798	0.425
Dong, 2014	0.974	0.449	2.113	-0.066	0.948
Gottesman, 2010	1.145	0.893	1.468	1.065	0.287
Coffey, 1983	0.974	0.451	2.105	-0.066	0.948
Humphreys, 2016	1.110	0.561	2.195	0.300	0.764
Kazmierski, 2014b	0.467	0.155	1.405	-1.355	0.175
Khan, 2014	2.886	2.016	4.132	5.789	0.000
Mardani, 2012	8.358	1.850	37.764	2.759	0.006
Martin, 2010	1.147	0.971	1.354	1.611	0.107
Miyazaki, 2011	1.323	0.848	2.065	1.232	0.218
Mu, 2010	0.929	0.543	1.589	-0.271	0.787
Norkeine, 2007	1.053	0.438	2.536	0.116	0.908
Omiya, 2015	1.600	0.237	10.809	0.482	0.630
Otomo, 2013	2.012	0.617	6.562	1.159	0.246
Ringaitiene, 2015	1.263	0.236	6.766	0.273	0.785
Rudolph, 2005	1.545	0.318	7.502	0.540	0.589
Santana Santos, 2004	1.059	0.604	1.858	0.201	0.841
Sevuk, 2015	0.748	0.413	1.356	-0.958	0.338
Zhang, 2015	1.115	0.649	1.918	0.395	0.693
	1.153	0.939	1.415	1.360	0.174

Pre-Operative (Continuous)

Age (years) *

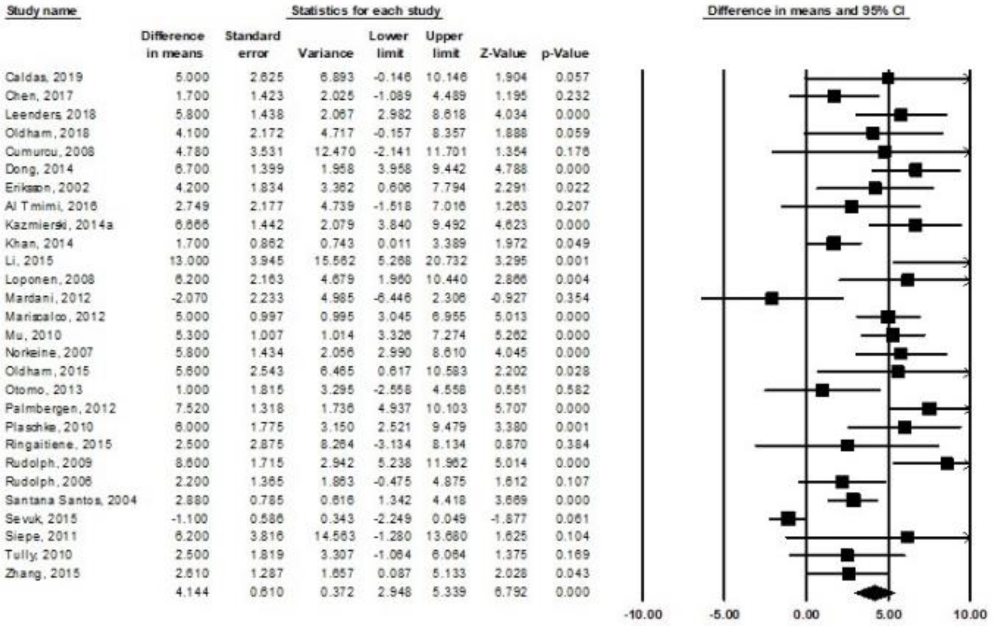

Study name	Difference in means	Standard error	Variance	Lower limit	Upper limit	Z-Value	p-Value
Caldas, 2019	5.000	2.625	6.893	-0.146	10.146	1.904	0.057
Chen, 2017	1.700	1.423	2.025	-1.089	4.489	1.195	0.232
Leenders, 2018	5.800	1.438	2.067	2.982	8.618	4.034	0.000
Oldham, 2018	4.100	2.172	4.717	-0.157	8.357	1.888	0.059
Cumurcu, 2008	4.780	3.531	12.470	-2.141	11.701	1.354	0.176
Dong, 2014	6.700	1.399	1.958	3.958	9.442	4.788	0.000
Eriksson, 2002	4.200	1.834	3.362	0.606	7.794	2.291	0.022
Al Tmimi, 2016	2.749	2.177	4.739	-1.518	7.016	1.263	0.207
Kazmierski, 2014a	6.666	1.442	2.079	3.840	9.492	4.623	0.000
Khan, 2014	1.700	0.862	0.743	0.011	3.389	1.972	0.049
Li, 2015	13.000	3.945	15.562	5.268	20.732	3.295	0.001
Loponen, 2008	6.200	2.163	4.679	1.960	10.440	2.866	0.004
Mardani, 2012	-2.070	2.233	4.985	-6.446	2.306	-0.927	0.354
Mariscalco, 2012	5.000	0.997	0.995	3.045	6.955	5.013	0.000
Mu, 2010	5.300	1.007	1.014	3.326	7.274	5.262	0.000
Norkeine, 2007	5.800	1.434	2.056	2.990	8.610	4.045	0.000
Oldham, 2015	5.600	2.543	6.465	0.617	10.583	2.202	0.028
Otomo, 2013	1.000	1.815	3.295	-2.558	4.558	0.551	0.582
Palmbergen, 2012	7.520	1.318	1.736	4.937	10.103	5.707	0.000
Plaschke, 2010	6.000	1.775	3.150	2.521	9.479	3.380	0.001
Ringaitiene, 2015	2.500	2.875	8.264	-3.134	8.134	0.870	0.384
Rudolph, 2009	8.600	1.715	2.942	5.238	11.962	5.014	0.000
Rudolph, 2006	2.200	1.365	1.863	-0.475	4.875	1.612	0.107
Santana Santos, 2004	2.880	0.785	0.616	1.342	4.418	3.669	0.000
Sevuk, 2015	-1.100	0.586	0.343	-2.249	0.049	-1.877	0.061
Siepe, 2011	6.200	3.816	14.563	-1.280	13.680	1.625	0.104
Tully, 2010	2.500	1.819	3.307	-1.064	6.064	1.375	0.169
Zhang, 2015	2.610	1.287	1.657	0.087	5.133	2.028	0.043
	4.144	0.610	0.372	2.948	5.339	6.792	0.000

BMI

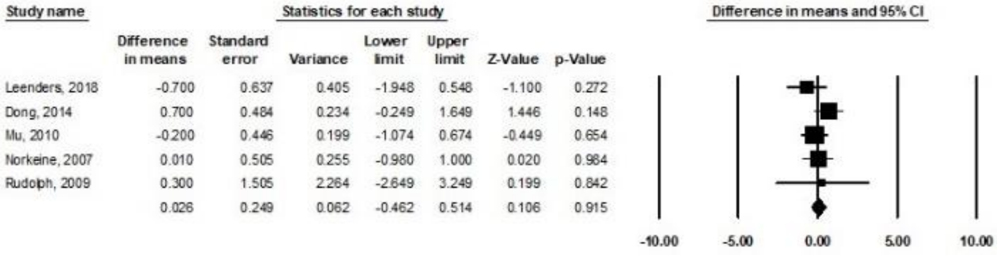

Study name	Difference in means	Standard error	Variance	Lower limit	Upper limit	Z-Value	p-Value
Leenders, 2018	-0.700	0.637	0.405	-1.948	0.548	-1.100	0.272
Dong, 2014	0.700	0.484	0.234	-0.249	1.649	1.446	0.148
Mu, 2010	-0.200	0.446	0.199	-1.074	0.674	-0.449	0.654
Norkeine, 2007	0.010	0.505	0.255	-0.980	1.000	0.020	0.984
Rudolph, 2009	0.300	1.505	2.264	-2.649	3.249	0.199	0.842
	0.026	0.249	0.062	-0.462	0.514	0.106	0.915

Cognition: All tests

Study name	Std diff in means	Standard error	Variance	Lower limit	Upper limit	Z-Value	p-Value
Caldas, 2019	-1.059	0.295	0.087	-1.638	-0.481	-3.588	0.000
Chen, 2017	-0.316	0.184	0.034	-0.677	0.046	-1.712	0.087
Oldham, 2018	-0.445	0.240	0.057	-0.914	0.025	-1.855	0.064
Al Tmimi, 2016	0.000	0.258	0.066	-0.505	0.505	0.000	1.000
Kazmierski, 2014a	-0.737	0.202	0.041	-1.132	-0.342	-3.654	0.000
Li, 2015	-0.912	0.464	0.215	-1.821	-0.004	-1.968	0.049
Oldham, 2015	-0.784	0.293	0.086	-1.359	-0.210	-2.678	0.007
Otomo, 2013	-0.772	0.268	0.072	-1.297	-0.247	-2.880	0.004
Rudolph, 2009	-0.572	0.248	0.061	-1.057	-0.086	-2.309	0.021
	-0.576	0.105	0.011	-0.782	-0.370	-5.481	0.000

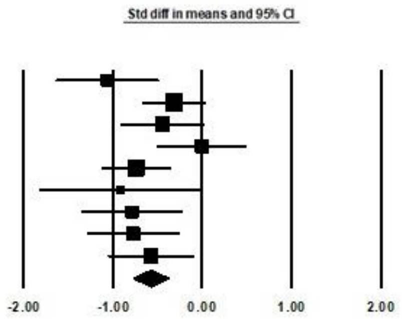

Cognition: MMSE only

Study name	Difference in means	Standard error	Variance	Lower limit	Upper limit	Z-Value	p-Value
Caldas, 2019	-4.627	1.226	1.503	-7.030	-2.224	-3.774	0.000
Chen, 2017	-0.200	0.116	0.013	-0.428	0.028	-1.722	0.085
Oldham, 2018	-0.900	0.482	0.232	-1.845	0.045	-1.867	0.062
Al Tmimi, 2016	0.000	0.394	0.155	-0.773	0.773	0.000	1.000
Li, 2015	-3.300	1.611	2.595	-6.457	-0.143	-2.048	0.041
Oldham, 2015	-1.600	0.587	0.344	-2.750	-0.450	-2.726	0.006
Rudolph, 2009	-1.400	0.594	0.353	-2.565	-0.235	-2.356	0.018
	-1.136	0.394	0.155	-1.907	-0.364	-2.884	0.004

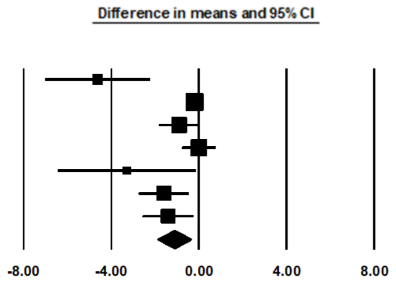

Depression GDS

Study name	Difference in means	Standard error	Variance	Lower limit	Upper limit	Z-Value	p-Value
Oldham, 2018	1.000	0.599	0.359	-0.174	2.174	1.669	0.095
Oldham, 2015	0.400	0.716	0.513	-1.004	1.804	0.559	0.576
	0.753	0.459	0.211	-0.148	1.654	1.639	0.101

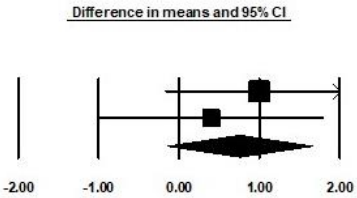

Education (years)

Study name	Difference in means	Standard error	Variance	Lower limit	Upper limit	Z-Value	p-Value
Caldas, 2019	-1.361	1.684	2.837	-4.663	1.940	-0.808	0.419
Kazmierski, 2014c	-1.761	0.549	0.302	-2.838	-0.684	-3.206	0.001
Kazmierski, 2014b	-1.220	0.878	0.772	-2.941	0.502	-1.388	0.165
Li, 2015	1.300	2.290	5.242	-3.188	5.788	0.568	0.570
Mu, 2010	0.000	0.558	0.312	-1.094	1.094	0.000	1.000
Otomo, 2013	-1.000	0.748	0.559	-2.466	0.466	-1.337	0.181
	-0.926	0.370	0.137	-1.652	-0.200	-2.499	0.012

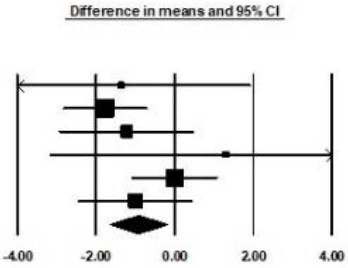

EuroSCORE

Study name	Difference in means	Standard error	Variance	Lower limit	Upper limit	Z-Value	p-Value
Al Tmimi, 2016	0.305	0.248	0.061	-0.180	0.791	1.233	0.218
Caldas, 2019	0.992	0.652	0.426	-0.286	2.271	1.521	0.128
Dong, 2014	0.500	0.198	0.039	0.111	0.889	2.519	0.012
Loponen, 2008	1.800	0.771	0.594	0.289	3.311	2.335	0.020
Martin, 2012	5.600	0.442	0.196	4.733	6.467	12.664	0.000
Mu, 2010	1.200	0.251	0.063	0.708	1.692	4.777	0.000
Norkeine, 2007	1.700	0.379	0.143	0.958	2.442	4.489	0.000
Ringaitiene, 2015	0.000	0.542	0.294	-1.062	1.062	0.000	1.000
Sevuk, 2015	0.010	0.042	0.002	-0.073	0.093	0.236	0.814
Zhang, 2015	1.690	0.315	0.100	1.072	2.308	5.357	0.000
	1.347	0.394	0.155	0.575	2.120	3.417	0.001

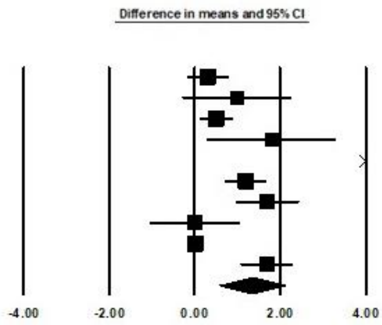

LVEF (%)

Study name	Difference in means	Standard error	Variance	Lower limit	Upper limit	Z-Value	p-Value
Caldas, 2019	-3.577	1.461	2.133	-6.440	-0.714	-2.449	0.014
Chen, 2017	2.300	1.657	2.745	-0.948	5.548	1.388	0.165
Kazmierski, 2014c	-0.054	1.882	3.540	-3.742	3.634	-0.029	0.977
Kazmierski, 2014b	0.398	2.292	5.255	-4.096	4.891	0.173	0.862
Khan, 2014	-2.490	0.870	0.756	-4.194	-0.786	-2.863	0.004
Mardani, 2012	0.120	0.922	0.849	-1.686	1.926	0.130	0.896
Mu, 2010	2.700	1.222	1.492	0.306	5.094	2.210	0.027
Norkeine, 2007	6.700	1.455	2.118	3.848	9.552	4.604	0.000
Ringaitiene, 2015	3.900	2.524	6.372	-1.047	8.847	1.545	0.122
Rudolph, 2009	1.000	2.426	5.887	-3.756	5.756	0.412	0.680
Santana Santos, 2004	4.000	1.725	2.975	0.619	7.381	2.319	0.020
	1.247	0.990	0.979	-0.692	3.187	1.260	0.208

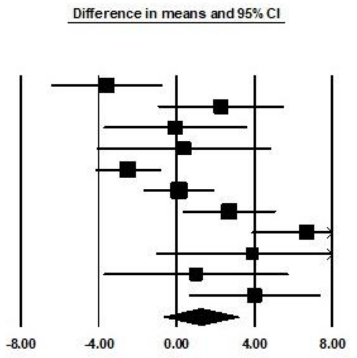

Intra-Operative (Continuous)

ACC time (mins)

Study name	Difference in means	Standard error	Variance	Lower limit	Upper limit	Z-Value	p-Value
Caldas, 2019	-13.000	6.766	45.776	-26.261	0.261	-1.921	0.055
Leenders, 2018	6.100	2.598	6.749	1.008	11.192	2.348	0.019
Cumurcu, 2008	18.940	8.191	67.093	2.886	34.994	2.312	0.021
Eriksson, 2002	4.300	5.280	27.879	-6.049	14.649	0.814	0.415
Kazmierski, 2014a	4.012	2.586	6.687	-1.056	9.081	1.552	0.121
Kazmierski, 2014b	5.534	2.703	7.307	0.236	10.832	2.047	0.041
Khan, 2014	28.220	2.004	4.017	24.292	32.148	14.081	0.000
Loponen, 2008	8.000	6.821	46.528	-5.369	21.369	1.173	0.241
Mardani, 2012	2.000	2.182	4.760	-2.276	6.276	0.917	0.359
Mariscalco, 2012	19.800	3.501	12.260	12.937	26.663	5.655	0.000
Nikolic, 2012	1.177	2.456	6.032	-3.637	5.991	0.479	0.632
Norkeine, 2007	3.100	3.378	11.414	-3.522	9.722	0.918	0.359
Ringaitiene, 2015	-5.500	6.913	47.789	-19.049	8.049	-0.796	0.426
Santana Santos, 2004	2.040	3.579	12.810	-4.975	9.055	0.570	0.569
Sevuk, 2015	-0.733	3.151	9.930	-6.910	5.443	-0.233	0.816
Tully, 2010	8.100	3.994	15.956	0.271	15.929	2.028	0.043
	5.970	2.732	7.462	0.616	11.324	2.185	0.029

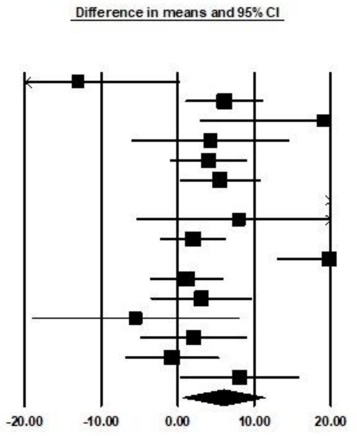

CPB time (mins)

Study name	Difference in means	Standard error	Variance	Lower limit	Upper limit	Z-Value	p-Value
Caldas, 2019	-7.000	7.876	62.035	-22.437	8.437	-0.889	0.374
Cumurcu, 2008	15.260	8.490	72.077	-1.380	31.900	1.797	0.072
Eriksson, 2002	8.900	9.834	96.701	-10.374	28.174	0.905	0.365
Gottesman, 2010	5.100	1.922	3.695	1.333	8.867	2.653	0.008
Kazmierski, 2014a	12.660	3.839	14.740	5.135	20.184	3.297	0.001
Kazmierski, 2014b	15.349	4.966	24.663	5.615	25.082	3.091	0.002
Khan, 2014	2.100	3.316	10.995	-4.399	8.599	0.633	0.527
Leenders, 2018	0.764	5.346	28.576	-9.713	11.241	0.143	0.886
Li, 2015	46.200	45.417	2062.711	-42.816	135.216	1.017	0.309
Loponen, 2008	10.000	7.903	62.462	-5.490	25.490	1.265	0.206
Mardani, 2012	2.000	3.442	11.847	-4.746	8.746	0.581	0.561
Mariscalco, 2012	25.700	4.450	19.799	16.979	34.421	5.776	0.000
Nikolic, 2012	9.073	3.045	9.272	3.105	15.041	2.980	0.003
Norkeine, 2007	11.800	6.069	36.837	-0.096	23.696	1.944	0.052
Oldham, 2018	-3.000	9.730	94.681	-22.071	16.071	-0.308	0.758
Otomo, 2013	21.000	16.018	256.565	-10.394	52.394	1.311	0.190
Ringaitiene, 2015	-0.100	10.799	116.611	-21.265	21.065	-0.009	0.993
Rudolph, 2009	2.000	6.794	46.158	-11.316	15.316	0.294	0.768
Santana Santos, 2004	3.970	5.280	27.873	-6.378	14.318	0.752	0.452
Sevuk, 2015	0.282	4.949	24.492	-9.418	9.982	0.057	0.955
Siepe, 2011	20.000	11.678	136.385	-2.889	42.889	1.713	0.087
	7.406	1.724	2.972	4.027	10.784	4.296	0.000

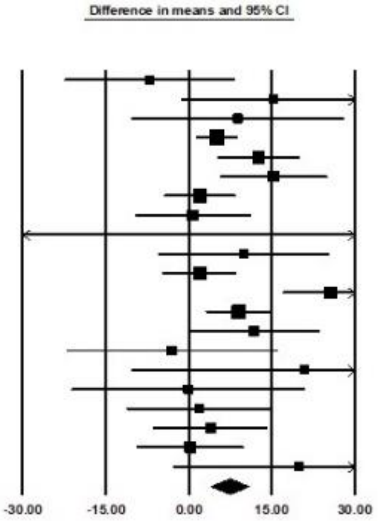

Duration of surgery (mins)	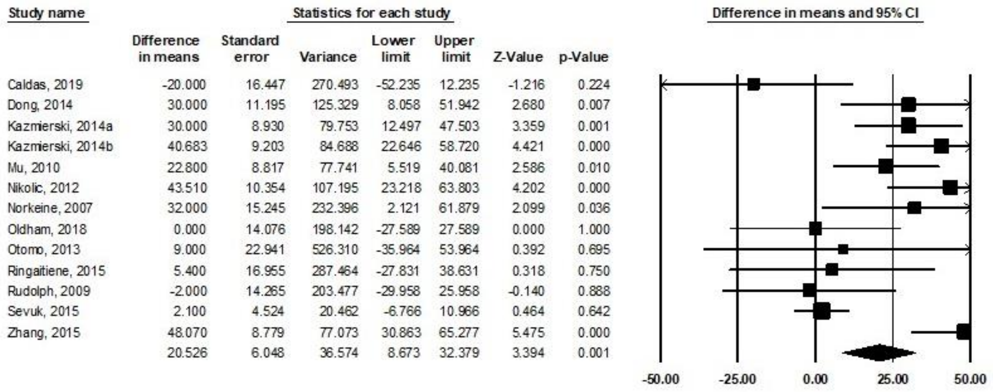

Study name	Difference in means	Standard error	Variance	Lower limit	Upper limit	Z-Value	p-Value
Caldas, 2019	-20.000	16.447	270.493	-52.235	12.235	-1.216	0.224
Dong, 2014	30.000	11.195	125.329	8.058	51.942	2.680	0.007
Kazmierski, 2014a	30.000	8.930	79.753	12.497	47.503	3.359	0.001
Kazmierski, 2014b	40.683	9.203	84.688	22.646	58.720	4.421	0.000
Mu, 2010	22.800	8.817	77.741	5.519	40.081	2.586	0.010
Nikolic, 2012	43.510	10.354	107.195	23.218	63.803	4.202	0.000
Norkeine, 2007	32.000	15.245	232.396	2.121	61.879	2.099	0.036
Oldham, 2018	0.000	14.076	198.142	-27.589	27.589	0.000	1.000
Otomo, 2013	9.000	22.941	526.310	-35.964	53.964	0.392	0.695
Ringaitiene, 2015	5.400	16.955	287.464	-27.831	38.631	0.318	0.750
Rudolph, 2009	-2.000	14.265	203.477	-29.958	25.958	-0.140	0.888
Sevuk, 2015	2.100	4.524	20.462	-6.766	10.966	0.464	0.642
Zhang, 2015	48.070	8.779	77.073	30.863	65.277	5.475	0.000
	20.526	6.048	36.574	8.673	32.379	3.394	0.001

Difference in means and 95% CI: -50.00, -25.00, 0.00, 25.00, 50.00

Intubation time (hours)	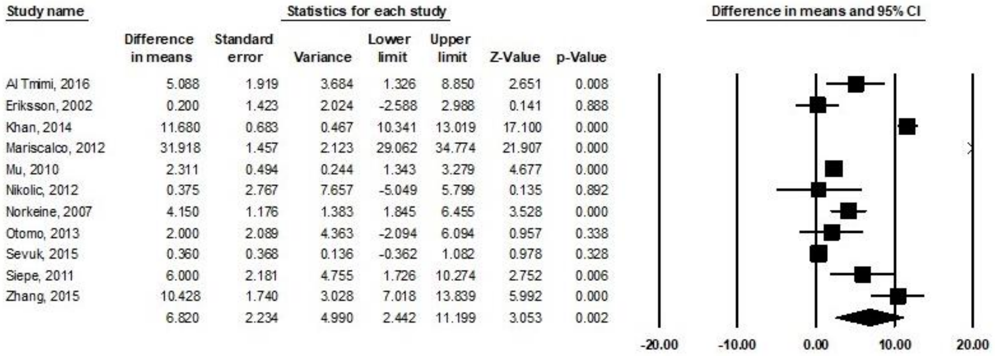

Study name	Difference in means	Standard error	Variance	Lower limit	Upper limit	Z-Value	p-Value
Al Trmimi, 2016	5.088	1.919	3.684	1.326	8.850	2.651	0.008
Eriksson, 2002	0.200	1.423	2.024	-2.588	2.988	0.141	0.888
Khan, 2014	11.680	0.683	0.467	10.341	13.019	17.100	0.000
Mariscalco, 2012	31.918	1.457	2.123	29.062	34.774	21.907	0.000
Mu, 2010	2.311	0.494	0.244	1.343	3.279	4.677	0.000
Nikolic, 2012	0.375	2.767	7.657	-5.049	5.799	0.135	0.892
Norkeine, 2007	4.150	1.176	1.383	1.845	6.455	3.528	0.000
Otomo, 2013	2.000	2.089	4.363	-2.094	6.094	0.957	0.338
Sevuk, 2015	0.360	0.368	0.136	-0.362	1.082	0.978	0.328
Siepe, 2011	6.000	2.181	4.755	1.726	10.274	2.752	0.006
Zhang, 2015	10.428	1.740	3.028	7.018	13.839	5.992	0.000
	6.820	2.234	4.990	2.442	11.199	3.053	0.002

Difference in means and 95% CI: -20.00, -10.00, 0.00, 10.00, 20.00

Number of grafts	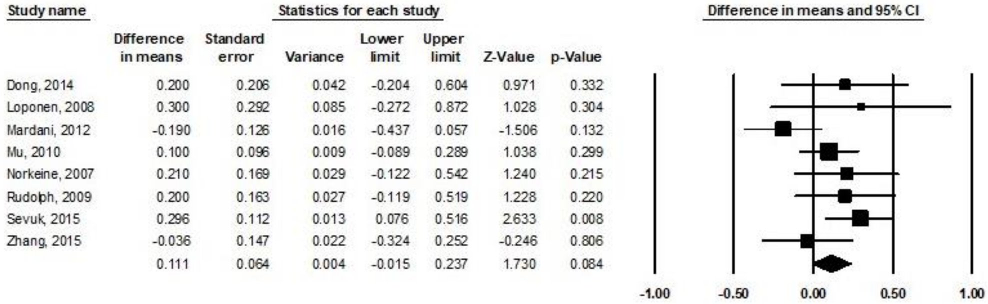

Study name	Difference in means	Standard error	Variance	Lower limit	Upper limit	Z-Value	p-Value
Dong, 2014	0.200	0.206	0.042	-0.204	0.604	0.971	0.332
Loponen, 2008	0.300	0.292	0.085	-0.272	0.872	1.028	0.304
Mardani, 2012	-0.190	0.126	0.016	-0.437	0.057	-1.506	0.132
Mu, 2010	0.100	0.096	0.009	-0.089	0.289	1.038	0.299
Norkeine, 2007	0.210	0.169	0.029	-0.122	0.542	1.240	0.215
Rudolph, 2009	0.200	0.163	0.027	-0.119	0.519	1.228	0.220
Sevuk, 2015	0.296	0.112	0.013	0.076	0.516	2.633	0.008
Zhang, 2015	-0.036	0.147	0.022	-0.324	0.252	-0.246	0.806
	0.111	0.064	0.004	-0.015	0.237	1.730	0.084

Difference in means and 95% CI: -1.00, -0.50, 0.00, 0.50, 1.00

Post-Operative (Categorical)

Arrhythmia, incl. AF	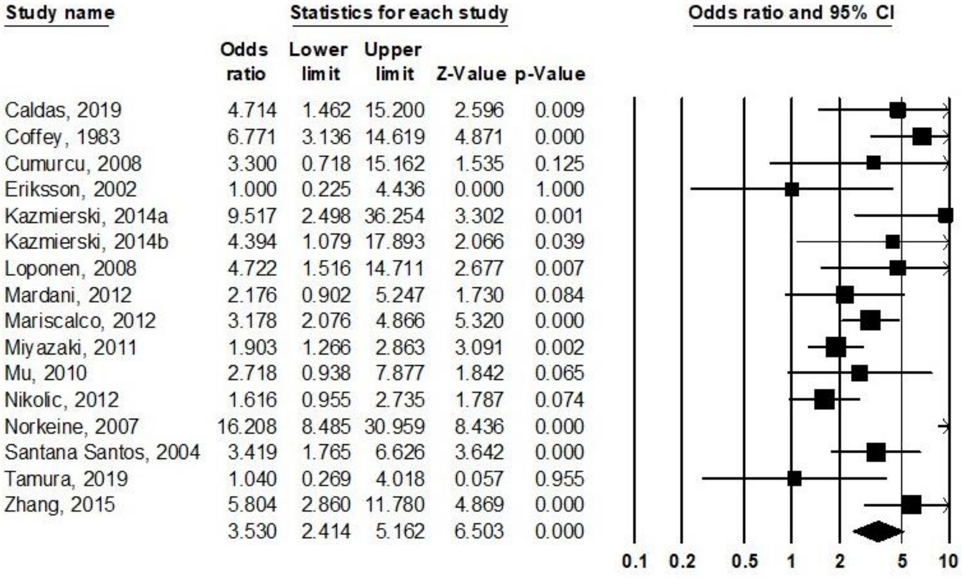

Study name	Odds ratio	Lower limit	Upper limit	Z-Value	p-Value
Caldas, 2019	4.714	1.462	15.200	2.596	0.009
Coffey, 1983	6.771	3.136	14.619	4.871	0.000
Cumurcu, 2008	3.300	0.718	15.162	1.535	0.125
Eriksson, 2002	1.000	0.225	4.436	0.000	1.000
Kazmierski, 2014a	9.517	2.498	36.254	3.302	0.001
Kazmierski, 2014b	4.394	1.079	17.893	2.066	0.039
Loponen, 2008	4.722	1.516	14.711	2.677	0.007
Mardani, 2012	2.176	0.902	5.247	1.730	0.084
Mariscalco, 2012	3.178	2.076	4.866	5.320	0.000
Miyazaki, 2011	1.903	1.266	2.863	3.091	0.002
Mu, 2010	2.718	0.938	7.877	1.842	0.065
Nikolic, 2012	1.616	0.955	2.735	1.787	0.074
Norkeine, 2007	16.208	8.485	30.959	8.436	0.000
Santana Santos, 2004	3.419	1.765	6.626	3.642	0.000
Tamura, 2019	1.040	0.269	4.018	0.057	0.955
Zhang, 2015	5.804	2.860	11.780	4.869	0.000
	3.530	2.414	5.162	6.503	0.000

Odds ratio and 95% CI: 0.1, 0.2, 0.5, 1, 2, 5, 10

**Post-Operative (Continuous)**	
LOS in ICU (days)	See forest plot below

Study name	Difference in means	Standard error	Variance	Lower limit	Upper limit	Z-Value	p-Value
Al Tmimi, 2016	1.491	0.339	0.115	0.826	2.155	4.396	0.000
Caldas, 2019	2.910	1.833	3.361	-0.683	6.503	1.587	0.112
Chen, 2017	2.253	0.272	0.074	1.721	2.786	8.295	0.000
Eriksson, 2002	-0.100	0.209	0.044	-0.509	0.309	-0.479	0.632
Khan, 2014	6.510	0.234	0.055	6.052	6.968	27.852	0.000
Mardani, 2012	0.530	0.251	0.063	0.037	1.023	2.108	0.035
Mariscalco, 2012	2.188	0.074	0.005	2.043	2.333	29.630	0.000
Mu, 2010	0.920	0.197	0.039	0.533	1.306	4.666	0.000
Norkeine, 2007	4.800	0.438	0.191	3.943	5.657	10.971	0.000
Omiya, 2015	1.385	0.349	0.122	0.701	2.070	3.965	0.000
Palmbergen, 2012	2.990	0.385	0.148	2.236	3.744	7.773	0.000
Siepe, 2011	1.500	1.381	1.906	-1.206	4.206	1.087	0.277
Subramaniam, 2019	1.412	0.353	0.125	0.720	2.105	3.996	0.000
Zhang, 2015	2.500	0.311	0.097	1.890	3.110	8.035	0.000
	2.221	0.462	0.213	1.316	3.126	4.812	0.000

Difference in means and 95% CI

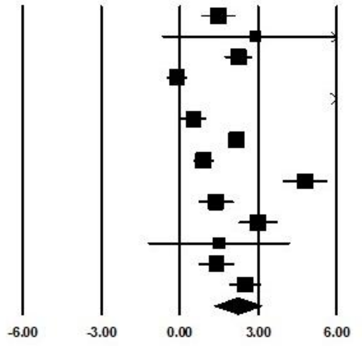

**Figure S2. Funnel plots for statistically significant analyses in regard to delirium post-CABG, and results of publication bias/small-study effect investigation when more than 10 studies were available.**	**Egger's Test**		**Trim and Fill**		
	**Intercept**	**p value (1-tailed)**	**No. imputed studies**	**OR/MD† /SMD‡**	**95%CI**
**Preoperative (Categorical)**					
Arrhythmia, incl. AF 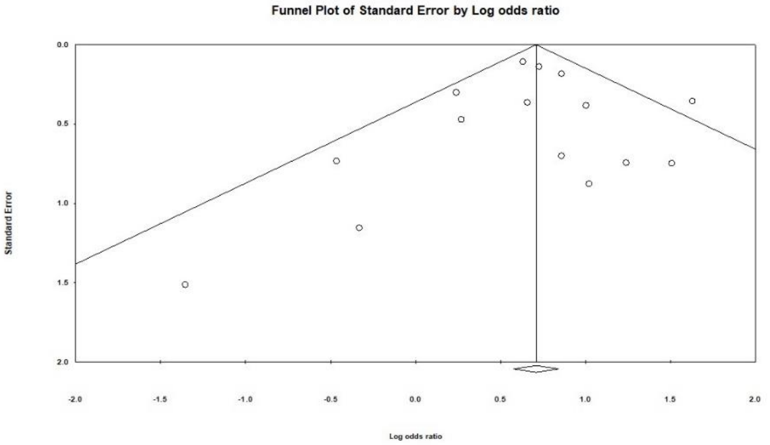	-0.08	.432	-	-	-
BMI ≥30 only 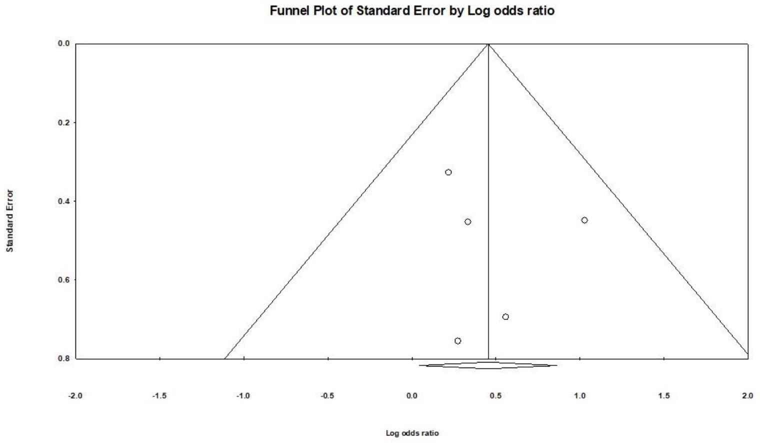	-	-	-	-	-

Cognitive Impairment	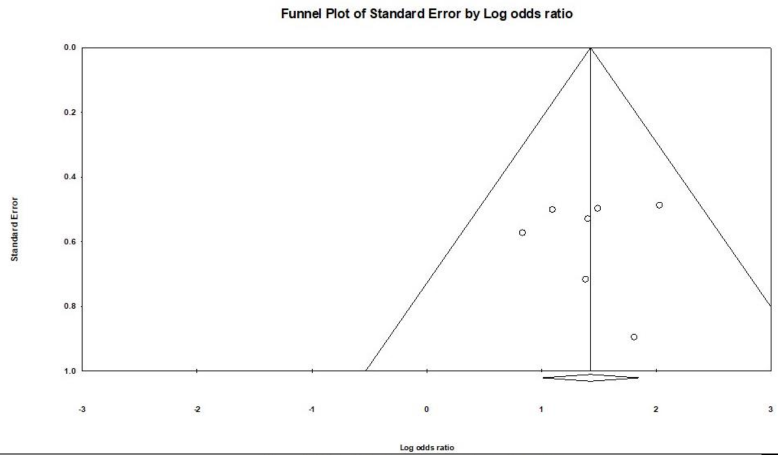	-	-	-	-	-
Depression	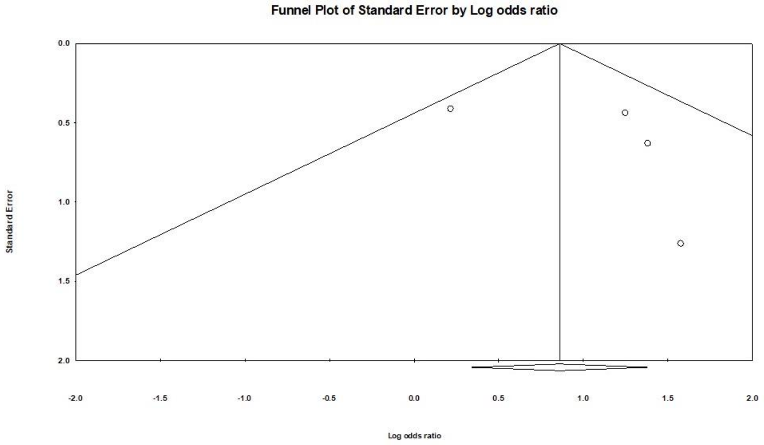	-	-	-	-	-

Diabetes	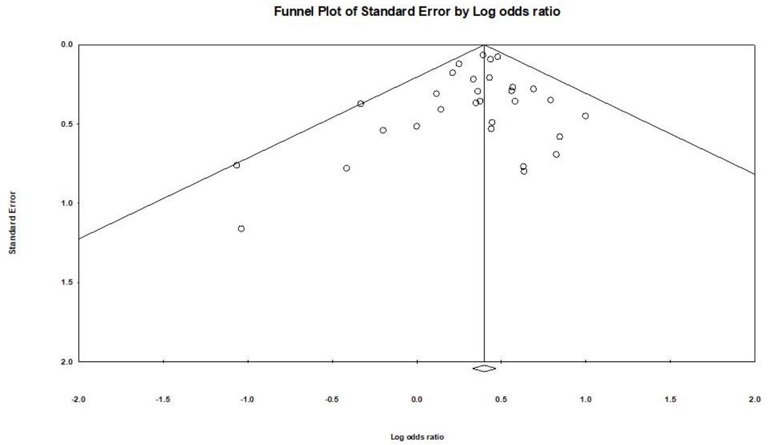	-0.27	.143	-	-	-
Hypertension	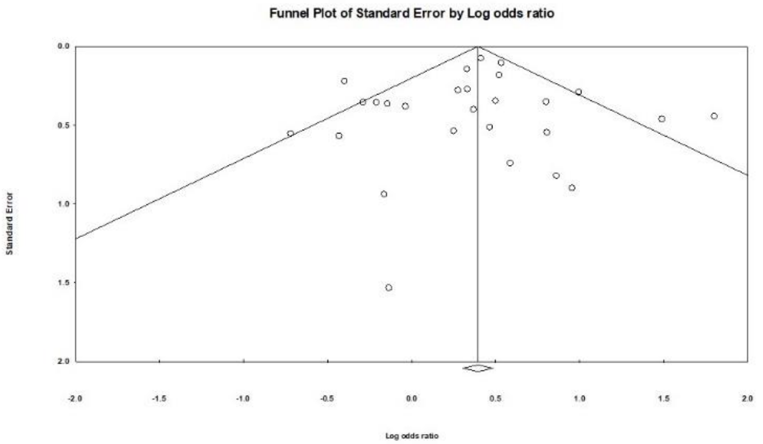	-0.18	.347	-	-	-

Kidney Injury	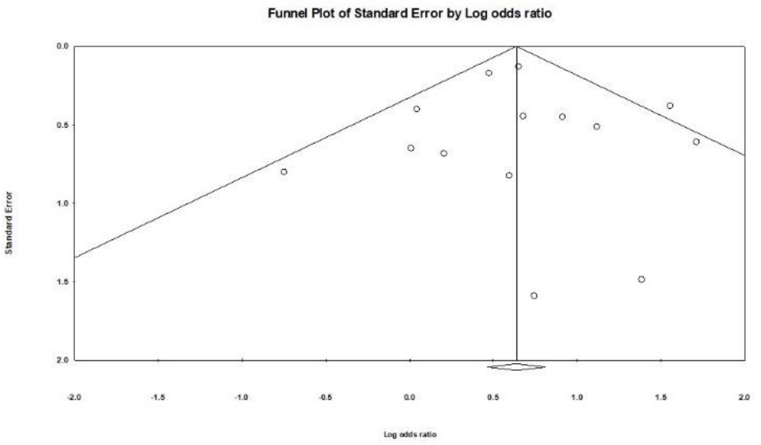	0.13	.406	-	-	-
Previous stroke, TIA, CVA	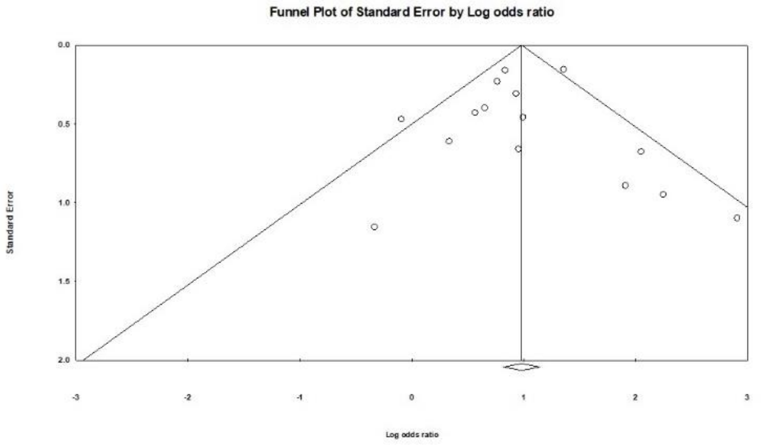	-0.03	.483	-	-	-

PVD	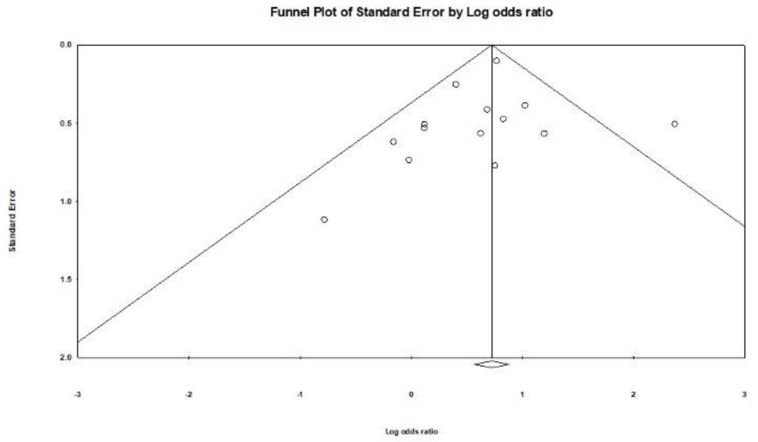	-0.35	.266	-	-	-
**Preoperative (Continuous)**						
Age	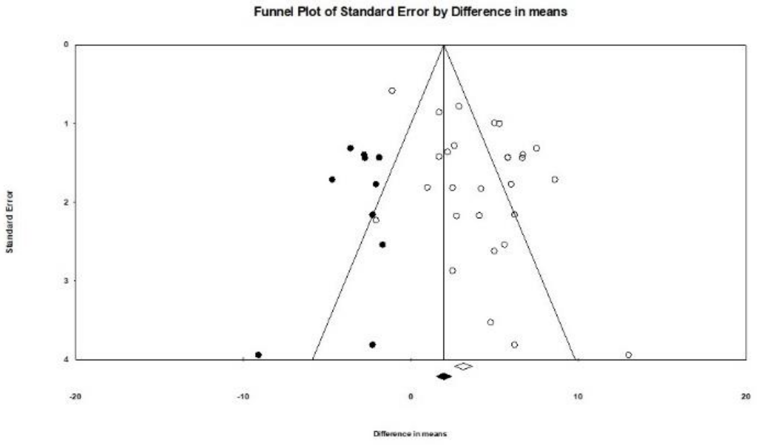	2.44	.003	11	2.24†	1.04-3.45

Cognition: All tests	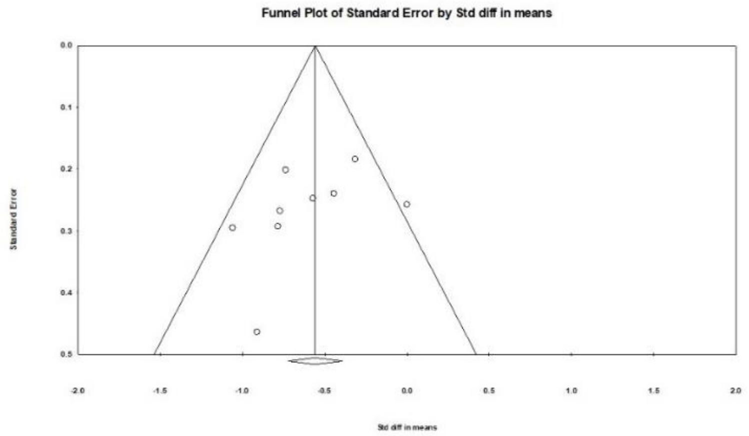	-	-	-	-	-
Cognition: MMSE only	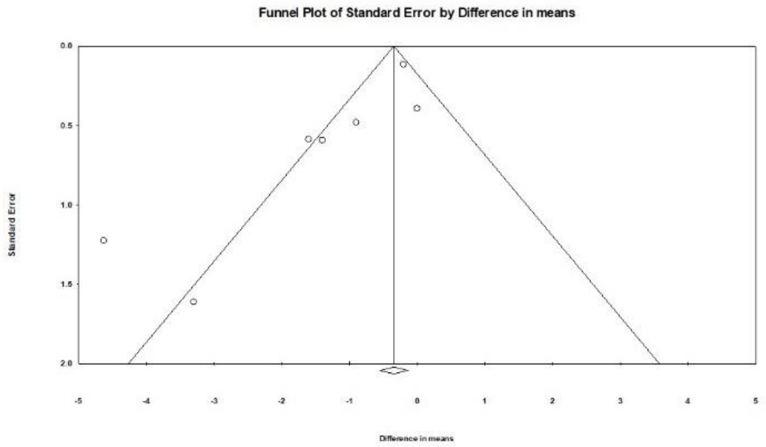	-	-	-	-	

Education (years)	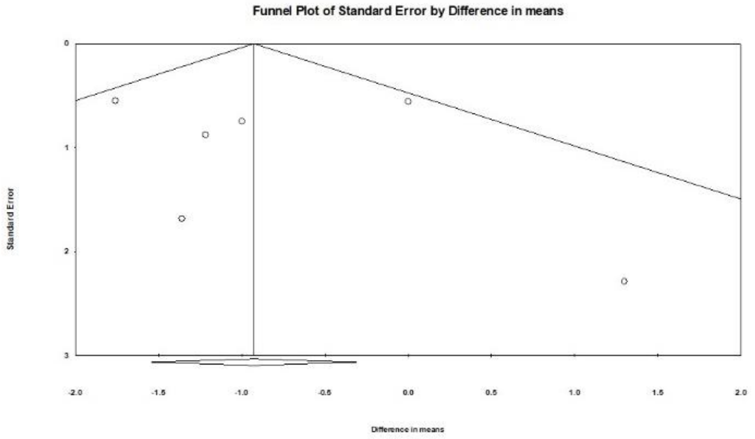	-	-	-	-	
EuroSCORE	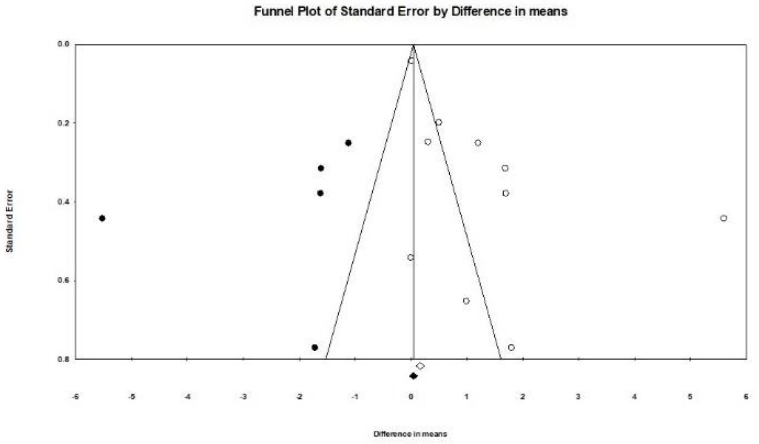	4.36	.010	5	0.15†	-0.60-0.90
**Intra-Operative (Continuous)**						

ACC time (mins)	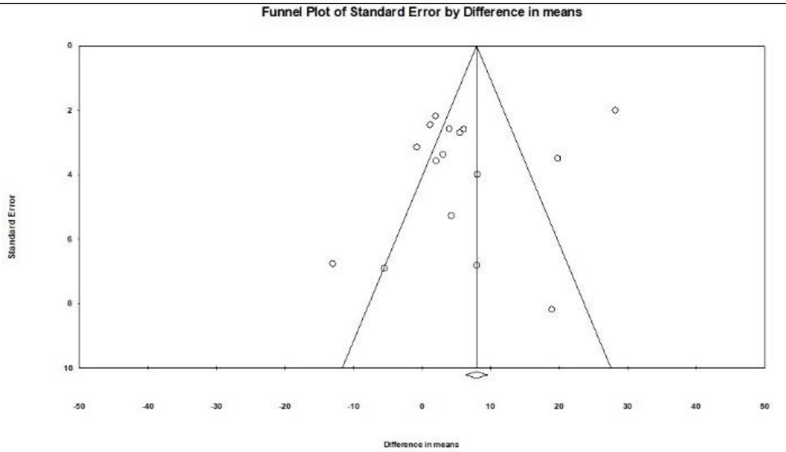	-2.45	.143	-	-	-
CPB time (mins)	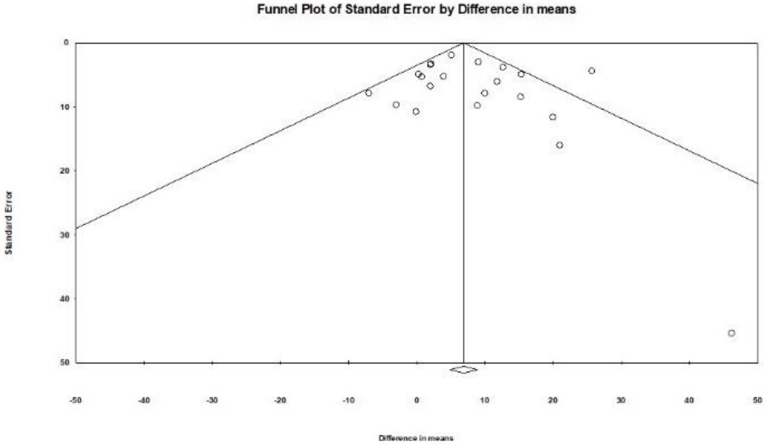	0.45	.241	-	-	-

Duration of surgery (mins)	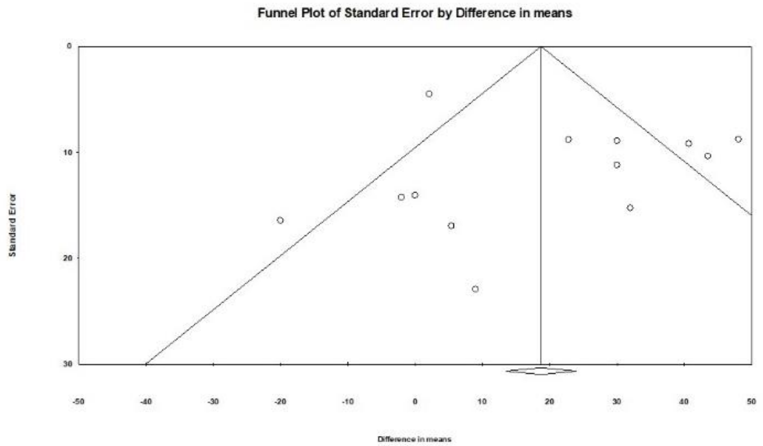	0.77	.296	-	-	-
Intubation time (hours)	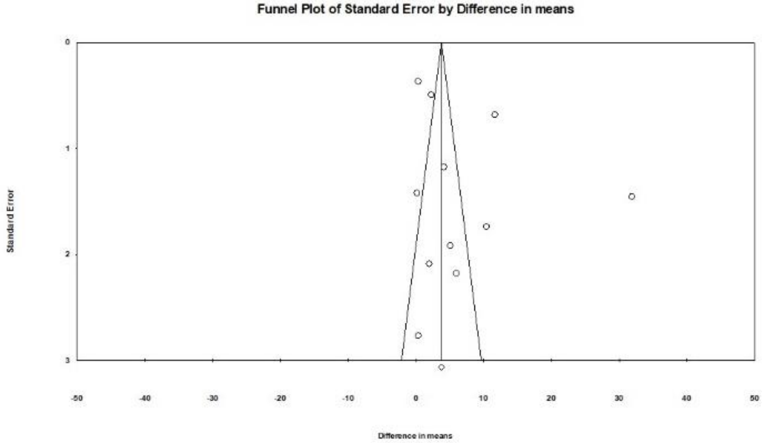	5.20	.106	-	-	-
**Post-Operative (Categorical)**						

Arrhythmia, incl. AF 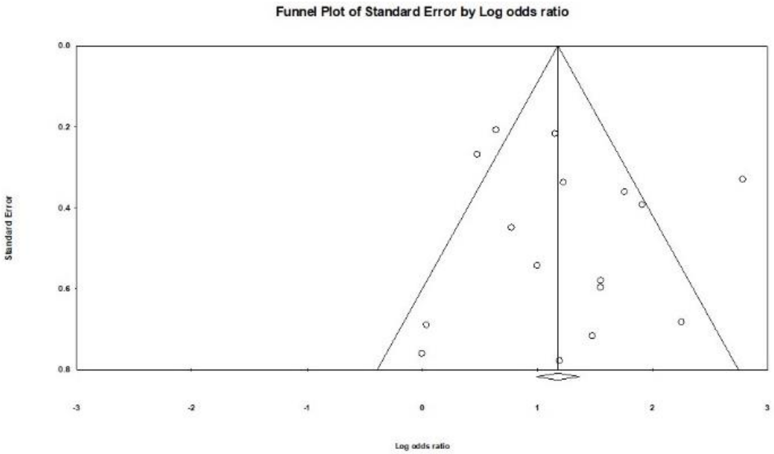	0.76	.260	-	-	
**Post-Operative (Continuous)**					
LOS in ICU (days) 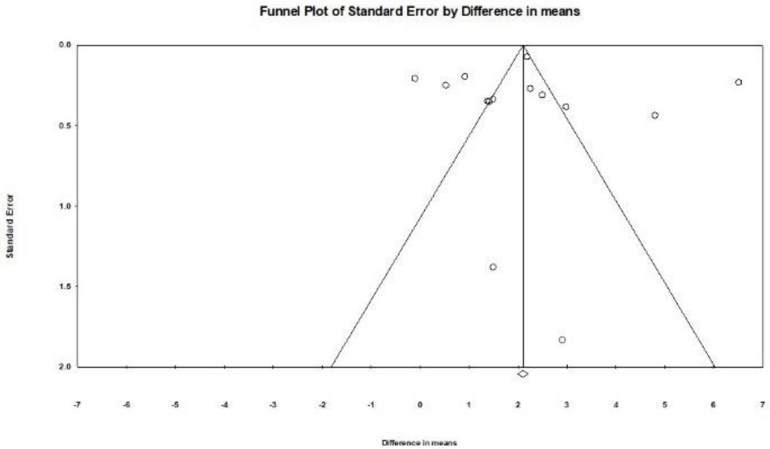	0.32	.459	-	-	-

Figure S3. Forest plots for acute cognitive decline post-CABG analyses.

Variable	Forest Plot
**Pre-Operative (Categorical)**	

Arrhythmia, incl. AF

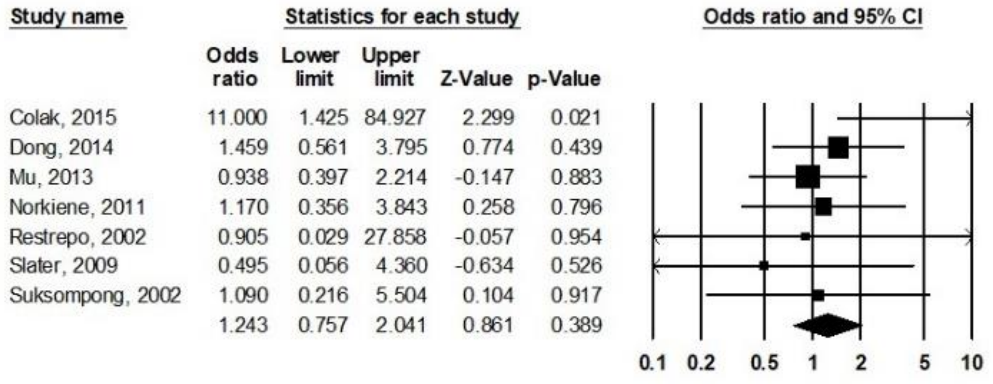

Cognitive Impairment

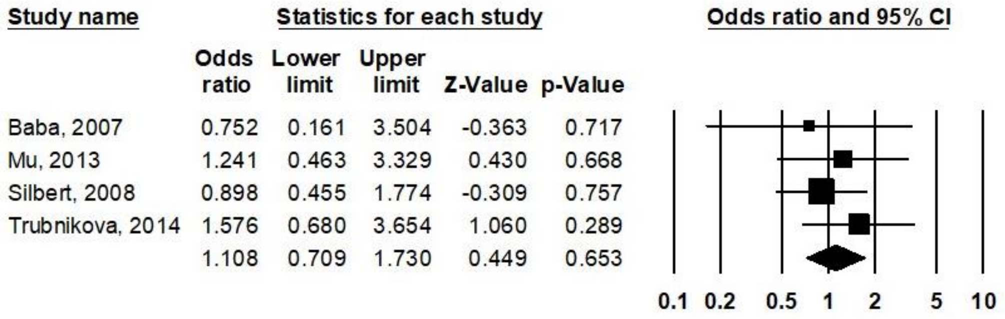

Depression

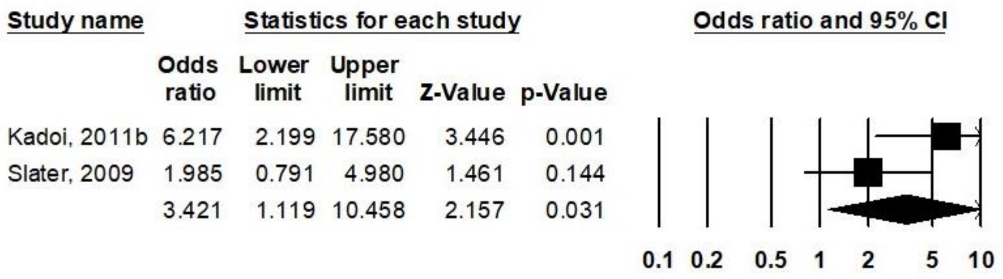

Diabetes

Study name	Odds ratio	Lower limit	Upper limit	Z-Value	p-Value
Baba, 2007	1.148	0.570	2.312	0.387	0.698
Boodhwani, 2006	1.089	0.723	1.642	0.409	0.682
Colak, 2015	1.149	0.607	2.176	0.427	0.669
deTournay-Jette, 2011	0.933	0.237	3.674	-0.099	0.921
Dong, 2014	2.810	1.151	6.860	2.268	0.023
Goto, 2000	1.717	0.686	4.299	1.155	0.248
Harmon, 2005	0.619	0.036	10.775	-0.329	0.742
Kadoi, 2005	1.683	1.016	2.788	2.020	0.043
Kumpaitiene, 2019	0.794	0.231	2.725	-0.366	0.714
Mu, 2013	2.395	1.244	4.609	2.614	0.009
Norkiene, 2011	1.367	0.464	4.027	0.567	0.571
Oh, 2008	1.676	0.403	6.966	0.711	0.477
Reents, 2002	1.901	0.542	6.675	1.003	0.316
Restrepo, 2002	0.750	0.050	11.311	-0.208	0.835
Slater, 2009	0.834	0.483	1.442	-0.648	0.517
Suksompong, 2002	1.260	0.479	3.314	0.468	0.639
Toeg, 2013	1.950	1.391	2.733	3.878	0.000
	1.443	1.211	1.719	4.104	0.000

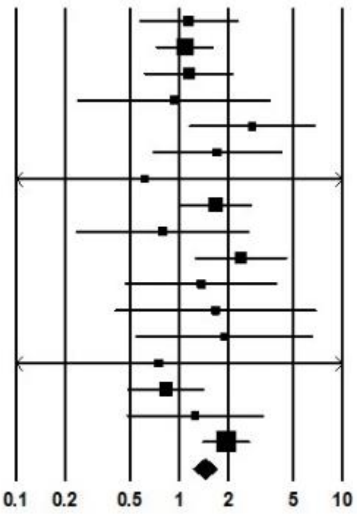

Dyslipidemia/Hyperlipidemia

Study name	Odds ratio	Lower limit	Upper limit	Z-Value	p-Value
Baba, 2007	1.079	0.539	2.161	0.214	0.830
deTournay-Jette, 2011	0.630	0.119	3.336	-0.544	0.587
Dong, 2014	1.071	0.470	2.444	0.164	0.870
Goto, 2000	0.719	0.275	1.880	-0.673	0.501
Mu, 2013	1.038	0.546	1.973	0.115	0.909
Suksompong, 2002	4.730	1.557	14.371	2.741	0.006
	1.164	0.739	1.835	0.655	0.512

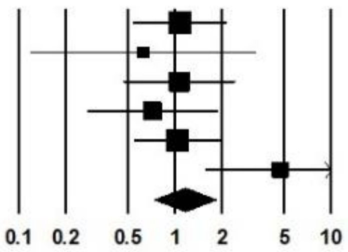

Hypertension

Study name	Odds ratio	Lower limit	Upper limit	Z-Value	p-Value
Baba, 2007	0.929	0.407	2.119	-0.176	0.860
Colak, 2015	1.541	0.629	3.774	0.946	0.344
deTournay-Jette, 2011	4.000	1.007	15.895	1.969	0.049
Dong, 2014	2.063	0.867	4.904	1.638	0.101
Goto, 2000	2.005	0.641	6.268	1.196	0.232
Harmon, 2005	1.926	0.496	7.485	0.946	0.344
Kadoi, 2001	1.100	0.074	16.316	0.069	0.945
Kadoi, 2005	6.183	2.962	12.907	4.852	0.000
Kadoi, 2011a	1.400	1.043	1.878	2.244	0.025
Kadoi, 2011b	2.059	0.887	4.778	1.681	0.093
Mu, 2013	2.283	1.178	4.425	2.446	0.014
Norkiene, 2011	1.239	0.372	4.134	0.349	0.727
Restrepo, 2002	0.500	0.029	8.706	-0.475	0.634
Slater, 2009	1.498	0.808	2.775	1.284	0.199
Suksompong, 2002	3.750	1.158	12.141	2.205	0.027
	1.914	1.447	2.530	4.555	0.000

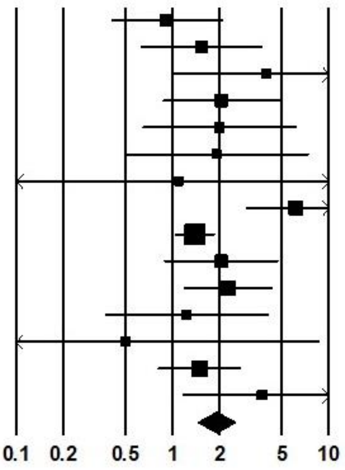

Sex (male)

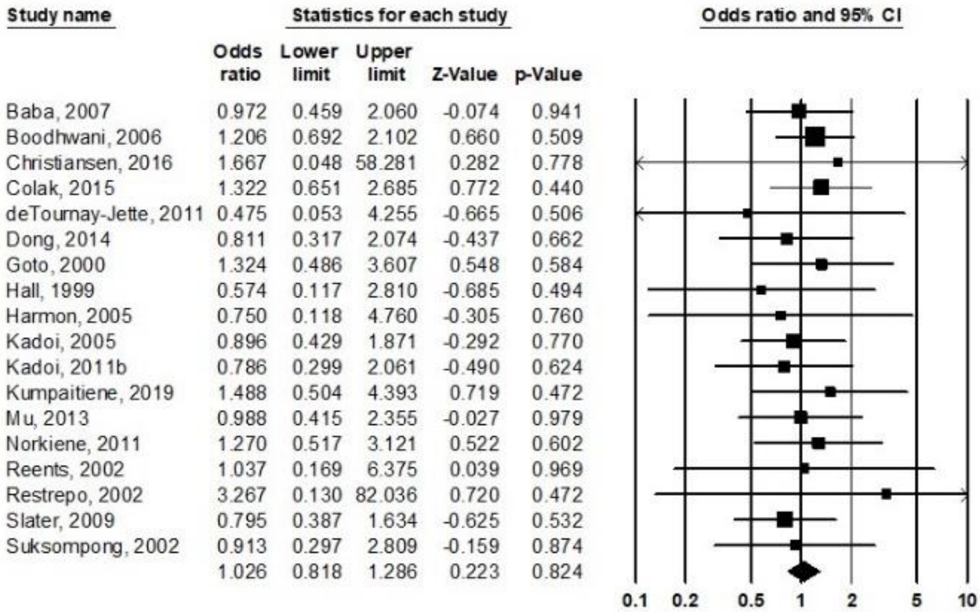

Study name	Odds ratio	Lower limit	Upper limit	Z-Value	p-Value
Baba, 2007	0.972	0.459	2.060	-0.074	0.941
Boodhwani, 2006	1.206	0.692	2.102	0.660	0.509
Christiansen, 2016	1.667	0.048	58.281	0.282	0.778
Colak, 2015	1.322	0.651	2.685	0.772	0.440
deTournay-Jette, 2011	0.475	0.053	4.255	-0.665	0.506
Dong, 2014	0.811	0.317	2.074	-0.437	0.662
Goto, 2000	1.324	0.486	3.607	0.548	0.584
Hall, 1999	0.574	0.117	2.810	-0.685	0.494
Harmon, 2005	0.750	0.118	4.760	-0.305	0.760
Kadoi, 2005	0.896	0.429	1.871	-0.292	0.770
Kadoi, 2011b	0.786	0.299	2.061	-0.490	0.624
Kumpaitiene, 2019	1.488	0.504	4.393	0.719	0.472
Mu, 2013	0.988	0.415	2.355	-0.027	0.979
Norkiene, 2011	1.270	0.517	3.121	0.522	0.602
Reents, 2002	1.037	0.169	6.375	0.039	0.969
Restrepo, 2002	3.267	0.130	82.036	0.720	0.472
Slater, 2009	0.795	0.387	1.634	-0.625	0.532
Suksompong, 2002	0.913	0.297	2.809	-0.159	0.874
	1.026	0.818	1.286	0.223	0.824

Kidney injury

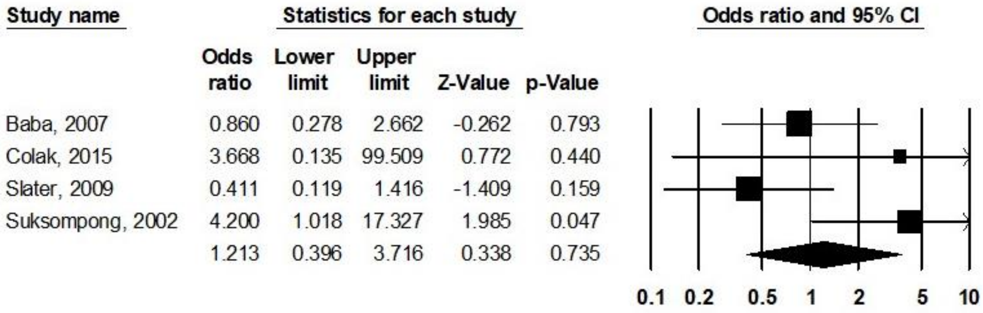

Study name	Odds ratio	Lower limit	Upper limit	Z-Value	p-Value
Baba, 2007	0.860	0.278	2.662	-0.262	0.793
Colak, 2015	3.668	0.135	99.509	0.772	0.440
Slater, 2009	0.411	0.119	1.416	-1.409	0.159
Suksompong, 2002	4.200	1.018	17.327	1.985	0.047
	1.213	0.396	3.716	0.338	0.735

Previous MI <90 days

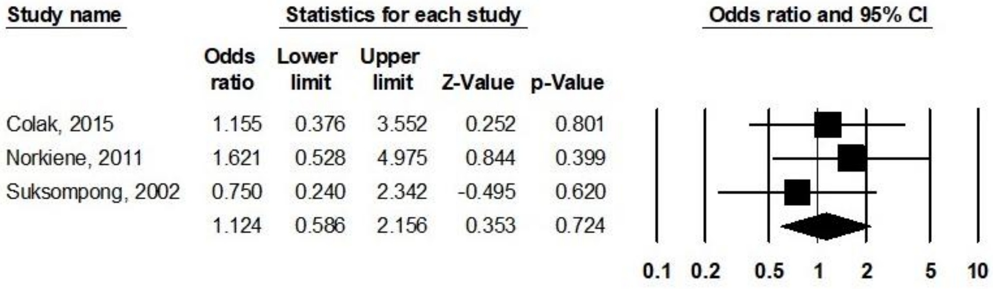

Study name	Odds ratio	Lower limit	Upper limit	Z-Value	p-Value
Colak, 2015	1.155	0.376	3.552	0.252	0.801
Norkiene, 2011	1.621	0.528	4.975	0.844	0.399
Suksompong, 2002	0.750	0.240	2.342	-0.495	0.620
	1.124	0.586	2.156	0.353	0.724

Previous MI history/ever

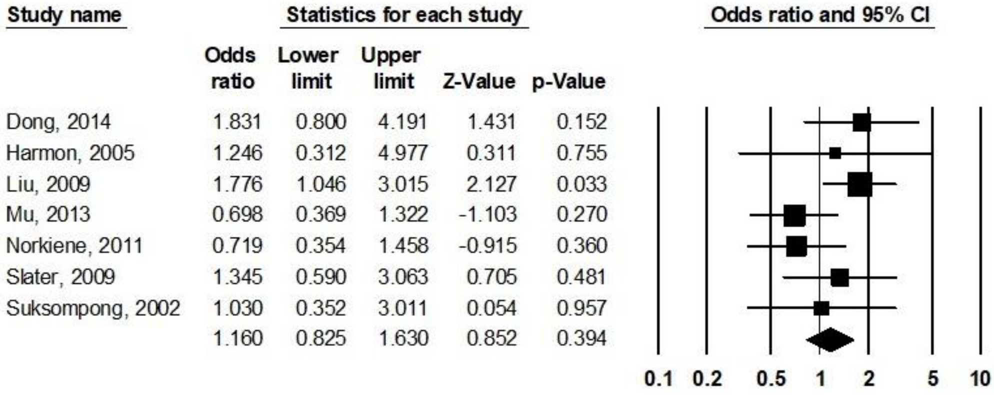

Study name	Odds ratio	Lower limit	Upper limit	Z-Value	p-Value
Dong, 2014	1.831	0.800	4.191	1.431	0.152
Harmon, 2005	1.246	0.312	4.977	0.311	0.755
Liu, 2009	1.776	1.046	3.015	2.127	0.033
Mu, 2013	0.698	0.369	1.322	-1.103	0.270
Norkiene, 2011	0.719	0.354	1.458	-0.915	0.360
Slater, 2009	1.345	0.590	3.063	0.705	0.481
Suksompong, 2002	1.030	0.352	3.011	0.054	0.957
	1.160	0.825	1.630	0.852	0.394

Previous stroke, TIA, CVA

Study name	Odds ratio	Lower limit	Upper limit	Z-Value	p-Value
Baba, 2007	4.799	1.899	12.129	3.315	0.001
Dong, 2014	2.814	0.795	9.959	1.604	0.109
Mu, 2013	1.445	0.576	3.623	0.785	0.433
Restrepo, 2002	4.500	0.190	106.823	0.931	0.352
Slater, 2009	1.652	0.553	4.938	0.899	0.369
	2.435	1.468	4.038	3.447	0.001

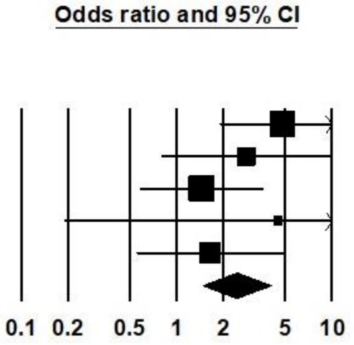

PVD

Study name	Odds ratio	Lower limit	Upper limit	Z-Value	p-Value
Baba, 2007	0.414	0.120	1.432	-1.393	0.164
Boodhwani, 2006	0.990	0.560	1.748	-0.036	0.971
Goto, 2000	4.353	1.185	15.987	2.216	0.027
Restrepo, 2002	0.486	0.018	12.929	-0.431	0.666
	1.087	0.417	2.831	0.170	0.865

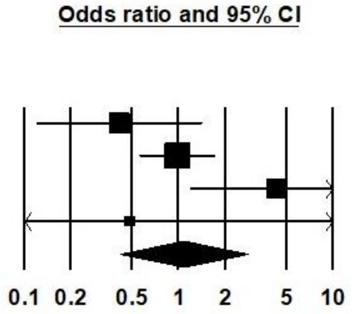

Smoking current/history

Study name	Odds ratio	Lower limit	Upper limit	Z-Value	p-Value
Baba, 2007	0.902	0.445	1.826	-0.287	0.774
Dong, 2014	1.075	0.475	2.431	0.174	0.862
Kadoi, 2001	0.100	0.005	1.924	-1.526	0.127
Kadoi, 2005	1.033	0.553	1.931	0.103	0.918
Liu, 2009	0.337	0.189	0.603	-3.668	0.000
Mu, 2013	1.382	0.740	2.581	1.016	0.310
Norkiene, 2011	1.693	0.755	3.798	1.278	0.201
Restrepo, 2002	15.000	0.597	376.696	1.647	0.100
Slater, 2009	1.729	0.997	2.997	1.950	0.051
	1.033	0.642	1.664	0.135	0.892

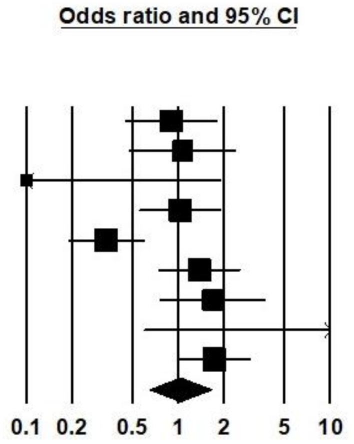

Pre-Operative (Continuous)

Age (years)

Study name	Difference in means	Standard error	Variance	Lower limit	Upper limit	Z-Value	p-Value
Baba, 2007	0.700	0.987	0.973	-1.234	2.634	0.710	0.478
Boodhwani, 2006	0.500	0.038	0.001	0.425	0.575	13.005	0.000
Christiansen, 2016	1.900	10.530	110.882	-18.739	22.539	0.180	0.857
Colak, 2015	7.300	1.091	1.191	5.161	9.439	6.689	0.000
deTournay-Jette, 2011	-3.010	1.539	2.368	-6.026	0.006	-1.956	0.050
Dong, 2014	6.000	1.526	2.330	3.008	8.992	3.931	0.000
Goto, 2000	1.500	1.168	1.364	-0.789	3.789	1.284	0.199
Hall, 1999	3.300	3.234	10.460	-3.039	9.639	1.020	0.308
Hamon, 2005	8.300	2.425	5.883	3.546	13.054	3.422	0.001
Kadoi, 2005	7.000	1.500	2.251	4.060	9.940	4.666	0.000
Kadoi, 2011b	-0.200	1.098	1.206	-2.352	1.952	-0.182	0.855
Kumpaitiene, 2019	4.000	2.176	4.736	-0.265	8.265	1.838	0.066
Liu, 2009	2.000	1.117	1.247	-0.188	4.188	1.791	0.073
Mu, 2013	2.000	1.413	1.996	-0.769	4.769	1.416	0.157
Newman, 1987	6.800	2.045	4.180	2.793	10.807	3.326	0.001
Norkiene, 2011	2.400	1.290	1.665	-0.129	4.929	1.860	0.063
Reents, 2002	1.000	2.465	6.075	-3.831	5.831	0.406	0.685
Restrepo, 2002	0.570	6.428	41.315	-12.028	13.168	0.089	0.929
Scott, 2002	5.900	0.392	0.153	5.132	6.668	15.059	0.000
Slater, 2009	-0.020	1.313	1.724	-2.593	2.553	-0.015	0.988
Stump, 1996	2.000	1.810	3.276	-1.548	5.548	1.105	0.269
Sylivris, 1998	-2.800	2.776	7.704	-8.240	2.640	-1.009	0.313
	2.688	0.760	0.577	1.198	4.177	3.537	0.000

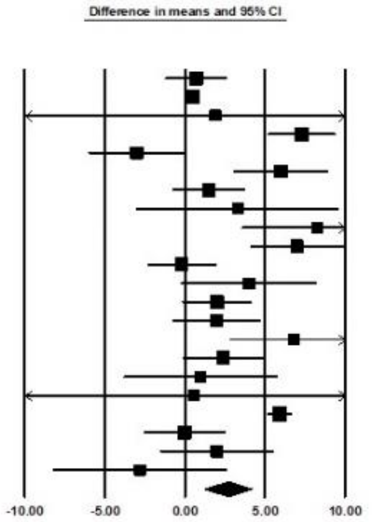

BMI

Study name	Difference in means	Standard error	Variance	Lower limit	Upper limit	Z-Value	p-Value
Boodhwani, 2006	-0.200	0.029	0.001	-0.257	-0.143	-6.936	0.000
deTournay-Jette, 2011	1.510	1.495	2.234	-1.420	4.440	1.010	0.312
Dong, 2014	0.500	0.521	0.271	-0.521	1.521	0.960	0.337
Mu, 2013	-0.200	0.470	0.220	-1.120	0.720	-0.426	0.670
Norkiene, 2011	-1.000	1.721	2.963	-4.374	2.374	-0.581	0.561
	-0.197	0.029	0.001	-0.254	-0.141	-6.874	0.000

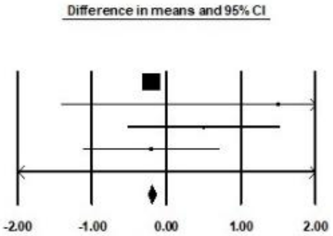

Cognition: All tests

Study name	Std diff in means	Standard error	Variance	Lower limit	Upper limit	Z-Value	p-Value
deTournay-Jette, 2011	0.823	0.344	0.119	0.148	1.498	2.390	0.017
Hall, 1999	0.237	0.346	0.120	-0.441	0.916	0.685	0.493
Kumpaitiene, 2019	-0.294	0.271	0.073	-0.824	0.236	-1.086	0.277
	0.230	0.334	0.112	-0.425	0.884	0.687	0.492

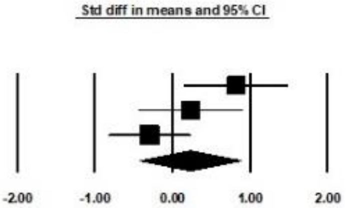

Cognition: MMSE only

Study name	Difference in means	Standard error	Variance	Lower limit	Upper limit	Z-Value	p-Value
deTournay-Jette, 2011	1.110	0.453	0.205	0.223	1.997	2.452	0.014
Kumpaitiene, 2019	-0.600	0.550	0.302	-1.677	0.477	-1.092	0.275
	0.283	0.855	0.730	-1.391	1.958	0.332	0.740

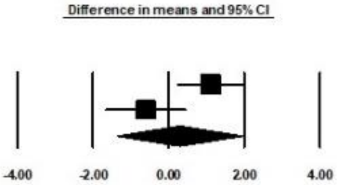

Depression: All tests

Study name	Std diff in means	Standard error	Variance	Lower limit	Upper limit	Z-Value	p-Value
Harmon, 2004	-0.310	0.340	0.116	-0.976	0.357	-0.910	0.363
Harmon, 2005	-0.039	0.342	0.117	-0.709	0.632	-0.113	0.910
Kadoi, 2011b	2.024	0.259	0.067	1.516	2.532	7.807	0.000
Silbert, 2006	0.274	0.151	0.023	-0.023	0.571	1.807	0.071
	0.501	0.499	0.249	-0.478	1.479	1.003	0.316

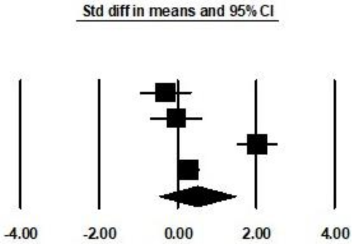

Education (years)

Study name	Difference in means	Standard error	Variance	Lower limit	Upper limit	Z-Value	p-Value
Colak, 2015	-1.200	0.492	0.242	-2.164	-0.236	-2.440	0.015
deTournay-Jette, 2011	1.070	1.436	2.063	-1.745	3.885	0.745	0.456
Hall, 1999	-1.600	0.913	0.834	-3.390	0.190	-1.752	0.080
Harmon, 2005	-1.300	1.217	1.480	-3.684	1.084	-1.069	0.285
Kumpaitiene, 2019	0.500	1.327	1.761	-2.101	3.101	0.377	0.706
Mu, 2013	0.600	0.534	0.285	-0.446	1.646	1.125	0.261
	-0.435	0.493	0.243	-1.400	0.531	-0.883	0.377

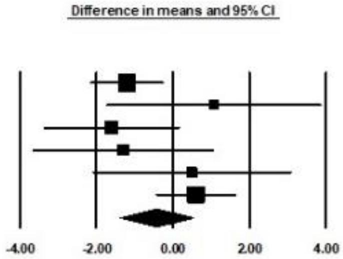

EuroSCORE

Study name	Difference in means	Standard error	Variance	Lower limit	Upper limit	Z-Value	p-Value
Colak, 2015	1.000	0.245	0.060	0.519	1.481	4.075	0.000
Dong, 2014	0.600	0.214	0.046	0.181	1.019	2.806	0.005
Mu, 2013	0.459	0.311	0.097	-0.151	1.068	1.475	0.140
Norkiene, 2011	1.000	0.382	0.146	0.252	1.748	2.621	0.009
	0.742	0.134	0.018	0.480	1.005	5.540	0.000

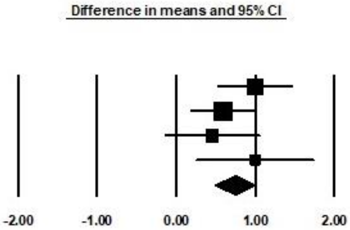

LVEF %

Study name	Difference in means	Standard error	Variance	Lower limit	Upper limit	Z-Value	p-Value
Harmon, 2005	-9.000	2.336	5.459	-13.579	-4.421	-3.852	0.000
Kadoi, 2011b	-2.000	1.585	2.511	-5.106	1.106	-1.262	0.207
Kadoi, 2005	-3.000	1.775	3.150	-6.479	0.479	-1.690	0.091
Mu, 2013	2.900	1.682	2.829	-0.396	6.196	1.724	0.085
Norkiene, 2011	-2.000	1.462	2.137	-4.865	0.865	-1.368	0.171
Reents, 2002	-10.000	3.109	9.665	-16.093	-3.907	-3.217	0.001
Kumpaitiene, 2019	1.000	2.193	4.810	-3.298	5.298	0.456	0.648
Colak, 2015	0.100	1.458	2.125	-2.757	2.957	0.069	0.945
Slater, 2009	0.100	1.551	2.406	-2.940	3.140	0.064	0.949
	-1.966	1.147	1.315	-4.213	0.281	-1.715	0.086

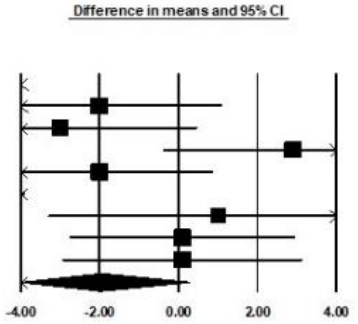

Intra-Operative (Continuous)

ACC time (mins)

Study name	Difference in means	Standard error	Variance	Lower limit	Upper limit	Z-Value	p-Value
Dong, 2014	-4.000	3.672	13.485	-11.197	3.197	-1.089	0.276
Hall, 1999	-18.000	5.889	34.679	-29.542	-6.458	-3.057	0.002
Jonsson, 2004	-6.500	7.258	52.681	-20.726	7.726	-0.896	0.370
Norkiene, 2010	4.000	2.682	7.195	-1.257	9.257	1.491	0.136
Restrepo, 2002	2.000	3.657	13.374	-5.168	9.168	0.547	0.584
Robson, 2000	-2.000	6.786	46.052	-15.301	11.301	-0.295	0.768
Stump, 1996	2.700	1.814	3.290	-0.855	6.255	1.489	0.137
	-1.337	2.333	5.441	-5.909	3.234	-0.573	0.566

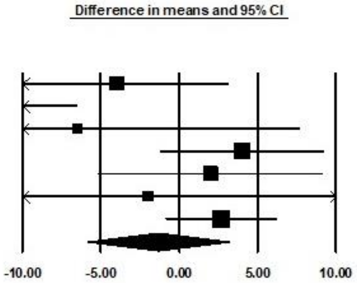

CPB time (mins)

Study name	Difference in means	Standard error	Variance	Lower limit	Upper limit	Z-Value	p-Value
Boodhwani, 2006	-0.100	0.156	0.024	-0.406	0.206	-0.641	0.521
Colak, 2015	7.000	4.582	20.997	-1.981	15.981	1.528	0.127
Goto, 2000	-6.100	5.015	25.155	-15.930	3.730	-1.216	0.224
Harmon, 2005	9.400	7.809	60.976	-5.905	24.705	1.204	0.229
Kadoi, 2005	7.000	3.410	11.628	0.317	13.683	2.053	0.040
Kadoi, 2011b	-3.200	3.869	14.971	-10.784	4.384	-0.827	0.408
Kumpaitiene, 2019	-4.000	5.485	30.087	-14.751	6.751	-0.729	0.466
Newman, 1987	18.600	7.989	63.830	2.941	34.259	2.328	0.020
Norkiene, 2011	6.000	4.468	19.965	-2.758	14.758	1.343	0.179
Reents, 2002	-6.000	11.208	125.618	-27.967	15.967	-0.535	0.592
Slater, 2009	3.900	2.699	7.284	-1.390	9.190	1.445	0.148
Smith, 1986	13.000	8.217	67.527	-3.106	29.106	1.582	0.114
Sylivris, 1998	30.000	11.846	140.317	6.783	53.217	2.533	0.011
	3.390	1.778	3.162	-0.095	6.876	1.907	0.057

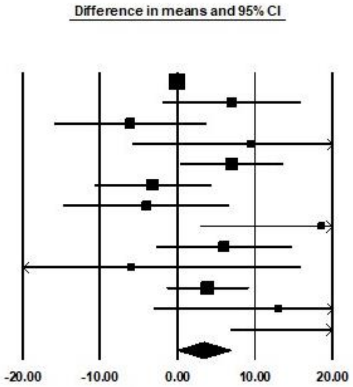

Duration of surgery (mins)

Study name	Difference in means	Standard error	Variance	Lower limit	Upper limit	Z-Value	p-Value
Baba, 2007	9.000	12.219	149.307	-14.949	32.949	0.737	0.461
deTournay-Jette, 2011	10.000	13.167	173.366	-15.807	35.807	0.759	0.448
Dong, 2014	30.000	11.954	142.898	6.571	53.429	2.510	0.012
Mu, 2013	16.200	10.029	100.584	-3.457	35.857	1.615	0.106
Norkiene, 2011	18.300	7.616	58.003	3.373	33.227	2.403	0.016
Reents, 2002	-12.000	13.111	171.891	-37.697	13.697	-0.915	0.360
	13.525	5.027	25.266	3.673	23.377	2.691	0.007

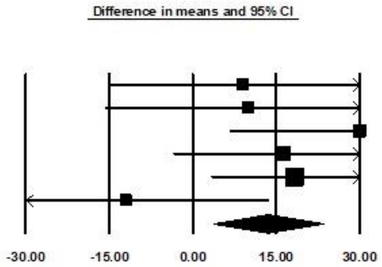

Intubation time (hours)

Study name	Difference in means	Standard error	Variance	Lower limit	Upper limit	Z-Value	p-Value
Boodhwani, 2006	2.300	0.064	0.004	2.174	2.426	35.907	0.000
Colak, 2015	1.800	0.581	0.337	0.661	2.939	3.099	0.002
Liu, 2009	1.800	0.784	0.614	0.264	3.336	2.297	0.022
Mu, 2013	0.224	0.854	0.729	-1.449	1.898	0.263	0.793
Norkiene, 2011	2.082	1.511	2.282	-0.879	5.043	1.378	0.168
Reents, 2002	2.200	1.736	3.015	-1.203	5.603	1.267	0.205
	1.946	0.292	0.085	1.374	2.517	6.672	0.000

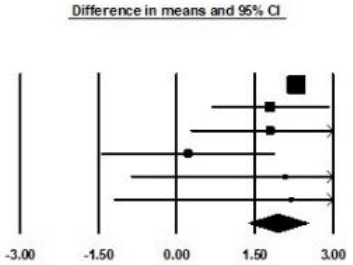

Number of grafts

Study name	Difference in means	Standard error	Variance	Lower limit	Upper limit	Z-Value	p-Value
Boodhwani, 2006	0.000	0.004	0.000	-0.008	0.008	0.000	1.000
Colak, 2015	0.100	0.091	0.008	-0.078	0.278	1.100	0.271
Dong, 2014	0.200	0.226	0.051	-0.243	0.643	0.884	0.377
Harmon, 2005	0.200	0.433	0.188	-0.650	1.050	0.461	0.645
Mu, 2013	0.200	0.165	0.027	-0.124	0.524	1.212	0.226
Norkiene, 2011	0.300	0.196	0.038	-0.084	0.684	1.533	0.125
Reents, 2002	-0.200	0.289	0.083	-0.766	0.366	-0.693	0.489
	0.025	0.030	0.001	-0.034	0.085	0.842	0.400

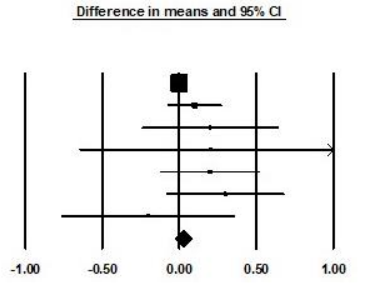

Total Microemboli

Study name	Std diff in means	Standard error	Variance	Lower limit	Upper limit	Z-Value	p-Value
Liu, 2009	0.236	0.134	0.018	-0.026	0.499	1.763	0.078
Rodriguez, 2010	-0.055	0.107	0.011	-0.264	0.154	-0.512	0.608
Royse, 2000	0.681	0.550	0.302	-0.397	1.758	1.238	0.216
Stump, 1996	0.295	0.170	0.029	-0.038	0.627	1.736	0.082
	0.157	0.113	0.013	-0.066	0.379	1.381	0.167

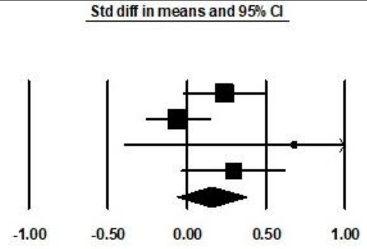

Post-Operative (Categorical)

Arrhythmia, incl. AF

Study name	Odds ratio	Lower limit	Upper limit	Z-Value	p-Value
Boodhwani, 2006	1.398	0.922	2.122	1.576	0.115
Colak, 2015	1.176	0.591	2.342	0.462	0.644
Mu, 2013	1.531	0.210	11.147	0.421	0.674
Norkiene, 2011	3.265	0.966	11.035	1.905	0.057
Restrepo, 2002	2.000	0.134	29.808	0.503	0.615
Suksompong, 2002	0.870	0.220	3.435	-0.199	0.842
	1.402	1.013	1.941	2.038	0.042

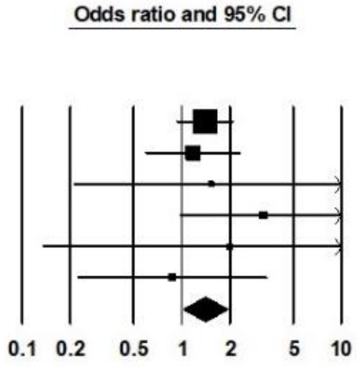

Delirium

Study name	Odds ratio	Lower limit	Upper limit	Z-Value	p-Value
Colak, 2015	4.171	1.458	11.935	2.662	0.008
Norkiene, 2011	9.019	1.076	75.621	2.027	0.043
Reents, 2002	39.000	2.021	752.636	2.426	0.015
	6.147	2.322	16.269	3.657	0.000

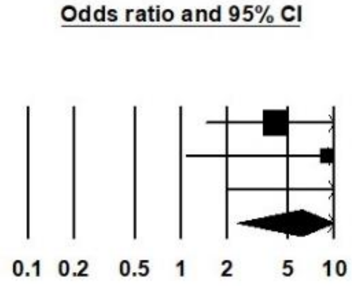

Post-Operative (Continuous)

LOS in ICU (days)

Study name	Difference in means	Standard error	Variance	Lower limit	Upper limit	Z-Value	p-Value
Boodhwani, 2006	0.290	0.008	0.000	0.274	0.306	36.220	0.000
Colak, 2015	0.300	0.146	0.021	0.015	0.585	2.060	0.039
deTournay-Jette, 2011	-0.710	0.479	0.230	-1.649	0.229	-1.481	0.138
Harmon, 2005	0.075	0.188	0.036	-0.294	0.444	0.398	0.691
Mu, 2013	-0.242	0.240	0.058	-0.712	0.228	-1.010	0.312
Norkiene, 2011	0.600	0.216	0.047	0.176	1.024	2.774	0.006
Sahan, 2018	1.200	0.240	0.057	0.730	1.670	5.007	0.000
	0.294	0.132	0.017	0.036	0.552	2.235	0.025

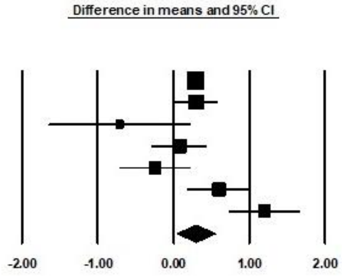

**Figure S4. Funnel plots for statistically significant analyses in regard to acute cognitive decline post-CABG, and results of publication bias/small-study effect investigation when more than 10 studies were available.**		**Egger's Test**		**Trim and Fill**		
		**Intercept**	**p value (1-tailed)**	**No. imputed studies**	**OR/MD† /SMD‡**	**95%CI**
**Preoperative (Categorical)**						
Depression	Only 2 studies, could not produce funnel plot			-	-	-
Diabetes	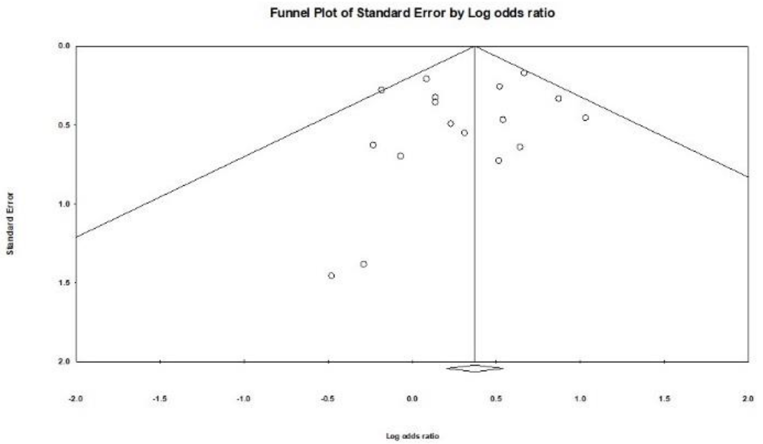	-0.39	.232	-	-	-

Hypertension	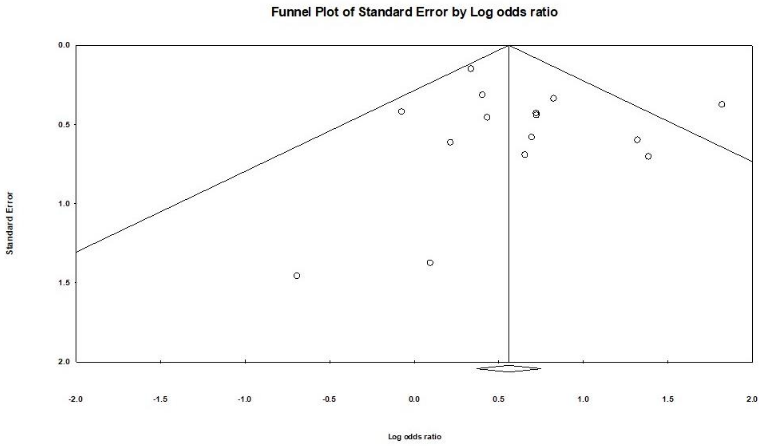	0.55	.197	-	-	-
Previous stroke, TIA, CVA	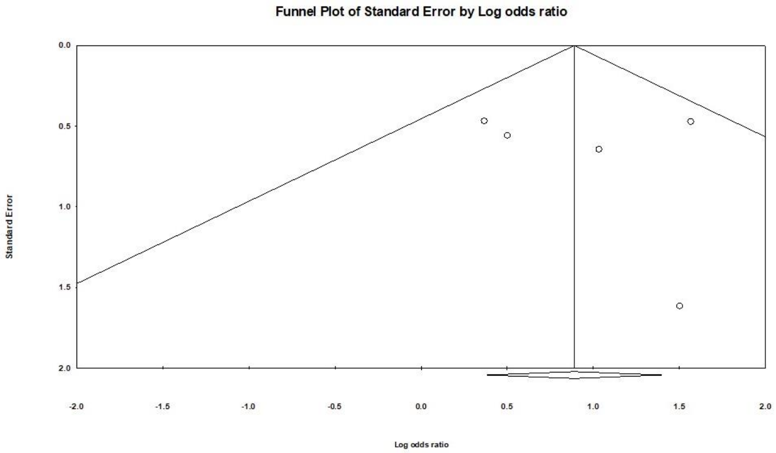	-	-	-	-	-
**Preoperative (Continuous)**						

<table>
<tr><td>Age</td><td>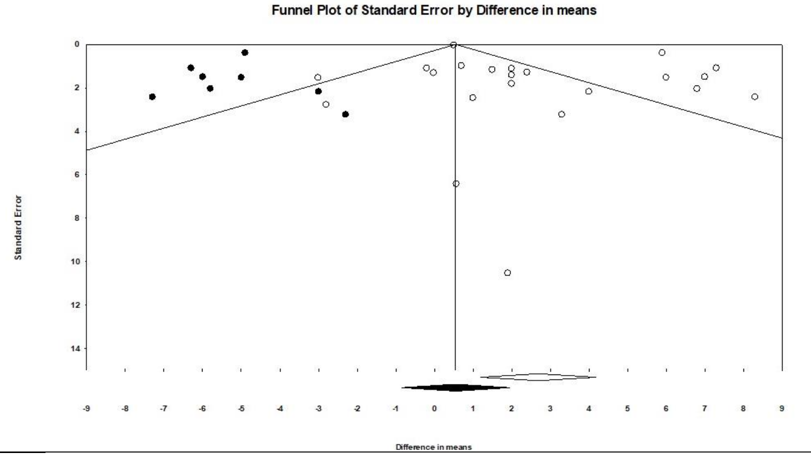
</td><td>1.80</td><td>.015</td><td>8</td><td>0.55†</td><td>-0.86-1.95</td></tr>
<tr><td>BMI</td><td>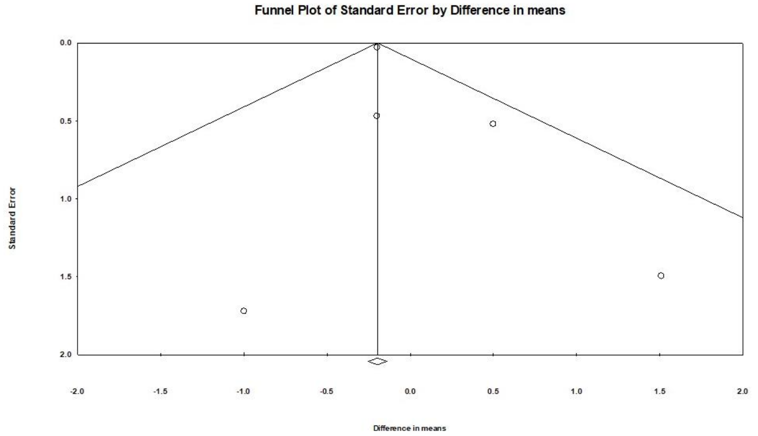
</td><td>-</td><td>-</td><td>-</td><td>-</td><td>-</td></tr>
</table>

EuroSCORE	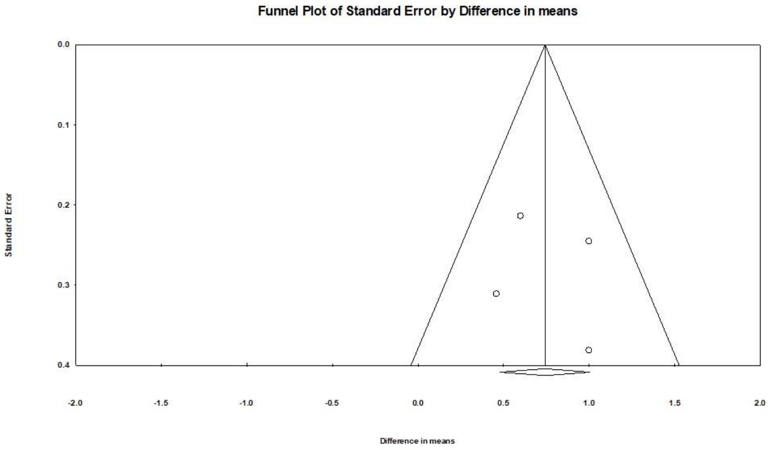	-	-	-	-	-
**Intra-Operative (Continuous)**						
Duration of surgery (mins)	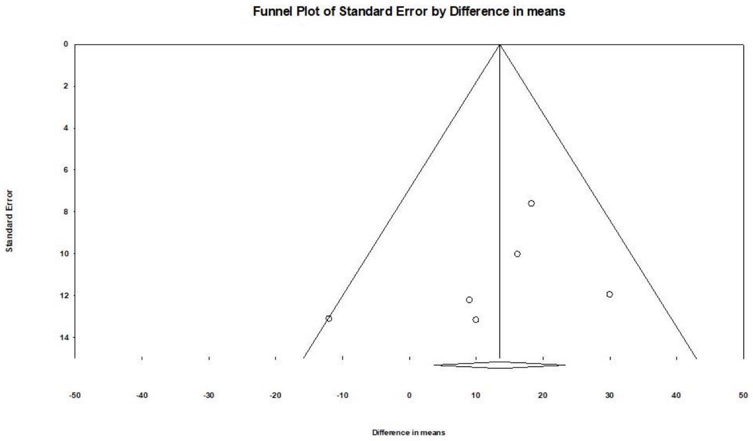	-	-	-	-	-

Intubation time (hours)	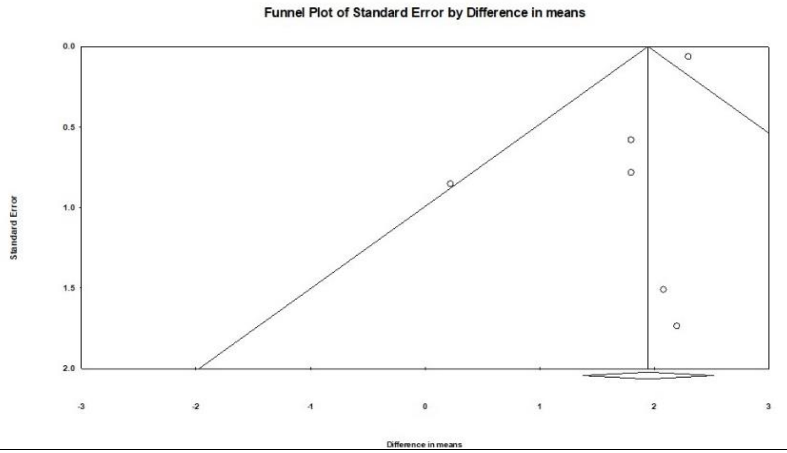	-	-	-	-	-
**Post-Operative (Categorical)**						
Arrhythmia, incl. AF	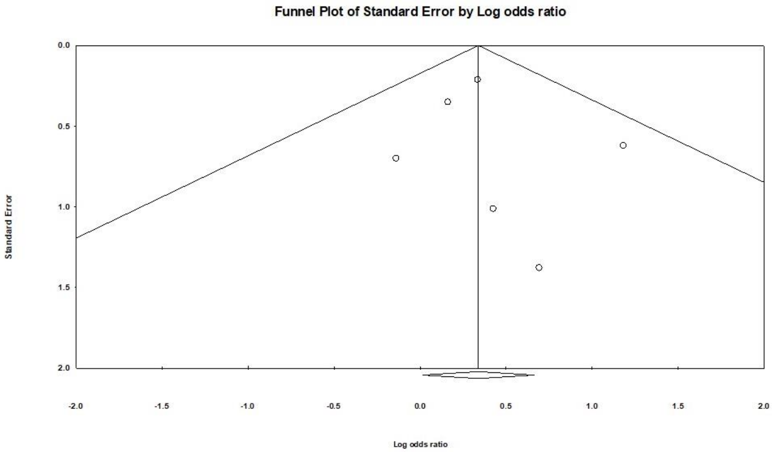	-	-	-	-	-

Delirium	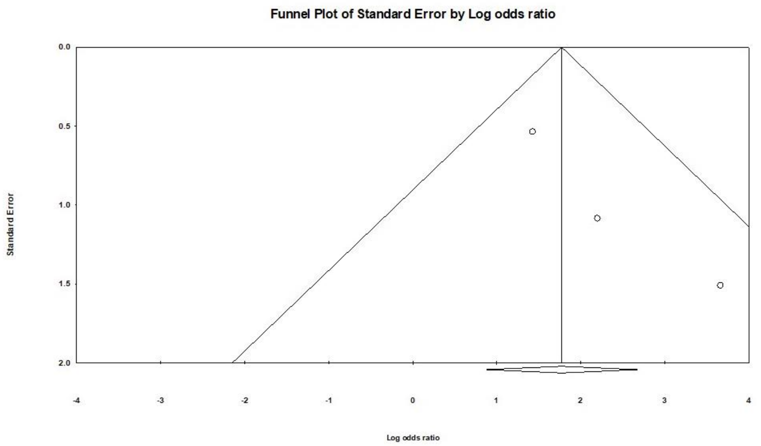	-	-	-	-	-
**Post-Operative (Continuous)**						
LOS in ICU (days)	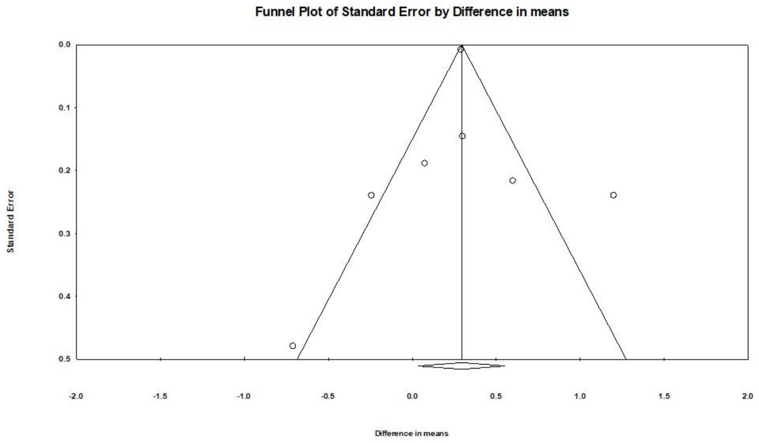	-	-	-	-	-

Figure S5. Forest plots for mid-term cognitive decline post-CABG analyses.

Variable	Forest Plot
**Pre-Operative (Categorical)**	
Depression	see below
Diabetes	see below
Sex (male)	see below

Depression

Study name	Comparison	Odds ratio	Lower limit	Upper limit	Z-Value	p-Value
Kadoi, 2011b	Blank	3.365	1.218	9.298	2.340	0.019
Smith, 2000	Blank	2.000	0.825	4.851	1.533	0.125
		2.504	1.284	4.883	2.693	0.007

Statistics for each study

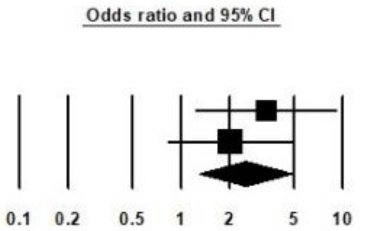

Diabetes

Study name	Comparison	Odds ratio	Lower limit	Upper limit	Z-Value	p-Value
deTournay-Jette, 2011	Blank	0.719	0.237	2.179	-0.584	0.560
Dieleman, 2009	Blank	1.403	0.384	5.131	0.512	0.609
Dijk, 2004	Blank	1.096	0.463	2.595	0.208	0.835
Kadoi, 2002	Blank	3.500	0.645	18.980	1.452	0.146
Kadoi, 2005	Blank	3.163	1.562	6.405	3.198	0.001
Kadoi, 2007	Blank	6.894	2.511	18.924	3.747	0.000
Liu, 2009	Blank	0.375	0.104	1.347	-1.503	0.133
Mathew, 2006	Blank	1.050	0.366	3.014	0.091	0.927
Mathew, 2007	Blank	1.138	0.762	1.701	0.632	0.527
Swaminathan, 2002	Blank	1.765	0.975	3.196	1.878	0.060
		1.536	0.984	2.396	1.890	0.059

Statistics for each study

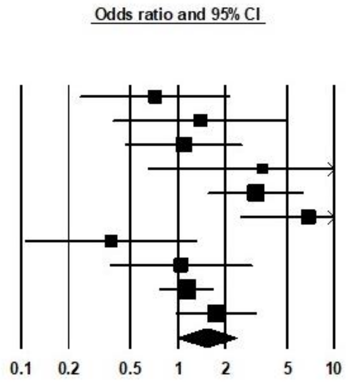

Sex (male)

Study name	Comparison	Odds ratio	Lower limit	Upper limit	Z-Value	p-Value
deTournay-Jette, 2011	Blank	1.556	0.359	6.739	0.591	0.555
Dieleman, 2009	Blank	0.679	0.256	1.802	-0.777	0.437
Dijk, 2004	Blank	1.000	0.531	1.884	-0.000	1.000
Kadoi, 2007	Blank	0.806	0.324	2.007	-0.464	0.643
Kadoi, 2011b	Blank	1.046	0.333	3.283	0.077	0.938
Kadoi, 2005	Blank	0.676	0.318	1.436	-1.018	0.309
Khatri, 1999	Blank	0.670	0.331	1.354	-1.116	0.264
Mathew, 2007	Blank	1.204	0.805	1.801	0.906	0.365
Mathew, 2006	Blank	1.000	0.363	2.757	0.000	1.000
Smith, 2000	Blank	0.862	0.420	1.769	-0.405	0.686
Swaminathan, 2002	Blank	1.490	0.868	2.558	1.447	0.148
Slater, 2009	Blank	0.860	0.359	2.062	-0.338	0.735
		1.005	0.818	1.234	0.043	0.965

Statistics for each study

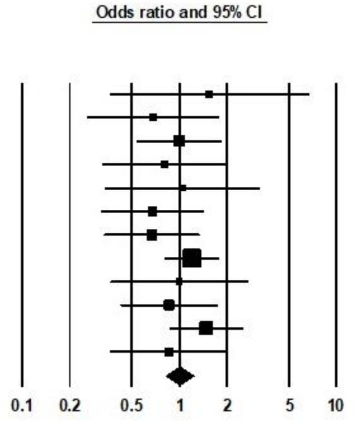

Hypertension

Study name	Odds ratio	Lower limit	Upper limit	Z-Value	p-Value
deTournay-Jette, 2011	1.016	0.341	3.026	0.028	0.978
Dieleman, 2009	0.901	0.342	2.375	-0.211	0.833
Kadoi, 2001	0.200	0.100	0.400	-4.551	0.000
Kadoi, 2002	0.500	0.129	1.936	-1.003	0.316
Kadoi, 2003	0.300	0.087	1.039	-1.899	0.058
Kadoi, 2005	0.925	0.438	1.954	-0.205	0.837
Kadoi, 2007	1.195	0.478	2.983	0.381	0.703
Kadoi, 2011b	1.222	0.456	3.273	0.399	0.690
Mathew, 2006	2.092	0.626	6.991	1.200	0.230
Mathew, 2007	1.000	0.689	1.450	-0.000	1.000
Smith, 2000	1.000	0.581	1.722	0.000	1.000
Swaminathan, 2002	2.083	1.282	3.386	2.963	0.003
	0.887	0.594	1.324	-0.586	0.558

Odds ratio and 95% CI

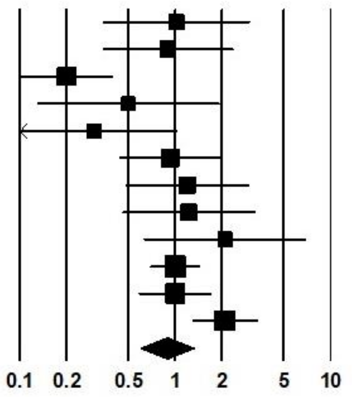

Previous MI history/ever

Study name	Odds ratio	Lower limit	Upper limit	Z-Value	p-Value
Mathew, 2006	0.643	0.246	1.676	-0.904	0.366
Mathew, 2007	0.721	0.471	1.103	-1.509	0.131
Smith, 2000	1.310	0.770	2.229	0.995	0.320
	0.883	0.568	1.373	-0.553	0.580

Odds ratio and 95% CI

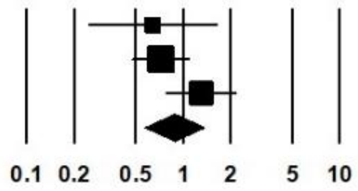

Previous stroke, TIA, CVA

Study name	Comparison	Odds ratio	Lower limit	Upper limit	Z-Value	p-Value
Dijk, 2004	Blank	0.742	0.145	3.800	-0.358	0.721
Mathew, 2007	Blank	0.922	0.180	4.737	-0.097	0.923
		0.827	0.260	2.628	-0.322	0.748

Odds ratio and 95% CI

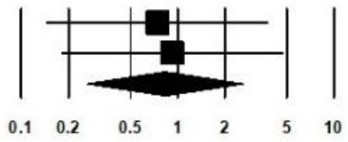

PVD

Study name	Comparison	Odds ratio	Lower limit	Upper limit	Z-Value	p-Value
Dijk, 2004	Blank	1.000	0.347	2.885	0.000	1.000
Mathew, 2007	Blank	0.628	0.337	1.173	-1.458	0.145
		0.708	0.414	1.213	-1.256	0.209

Odds ratio and 95% CI

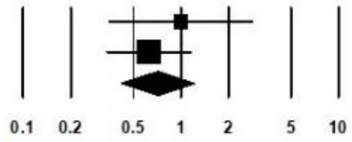

Smoking (current)

Study name	Odds ratio	Lower limit	Upper limit	Z-Value	p-Value
Djaiani, 2003	0.982	0.657	1.467	-0.089	0.929
Kadoi, 2001	0.900	0.222	3.656	-0.147	0.883
Kadoi, 2002	0.700	0.211	2.322	-0.583	0.560
Kadoi, 2003	0.700	0.130	3.770	-0.415	0.678
Kadoi, 2005	1.275	0.665	2.445	0.733	0.464
	1.003	0.733	1.374	0.021	0.983

Odds ratio and 95% CI

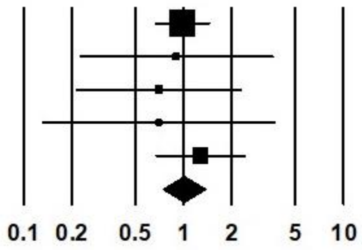

Pre-Operative (Continuous)

Age (years)

Study name	Difference in means	Standard error	Variance	Lower limit	Upper limit	Z-Value	p-Value
Braekken, 1998	7.605	6.935	48.090	-5.987	21.197	1.097	0.273
deTournay-Jette, 2011	-1.480	1.236	1.529	-3.903	0.943	-1.197	0.231
Dieleman, 2009	0.200	2.171	4.713	-4.055	4.455	0.092	0.927
Dijk, 2004	0.000	1.320	1.742	-2.587	2.587	0.000	1.000
Kadoi, 2005	1.000	1.775	3.149	-2.478	4.478	0.564	0.573
Kadoi, 2007	6.000	2.503	6.267	1.093	10.907	2.397	0.017
Kadoi, 2011b	1.900	1.501	2.253	-1.042	4.842	1.266	0.206
Liu, 2009	4.300	1.935	3.746	0.507	8.093	2.222	0.026
Mathew, 2006	3.000	2.507	6.283	-1.913	7.913	1.197	0.231
Mathew, 2007	0.400	0.956	0.914	-1.474	2.274	0.418	0.676
Robson, 2000	4.000	2.847	8.103	-1.579	9.579	1.405	0.160
Swaminathan, 2002	0.000	1.271	1.615	-2.491	2.491	0.000	1.000
	1.093	0.589	0.346	-0.060	2.247	1.856	0.063

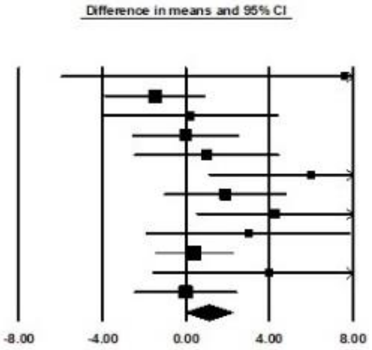

Cognition: All tests

Study name	Std diff in means	Standard error	Variance	Lower limit	Upper limit	Z-Value	p-Value
deTournay-Jette, 2011	-0.256	0.267	0.071	-0.779	0.266	-0.961	0.336
Mathew, 2007	0.396	0.093	0.009	0.214	0.578	4.259	0.000
Swaminathan, 2002	0.483	0.123	0.015	0.241	0.724	3.912	0.000
	0.303	0.148	0.022	0.013	0.593	2.048	0.041

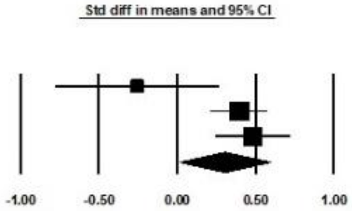

Cognition: CI only

Study name	Difference in means	Standard error	Variance	Lower limit	Upper limit	Z-Value	p-Value
Mathew, 2007	0.189	0.044	0.002	0.103	0.275	4.297	0.000
Swaminathan, 2002	0.880	0.222	0.049	0.445	1.315	3.966	0.000
	0.500	0.344	0.118	-0.174	1.174	1.455	0.146

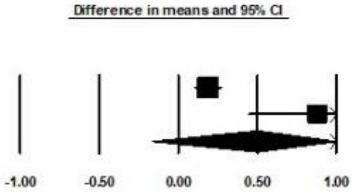

Depression: All tests

Study name	Std diff in means	Standard error	Variance	Lower limit	Upper limit	Z-Value	p-Value
Harmon, 2004	0.000	0.333	0.111	-0.653	0.653	0.000	1.000
Kadoi, 2011b	2.532	0.313	0.098	1.919	3.144	8.098	0.000
Silbert, 2006	0.060	0.172	0.029	-0.277	0.396	0.348	0.728
	0.856	0.781	0.610	-0.675	2.386	1.096	0.273

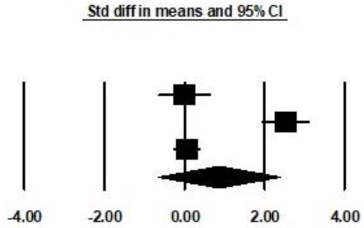

Education (years)

Study name	Difference in means	Standard error	Variance	Lower limit	Upper limit	Z-Value	p-Value
Braekken, 1998	-5.340	3.354	11.251	-11.914	1.234	-1.592	0.111
deTournay-Jette, 2011	0.650	1.114	1.241	-1.534	2.834	0.583	0.560
Mathew, 2006	-1.000	0.661	0.437	-2.295	0.295	-1.513	0.130
Mathew, 2007	0.400	0.292	0.085	-0.172	0.972	1.371	0.170
Swaminathan, 2002	0.500	0.377	0.142	-0.239	1.239	1.325	0.185
	0.130	0.356	0.127	-0.567	0.827	0.365	0.715

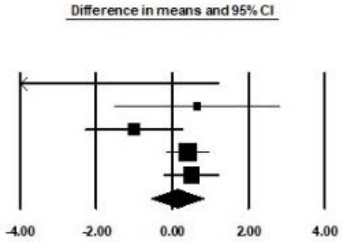

LVEF %

Study name	Difference in means	Standard error	Variance	Lower limit	Upper limit	Z-Value	p-Value
Kadoi, 2005	-2.000	1.589	2.524	-5.114	1.114	-1.259	0.208
Kadoi, 2007	1.000	2.431	5.910	-3.765	5.765	0.411	0.681
Kadoi, 2011b	0.900	1.518	2.304	-2.075	3.875	0.593	0.553
Mathew, 2006	2.000	2.608	6.802	-3.112	7.112	0.767	0.443
Mathew, 2007	1.100	1.085	1.177	-1.026	3.226	1.014	0.311
Braekken, 1998	7.043	13.513	182.612	-19.443	33.529	0.521	0.602
Swaminathan, 2002	1.000	1.444	2.085	-1.830	3.830	0.693	0.489
	0.610	0.635	0.403	-0.634	1.855	0.961	0.336

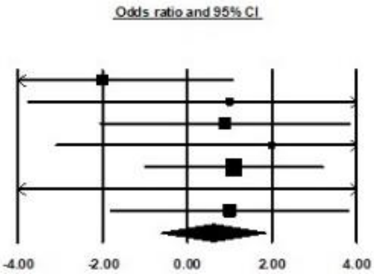

Intra-Operative (Continuous)	

ACC time (mins)

Study name	Difference in means	Standard error	Variance	Lower limit	Upper limit	Z-Value	p-Value
	Statistics for each study						
Braekken, 1998	8.086	10.815	116.956	-13.111	29.282	0.748	0.455
Mathew, 2006	-4.300	5.114	26.155	-14.324	5.724	-0.841	0.400
Mathew, 2007	-1.900	2.057	4.229	-5.931	2.131	-0.924	0.356
Swaminathan, 2002	0.100	2.790	7.784	-5.368	5.568	0.036	0.971
	-1.291	1.559	2.429	-4.346	1.763	-0.829	0.407

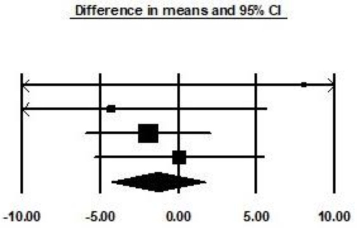

CPB time (mins)

Study name	Difference in means	Standard error	Variance	Lower limit	Upper limit	Z-Value	p-Value
	Statistics for each study						
Braekken, 1998	5.159	17.314	299.780	-28.776	39.094	0.298	0.766
Kadoi, 2005	8.000	3.639	13.242	0.868	15.132	2.198	0.028
Kadoi, 2007	3.000	4.684	21.942	-6.181	12.181	0.640	0.522
Kadoi, 2011b	9.900	6.036	36.428	-1.929	21.729	1.640	0.101
Mathew, 2006	-5.000	7.473	55.850	-19.647	9.647	-0.669	0.503
Mathew, 2007	-2.700	2.977	8.862	-8.535	3.135	-0.907	0.364
Swaminathan, 2002	-3.000	6.867	47.151	-16.458	10.458	-0.437	0.662
	2.149	2.343	5.490	-2.444	6.741	0.917	0.359

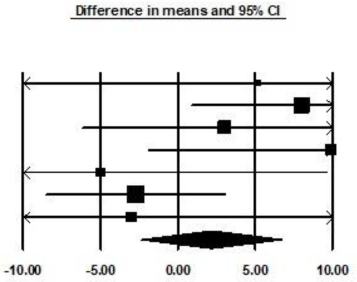

Number of grafts

Study name	Difference in means	Standard error	Variance	Lower limit	Upper limit	Z-Value	p-Value
	Statistics for each study						
Dieleman, 2009	0.020	0.244	0.059	-0.458	0.497	0.081	0.935
Mathew, 2006	-0.200	0.164	0.027	-0.521	0.121	-1.222	0.222
Mathew, 2007	0.000	0.083	0.007	-0.163	0.163	0.000	1.000
Swaminathan, 2002	-0.100	0.110	0.012	-0.315	0.115	-0.913	0.361
	-0.055	0.059	0.004	-0.171	0.062	-0.920	0.358

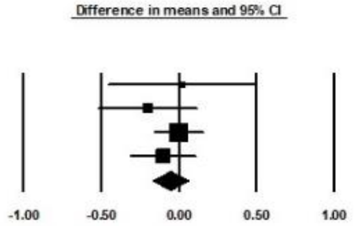

Total Microemboli

Study name	Std diff in means	Standard error	Variance	Lower limit	Upper limit	Z-Value	p-Value
	Statistics for each study						
Braekken, 1998	-0.770	0.778	0.605	-2.294	0.753	-0.991	0.322
Liu, 2009	-0.076	0.244	0.059	-0.553	0.402	-0.310	0.756
Rodriguez, 2010	-0.310	0.226	0.051	-0.752	0.133	-1.372	0.170
Royse, 2000	-1.491	0.535	0.287	-2.541	-0.442	-2.786	0.005
	-0.459	0.258	0.067	-0.965	0.048	-1.775	0.076

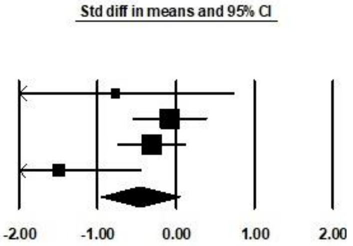

Post-Operative (Continuous)	

LOS in ICU (days)

Study name	Difference in means	Standard error	Variance	Lower limit	Upper limit	Z-Value	p-Value
	Statistics for each study						
Baba, 2007	0.880	0.276	0.076	0.338	1.422	3.185	0.001
Dieleman, 2009	-0.470	0.371	0.137	-1.197	0.257	-1.268	0.205
	0.228	0.675	0.455	-1.095	1.550	0.337	0.736

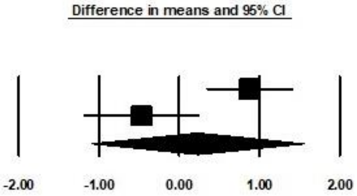

**Figure S6. Funnel plots for statistically significant analyses in regard to mid-term cognitive decline post-CABG, and results of publication bias/small-study effect investigation when more than 10 studies were available.**		**Egger's Test**		**Trim and Fill**		
		**Intercept**	**p value (1-tailed)**	**No. imputed studies**	**OR/MD† /SMD‡**	**95%CI**
**Preoperative (Categorical)**						
Depression	Only 2 studies, could not produce funnel plot	-	-	-	-	-
**Preoperative (Continuous)**						
Cognition: All tests	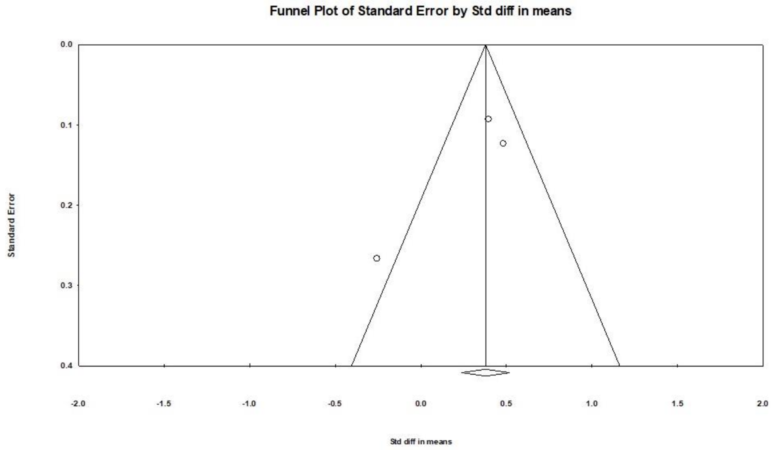	-	-	-	-	-

Figure S7. **Forest plots for long-term cognitive decline post-CABG analyses.**

Variable	Forest Plot
**Pre-Operative (Categorical)**	

Cognitive Impairment

Study name	Odds ratio	Lower limit	Upper limit	Z-Value	p-Value
Silbert, 2008	0.824	0.385	1.763	-0.499	0.618
Trubnikova, 2014	1.339	0.563	3.189	0.660	0.509
	1.018	0.574	1.803	0.060	0.952

Odds ratio and 95% CI

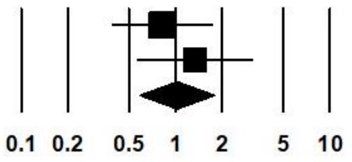

Diabetes

Study name	Odds ratio	Lower limit	Upper limit	Z-Value	p-Value
Dieleman, 2009	1.064	0.346	3.277	0.109	0.913
Lachmann, 2018	1.623	0.788	3.345	1.313	0.189
	1.435	0.781	2.636	1.164	0.245

Odds ratio and 95% CI

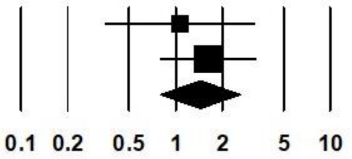

Sex (male)

Study name	Odds ratio	Lower limit	Upper limit	Z-Value	p-Value
Dieleman, 2009	0.819	0.365	1.837	-0.485	0.628
Kok, 2017	5.230	0.271	101.100	1.095	0.274
	1.170	0.279	4.906	0.215	0.830

Odds ratio and 95% CI

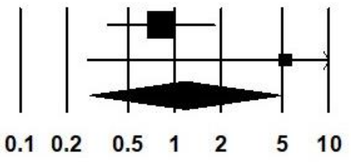

Hypertension

Study name	Odds ratio	Lower limit	Upper limit	Z-Value	p-Value
Dieleman, 2009	1.612	0.758	3.427	1.241	0.215
Lachmann, 2018	1.163	0.685	1.975	0.560	0.576
	1.296	0.840	1.998	1.171	0.241

Odds ratio and 95% CI

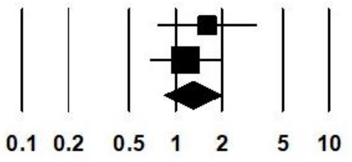

Variable	Forest Plot
**Pre-Operative (Continuous)**	

Age (years)

Study name	Difference in means	Standard error	Variance	Lower limit	Upper limit	Z-Value	p-Value
Dieleman, 2009	3.300	1.730	2.992	-0.090	6.690	1.908	0.056
Kok, 2017	-1.500	3.073	9.444	-7.523	4.523	-0.488	0.625
	1.572	2.304	5.308	-2.944	6.088	0.682	0.495

Difference in means and 95% CI

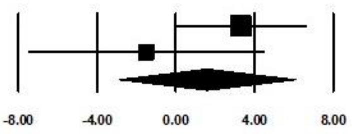

Variable	Forest Plot
**Intra-Operative (Continuous)**	

Number of grafts

Study name	Difference in means	Standard error	Variance	Lower limit	Upper limit	Z-Value	p-Value
Dieleman, 2009	0.047	0.192	0.037	-0.330	0.424	0.243	0.808
Kok, 2017	0.008	0.249	0.062	-0.480	0.496	0.034	0.973
	0.032	0.152	0.023	-0.266	0.331	0.213	0.832

Difference in means and 95% CI

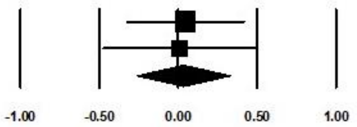

Supplement: Supplementary file 1 — Data S1 Tables S1–S9 Figures S1–S7 References 51 , 52 [file JAH3-9-e017275-s001.pdf]
